# Supplementary material for: Tritiation of aryl thianthrenium salts with a molecular palladium catalyst
Source: Nature. 2021 Dec 15;600(7889):444–9. doi: 10.1038/s41586-021-04007-y (PMC8674128; doi:10.1038/s41586-021-04007-y)
Supplement: Supplementary file 1 — This file contains detailed experimental procedures and spectroscopic data, and includes 11 supplementary tables and 33 supplementary figures. [file 41586_2021_4007_MOESM1_ESM.pdf]

nature portfolio

https://doi.org/10.1038/s41586-021-04007-y

Supplementary information

Tritiation of aryl thianthrenium salts with a molecular palladium catalyst

In the format provided by the authors and unedited

SUPPLEMENTARY INFORMATION

Tritiation of aryl thianthrenium salts with a molecular palladium catalyst

Da Zhao[1], Roland Petzold[1], Jiyao Yan[1,2], Dieter Muri[3], Tobias Ritter[1]*

[1] Max-Planck-Institut für Kohlenforschung, Kaiser-Wilhelm-Platz 1, D-45470 Mülheim an der Ruhr, Germany

[2] Institute of Organic Chemistry, RWTH Aachen University, Landoltweg 1, 52074 Aachen, Germany.

[3] Pre-clinical CMC, Roche Pharma Research and Early Development, Roche Innovation Center Basel, 4070 Basel, Switzerland

*E-mail: ritter@kofo.mpg.de

CONTENTS

MATERIALS AND METHODS 11

Solvents 11

Chromatography 11

Spectroscopy and Instruments 11

Starting materials 12

EXPERIMENTAL DATA 13

Representative procedure for hydrogenolysis with $^1H_2$ and $^2H_2$ 13

Representative procedure for hydrogenolysis in a J-Young NMR tube 14

Representative procedure for the thianthrenation of arenes 15

Reaction optimization for hydrogenolysis of aryl thianthrenium salts 16

Table S1. Optimization of yield as a function of catalyst 16

Table S2. Optimization of yield as a function of ligand 16

Table S3. Optimization of yield as a function of solvent 18

Table S4. Optimization of yield as a function of base 19

Table S5. Optimization of yield as a function of concentration 19

Table S6. Comparison of different counterions 20

Representative procedure for hydrogenolysis under low pressures of $^2H_2$ 21

Table S7. Hydrogenolysis of aryl thianthrenium salts under different total pressures of $^2H_2$ 22

Table S8. Additive effect 22

Table S9. Superior catalytic performance of Pd[(P*t*Bu$_3$)]$_2$ in the preparation of deuterated famoxadone and boscalid. 23

Table S10. Effect of excess phosphine ligand in hydrogenolysis. 23

Hydrogenolysis of aryl thianthrenium salts in the presence of water 24

Hydrogenolysis of aryl thianthrenium salts on small scales 26

Effect of solubility in the hydrogenolysis of coordinating-group-containing aryl thianthrenium salt 28

Palladium-catalyzed hydrogenolysis of aryl (pseudo)halides 30

Table S11. Evaluation of additional electron-rich, monodentate phosphine ligands in hydrogenolysis of aryl (pseudo) halides 31

Palladium-catalyzed hydrogenolysis of aryl bromide in the presence of silver(I) 32

Mechanistic studies 34

Kinetic reaction profile of the reaction 34

Determination of the reaction order in catalyst 37

Analysis of the reaction order in catalyst 39

Kinetic H/D isotope effect analysis 40

Influence of H_2 pressure on the rate of hydrogenolysis 42

Determination of the reaction order in leaving group 43

Mercury poison test 44

Reaction of aryl palladium(II) bromide with H_2 46

Coordinating ability comparison of thianthrene leaving group and triflate anion 47

Infrared spectroscopy study 48

Reaction of T-shaped arylpalladium(II) triflate **S-3** with $^2H_2$ 50

Thianthrenation of arenes 50

2-Nitro-biphenyl-derived tetrafluorothianthrenium salt (**19-TFT**) 50

2,2,2-Trichloroethyl (4-phenylbutyl)carbamate (**24**) 52

2,2,2-Trichloroethyl (4-phenylbutyl)carbamate-derived thianthrenium salt (**24-TT**) 52

LHVS-derived thianthrenium salt (**26-TT**) 53

(2-Bromoethyl)benzene-derived thianthrenium salt (**27-TT1**) 55

Styrene-derived thianthrenium salt (**27-TT**) 56

Etofenprox-derived tetrafluorothianthrenium salt (**29-TFT$_2$**) 57

Benazepril methyl ester-derived thianthrenium salts (**30-TT** and **30-TT1**) 58

Hydrogenolysis of aryl (tetrafluoro)thianthrenium salts 60

4-[^{2}H]-Biphenyl (**[^{2}H]1**) 60

4-[^{2}H]-Fluorobenzene (**[^{2}H]2**) 61

2-Fluoro-(*p*-[^{2}H]phenoxy)benzonitrile (**[^{2}H]3**) 62

3-Phenyl-3-(4-[^{2}H]-phenyl) propan-1-ol (**[^{2}H]4**) 64

4-[^{2}H]-Benzyloxazolidinone (**[^{2}H]5**) 65

[^{2}H]Amiodarone (**[^{2}H]6**) 66

4-[^{2}H]-Chlorobenzene (**[^{2}H]7**) 67

1-(4-Bromophenoxy)-4-[^{2}H]-benzene (**[^{2}H]8**) 68

N-(4-[^{2}H]-Phenyl)benzamide (**[^{2}H]9**) 69

[^{2}H]Nefiracetam (**[^{2}H]10**) 70

[^{2}H](±)-Pyriproxyfen (**[^{2}H]11**) 71

4'-[^{2}H]-[1,1'-Biphenyl]-4-yl trifluoromethanesulfonate (**[^{2}H]12**) 72

[^{2}H]-Tetrahydrobenzofuranone (**[^{2}H]13**) 73

4-[^{2}H]-Acetylmethylalanate (**[^{2}H]14**) 74

[^{2}H]Indomethacin methylester (**[^{2}H]15**) 75

[^{2}H](±)Famoxadone (**[^{2}H]16**) 76

2-[^{2}H]-3-Methyl-5-acetyl thiophene (**[^{2}H]17**) 77

1-Phenyl-4-[^{2}H]-1H-pyrazole (**[^{2}H]18**) 78

2-Nitro-4'-[^{2}H]-biphenyl (**[^{2}H]19**) 79

[^{2}H]Strychnine (**[^{2}H]20**) 80

[^{2}H]Salicin pentahydrate (**[^{2}H]21**) 82

2-Cyano-5-[^{2}H]-6-methoxyquinoline (**[^{2}H]22**) ... 83

5-[^{2}H]-2-Methoxybenzaldehyde (**[^{2}H]23**) ... 84

2,2,2-Trichloroethyl (4-(4'-[^{2}H]phenyl)butyl)carbamate (**[^{2}H]24**) ... 85

[^{2}H]Boscalid (**[^{2}H]25**) ... 86

[^{2}H]LHVS (**[^{2}H]26**) ... 87

[^{2}H]Styrene (**[^{2}H]27**) ... 88

[^{2}H]Fenofibrate (**[^{2}H]28**) ... 89

[^{2}H]Etofenprox (**[^{2}H]29**) ... 90

[$^2H_2$]Etofenprox (**[$^2H_2$]29**) ... 92

[^{2}H]Benazepril methylester triflimide adduct (**[^{2}H]30**) ... 93

Reductive tritiation of aryl (tetrafluoro)thianthrenium salts ... 95

General information ... 95

[^{3}H]Nefiracetam (**[^{3}H]10**) ... 96

[^{3}H](±)-Pyriproxyfen (**[^{3}H]11**) ... 97

[^{3}H]Indomethacin methylester (**[^{3}H]15**) ... 98

[^{3}H]LHVS (**[^{3}H]26**) ... 100

[$^3H_2$]Etofenprox (**[$^3H_2$]29**) ... 101

SPECTROSCOPIC DATA ... 104

^{1}H NMR of 2-nitro-biphenyl-derived tetrafluorothianthrenium salt (**19-TFT**) ... 104

^{13}C NMR of 2-nitro-biphenyl-derived tetrafluorothianthrenium salt (**19-TFT**) ... 105

^{19}F NMR of 2-nitro-biphenyl-derived tetrafluorothianthrenium salt (**19-TFT**) ... 106

^{1}H NMR of 2,2,2-trichloroethyl (4-phenylbutyl)carbamate (**24**) ... 107

^{13}C NMR of 2,2,2-trichloroethyl (4-phenylbutyl)carbamate (**24**) ... 108

^{1}H NMR of 2,2,2-trichloroethyl (4-phenylbutyl)carbamate-derived thianthrenium salt (**24-TT**)......109

^{13}C NMR of 2,2,2-trichloroethyl (4-phenylbutyl)carbamate-derived thianthrenium salt (**24-TT**) 110

^{19}F NMR of 2,2,2-trichloroethyl (4-phenylbutyl)carbamate-derived thianthrenium salt (**24-TT**).....111

^{1}H NMR of LHVS-derived thianthrenium salt (**26-TT**) 112

^{13}C NMR of LHVS-derived thianthrenium salt (**26-TT**)..113

^{19}F NMR of LHVS-derived thianthrenium salt (**26-TT**) 114

^{1}H NMR of (2-bromoethyl)benzene-derived thianthrenium salt (**27-TT1**) 115

^{13}C NMR of (2-bromoethyl)benzene-derived thianthrenium salt (**27-TT1**)..............................116

^{19}F NMR of (2-bromoethyl)benzene-derived thianthrenium salt (**27-TT1**) 117

^{1}H NMR of styrene-derived thianthrenium salt (**27-TT**)..118

^{13}C NMR of styrene-derived thianthrenium salt (**27-TT**) 119

^{19}F NMR of styrene-derived thianthrenium salt (**27-TT**)...120

^{1}H NMR of etofenprox-derived tetrafluorothianthrenium salt (**29-TFT$_2$**)..............................121

^{13}C NMR of etofenprox-derived tetrafluorothianthrenium salt (**29-TFT$_2$**) 122

^{19}F NMR of etofenprox-derived tetrafluorothianthrenium salt (**29-TFT$_2$**)..............................123

^{1}H NMR of benazepril methyl ester-derived thianthrenium salt (**30-TT**) 124

^{13}C NMR of benazepril methyl ester-derived thianthrenium salt (**30-TT**)..............................125

^{19}F NMR of benazepril methyl ester-derived thianthrenium salt (**30-TT**) 126

NOESY spectrum of benazepril methyl ester-derived thianthrenium salt (**30-TT**)........................127

^{1}H NMR of benazepril methyl ester-derived thianthrenium salt (**30-TT1**) 128

^{13}C NMR of benazepril methyl ester-derived thianthrenium salt (**30-TT1**)............................129

^{19}F NMR of benazepril methyl ester-derived thianthrenium salt (**30-TT1**) 130

NOESY spectrum of benazepril methyl ester-derived thianthrenium salt (**30-TT1**)......................131

^{1}H NMR of 4-[^{2}H]-biphenyl (**[^{2}H]1**)....132

^{2}H NMR of 4-[^{2}H]-biphenyl (**[^{2}H]1**)....133

^{13}C NMR of 4-[^{2}H]-biphenyl (**[^{2}H]1**)....134

^{1}H NMR of 2-fluoro-(*p*-[^{2}H]phenoxy)benzonitrile (**[^{2}H]3**)....135

^{2}H NMR of 2-fluoro-(*p*-[^{2}H]phenoxy)benzonitrile (**[^{2}H]3**)....136

^{13}C NMR of 2-fluoro-(*p*-[^{2}H]phenoxy)benzonitrile (**[^{2}H]3**)....137

^{19}F NMR of 2-fluoro-(*p*-[^{2}H]phenoxy)benzonitrile (**[^{2}H]3**)....138

^{1}H NMR of 3-phenyl-3-(4-[^{2}H]-phenyl) propan-1-ol (**[^{2}H]4**)....139

^{2}H NMR of 3-phenyl-3-(4-[^{2}H]-phenyl) propan-1-ol (**[^{2}H]4**)....140

^{13}C NMR of 3-phenyl-3-(4-[^{2}H]-phenyl) propan-1-ol (**[^{2}H]4**)....141

^{1}H NMR of 4-[^{2}H]-benzyloxazolidinone (**[^{2}H]5**)....142

^{2}H NMR of 4-[^{2}H]-benzyloxazolidinone (**[^{2}H]5**)....143

^{13}C NMR of 4-[^{2}H]-benzyloxazolidinone (**[^{2}H]5**)....144

^{1}H NMR of [^{2}H]amiodarone (**[^{2}H]6**)....145

^{2}H NMR of [^{2}H]amiodarone (**[^{2}H]6**)....146

^{13}C NMR of [^{2}H]amiodarone (**[^{2}H]6**)....147

^{1}H NMR of 1-(4-bromophenoxy)-4-[^{2}H]-benzene (**[^{2}H]8**)....148

^{2}H NMR of 1-(4-bromophenoxy)-4-[^{2}H]-benzene (**[^{2}H]8**)....149

^{13}C NMR of 1-(4-bromophenoxy)-4-[^{2}H]-benzene (**[^{2}H]8**)....150

^{1}H NMR of *N*-(4-[^{2}H]-phenyl)benzamide (**[^{2}H]9**)....151

^{2}H NMR of *N*-(4-[^{2}H]-phenyl)benzamide (**[^{2}H]9**)....152

^{13}C NMR of *N*-(4-[^{2}H]-phenyl)benzamide (**[^{2}H]9**)....153

^{1}H NMR of [^{2}H]nefiracetam (**[^{2}H]10**)....154

^{2}H NMR of [^{2}H]nefiracetam (**[^{2}H]10**) 155

^{13}C NMR of [^{2}H]nefiracetam (**[^{2}H]10**) 156

^{1}H NMR of [^{2}H](±)-pyriproxyfen (**[^{2}H]11**) 157

^{2}H NMR of [^{2}H](±)-pyriproxyfen (**[^{2}H]11**) 158

^{13}C NMR of [^{2}H](±)-pyriproxyfen (**[^{2}H]11**) 159

^{1}H NMR of 4'-[^{2}H]-[1,1'-biphenyl]-4-yl trifluoromethanesulfonate (**[^{2}H]12**) 160

^{2}H NMR of 4'-[^{2}H]-[1,1'-biphenyl]-4-yl trifluoromethanesulfonate (**[^{2}H]12**) 161

^{13}C NMR of 4'-[^{2}H]-[1,1'-biphenyl]-4-yl trifluoromethanesulfonate (**[^{2}H]12**) 162

^{19}F NMR of 4'-[^{2}H]-[1,1'-biphenyl]-4-yl trifluoromethanesulfonate (**[^{2}H]12**) 163

^{1}H NMR of [^{2}H]-tetrahydrobenzofuranone (**[^{2}H]13**) 164

^{2}H NMR of [^{2}H]-tetrahydrobenzofuranone (**[^{2}H]13**) 165

^{13}C NMR of [^{2}H]-tetrahydrobenzofuranone (**[^{2}H]13**) 166

^{1}H NMR of 4-[^{2}H]-acetylmethylalanate (**[^{2}H]14**) 167

^{2}H NMR of 4-[^{2}H]-acetylmethylalanate (**[^{2}H]14**) 168

^{13}C NMR of 4-[^{2}H]-acetylmethylalanate (**[^{2}H]14**) 169

^{1}H NMR of [^{2}H]indomethacin methylester (**[^{2}H]15**) 170

^{2}H NMR of [^{2}H]indomethacin methylester (**[^{2}H]15**) 171

^{13}C NMR of [^{2}H]indomethacin methylester (**[^{2}H]15**) 172

^{1}H NMR of [^{2}H](±)-famoxadone (**[^{2}H]16**) 173

^{2}H NMR of [^{2}H](±)-famoxadone (**[^{2}H]16**) 174

^{13}C NMR of [^{2}H](±)-famoxadone (**[^{2}H]16**) 175

^{1}H NMR of 2-[^{2}H]-3-methyl-5-acetyl thiophene (**[^{2}H]17**) 176

^{2}H NMR of 2-[^{2}H]-3-methyl-5-acetyl thiophene (**[^{2}H]17**) 177

^{13}C NMR of 2-[^{2}H]-3-methyl-5-acetyl thiophene (**[^{2}H]17**) 178

^{1}H NMR of 1-phenyl-4-[^{2}H]-1H-pyrazole (**[^{2}H]18**)....179

^{2}H NMR of 1-phenyl-4-[^{2}H]-1H-pyrazole (**[^{2}H]18**)....180

^{13}C NMR of 1-phenyl-4-[^{2}H]-1H-pyrazole (**[^{2}H]18**) 181

^{1}H NMR of 2-nitro-4'-[^{2}H]-biphenyl (**[^{2}H]19**)....182

^{2}H NMR of 2-nitro-4'-[^{2}H]-biphenyl (**[^{2}H]19**)....183

^{13}C NMR of 2-nitro-4'-[^{2}H]-biphenyl (**[^{2}H]19**) 184

^{1}H NMR of [^{2}H]strychnine (**[^{2}H]20**) 185

^{2}H NMR of [^{2}H]strychnine (**[^{2}H]20**) 186

^{13}C NMR of [^{2}H]strychnine (**[^{2}H]20**)....187

^{1}H NMR of [^{2}H]salicin pentahydrate (**[^{2}H]21**) 188

^{2}H NMR of [^{2}H]salicin pentahydrate (**[^{2}H]21**) 189

^{13}C NMR of [^{2}H]salicin pentahydrate (**[^{2}H]21**) 190

^{1}H NMR of 2-cyano-5-[^{2}H]-6-methoxyquinoline (**[^{2}H]22**)....191

^{2}H NMR of 2-cyano-5-[^{2}H]-6-methoxyquinoline (**[^{2}H]22**)....192

^{13}C NMR of 2-cyano-5-[^{2}H]-6-methoxyquinoline (**[^{2}H]22**) 193

^{1}H NMR of 5-[^{2}H]-2-methoxybenzaldehyde (**[^{2}H]23**)....194

^{2}H NMR of 5-[^{2}H]-2-methoxybenzaldehyde (**[^{2}H]23**)....195

^{13}C NMR of 5-[^{2}H]-2-methoxybenzaldehyde (**[^{2}H]23**) 196

^{1}H NMR of 2,2,2-trichloroethyl (4-(4’-[^{2}H]phenyl)butyl)carbamate (**[^{2}H]24**) 197

^{2}H NMR of 2,2,2-trichloroethyl (4-(4’-[^{2}H]phenyl)butyl)carbamate (**[^{2}H]24**) 198

^{13}C NMR of 2,2,2-trichloroethyl (4-(4’-[^{2}H]phenyl)butyl)carbamate (**[^{2}H]24**)....199

^{1}H NMR of [^{2}H]boscalid (**[^{2}H]25**) 200

^{2}H NMR of [^{2}H]boscalid (**[^{2}H]25**) 201

^{13}C NMR of [^{2}H]boscalid (**[^{2}H]25**) 202

^{1}H NMR of [^{2}H]LHVS (**[^{2}H]26**) 203

^{2}H NMR of [^{2}H]LHVS (**[^{2}H]26**) 204

^{13}C NMR of [^{2}H]LHVS (**[^{2}H]26**) 205

^{1}H NMR of [^{2}H]fenofibrate (**[^{2}H]28**) 206

^{2}H NMR of [^{2}H]fenofibrate (**[^{2}H]28**) 207

^{13}C NMR of [^{2}H]fenofibrate (**[^{2}H]28**) 208

^{1}H NMR of [^{2}H]etofenprox (**[^{2}H]29**) 209

^{2}H NMR of [^{2}H]etofenprox (**[^{2}H]29**) 210

^{13}C NMR of [^{2}H]etofenprox (**[^{2}H]29**) 211

^{1}H NMR of [$^2H_2$]etofenprox (**[$^2H_2$]29**) 212

^{2}H NMR of [$^2H_2$]etofenprox (**[$^2H_2$]29**) 213

^{13}C NMR of [$^2H_2$]etofenprox (**[$^2H_2$]29**) 214

^{1}H NMR of [$^2H_2$]benazepril methylester triflimide adduct (**[^{2}H]30**) 215

^{2}H NMR of [$^2H_2$]benazepril methylester triflimide adduct (**[^{2}H]30**) 216

^{13}C NMR of [$^2H_2$]benazepril methylester triflimide adduct (**[^{2}H]30**) 217

^{19}F NMR of [$^2H_2$]benazepril methylester triflimide adduct (**[^{2}H]30**) 218

REFERENCES 219

MATERIALS AND METHODS

All air- and moisture-insensitive reactions were carried out under ambient atmosphere and monitored by thin-layer chromatography (TLC). Concentration under reduced pressure was performed by rotary evaporation at 25–40 °C at an appropriate pressure. Purified compounds were further dried under high vacuum (0.010–0.005 mbar). Yields refer to purified and spectroscopically pure compounds. All air- and moisture-sensitive manipulations were performed using oven-dried glassware (120 °C for a minimum of 12 hours) and standard Schlenk techniques under an atmosphere of argon.

Solvents

Anhydrous THF was obtained from Phoenix Solvent Drying Systems. All other deuterated solvents were purchased from Eur*iso*-Top.

Chromatography

Thin layer chromatography (TLC) was performed using EMD TLC plates pre-coated with 250 μm thickness silica gel 60 F_{254} plates and visualized by fluorescence quenching under 254 nm UV light, permanganate stain, cerium ammonium molybdate stain, or phosphomolybdic acid stain. Flash chromatography was performed using silica gel (40–63 μm particle size) purchased from Geduran. Preparatory high-performance liquid chromatographic separation was executed on a Shimadzu Prominence Preparative HPLC system with an YMC-Triart C18 HPLC column.

Spectroscopy and Instruments

NMR spectra were recorded on a Bruker Ascend™ 500 spectrometer operating at 500 MHz, 126 MHz, and 471 MHz for ^{1}H, ^{13}C, and ^{19}F acquisitions, respectively. ^{2}H NMR spectra were recorded on a Bruker AVANCE 600a spectrometer operating at 92 MHz. Chemical shifts are reported in ppm with the solvent resonance as the internal standard. For ^{1}H NMR: $CDCl_3$, δ 7.26; CD_2Cl_2, δ 5.32; CD_3CN, δ 1.94; CD_3OD, δ 3.31; THF-d_8, δ 3.58, 1.72. For ^{13}C NMR: $CDCl_3$, δ 77.16; CD_2Cl_2, δ 53.84; CD_3CN, δ 1.32, 118.26; CD_3OD, δ 49.00. For ^{2}H NMR: $CHCl_3$, δ 7.26; CH_2Cl_2, δ 5.32; CH_3CN, δ 1.94; CH_3OH, δ 3.31. ^{19}F NMR chemical shifts are reported with an added compound as the internal standard: 2-fluorobenzotrifluoride, δ –117.3; 4-fluorobenzotrifluoride, δ –109.4; 4,4'-difluorobenzophenone, δ –108.6 ppm. Data is reported as follows: s = singlet, d = doublet, t = triplet, q = quartet, m = multiplet, br = broad; coupling constants in Hz; integration[1].

Radiochemical purity was determined using a Radiomatic 150 TR radioflow detector with PerkinElmer UltimaFlo liquid scintillation cocktail. LC/MS of tritiated compounds were performed on an Agilent 6130 quadruple LC/MS with an Agilent 1260 infinity HPLC operating in the ES^+ ionization mode. HPLC analyses were performed on a Waters 2995 Alliance HPLC with a Waters 2996 PDA Detector. Infrared spectroscopy was recorded on a Thermo Scientific Nicolet iS5 FT-IR spectrophotometer.

Starting materials

All substrates and reagents were used as received from commercial suppliers or prepared according to published procedures, respectively, unless otherwise stated. Deuterium gas was purchased from Air Liquide and used without further treatments. K_3PO_4 was stored in an argon-filled glovebox after being dried at 200 °C for more than 48 h under high-vacuum. Aryl(tetrafluoro)thianthrenium salts were prepared according to published procedures[2,3]. 4-Biphenyl triflate[4] and 4-biphenyldiazonium tetrafluoroborate[5] were synthesized according to previously reported procedures.

EXPERIMENTAL DATA

Representative procedure for hydrogenolysis with $^1H_2$ and $^2H_2$

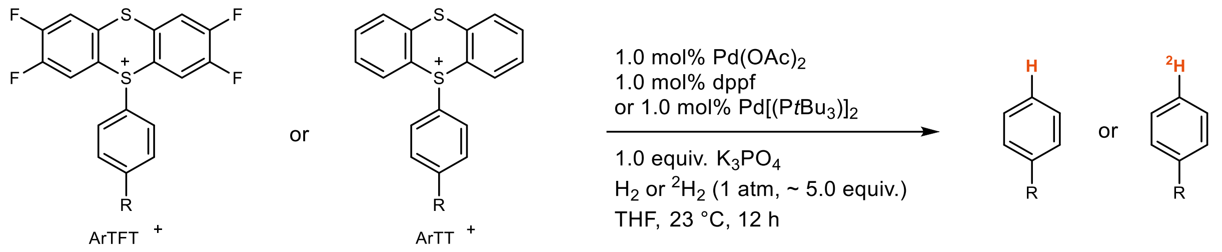

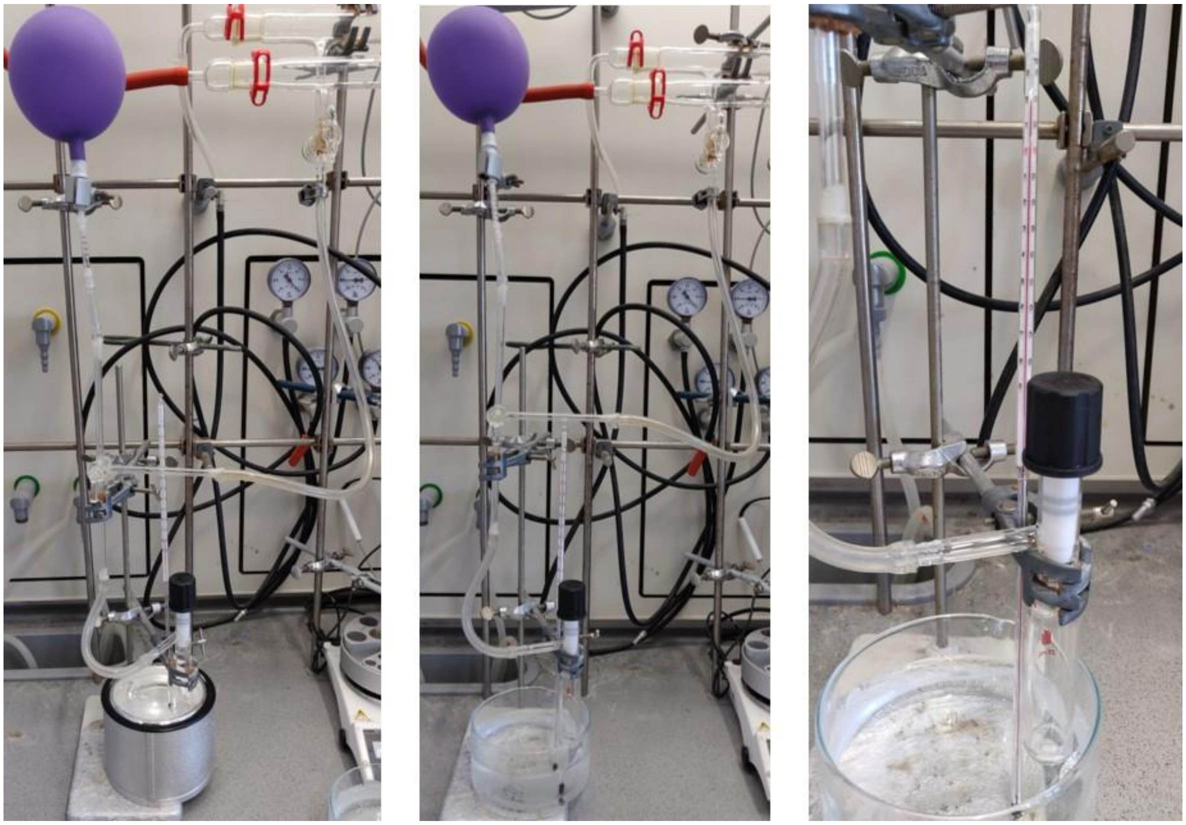

Figure S1. Reaction setup in a Schlenk tube.

Aryl thianthrenium salt (0.200 mmol, 1.00 equiv.), K_3PO_4 (42.5 mg, 0.200 mmol, 1.00 equiv.), and THF (0.5 mL, c = 0.2 M) were added to a 25-mL Schlenk tube containing a magnetic stir bar. Subsequently, a stock THF solution (0.5 mL) containing $Pd(OAc)_2$ (0.5 mg, 2 μmol, 1 mol%) and dppf (1.1 mg, 2.0 μmol, 1.0 mol%) or Pd[(P*t*Bu$_3$)]$_2$ (1.0 mg, 2.0 μmol, 1.0 mol%) was added to the reaction mixture. The Schlenk tube was then connected to a high vacuum line and a balloon containing H_2 or $^2H_2$ (1 atm) via a T-bore glass stopcock adaptor (Figure S1). The reaction mixture was degassed via three freeze-pump-thaw cycles. After the third freeze-pump-thaw cycle, H_2 or $^2H_2$ (1 atm) was introduced to the Schlenk tube while keeping the bottom of the

Schlenk tube submersed in a water bath (23 °C). After the Schlenk tube was warmed to 23 °C, the tube was sealed, and the reaction mixture was stirred vigorously at 23 °C. After 12 hours, the vessel was opened to air, and CH_2Cl_2 was added to the reaction mixture (5 mL). The resulting mixture was concentrated by rotary evaporation. The residue was purified by chromatography on silica gel to afford the desired product.

Representative procedure for hydrogenolysis in a J-Young NMR tube

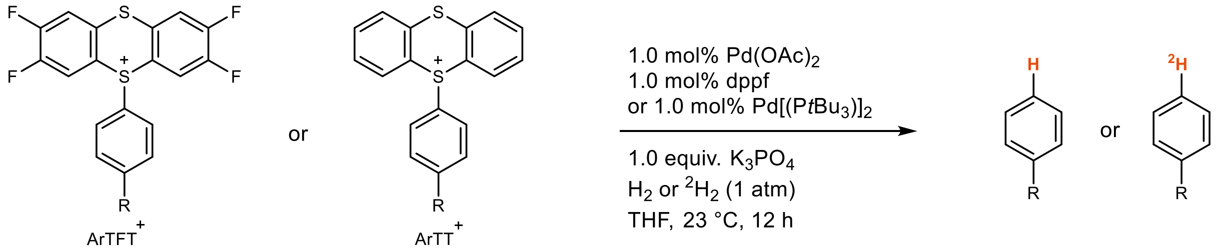

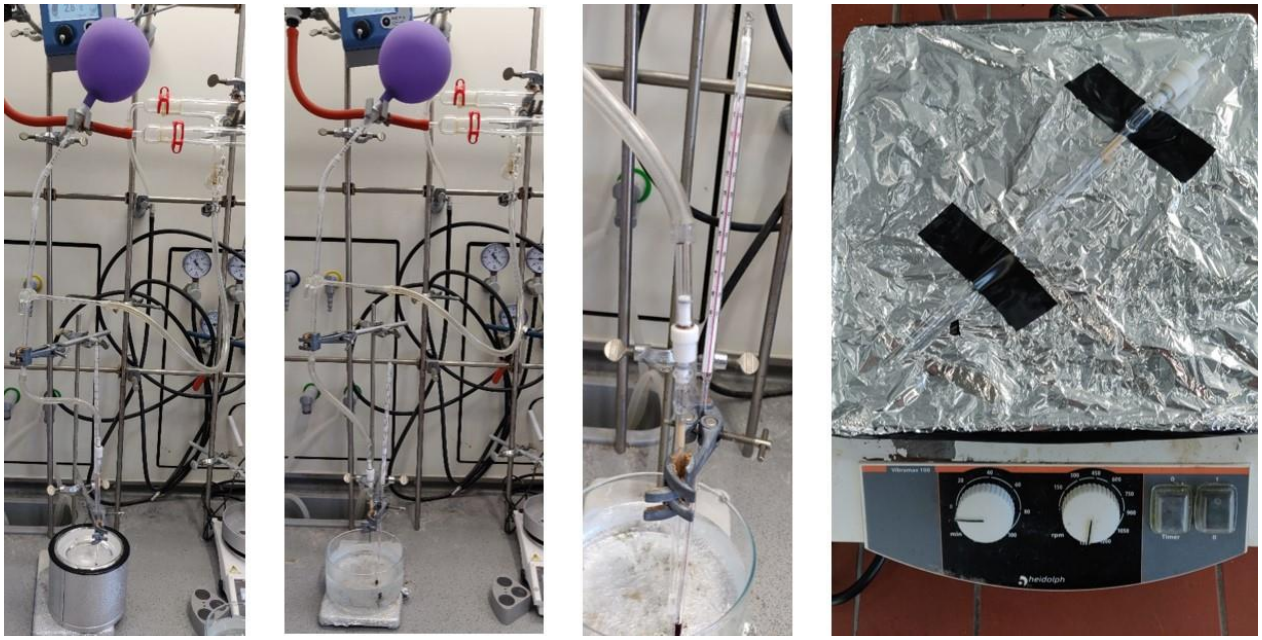

Figure S2. Reaction setup in a J-Young NMR tube.

Aryl thianthrenium salt (1.00 equiv.) and K_3PO_4 (1.00 equiv.) were added to a J-Young NMR tube. Subsequently, a stock THF solution (0.5 mL) containing $Pd(OAc)_2$ (1.0 mol%) and dppf (1.0 mol%) or $Pd[(P\textit{t}Bu_3)]_2$ (1.0 mol%) was added to the reaction mixture. The J-Young NMR tube was then connected to a high vacuum line and a balloon containing H_2 or $^2H_2$ (1 atm) via a T-bore glass stopcock adaptor (Figure S2). The reaction mixture was degassed via three freeze-pump-thaw cycles. After the third freeze-pump-thaw cycle, H_2 or $^2H_2$ (1 atm) was introduced to the NMR tube while keeping the bottom of the NMR tube submersed in a water bath (23 °C).

After the NMR tube was warmed to 23 °C, the NMR tube was sealed, and the reaction mixture was shaken vigorously at 23 °C on a circular vibrating shaker (Heidolph Vibramax 100). After 12 hours, the NMR tube was opened to air, and CH_2Cl_2 (5 mL) was added. The resulting mixture was concentrated by rotary evaporation and analyzed by NMR or mass analysis.

Representative procedure for the thianthrenation of arenes

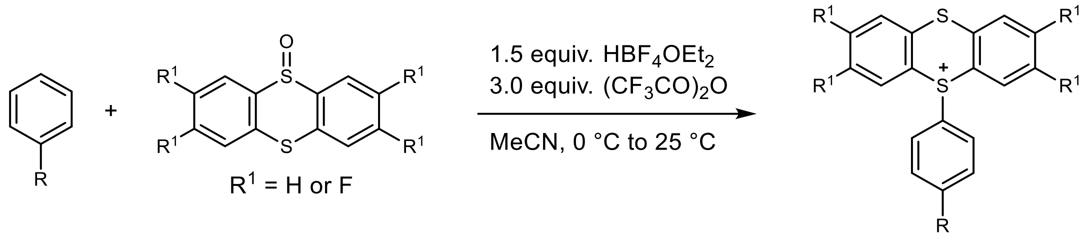

Under an ambient atmosphere, a 20 mL glass vial was charged with arene (0.50 mmol, 1.0 equiv.), (tetrafluoro)thianthrenium-*S*-oxide (0.50 mmol, 1.0 equiv.), and dry MeCN (2.0 – 4.0 mL, c = 0.13 – 0.25 M). After cooling to 0 °C, $HBF_4 \cdot OEt_2$ (1.5 equiv. + 1.0 equiv. per basic functional group) was added to the vial while stirring the reaction mixture. Other acids may be used instead of $HBF_4 \cdot OEt_2$ like triflic acid (TfOH). For acid sensitive substrates $BF_3 \cdot OEt_2$ or trimethylsilyltriflate (TMSOTf) can be used as well. Subsequently, trifluoroacetic anhydride (1.5 mmol, 3.0 equiv.) was added in one portion at 0 °C, resulting in a color change to deep purple. The vial was sealed with a screw-cap. The mixture was stirred at 0 °C for 1 h and then at 25 °C until the intensity of the purple color decreased. The solution was concentrated and the residue was diluted with 5 mL dichloromethane and poured onto a mixture of 30 mL dichloromethane, 20 mL saturated aqueous Na_2CO_3 solution, and 10 mL water. After stirring for 5 min at 25 °C, the mixture was poured into a separatory funnel, and the layers were separated. The dichloromethane layer was washed with aqueous $NaBF_4$ solution (2 × ca. 20 mL, 5 % w/w) and with water (2 × ca. 20 mL). Washing with $NaBF_4$ solution is only required if it is of interest that the product contains only one type of counterion, solutions containing other ions, like hexafluorophosphate or bis(trifluoromethanesulfonyl)amide may be used as well. The dichloromethane layer was dried over $MgSO_4$, filtered, and the solvent was removed under reduced pressure. In order to obtain analytically pure samples of thianthrenium salts, the residue was purified by chromatography on silica gel eluting with dichloromethane & *i*-PrOH, subsequently, the product was dissolved in 2 mL dichloromethane and precipitated with 20 mL Et_2O. The solid was dried in vacuo to afford the (tetrafluoro)thianthrenium salt.

Reaction optimization for hydrogenolysis of aryl thianthrenium salts

Table S1. Optimization of yield as a function of catalyst

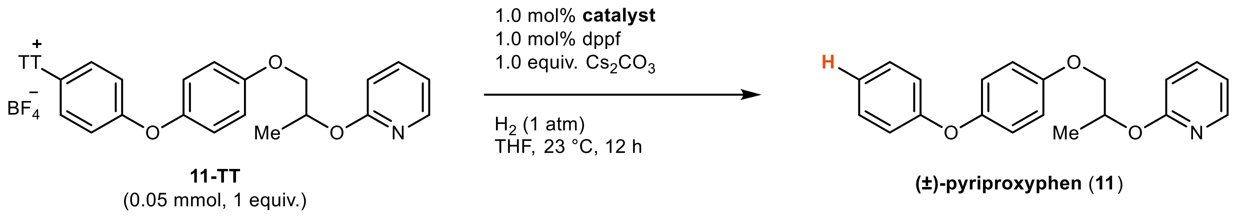

Entry	Catalyst	Yield[a]
1	$Pd(OAc)_2$	93%
2	No catalyst	0%
3	$PdCl_2$	<5%
4	$Pd(dba)_2$	28%
5	Pd[(P*t*Bu)$_3$]$_2$	61%
6	$Ni(COD)_2$ + 1.0 mol% bipyridine instead of dppf	0%

[a]Yield was determined by ^{1}H NMR using dibromomethane as an internal standard.

Table S2. Optimization of yield as a function of ligand

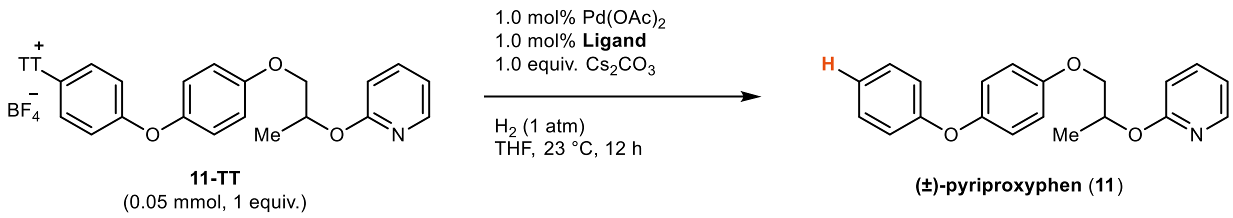

Entry	Ligand	Yield[a]
1	dppf	93%
2	PPh_3	49%
3	tris(2-furanyl)phosphine	81%
4	*t*BuXphos	71%
5	RuPhos	65%
6	dppe	80%
7	DPEphos	76%
8	dippf	64%
9	Xantphos	80%
10	Sphos	56%
11	dppbz	34%
12	tris(*p*-chlorophenyl)phosphine	62%
13	tris(4-methoxyphenyl)phosphine	41%
14	$P\textit{t}Bu_3\cdot HBF_4$	72%
15	Ipr	8%
16	$1\text{-}AdP\textit{t}Bu_2$	92%[b]

[a]Yield was determined by ^{1}H NMR using dibromomethane as an internal standard. [b]Isolated yield.

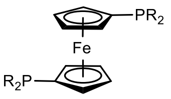

R = Ph, dppf;
R = *i*Pr, dippf

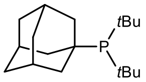

1-AdP*t*Bu_2

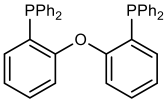

DPEphos

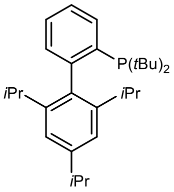

*t*BuXPhos

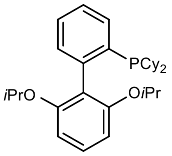

RuPhos

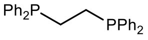

dppe

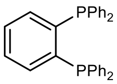

dppbz

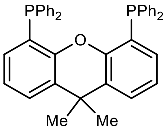

Xantphos

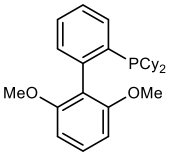

SPhos

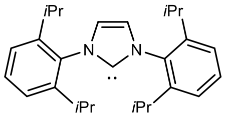

Ipr

Table S3. Optimization of yield as a function of solvent

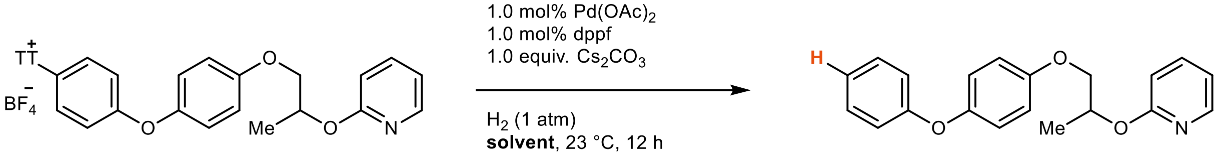

11-TT
(0.05 mmol, 1 equiv.)

(±)-pyriproxyphen (**11**)

Entry	Solvent	Yield[a]
1	THF	93%
2	MeCN	0%
3	DMF	0%
4	DMSO	0%
5	Dioxane	46%
6	PhCl	52%
7	Tol	25%
8	DCM	0%

[a]Yield was determined by ^{1}H NMR using dibromomethane as an internal standard.

Table S4. Optimization of yield as a function of base

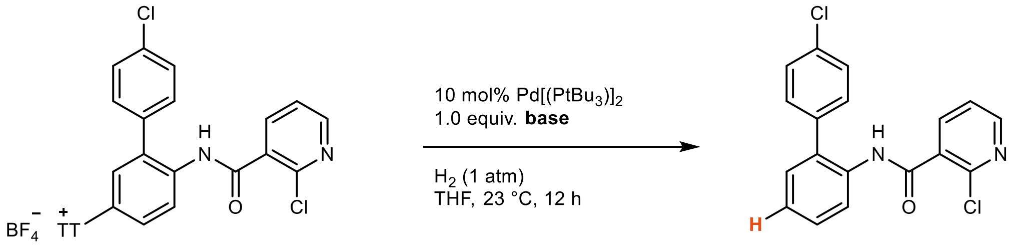

25-TT
(0.05 mmol, 1 equiv.)

boscalid (**25**)

Entry	Base	Yield[a]
1	K_3PO_4	99%
2	Cs_2CO_3	0%
3	$NaHCO_3$	0%
4	K_2HPO_4	62%
5	KOH	0%
6	DABCO	41%
7	MTBD	0%
8	pyridine	0%
9	CsF	0%
10	DIPEA	0%

[a]Yield was determined by ^{1}H NMR using dibromomethane as an internal standard.

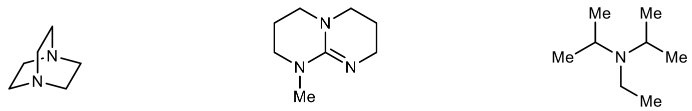

DABCO MTBD DIPEA

Table S5. Optimization of yield as a function of concentration

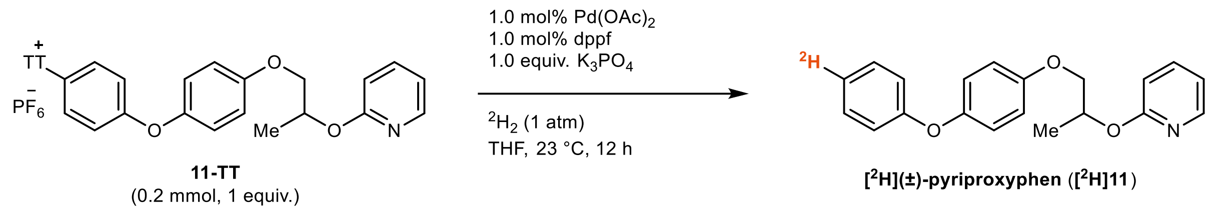

Entry	Concentration	Yield[a], ^{2}H-incorporation[b]
1	0.2 M	98%, >99%
2	0.4 M	93%, >99%

[a]Yield was determined by ^{1}H NMR using dibromomethane as an internal standard. [b]^{2}H-incorporation was determined by ^{1}H NMR by integrating the resonance at 7.12 ppm of the product and comparison to the internal standard dibromomethane with a resonance at 5.09 ppm.

Table S6. Comparison of different counterions

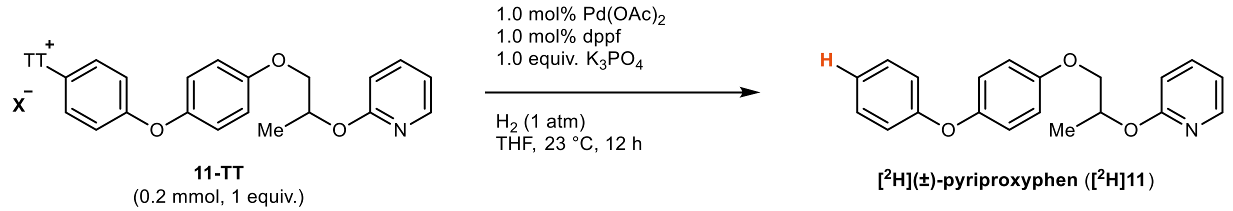

Entry	Counterions	Yield[a]
1	PF_6	98%
2	BF_4	93%

[a]Yield was determined by ^{1}H NMR using dibromomethane as an internal standard.

Representative procedure for hydrogenolysis under low pressures of $^2H_2$

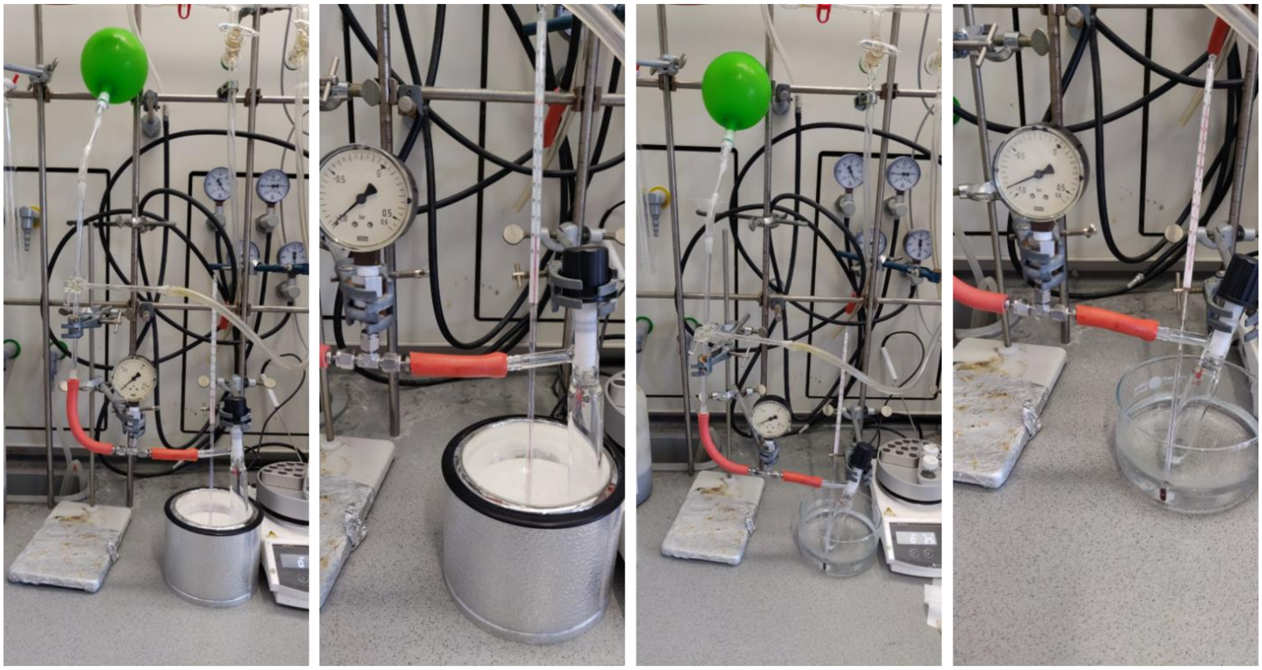

Figure S3. Reaction setup under low pressures of $^2H_2$.

Aryl thianthrenium salt (0.200 mmol, 1.00 equiv.), K_3PO_4 (42.5 mg, 0.200 mmol, 1.00 equiv.), and THF (0.5 mL, c = 0.2 M) were added to a 25-mL Schlenk tube containing a magnetic stir bar. Subsequently, a stock THF solution (0.5 mL) containing $Pd(OAc)_2$ (0.5 mg, 2 µmol, 1 mol%) and dppf (1.1 mg, 2.0 µmol, 1.0 mol%) or $Pd[(P\textit{t}Bu_3)]_2$ (1.0 mg, 2.0 µmol, 1.0 mol%) was added to the reaction mixture. The Schlenk tube was then connected to a high vacuum line and a balloon containing $^2H_2$ (1 atm) via a low pressure gas gauge attached to a T-bore glass stopcock adaptor (Figure S3). The reaction mixture was degassed via three freeze-pump-thaw cycles. After the third freeze-pump-thaw cycle, subatmospheric pressure $^2H_2$ (indicated by the low pressure gas gauge) was introduced to the Schlenk tube while keeping the bottom of the Schlenk tube submersed in a water bath (23 °C). After the Schlenk tube was warmed to 23 °C, the tube was sealed, and the reaction mixture was stirred vigorously at 23 °C. After 12 hours, the reaction vessel was opened to air, and CH_2Cl_2 (5 mL) was added to the reaction mixture was added to the reaction mixture. The resulting mixture was concentrated by rotary evaporation. The residue was purified by chromatography on silica gel to afford the desired product. The ^{2}H-incorporation was determined by ^{1}H NMR. ^{2}H-incorporation was determined by ^{1}H NMR by integrating the resonance at 7.12 ppm of the product and comparison to the internal standard dibromomethane with a resonance at 5.09 ppm.

Table S7. Hydrogenolysis of aryl thianthrenium salts under different total pressures of $^2H_2$

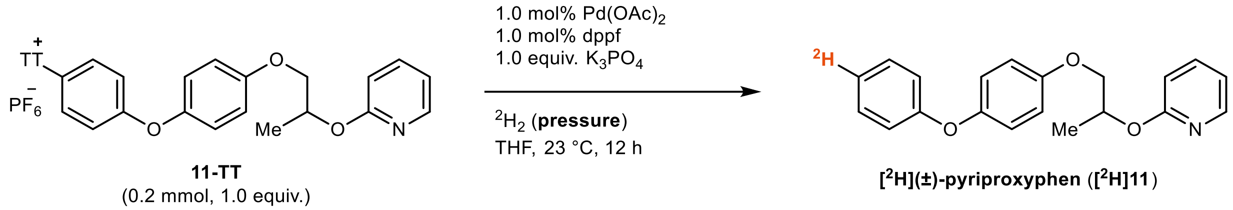

Entry	Total pressure of $^2H_2$	Yield[a], ^{2}H-incorporation[b]
1	1 atm	98%, >99%
2	0.6 atm	95%, >99%
3	0.1 atm	97%, >99%

[a]Yield was determined by ^{1}H NMR using dibromomethane as an internal standard. [b]^{2}H-incorporation was determined by ^{1}H NMR by integrating the resonance at 7.12 ppm of the product and comparison to the internal standard dibromomethane with a resonance at 5.09 ppm.

Table S8. Additive effect

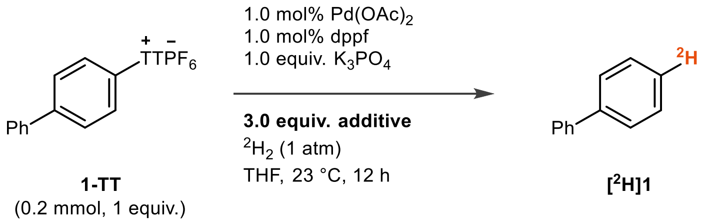

Entry	Additive	Yield[a], ^{2}H-incorporation[b]
1	none	87%, >99%
2	KCl	<2%, N.D.
3	KBr	<2%, N.D.
4	KOTf	29%, >99%
5	KPF_6	85%, >99%

[a]Yield was determined by ^{1}H NMR using mesitylene as an internal standard. [b]^{2}H-incorporation was determined by ^{1}H NMR by integrating the resonance at 7.40 ppm of the product and comparison to the

internal standard mesitylene with a resonance at 6.79 ppm. N.D., not determined.

Table S9. Superior catalytic performance of Pd[(P*t*Bu₃)]₂ in the preparation of deuterated famoxadone and boscalid.

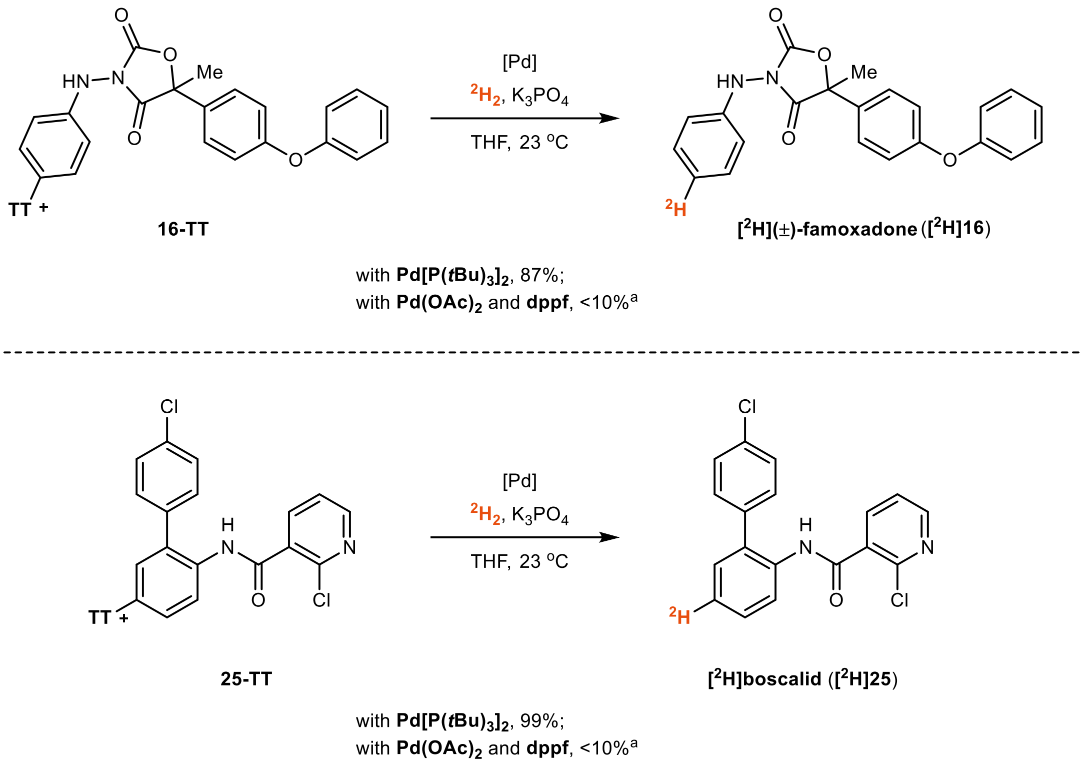

[a]Yield was determined by ^{1}H NMR using mesitylene as an internal standard.

Table S10. Effect of excess phosphine ligand in hydrogenolysis.

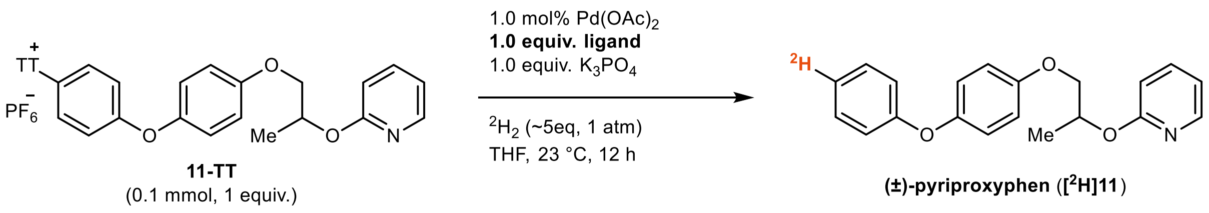

Entry	Ligand	Yield[a]
1	dppf	9%
2	P*t*Bu₃	0%

[a]Yield was determined by ^{1}H NMR using mesitylene as an internal standard.

Hydrogenolysis of aryl thianthrenium salts in the presence of water

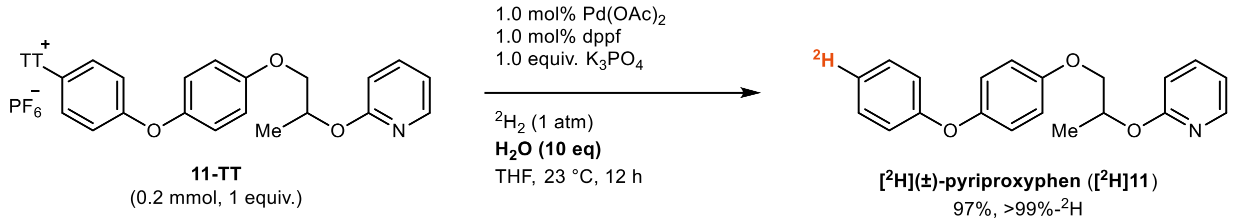

Pyriproxyfen-derived thianthrenium salt **11-TT** (136 mg, 0.200 mmol, 1.00 equiv.), K_3PO_4 (42.5 mg, 0.200 mmol, 1.00 equiv.), and water (36.0 mg, 36.0 µl, 2.00 mmol, 10.0 equiv.) were added to a 25-mL Schlenk tube containing a magnetic stir bar. Subsequently, a stock THF solution (1.0 mL, c = 0.2 M) containing $Pd(OAc)_2$ (0.5 mg, 2 µmol, 1 mol%) and dppf (1.1 mg, 2.0 µmol, 1.0 mol%) was added to the reaction mixture. The Schlenk tube was then connected to a high vacuum line and a balloon containing $^2H_2$ (1 atm) via a T-bore glass stopcock adaptor (Figure S1). The reaction mixture was degassed via three freeze-pump-thaw cycles. After the third freeze-pump-thaw cycle, $^2H_2$ (1 atm) was introduced to the Schlenk tube while keeping the bottom of the Schlenk tube submersed in a water bath (23 °C). After the Schlenk tube was warmed to 23 °C, the tube was sealed, and the reaction mixture was stirred vigorously at 23 °C. After 12 hours, the reaction vessel was opened to air, and CH_2Cl_2 (5 mL) was added to the reaction mixture. The resulting mixture was concentrated by rotary evaporation. CH_2Br_2 (69.5 mg, 28.1 µl, 0.200 mmol, 2.00 equiv.) was added as an internal standard. The resulting mixture was diluted with CD_3CN, and the ^1H NMR of the crude product mixture was recorded. The yield was determined by ^1H NMR by integrating the resonance at 6.72 ppm of the product and comparison to the internal standard CH_2Br_2 with a resonance at 5.09 ppm. The ^2H-incorporation of **11** was determined by ^1H NMR by integrating the resonance at 7.12 ppm of the product and comparison to the internal standard CH_2Br_2 with a resonance at 5.09 ppm (Figures S4 and S5).

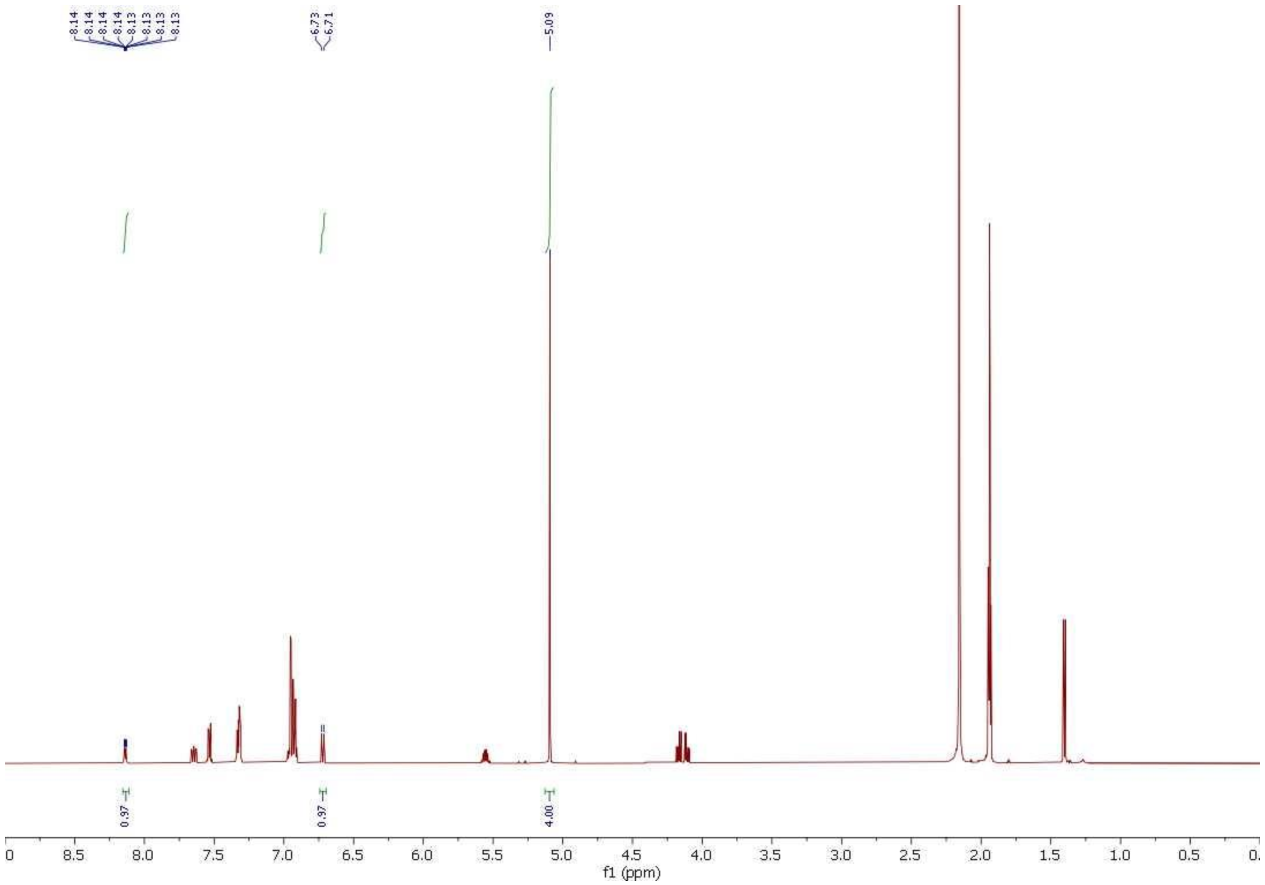

Figure S4. Crude ^{1}H NMR spectrum of the reaction mixture of **11-TT** with CH_2Br_2 as internal standard in CD_3CN.

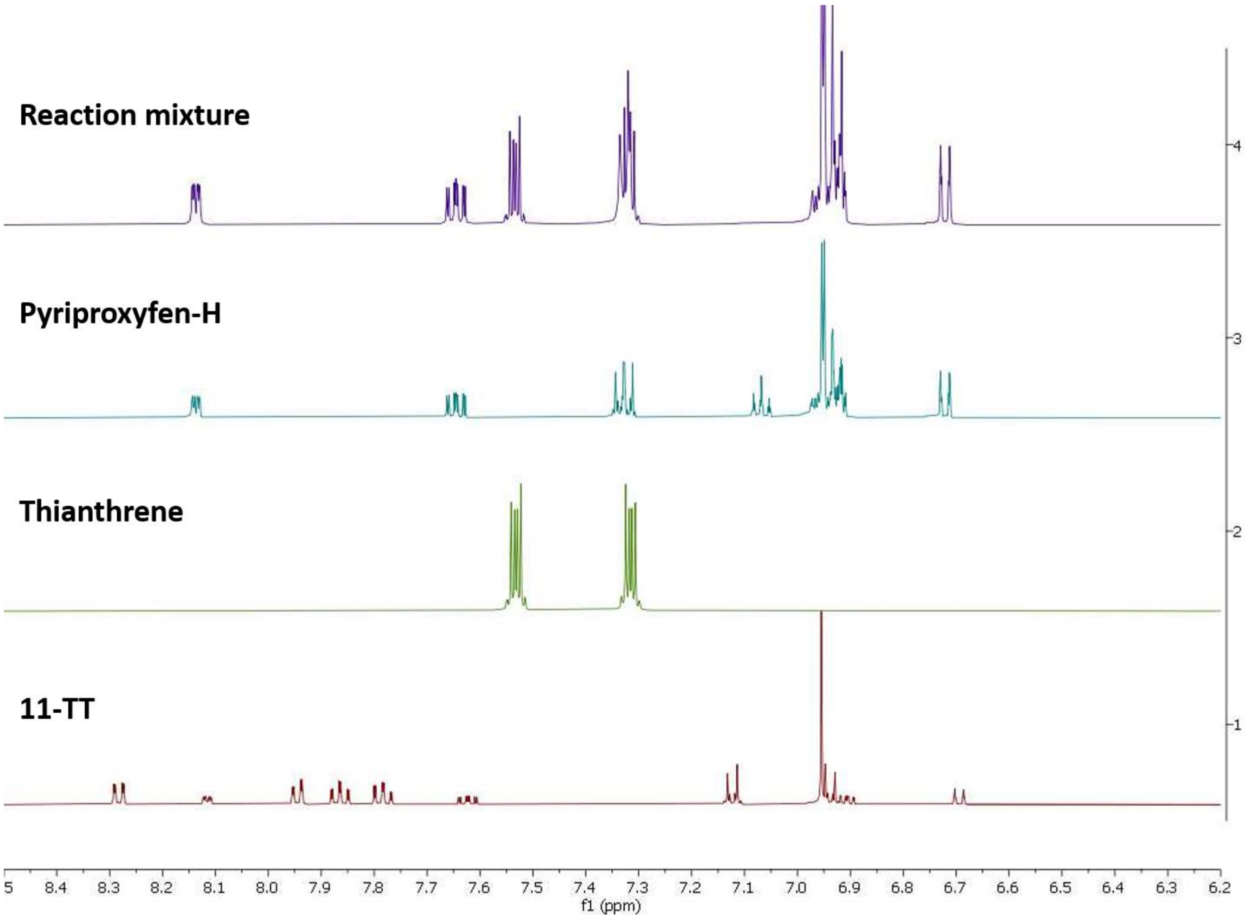

Figure S5. Comparison of ^{1}H NMR spectra of the reaction mixture of **11-TT**, pyriproxyfen-H, thianthrene and starting material **11-TT** in CD_3CN.

Hydrogenolysis of aryl thianthrenium salts on small scales

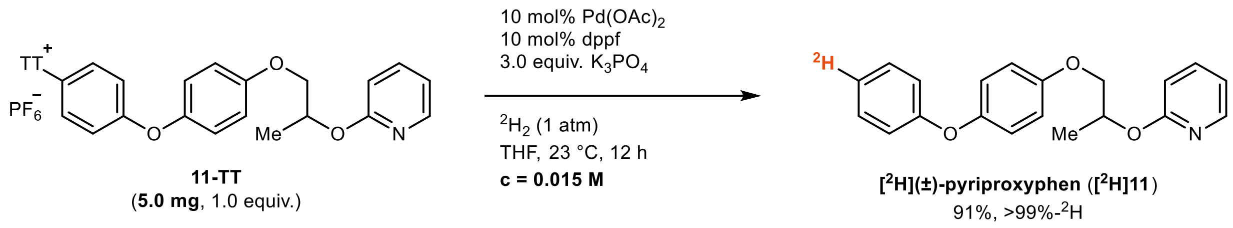

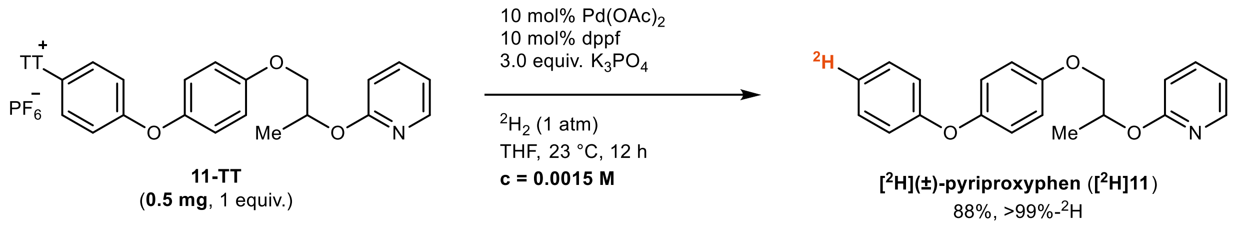

Pyriproxyfen-derived thianthrenium salt **11-TT** (0.5 mg or 5 mg, 1 equiv.) and K_3PO_4 (3.0 equiv.) were added to a J-Young NMR tube. Subsequently, a stock THF solution (0.5 mL) containing $Pd(OAc)_2$ (10 mol%) and dppf (10 mol%) was added to the reaction mixture. The J-Young NMR tube was then connected to a high vacuum line and a balloon containing $^2H_2$ (1 atm) via a T-bore glass stopcock adaptor (Figure S2). The reaction mixture was degassed via three freeze-pump-thaw cycles. After the third freeze-pump-thaw cycle, $^2H_2$ (1 atm) was introduced to the NMR tube while keeping the bottom of the NMR tube submersed in a water bath (23 °C). After the NMR tube was warmed to 23 °C, the NMR tube was sealed, and the reaction mixture was shaken vigorously at 23 °C on a circular vibrating shaker (Heidolph Vibramax 100). After 12 hours, the NMR tube was opened to air, and CH_2Cl_2 (5 mL) was added to the reaction mixture. The resulting mixture was concentrated by rotary evaporation. The resulting mixture was diluted with a stock CD_3CN solution containing CH_2Br_2 (2.5 equiv.) as an internal standard. The yield was determined by ^1H NMR by integrating the resonance at 6.72 ppm of the product and comparison to the internal standard CH_2Br_2 with a resonance at 5.09 ppm. The ^2H-incorporation of **11** was determined by ^1H NMR analysis by integrating the resonance at 7.12 ppm of the product and comparison to the internal standard CH_2Br_2 with a resonance at 5.09 ppm (Figures S6 and S7).

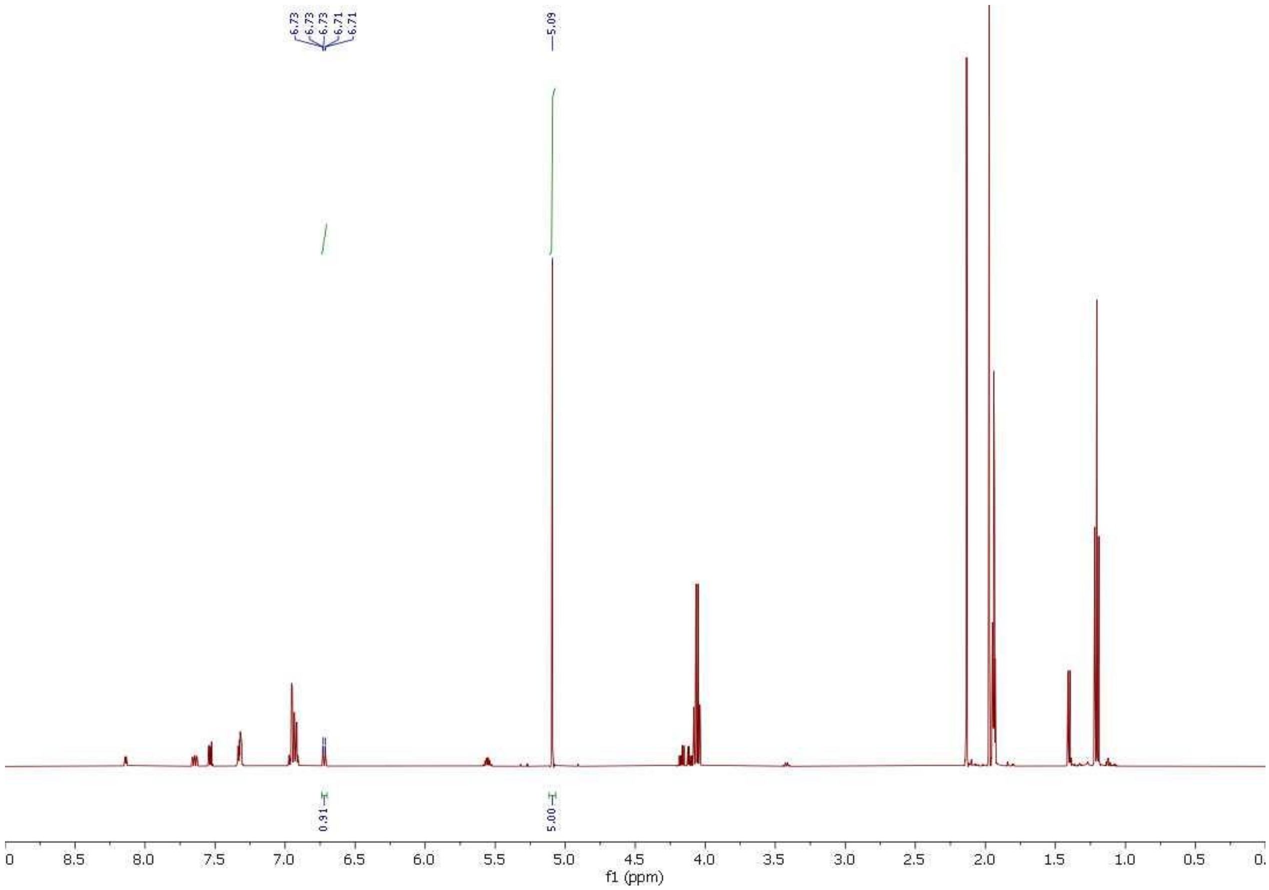

Figure S6. Crude ^{1}H NMR spectrum of the reaction mixture of **11-TT** (5.0 mg scale) with CH_2Br_2 as internal standard in CD_3CN.

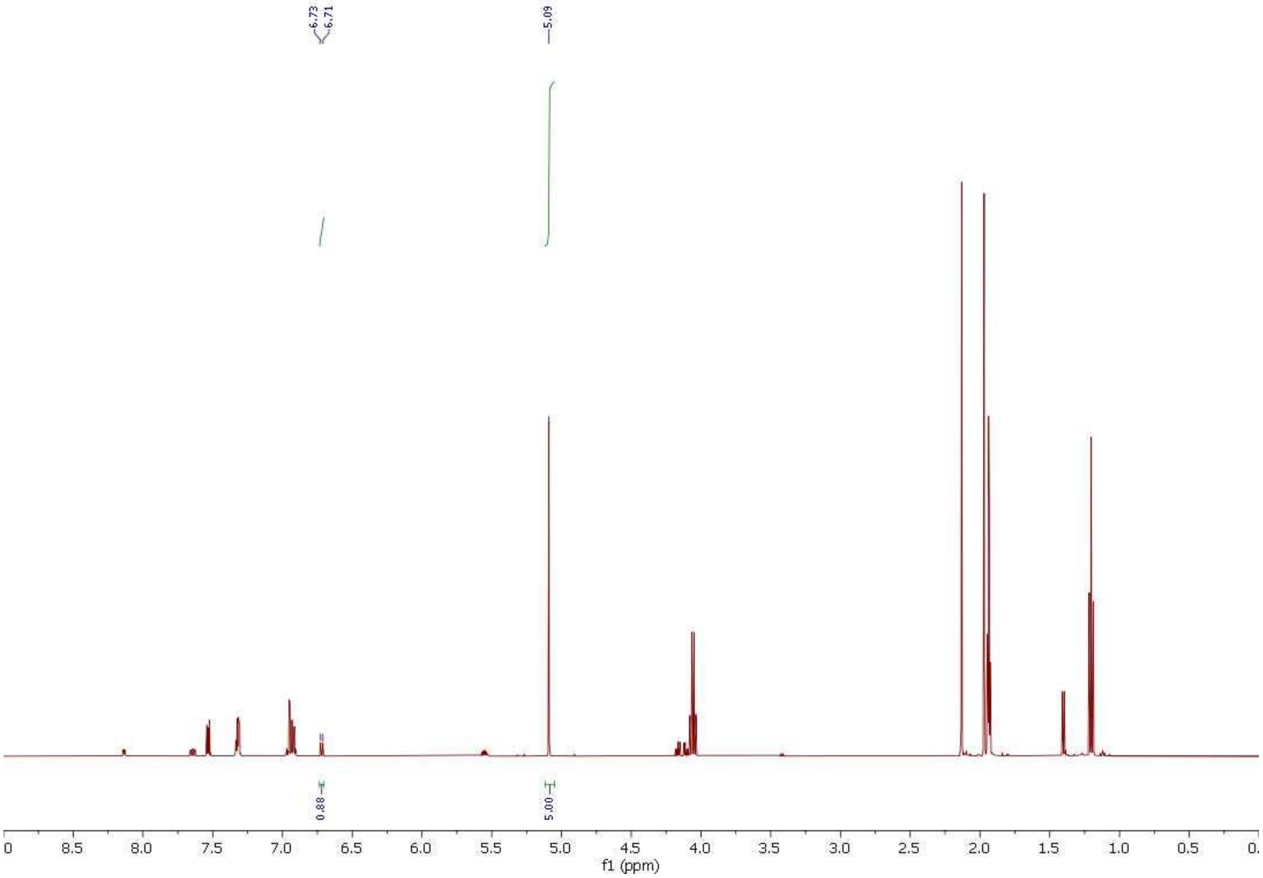

Figure S7. Crude ^{1}H NMR spectrum of the reaction mixture of **11-TT** (0.5 mg scale) with CH_2Br_2 as internal standard in CD_3CN.

Effect of solubility in the hydrogenolysis of coordinating-group-containing aryl thianthrenium salt

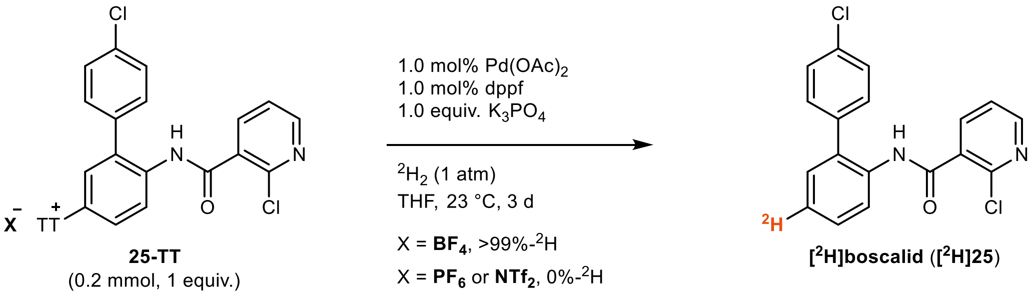

Boscalid-derived thianthrenium salt **25-TT** with different counterions (0.200 mmol, 1.00 equiv.) and K_3PO_4 (42.5 mg, 0.200 mmol, 1.00 equiv.) were added to a 25-mL Schlenk tube containing

a magnetic stir bar. Subsequently, a stock THF solution (1.0 mL, c = 0.2 M) containing $Pd(OAc)_2$ (0.5 mg, 2 μmol, 1 mol%) and dppf (1.1 mg, 2.0 μmol, 1.0 mol%) was added to the reaction mixture. The Schlenk tube was then connected to a high vacuum line and a balloon containing $^2H_2$ (1 atm) via a T-bore glass stopcock adaptor (Figure S1). The reaction mixture was degassed via three freeze-pump-thaw cycles. After the third freeze-pump-thaw cycle, $^2H_2$ (1 atm) was introduced to the Schlenk tube while keeping the bottom of the Schlenk tube submersed in a water bath (23 °C). After the Schlenk tube was warmed to 23 °C, the tube was sealed, and the reaction mixture was stirred vigorously at 23 °C. After 3 days, the reaction vessel was opened to air, and CH_2Cl_2 (5 mL) was added to the reaction mixture. The resulting mixture was concentrated by rotary evaporation. The resulting mixture was concentrated by rotary evaporation. The residue was purified by chromatography on silica gel, eluting with EtOAc/pentane 1:3 (v/v) to afford boscalid **25** as a colorless solid. The ^{2}H-incorporation of **25** was determined by mass analysis. For the reaction of boscalid-derived thianthrenium salt **25-TT** with BF_4 as counterion, which has poor solubility in THF, >99%-^{2}H incorporation in the isolated product **25** were observed. Whereas for the reaction of boscalid-derived thianthrenium salt **25-TT** with PF_6 or NTF_2 as counterion, which has relatively better solubility than that with BF_4 as counterion, 0%-^{2}H incorporation in the isolated product **25** were observed.

These results indicate that the appropriate choice of counterion for the arylthianthrenium salt starting material can reduce its solubility in THF to the point that a lower effective concentration than active catalyst is present in the liquid phase, with the reservoir of starting material in the solid phase, leaching material for conversion as the reaction progresses.

Palladium-catalyzed hydrogenolysis of aryl (pseudo)halides

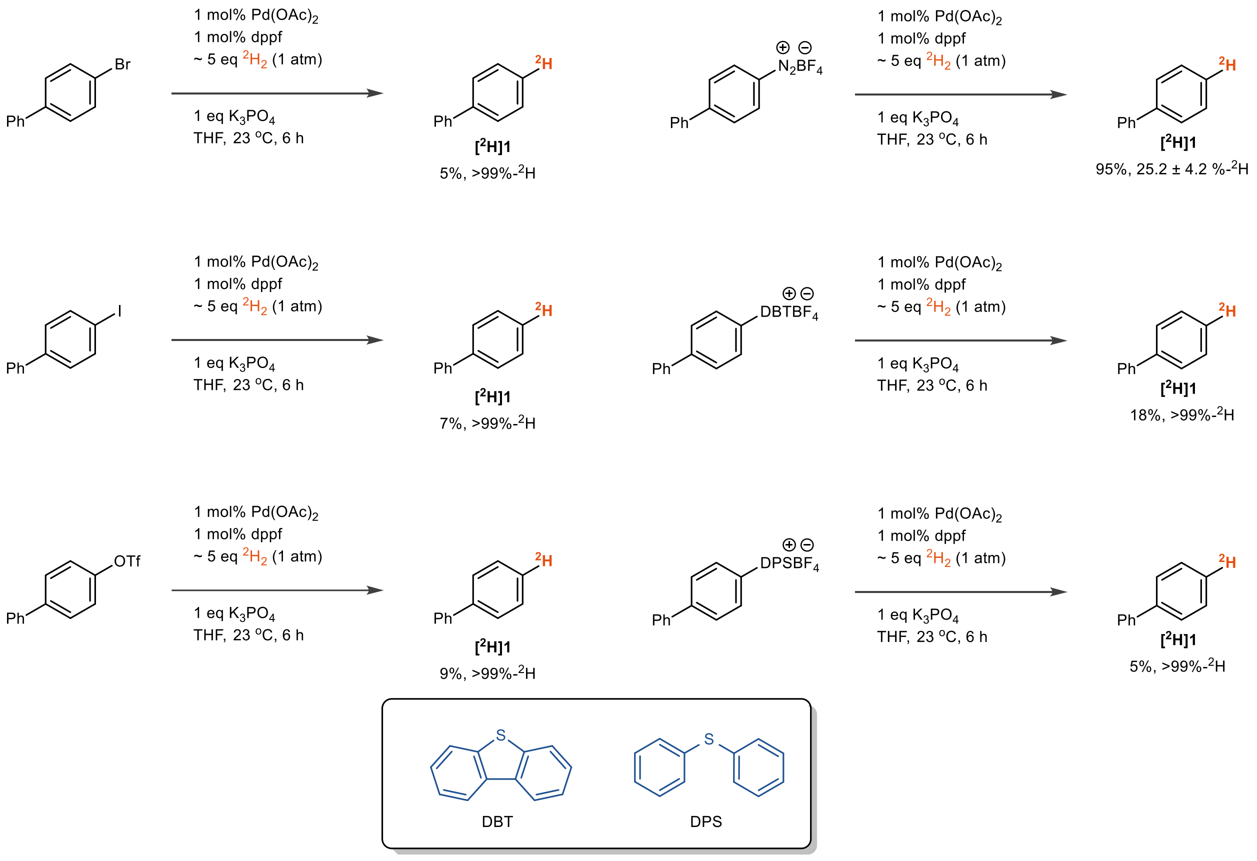

Scheme S1. Hydrogenolysis of aryl (pseudo)halides.

Biphenyl-derived (pseudo) halides (0.200 mmol, 1.00 equiv.), K_3PO_4 (42.5 mg, 0.200 mmol, 1.00 equiv.), and THF (0.5 mL, c = 0.2 M) were added to a 25-mL Schlenk tube containing a magnetic stir bar. Subsequently, a stock THF solution (0.5 mL) containing $Pd(OAc)_2$ (0.5 mg, 2 μmol, 1 mol%) and dppf (1.1 mg, 2.0 μmol, 1.0 mol%) was added to the reaction mixture. The Schlenk tube was then connected to a high vacuum line and a balloon containing $^2H_2$ (1 atm) via a T-bore glass stopcock adaptor (Figure S1). The reaction mixture was degassed via three freeze-pump-thaw cycles. After the third freeze-pump-thaw cycle, $^2H_2$ (1 atm) was introduced to the Schlenk tube while keeping the bottom of the Schlenk tube submersed in a water bath (23 °C). After the Schlenk tube was warmed to 23 °C, the tube was sealed, and the reaction mixture was stirred vigorously at 23 °C. After 12 hours, the reaction vessel was opened to air, and CH_2Cl_2 (5 mL) was added to the reaction mixture. The resulting mixture was concentrated by rotary evaporation. The residue was purified by chromatography on silica gel, eluting with EtOAc/hexanes 1:20 (v/v) to afford [^{2}H]-4-biphenyl as a colorless solid. The ^{2}H-incorporation was analyzed by mass analysis.

Note: *For hydrogenolysis of biphenyl-derived diazonium salt, before ^{2}H gas was introduced, the starting material decomposed to biphenyl rapidly in solution in the presence of palladium catalyst, thus lowering the ^{2}H-incorporation (25.2 ± 4.2 %-^{2}H).*

Table S11. Evaluation of additional electron-rich, monodentate phosphine ligands in hydrogenolysis of aryl (pseudo) halides

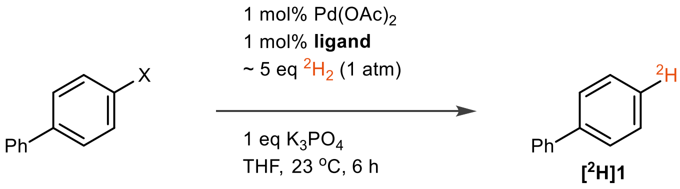

X	ligand	yield of $[^{2}H]1^{a}$
I	BrettPhos	<3%
	*t*BuXPhos	<3%
	AlPhos	<4%
Br	BrettPhos	<2%
	*t*BuXPhos	9%
	AlPhos	9%
OTf	BrettPhos	<1%
	*t*BuXPhos	<1%
	AlPhos	<1%

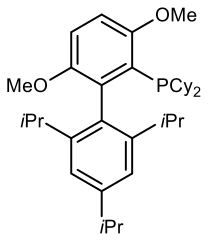

BrettPhos

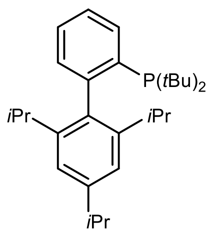

*t*BuXPhos

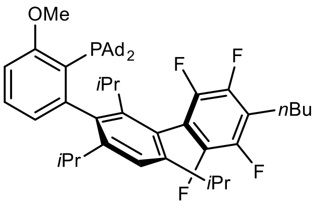

AlPhos

[a]Yield was determined by ^{1}H NMR using mesitylene as an internal standard.

Biphenyl-derived (pseudo) halides (0.200 mmol, 1.00 equiv.), K_3PO_4 (42.5 mg, 0.200 mmol, 1.00 equiv.), and THF (0.5 mL, c = 0.2 M) were added to a 25-mL Schlenk tube containing a magnetic stir bar. Subsequently, a stock THF solution (0.5 mL) containing $Pd(OAc)_2$ (0.5 mg, 2 μmol, 1 mol%) and electron-rich, monodentate phosphine ligand (2.0 μmol, 1.0 mol%) was added to the reaction mixture. The Schlenk tube was then connected to a high vacuum line and a balloon containing $^{2}H_2$ (1 atm) via a T-bore glass stopcock adaptor (Figure S1). The reaction mixture was degassed via three freeze-pump-thaw cycles. After the third freeze-pump-thaw cycle, $^{2}H_2$ (1 atm) was introduced to the Schlenk tube while keeping the bottom of the Schlenk tube submersed in a water bath (23 °C). After the Schlenk tube was warmed to 23 °C, the tube was sealed, and the reaction mixture was stirred vigorously at 23 °C. After 12 hours, the reaction vessel was opened to air, and CH_2Cl_2 (5 mL) was added to the reaction mixture. The resulting mixture was concentrated by rotary evaporation and diluted with a stock CD_3CN solution

containing mesitylene (1.0 equiv.) as an internal standard. The yield was determined by ^{1}H NMR. In all cases, the desired deuterated products was observed in <10% yields (Table S11).

The residue was purified by chromatography on silica gel, eluting with EtOAc/hexanes 1:20 (v/v) to afford [^{2}H]-4-biphenyl as a colorless solid. The ^{2}H-incorporation was analyzed by mass analysis.

Palladium-catalyzed hydrogenolysis of aryl bromide in the presence of silver(I)

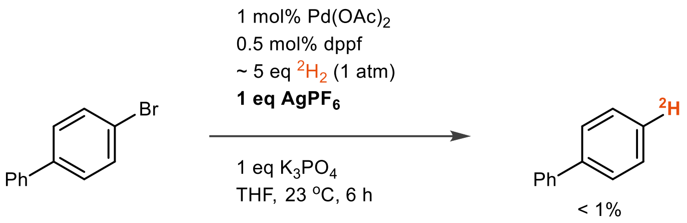

4-Bromobiphenyl (46.6 mg, 0.200 mmol, 1.00 equiv.), K_3PO_4 (42.5 mg, 0.200 mmol, 1.00 equiv.), and $AgPF_6$ (51.4 mg, 0.200 mmol, 1.00 equiv.) were added to a 25-mL Schlenk tube containing a magnetic stir bar. Subsequently, a stock THF solution (1.0 mL, c = 0.2 M) containing $Pd(OAc)_2$ (0.5 mg, 2 μmol, 1 mol%) and dppf (1.1 mg, 2.0 μmol, 1.0 mol%) was added to the reaction mixture. The Schlenk tube was then connected to a high vacuum line and a balloon containing $^2H_2$ (1 atm) via a T-bore glass stopcock adaptor (Figure S1). The reaction mixture was degassed via three freeze-pump-thaw cycles. After the third freeze-pump-thaw cycle, $^2H_2$ (1 atm) was introduced to the Schlenk tube while keeping the bottom of the Schlenk tube submersed in a water bath (23 °C). After the Schlenk tube was warmed to 23 °C, the tube was sealed, and the reaction mixture was stirred vigorously at 23 °C. After 12 hours, the reaction vessel was opened to air, and CH_2Cl_2 (5 mL) was added to the reaction mixture. The resulting mixture was concentrated by rotary evaporation. Mesitylene (48.0 mg, 56 μL, 0.400 mmol, 2.00 equiv.) was added as an internal standard. The resulting mixture was diluted with CD_2Cl_2, and the ^{1}H NMR of the crude product mixture was recorded, and only trace amount of the desired product (<1%) was observed (Figures S8 and S9).

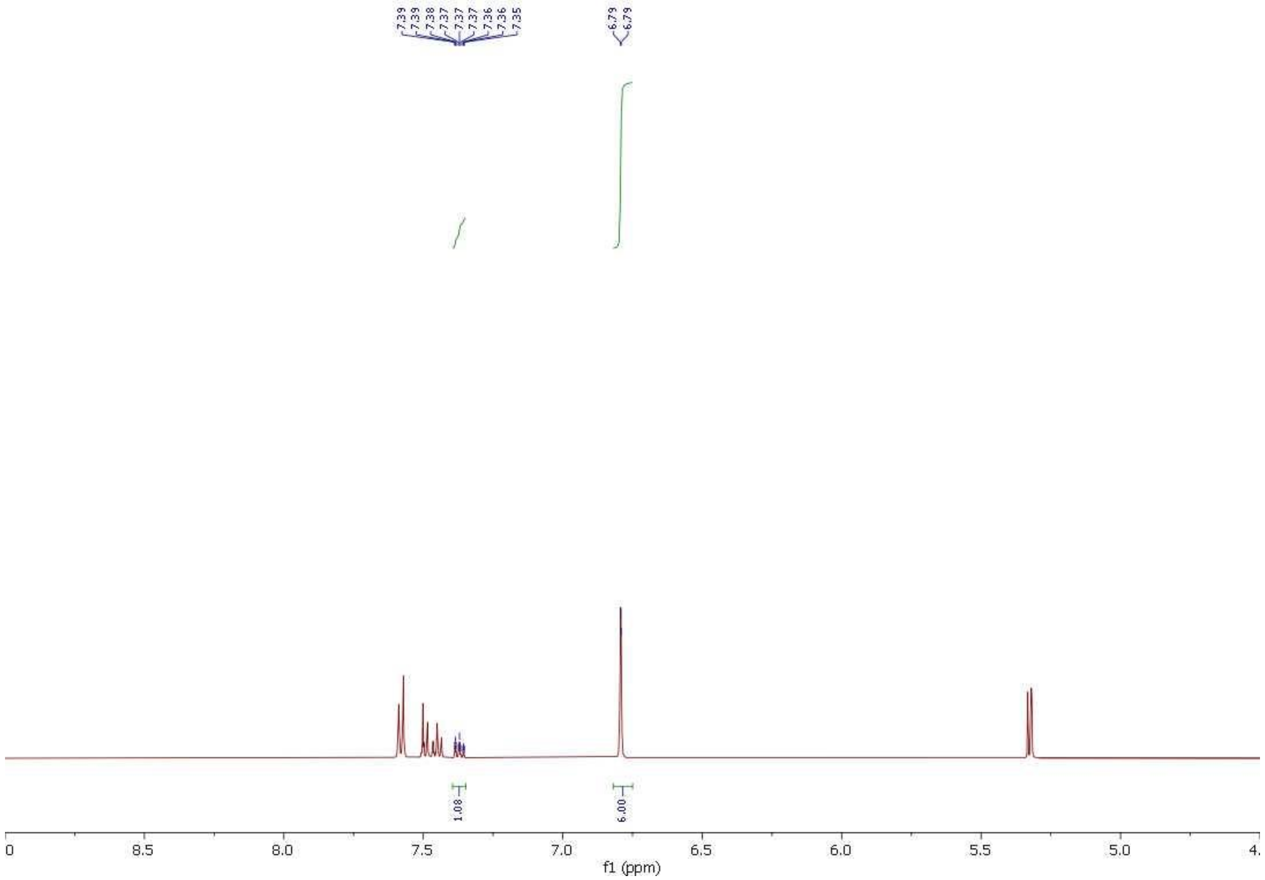

Figure S8. Crude ^{1}H NMR spectrum of the reaction mixture of 4-bromobiphenyl in the presence of silver(I) with mesitylene as internal standard in CD_2Cl_2.

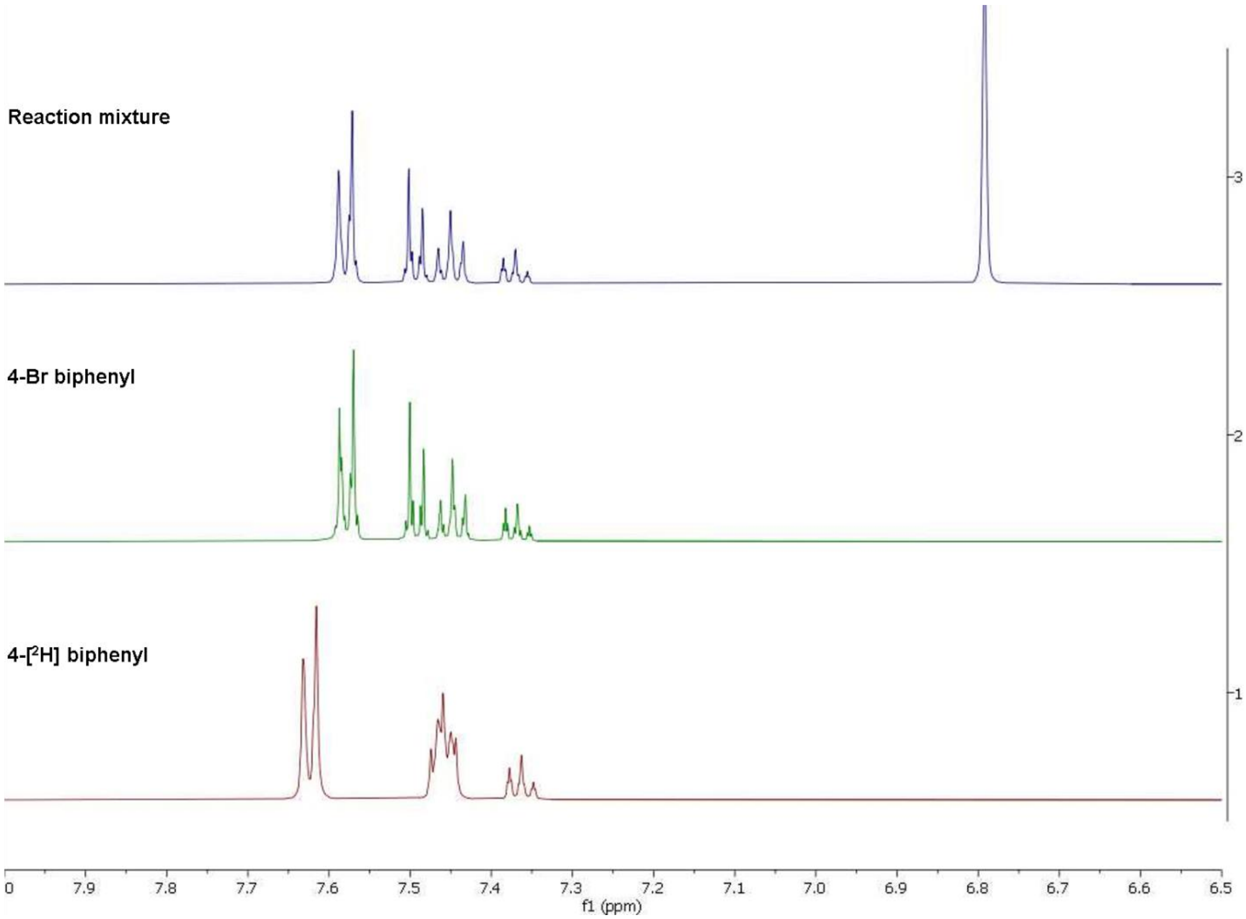

Figure S9. Comparison of ^{1}H NMR spectra of the reaction mixture of 4-bromobiphenyl in the presence of silver(I), starting material 4-bromobiphenyl and 4-[^{2}H] biphenyl in CD_2Cl_2.

Mechanistic studies

Kinetic reaction profile of the reaction

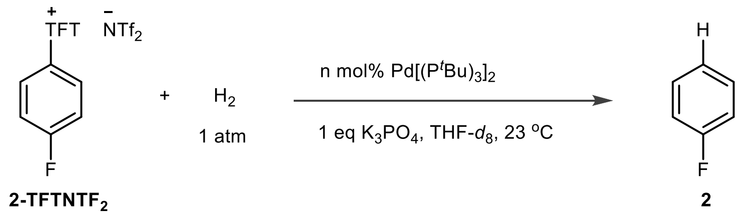

Fluorobenzene-derived tetrafluorothianthrenium salt **2-TFTNTF₂** (10.0 mg, 15.0 μmol, 1.00 equiv.) and K_3PO_4 (3.1 mg, 15 μmol, 1.0 equiv.) were added to a J-Young NMR tube. Subsequently, a stock THF-d_8 solution (0.5 mL) containing Pd[(P*t*Bu$_3$)]$_2$ (5.0 mol%, 10 mol% or 15 mol%) and internal standard 4-fluoroanisole (0.14 mg, 15 μmol, 1.0 equiv) was added to the reaction mixture. The J-Young NMR tube was then connected to a high vacuum line and a

balloon containing H_2 (1 atm) via a T-bore glass stopcock adaptor (Figure S2). The reaction mixture was degassed via three freeze-pump-thaw cycles. After the third freeze-pump-thaw cycle, H_2 (1 atm) was introduced to the NMR tube while keeping the bottom of the NMR tube submersed in a water bath (23 °C). After the NMR tube was warmed to 23 °C, the NMR tube was sealed, and the reaction mixture was shaken vigorously at 23 °C on a circular vibrating shaker (Heidolph Vibramax 100). After the indicated time, the NMR tube was removed from the vibrating shaker, and the ^{19}F NMR of the crude product mixture was recorded. Then the NMR tube was recycled and shaken vigorously on the vibrating shaker. The yields of fluorobenzene reflect an average of two independent trials. The yield was plotted as a function of time. The full kinetic reaction profile of the reaction was recorded (Figure S10-S12), and we observed a first-order kinetics (Figures S13-S15). Furthermore, no induction period was observed in all cases.

Note: *Due to the low solubility of the fluorobenzene-derived tetrafluorothianthrenium tetrafluoroborate (**2-TFTBF$_4$**) and the hexafluorophosphate salt (**2-TFTPF$_6$**) in THF-d$_8$, the BF_4 and PF_6 counterions were replaced with Tf_2N^- to generate a more soluble fluorobenzene-derived tetrafluorothianthrenium bis(trifluoromethanesulfonyl)amide salt (**2-TFTNTF$_2$**). Without shaking the NMR tube, there is virtually no conversion of fluorobenzene-derived tetrafluorothianthrenium salt during the NMR acquisition time.*

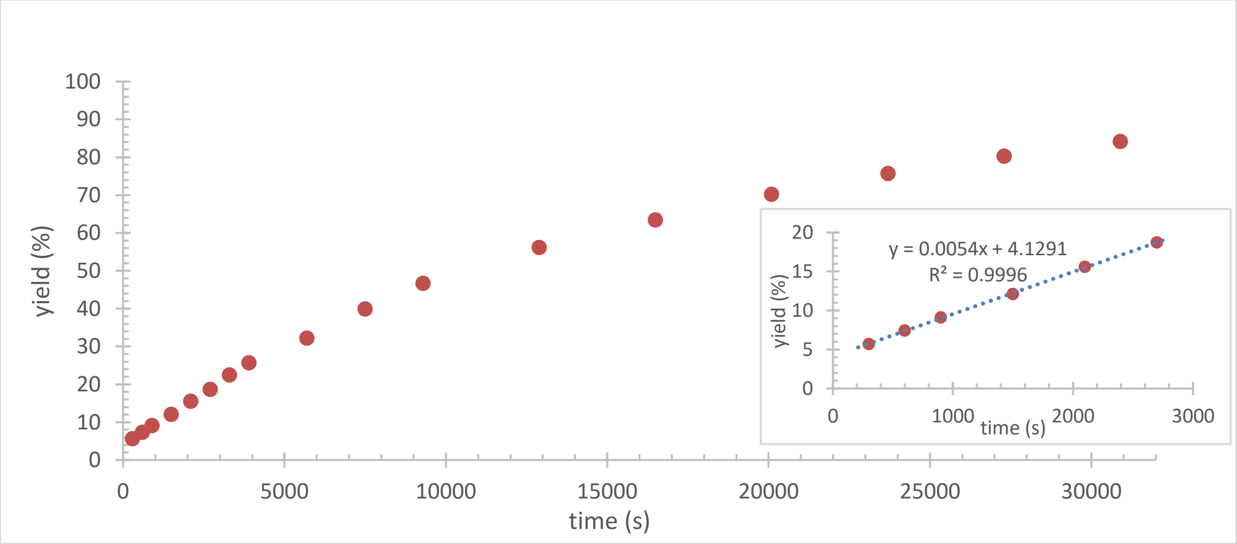

Figure S10. Full kinetic reaction profile of fluorobenzene with 5% of catalyst.

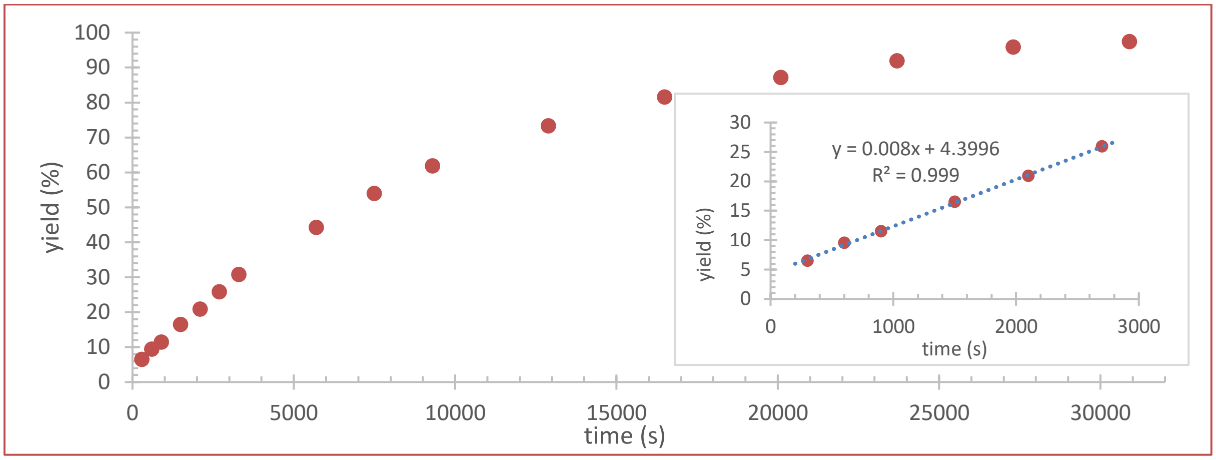

Figure S11. Full kinetic reaction profile of fluorobenzene with 10% of catalyst.

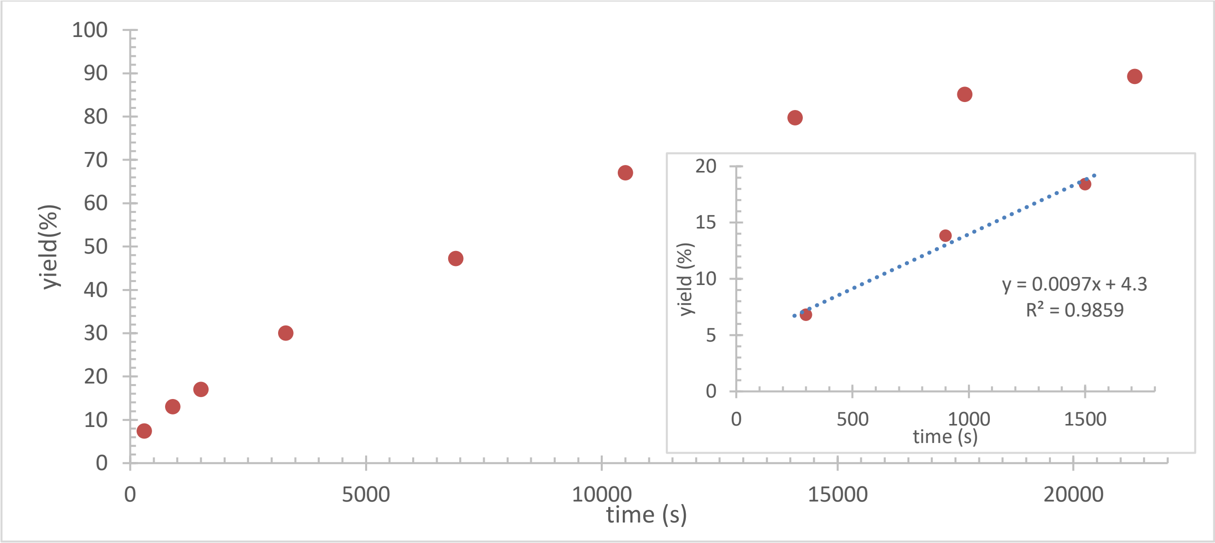

Figure S12. Full kinetic reaction profile of fluorobenzene with 15% of catalyst.

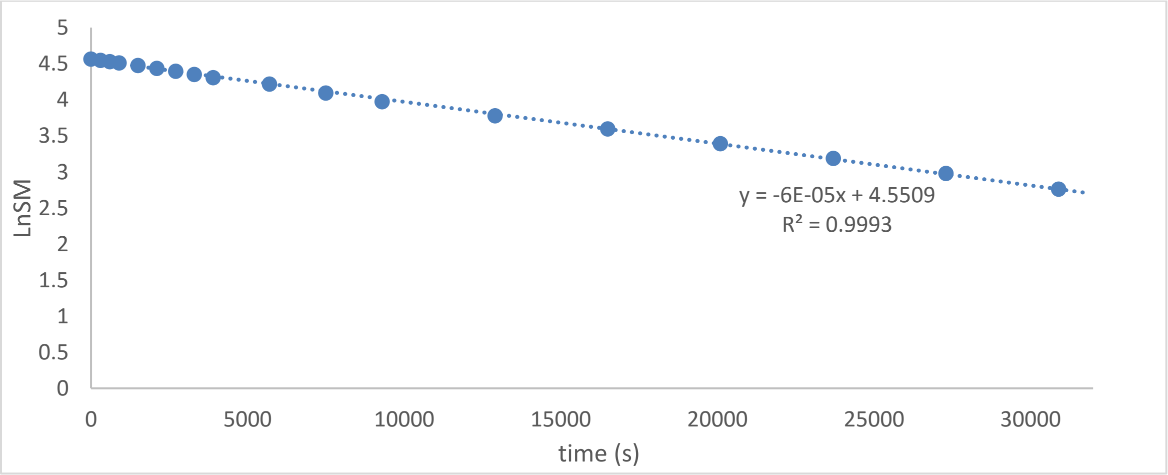

Figure S13. Plot of the natural logarithm of the concentration of fluorobenzene-derived tetrafluorothianthrenium salt versus time with 5% of catalyst.

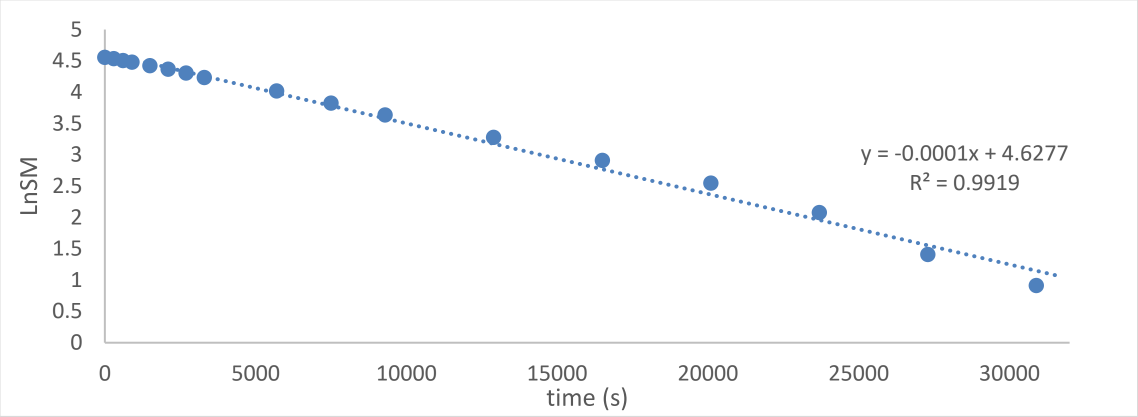

Figure S14. Plot of the natural logarithm of the concentration of fluorobenzene-derived tetrafluorothianthrenium salt versus time with 10% of catalyst.

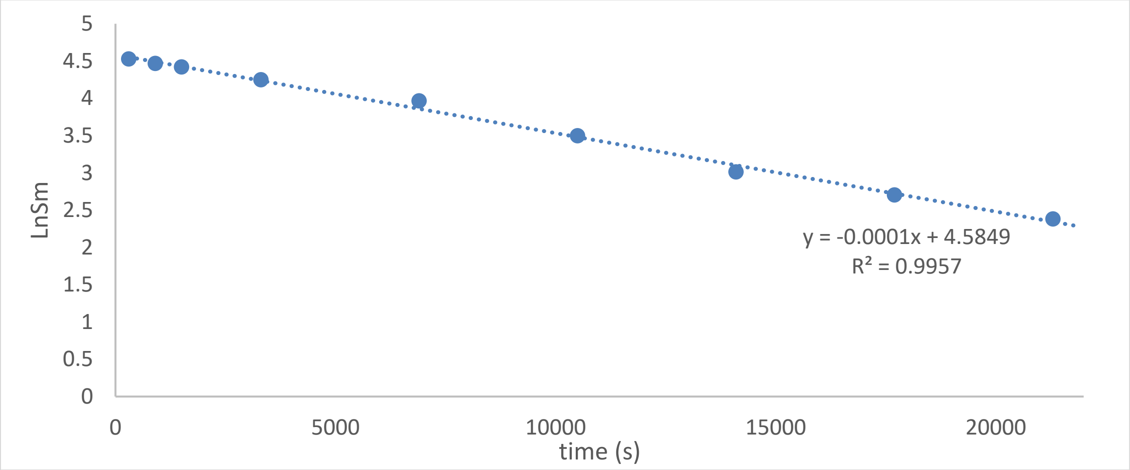

Figure S15. Plot of the natural logarithm of the concentration of fluorobenzene-derived tetrafluorothianthrenium salt versus time with 15% of catalyst.

Determination of the reaction order in catalyst

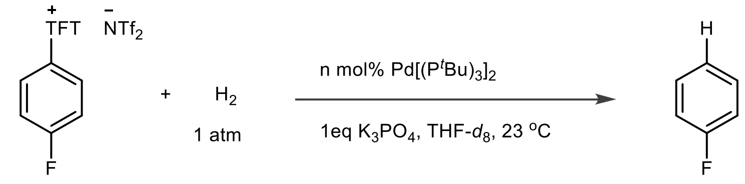

The order of catalyst was investigated by a normalized time scale to plot the concentration of starting material directly against t•[cat]^n , in which n is the order of the catalyst, in accordance with Burés' graphical rate law analysis (Figure S16-S18)[6]. The three different catalyst loadings overlay when n = 0.5, indicating that the reaction is half order in the palladium complex Pd[P*t*Bu)$_3$]$_2$.

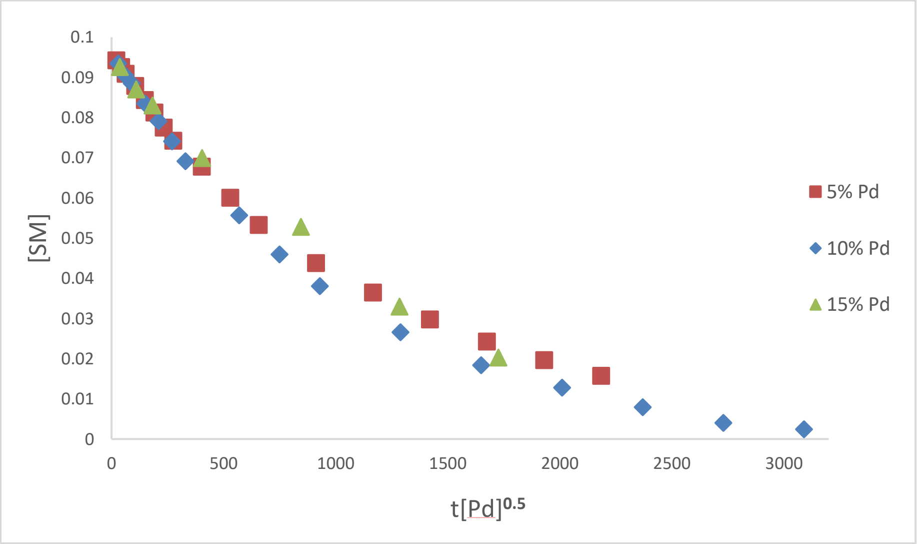

Figure S16. Burés' graphical rate law analysis for n = 0.5.

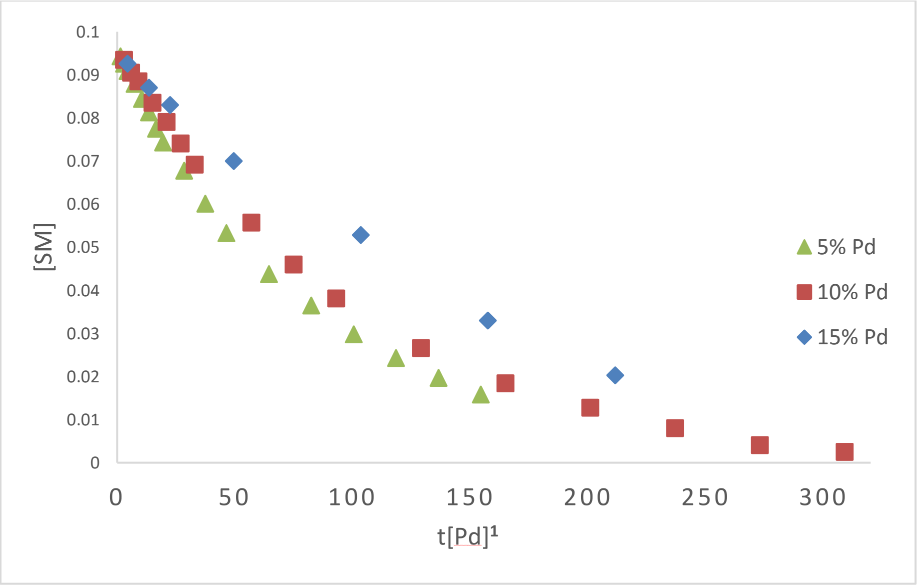

Figure S17. Burés' graphical rate law analysis for n = 1.0.

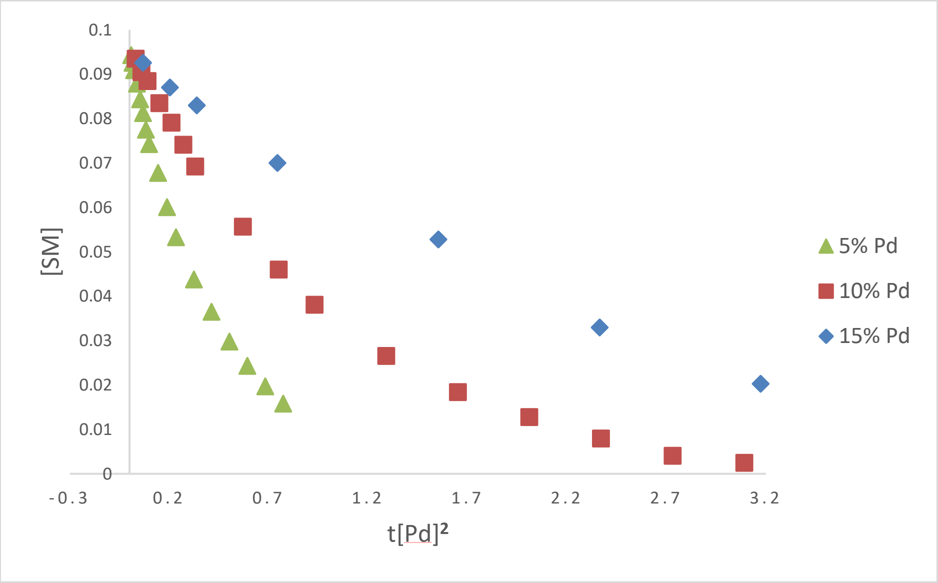

Figure S18. Burés' graphical rate law analysis for n = 2.0.

Analysis of the reaction order in catalyst

The rate equations of hydrogenolysis of aryl thianthrenium salt based on mechanism shown in Figure 4 are listed as eqs 1-5:

$$Pd^0[P(tBu)_3]_2 \underset{k_{-1}}{\overset{k_1}{\rightleftharpoons}} Pd^0[P(tBu)_3] + P(tBu)_3 \qquad \text{eq 1}$$

$$Pd^0[P(tBu)_3] + ArTT^+ \underset{k_{-2}}{\overset{k_2}{\rightleftharpoons}} [P(tBu)_3]Pd^{II}Ar^+ + TT \qquad \text{eq 2}$$

$$[P(tBu)_3]Pd^{II}Ar^+ + H_2 \underset{k_{-3}}{\overset{k_3}{\rightleftharpoons}} [P(tBu)_3]Pd^{II}Ar(H_2)^+ \qquad \text{eq 3}$$

$$[P(tBu)_3]Pd^{II}Ar(H_2)^+ + base \xrightarrow{k_4} [P(tBu)_3]Pd^{II}Ar(H) + baseH \qquad \text{eq 4}$$

$$[P(tBu)_3]Pd^{II}Ar(H) \xrightarrow{k_5} ArH + Pd^0P(tBu)_3 \qquad \text{eq 5}$$

The rate of the hydrogenolysis is shown in eq 6:

$$\text{rate} = k_4\,[[\text{P}(t\text{Bu})_3]\text{Pd}^{\text{II}}\text{Ar}(\text{H}_2)^{+}]\,[\text{base}] \qquad \text{eq 6}$$

By solving the equilibria in eq 3, the concentration of $[P(tBu)_3]PdAr(H_2)$ is derived in eq 7:

$$[[\text{P}(t\text{Bu})_3]\text{Pd}^{\text{II}}\text{Ar}(\text{H}_2)^{+}] = K_3\,[[\text{P}(t\text{Bu})_3]\text{Pd}^{\text{II}}\text{Ar}^{+}]\,[\text{H}_2] \qquad \text{eq 7}$$

Similarly, the concentrations of $[P(tBu)_3]PdAr$ and $[P(tBu)_3]Pd$ can be derived in eq 8-9:

$$[[\text{P}(t\text{Bu})_3]\text{PdAr}^{+}] = K_2\,[\text{Pd}[\text{P}(t\text{Bu})_3]]\,[\text{ArTT}^{+}]/[\text{TT}] \qquad \text{eq 8}$$

$$[\text{Pd}[\text{P}(t\text{Bu})_3]] = K_1\,[\text{Pd}[\text{P}(t\text{Bu})_3]_2]\,/\,[\text{P}(t\text{Bu})_3] \qquad \text{eq 9}$$

Considering the concentration of free phosphine equals to that of $[P(tBu)_3]Pd$ in eq 1, the concentration of $[P(tBu)_3]Pd$ at every point in time is proportional to the square root of the catalyst concentration, as shown in eq 10:

$$[\text{Pd}[\text{P}(t\text{Bu})_3]] = (K_1[\text{Pd}[\text{P}(t\text{Bu})_3]_2])^{0.5} \qquad \text{eq 10}$$

Substituting eqs 7,8 and 10 into eq 6 yields the final rate expression, which is in agreement of the half-order kinetics with respect to palladium catalyst.

$$\textbf{rate} = k\,[Pd(PtBu_3)_2]^{\frac{1}{2}}\,[ArTT^{+}][H_2];\ \text{, where } k = [K_1]^{\frac{1}{2}}\cdot K_2\cdot K_3\cdot k_4.$$

Note: The analysis is independent of the turnover-limiting step, or the following steps, meaning, even if our proposal for the following steps were wrong, we can state unambiguously that ligand dissociation from the pre-catalyst as proposed results in the reaction order of 0.5 for the monomeric palladium pre-catalyst.

Kinetic H/D isotope effect analysis

Independent reactions with H_2 and $^2H_2$

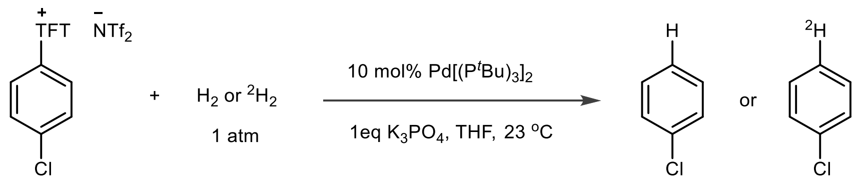

7-TFTNTF$_2$ **7** **[^{2}H]7**

Chlorobenzene-derived tetrafluorothianthrenium salt **7-TFTNTF$_2$** (10.0 mg, 15.0 µmol, 1.0 equiv.), and K_3PO_4 (3.1 mg, 15 µmol, 1.0 equiv.) were added to a J-Young NMR tube. Subsequently, a stock THF solution (0.5 mL) containing Pd[(P*t*Bu$_3$)]$_2$ (0.77 mg, 1.5 µmol, 10 mol%) and internal standard mesitylene (1.8 mg, 0.015 mmol, 1.0 equiv.) was added to the reaction mixture. The J-Young NMR tube was then connected to a high vacuum line and a balloon containing H_2 or $^2H_2$ (1 atm) via a T-bore glass stopcock adaptor (Figure S2). The reaction mixture was degassed via three freeze-pump-thaw cycles. After the third freeze-pump-thaw cycle, H_2 or $^2H_2$ (1 atm) was introduced to the NMR tube while keeping the bottom of the NMR tube submersed in a water bath (23 °C). After the NMR tube was warmed to 23 °C, the NMR tube was sealed, and the reaction mixture was shaken vigorously at 23 °C on a circular vibrating shaker (Heidolph Vibramax 100). After the indicated time, the NMR tube was removed from the vibrating shaker, and the ^1H NMR of the crude product mixture was recorded. Then the NMR tube was recycled and shaken vigorously on the vibrating shaker. The yields of chlorobenzene was determined by ^1H NMR integration relative to the internal standard and reflect an average of two independent trials. An isotope effect (k_H/k_D = 3.12) was obtained by comparing the initial rates (Figure S19).

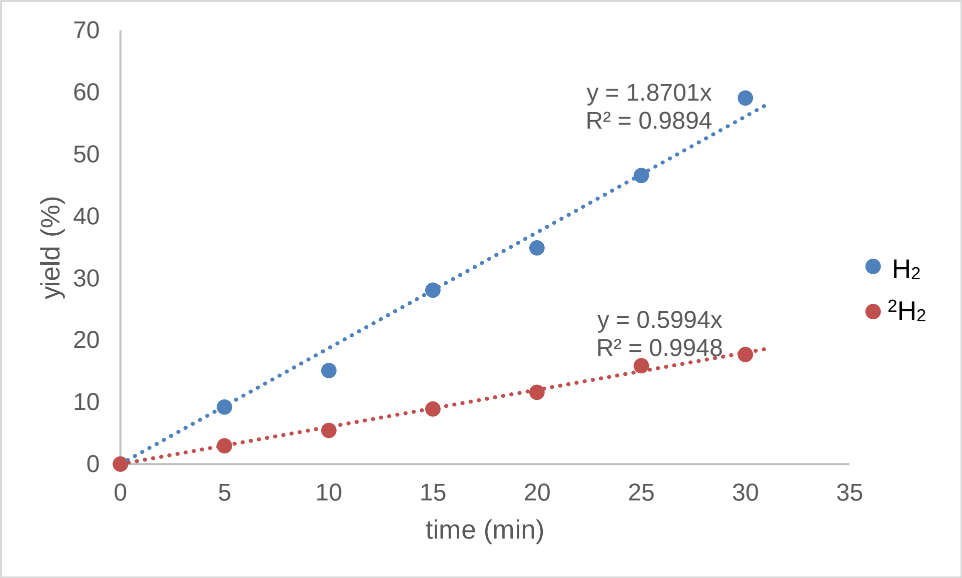

Figure S19. Initial rates for hydrogenolysis of chlorobenzene-derived tetrafluorothianthrenium salt with H_2 and $^2H_2$.

Intermolecular competition reaction with H_2 and $^2H_2$

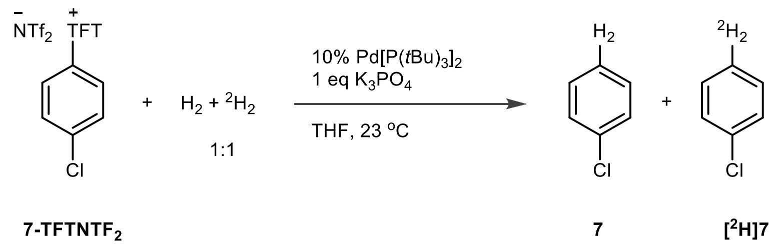

Chlorobenzene-derived tetrafluorothianthrenium salt **7-TFTNTF₂** (10.0 mg, 15.0 µmol, 1.0 equiv.), and K_3PO_4 (3.1 mg, 15 µmol, 1.0 equiv.) were added to a J-Young NMR tube. Subsequently, a stock THF solution (1.0 mL) containing Pd[(P*t*Bu$_3$)]$_2$ (0.77 mg, 1.5 µmol, 10 mol%) was added to the reaction mixture. The J-Young NMR tube was then connected to a high vacuum line and another Schlenk tube containing H_2 and $^2H_2$ (1:1, total pressure of 1 atm) via a T-bore glass stopcock adaptor (Figure S2). The reaction mixture was degassed via three freeze-pump-thaw cycles. After the third freeze-pump-thaw cycle, the mixture of H_2 and $^2H_2$ (1:1, total pressure of 1 atm) was introduced to the Schlenk tube while keeping the bottom of the Schlenk tube submersed in a water bath (23 °C). After the Schlenk tube was warmed to 23 °C, the Schlenk tube was sealed, and the reaction mixture was stirred vigorously at 23 °C. After 12 hours, the reaction vessel was opened to air, and CH_2Cl_2 (5 mL) was added to the reaction mixture. The resulting mixture was gently concentrated by rotary evaporation at 0 °C. The ^{2}H-incorporation of the product chlorobenzene was determined by mass analysis. An isotope effect (k_H/k_D = 1.06) was calculated from the ^{2}H-incorporation of the product and reflects an average of three independent trials.

Influence of H_2 pressure on the rate of hydrogenolysis

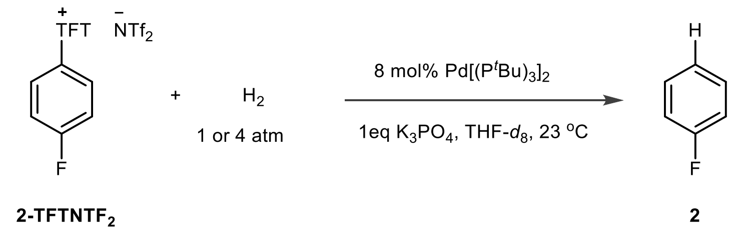

Fluorobenzene-derived tetrafluorothianthrenium salt **2-TFTNTF₂** (6.6 mg, 10 µmol, 1.0 equiv.), and K_3PO_4 (2.1 mg, 10 µmol, 1.0 equiv.) were added to a J-Young NMR tube. Subsequently, a stock THF-d_8 solution (0.5 mL) containing Pd[(P*t*Bu$_3$)]$_2$ (0.62 mg, 0.80 µmol, 8.0 mol%) and internal standard 4-fluoroanisole (0.13 mg, 0.11 µL, 10 µmol, 1.0 equiv) was added to the reaction mixture. The J-Young NMR tube was then connected to a high vacuum line and a

balloon containing H_2 (1 or 4 atm) via a T-bore glass stopcock adaptor (Figure S2). The reaction mixture was degassed via three freeze-pump-thaw cycles. After the third freeze-pump-thaw cycle, H_2 (1 or 4 atm) was introduced to the NMR tube while keeping the bottom of the NMR tube submersed in a water bath (23 °C). After the NMR tube was warmed to 23 °C, the NMR tube was sealed, and the reaction mixture was shaken vigorously at 23 °C on a circular vibrating shaker (Heidolph Vibramax 100). After the indicated time, the NMR tube was removed from the vibrating shaker, and the ^{19}F NMR of the crude product mixture was recorded. Then the NMR tube was recycled and shaken vigorously on the vibrating shaker. The yields of fluorobenzene reflect an average of two independent trials. The yield was plotted as a function of time. We observe that the hydrogenolysis of aryl thianthrenium salts proceeds faster under higher pressure of H_2. (Figures S20).

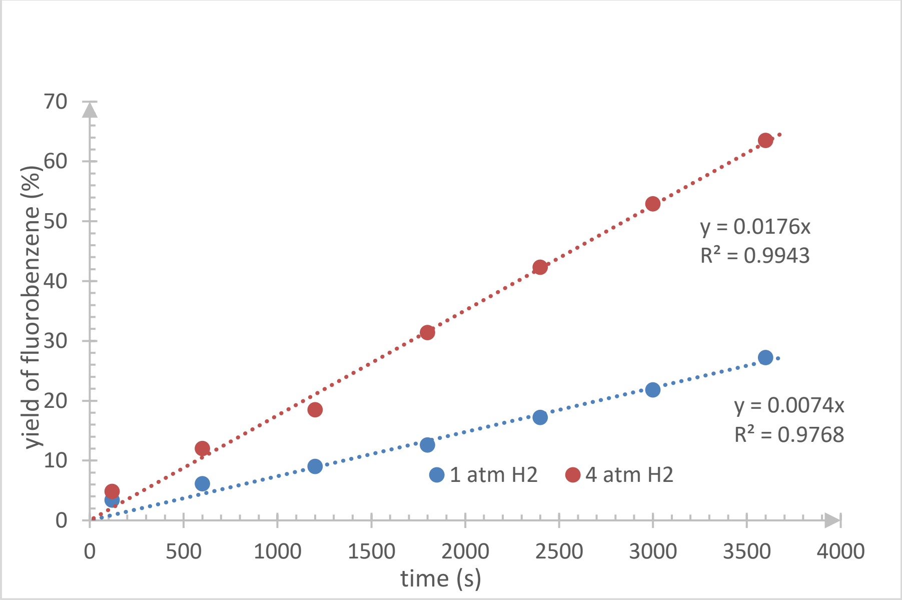

Figure S20. Pressure dependence in hydrogenolysis.

Determination of the reaction order in leaving group

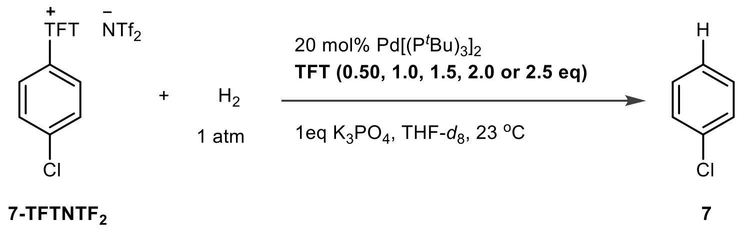

Chlorobenzene-derived tetrafluorothianthrenium salt **7-TFTNTF₂** (6.8 mg, 10 µmol, 1.0 equiv.), tetrafluorothianthrene (0.50, 1.0, 1.5, 2.0 or 2.5 equiv.) and K_3PO_4 (2.1 mg, 10 µmol, 1.0 equiv.) were added to a J-Young NMR tube. Subsequently, a stock THF-d_8 solution (0.5 mL) containing

$Pd[(PtBu_3)]_2$ (1.6 mg, 2.0 µmol, 20 mol%) and internal standard mesitylene (1.2 mg, 1.4 µL, 10 µmol, 1.0 equiv.) was added to the reaction mixture. The J-Young NMR tube was then connected to a high vacuum line and a balloon containing H_2 (1 atm) via a T-bore glass stopcock adaptor (Figure S2). The reaction mixture was degassed via three freeze-pump-thaw cycles. After the third freeze-pump-thaw cycle, H_2 (1 atm) was introduced to the NMR tube while keeping the bottom of the NMR tube submersed in a water bath (23 °C). After the NMR tube was warmed to 23 °C, the NMR tube was sealed, and the reaction mixture was shaken vigorously at 23 °C on a circular vibrating shaker (Heidolph Vibramax 100). After the indicated time, the NMR tube was removed from the vibrating shaker, and the ^1H NMR of the crude product mixture was recorded. Then the NMR tube was recycled and shaken vigorously on the vibrating shaker. The yields of chlorobenzene reflect an average of two independent trials. The initial rate was plotted as a function of equivalent of leaving group. We observe a zero order in leaving group for the hydrogenolysis reaction (Figure S21).

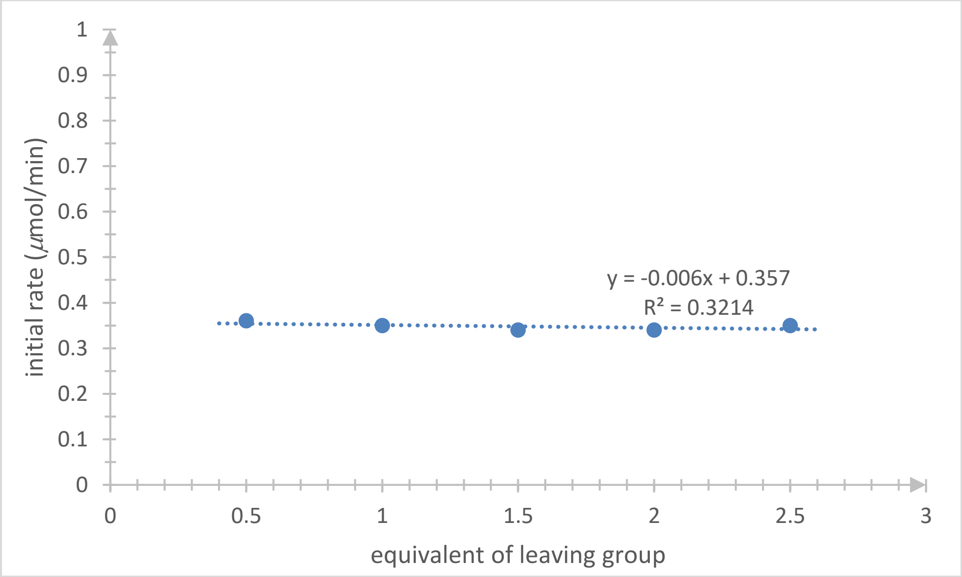

Figure S21. Plot of initial rates as a function of leaving group equivalent.

Mercury poison test

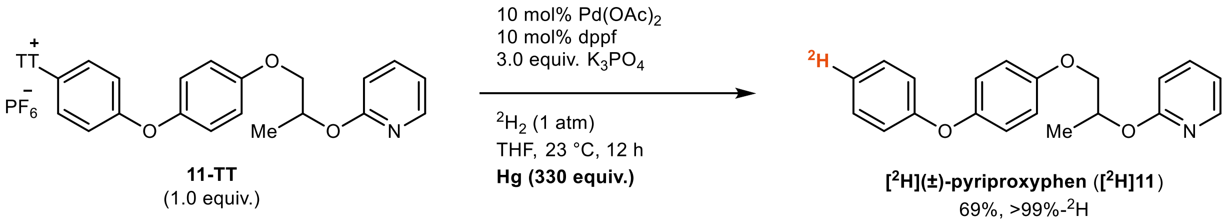

Pyriproxyfen-derived thianthrenium salt **11-TT** (0.5 mg, 0.7 µmol, 1 equiv.), K_3PO_4 (0.5 mg, 2 µmol, 3 equiv.), and mercury (48.6 mg, 0.240 mmol, 330 equiv.) were added to a J-Young NMR tube. Subsequently, a stock THF solution (0.5 mL) containing $Pd(OAc)_2$ (10 mol%) and dppf (10 mol%) was added to the reaction mixture. The J-Young NMR tube was then connected to a high vacuum line and a balloon containing $^2H_2$ (1 atm) via a T-bore glass stopcock adaptor (Figure S2). The reaction mixture was degassed via three freeze-pump-thaw cycles. After the third freeze-pump-thaw cycle, $^2H_2$ (1 atm) was introduced to the NMR tube while keeping the bottom of the NMR tube submersed in a water bath (23 °C). After the NMR tube was warmed to 23 °C, the NMR tube was sealed, and the reaction mixture was shaken vigorously at 23 °C on a circular vibrating shaker (Heidolph Vibramax 100). After 12 hours, the NMR tube was opened to air, and CH_2Cl_2 (5 mL) was added to the reaction mixture. The resulting mixture was concentrated by rotary evaporation. The resulting residue was diluted with 1 mL CD_3CN stock solution containing CH_2Br_2 (2 equiv.) as an internal standard. The yield of **11** was determined by ^1H NMR by integrating the resonance at 6.72 ppm of the product and comparison to the internal standard CH_2Br_2 with a resonance at 5.09 ppm. The deuterium incorporation of **11** was determined by mass analysis (98.8 ± 0.7%). Starting material **11-TT** remained as mass balance in this reaction.

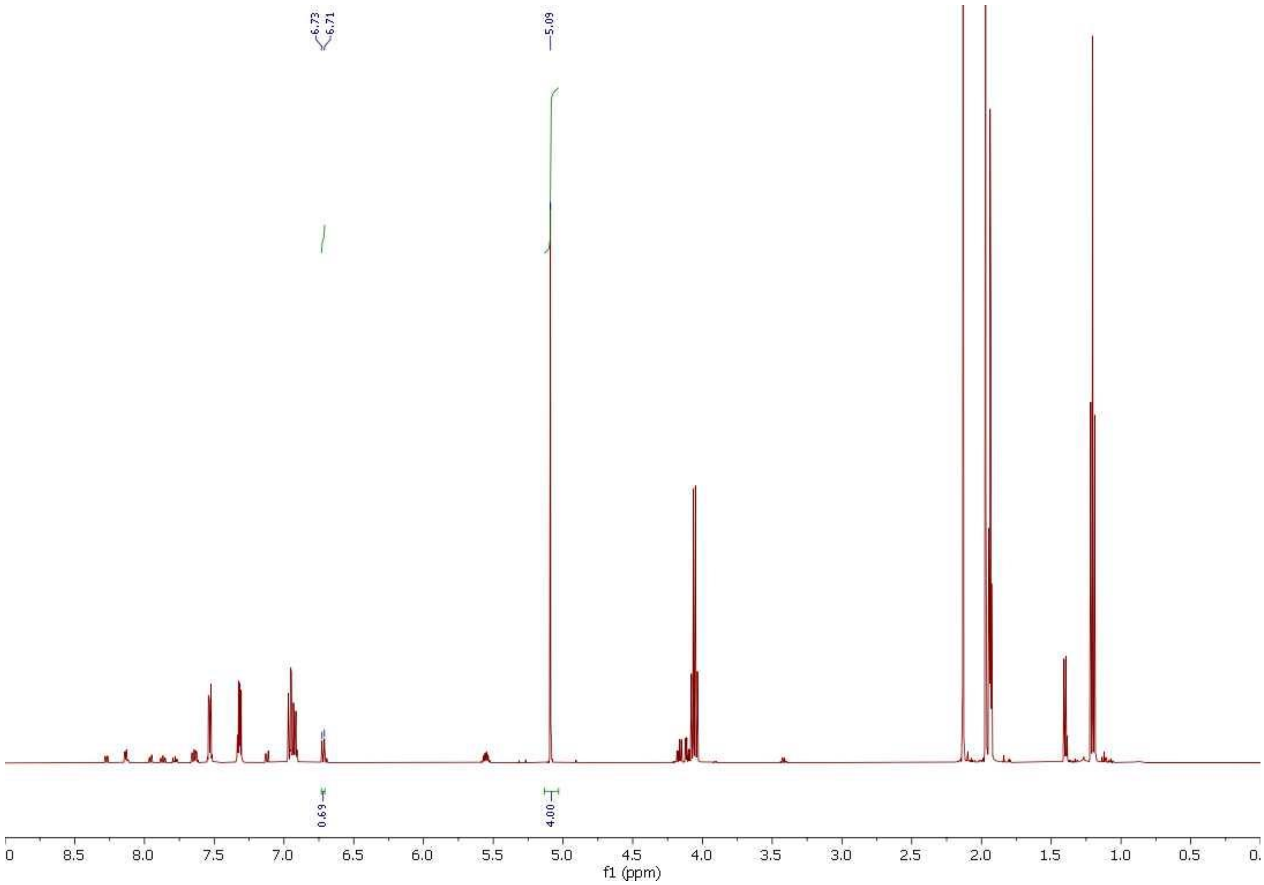

Figure S22. Crude ^{1}H NMR spectrum of the reaction mixture of **11-TT** in the presence of excess mercury with CH_2Br_2 as internal standard in CD_3CN.

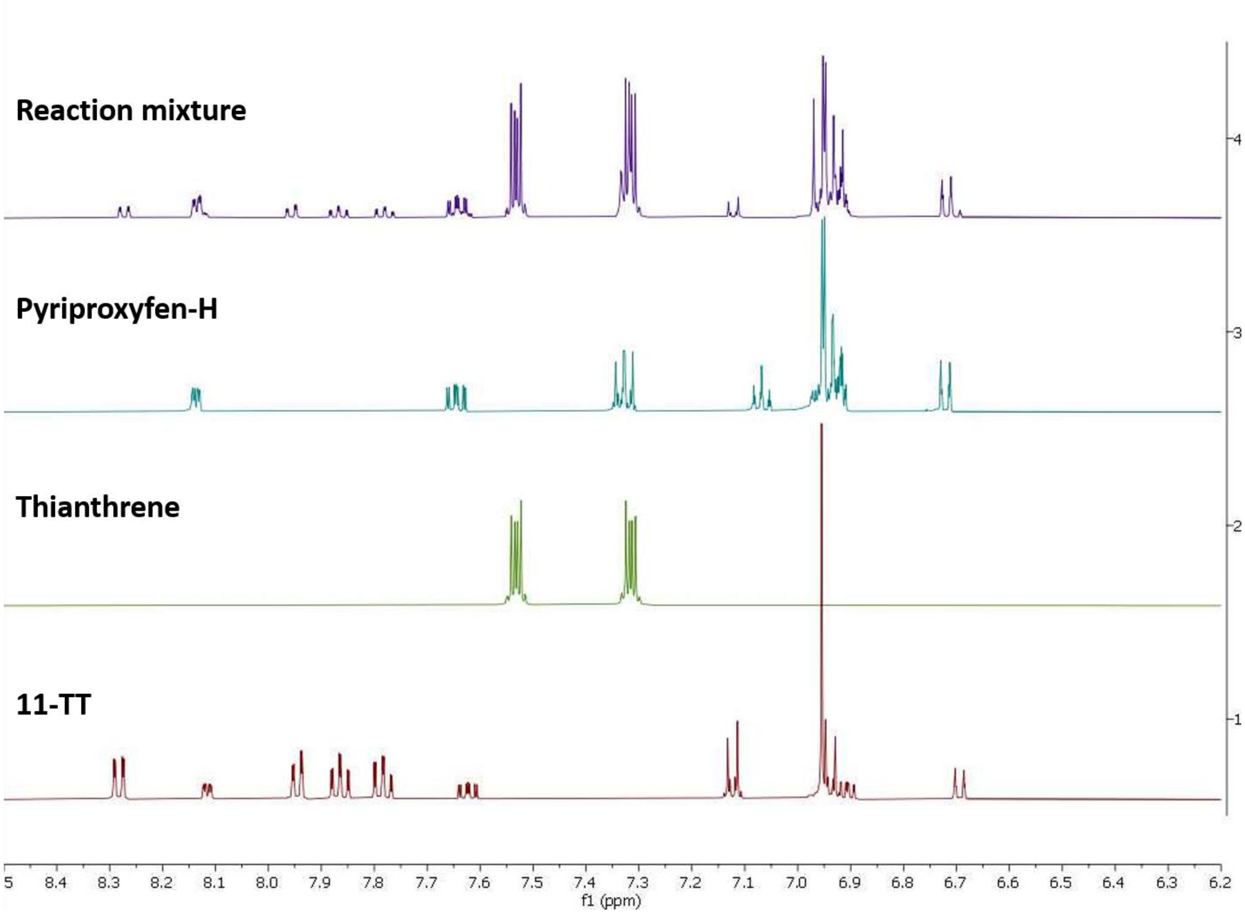

Figure S23. Comparison of ^{1}H NMR spectra of the reaction mixture of **11-TT**, pyriproxyfen-H, thianthrene and starting material **11-TT** in CD_3CN.

Reaction of aryl palladium(II) bromide with H_2

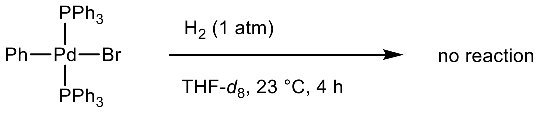

S-1

Aryl palladium(II) **S-1**[9] (5.0 mg, 6.3 µmol, 1.0 equiv) and THF-*d*$_8$ (0.5 mL) was added to a J-Young NMR tube. The J-Young NMR tube was then connected to a high vacuum line and a balloon containing H_2 (1 atm) via a T-bore glass stopcock adaptor (Figure S2). The reaction mixture was degassed via three freeze-pump-thaw cycles. After the third freeze-pump-thaw cycle, H_2 (1 atm) was introduced to the NMR tube while keeping the bottom of the NMR tube

submersed in a water bath (23 °C). After the NMR tube was warmed to 23 °C, the NMR tube was sealed, and the reaction mixture was shaken vigorously at 23 °C on a circular vibrating shaker (Heidolph Vibramax 100). After the indicated time, the NMR tube was removed from the vibrating shaker, and the ^{31}P NMR of the crude product mixture was recorded. Then the NMR tube was recycled and shaken vigorously on the vibrating shaker. Starting material **S-1** was the only species observed in ^{31}P NMR (Figure S24). *The results suggest that the halides outcompete H_2 for coordination on palladium center and the hydrogenolysis of aryl halides may operate through heterogeneous catalysis.*

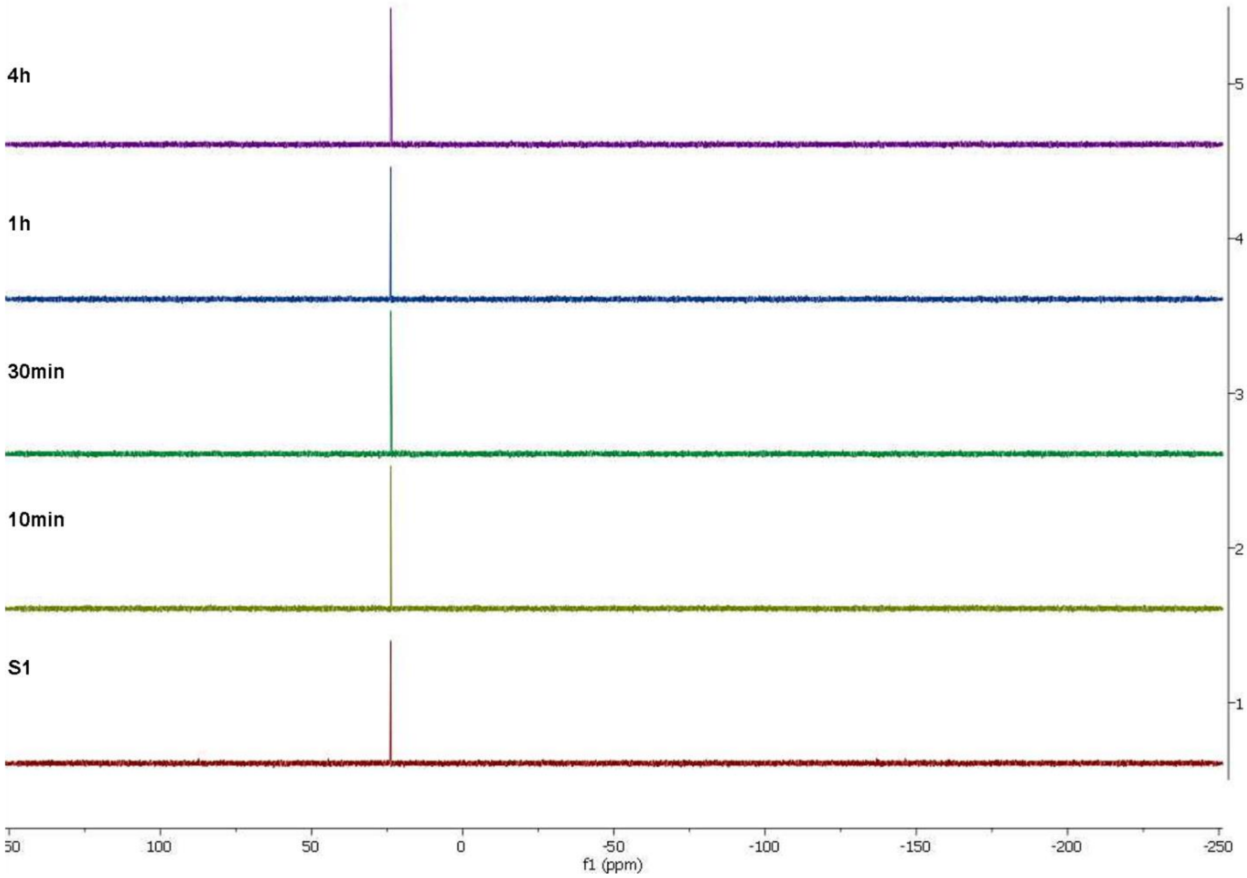

Figure S24. Comparison of ^{31}P NMR spectra of starting material **S-1** and reaction mixture (10 min, 30 min, 1 h and 4 h) in THF-d_8.

Coordinating ability comparison of thianthrene leaving group and triflate anion

The coordinating ability of thianthrene and triflate anion to arylpalladium(II) was monitored by IR spectroscopy (Scheme S2 and Figure S25).

Infrared spectroscopy study

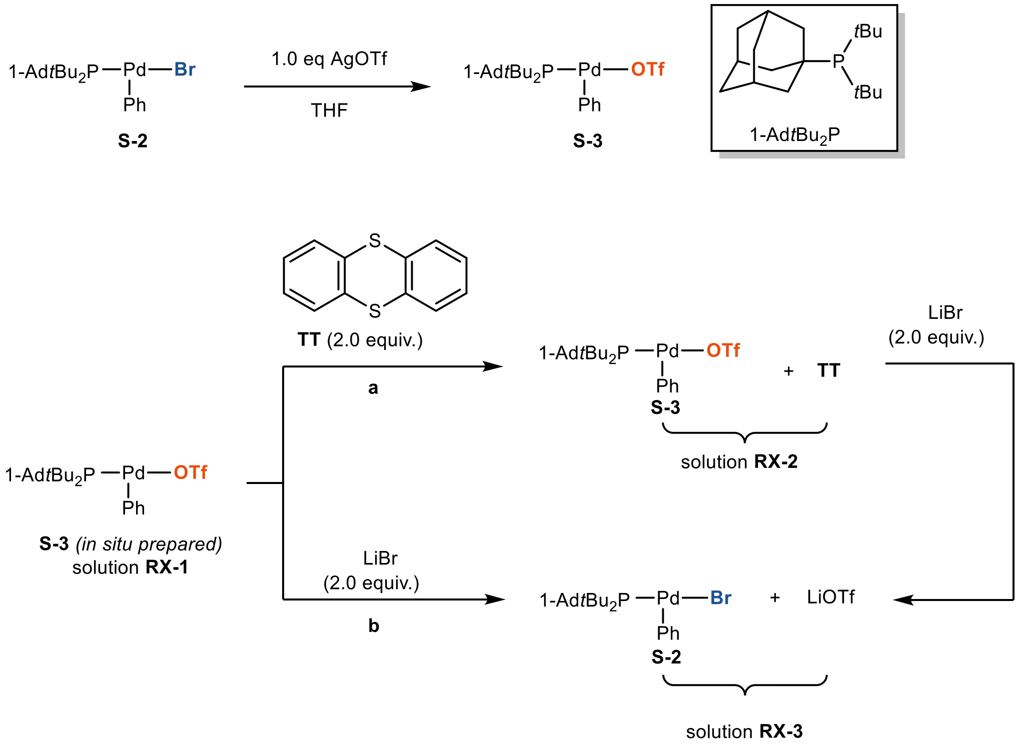

Scheme S2. Coordinating ability test of thianthrene and triflate anion by infrared spectroscopy study.

T-shaped arylpalladium(II) triflate **S-3** was prepared *in situ* from the corresponding arylpalladium(II) bromide **S-2** according to a reported method[7]. In an argon-filled glove box, arylpalladium(II) bromide **S-2** (10.9 mg, 20.0 µmol, 1.00 equiv.) and silver triflate (5.1 mg, 20 µmol, 1.0 equiv.) were added to a 4-mL vial containing a magnetic stir bar. Tetrahydrofuran (0.50 mL, c = 40 mM) was then added to the vial, affording a cloudy dark green mixture. The reaction mixture was stirred for 2 min at 28 °C. The reaction mixture was filtered through a syringe filter to afford a clear THF solution **RX-1**. Infrared spectroscopy of solution **RX-1** was recorded on a Thermo Scientific Nicolet iS5 FT-IR spectrophotometer, which is installed in the same glove box. It has been documented that free triflate typically vibrates at 1280 cm^{-1}, and coordinated triflates vibrate closer to 1380 cm^{-1} [8]. Hartwig *et al.* reported[7] that the infrared spectrum of **S-3** in THF contained a band at 1395 cm^{-1}, indicating that the triflate is bound to palladium[7]. Similarly, the triflate in **S-3** exhibits one characteristic band at 1395 cm^{-1} in the IR spectrum (Figure S25).

Solution **RX-1** was divided into two portions (**a** and **b**, 0.2 mL each) by a syringe. Thianthrene (4.3 mg, 20 µmol, 2.0 equiv.) was added to portion **a** of solution **RX-1**, affording a solution **RX-2**, and infrared spectroscopy of **RX-2** was recorded. The characteristic band of triflate in **S-3** at 1395 cm^{-1} was observed, indicating that triflate anion outcompetes thianthrene for coordination to palladium in these complexes. This characteristic band of triflate at 1395 cm^{-1} was no longer observed when LiBr (1.7 mg, 20 µmol, 2.0 equiv.) was added to the reaction mixture **RX-2**, indicating that bromide outcompetes triflate for coordination on the palladium center.

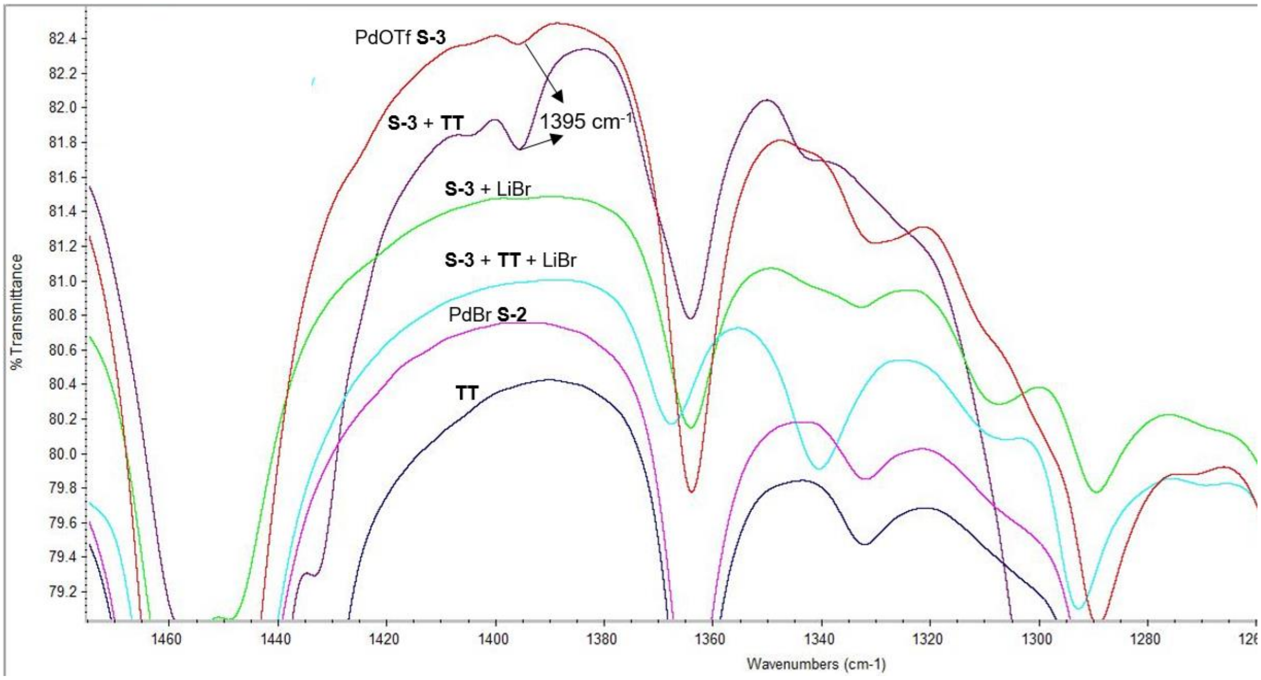

Figure S25. Comparison of infrared spectra of **S-3, S-3** + **TT**, **S-3** + LiBr, **S-3** + **TT**+ LiBr, **S-2** and **TT** in THF.

In a parallel measurement, LiBr (1.7 mg, 20 µmol, 2.0 equiv.) was directly added to portion **b** of solution **RX-1**, affording a reaction mixture **RX-3**. The infrared spectrum of **RX-3** was recorded. The characteristic band of triflate in **S-3** at 1395 cm^{-1} disappeared (Figure S25), again, indicating that bromide outcompetes triflate for coordination to the palladium center. These results suggest that the coordinating ability of thianthrene to aryl palladium(II) is weaker than triflate and bromide anions, respectively.

Reaction of T-shaped arylpalladium(II) triflate S-3 with $^2H_2$

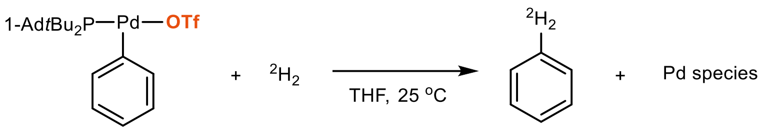

S-3
(*in situ* prepared)

Scheme S3. Reaction of T-shaped Pd(II) triflate **S-3** with $^2H_2$.

T-shaped arylpalladium(II) triflate **S-3** was prepared *in situ* from the corresponding arylpalladium(II) bromide **S-2** according to a reported method[7]. In an argon-filled glove box, arylpalladium(II) bromide **S-2** (10.9 mg, 20.0 µmol, 1.00 equiv.) and silver triflate (5.1 mg, 20 µmol, 1.0 equiv.) was added to a 4-mL vial containing a magnetic stir bar. Tetrahydrofuran (0.50 mL, c = 40 mM) was then added to the vial, affording a cloudy dark green mixture. The reaction mixture was stirred for 2 min at 28 °C. The reaction mixture was filtered through a syringe filter to a J-Young NMR tube. The J-Young NMR tube was sealed and transferred from the glove box. Then the J-Young NMR tube was connected to a high vacuum line and a balloon containing $^2H_2$ (1 atm) via a T-bore glass stopcock adaptor (Figure S2). The reaction mixture was degassed via three freeze-pump-thaw cycles. After the third freeze-pump-thaw cycle, $^2H_2$ (1 atm) was introduced to the NMR tube while keeping the bottom of the NMR tube submersed in a water bath (23 °C). After the NMR tube was warmed to 23 °C, the NMR tube was sealed, and the reaction mixture was shaken vigorously at 23 °C on a circular vibrating shaker (Heidolph Vibramax 100). After 2 hours, the reaction vessel was opened to air, and CH_2Cl_2 (5 mL) was added to the reaction mixture. The resulting mixture was concentrated by rotary evaporation and analyzed by mass analysis. 0%-^{2}H incorporation was detected in product benzene by mass analysis.

Thianthrenation of arenes

2-Nitro-biphenyl-derived tetrafluorothianthrenium salt (19-TFT)

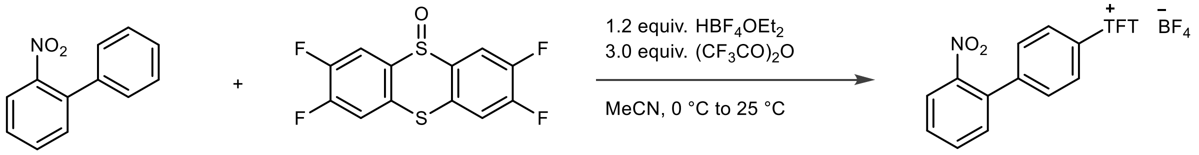

19 **19-TFT**

Under an ambient atmosphere, a 20 mL glass vial was charged with 2-nitro-biphenyl **19** (398 mg, 2.00 mmol, 1.00 equiv.), (tetrafluoro)thianthrenium-*S*-oxide (609 mg, 2.00 mmol, 1.00 equiv.), and dry MeCN (10 mL, c = 0.20 M). After cooling to 0 °C, $HBF_4 \cdot OEt_2$ (0.39 g, 0.32 mL, 2.4 mmol, 1.2 equiv.) was added to the vial while stirring the reaction mixture. Subsequently, trifluoroacetic anhydride (1.3 g, 1.5 mL, 6.0 mmol, 3.0 equiv.) was added in one portion at 0 °C, resulting in a color change to deep purple. The vial was sealed with a screw-cap. The mixture was stirred at 0 °C for 1 h and then at 25 °C until the intensity of the purple color decreased visually. The solution was concentrated and the residue was diluted with 5 mL dichloromethane and poured onto a mixture of 30 mL dichloromethane, 20 mL saturated aqueous Na_2CO_3 solution, and 10 mL water. After stirring for 5 min at 25 °C, the mixture was poured into a separatory funnel, and the layers were separated. The dichloromethane layer was washed with aqueous $NaBF_4$ solution (2 × ca. 20 mL, 5 % w/w) and with water (2 × ca. 20 mL). The dichloromethane layer was dried over Na_2SO_4, filtered, and the solvent was removed under reduced pressure. The residue was purified by chromatography on silica gel eluting with CH_2Cl_2/MeOH 30:1 (v/v) to afford 550 mg (48% yield) of the title compound **19-TFT** as a colorless solid.

R_f = 0.35 (CH_2Cl_2/MeOH 9:1 (v:v))

NMR Spectroscopy:

^{1}H NMR (500 MHz, CD_3CN, 298 K, δ): 8.45 (ddd, *J* = 8.9, 7.2, 1.3 Hz, 2H), 8.02 – 7.97 (m, 3H), 7.75 (td, *J* = 7.6, 1.3 Hz, 1H), 7.64 (ddd, *J* = 8.2, 7.5, 1.5 Hz, 1H), 7.49 – 7.46 (m, 2H), 7.42 (dd, *J* = 7.7, 1.4 Hz, 1H), 7.29 – 7.26 (m, 2H).

^{13}C NMR (126 MHz, CD_3CN, 298 K, δ): 155.9 (d, *J* = 13.2 Hz), 153.8 (d, *J* = 13.2 Hz), 152.6 (d, *J* = 13.8 Hz), 150.6 (d, *J* = 13.8 Hz), 144.4, 135.4 (dd, *J* = 8.6, 4.0 Hz), 134.5 (d, *J* = 26.6 Hz), 132.9, 130.9, 130.9, 129.4, 125.8 (d, *J* = 2.5 Hz), 125.6, 123.2, 121.3 (d, *J* = 21.9 Hz), 115.1 (d, *J* = 4.0 Hz).

^{19}F NMR (471 MHz, CD_3CN, 298 K, δ): –124.9 (m), –133.4 (m), –151.7 (s).

HRMS-ESI POS (m/z) calc'd for $C_{24}H_{12}N_1O_2S_2F_4$ $[M-BF_4]^+$, 486.0237; found, 486.0240; deviation: 0.6 ppm.

2,2,2-Trichloroethyl (4-phenylbutyl)carbamate (24)

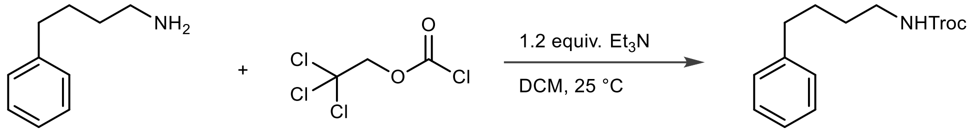

S-4 24

Under an ambient atmosphere, a 20 mL glass vial was charged with 4-phenylbutylamine **S-4** (318 µL, 298 mg, 2.00 mmol, 1.00 equiv.), 2,2,2-trichloroethoxycarbonyl chloride (330 µL, 508 mg, 2.40 mmol, 1.20 equiv.), and dry DCM (8.0 mL, c = 0.25 M). After cooling to 0 °C, triethylamine (330 µL, 243 mg, 2.40 mmol, 1.20 equiv.) was added to the vial. The resulting mixture was stirred at 25 °C for 1 h. Then the solvent was removed under reduced pressure. The residue was purified by chromatography on silica gel eluting with pentane/EA 10:1 (v/v) to afford 584 mg (90% yield) of the title compound as a colorless oil.

R_f = 0.44 (CH_2Cl_2/EA 8:1 (v:v))

NMR Spectroscopy:

^{1}H NMR (500 MHz, $CDCl_3$, 298 K, δ): 7.31 – 7.25 (m, 2H), 7.23 – 7.12 (m, 3H), 4.94 (s, 1H), 4.72 (s, 2H), 3.26 (td, *J* = 7.0, 6.0 Hz, 2H), 2.64 (t, *J* = 7.5 Hz, 2H), 1.73 – 1.63 (m, 2H), 1.61 – 1.52 (m, 2H).

^{13}C NMR (126 MHz, $CDCl_3$, 298 K, δ): 154.7, 142.1, 128.5, 128.5, 126.0, 95.8, 74.6, 41.3, 35.6, 29.5, 28.6.

HRMS-ESI POS (m/z) calc'd for $C_{13}H_{16}N_1O_2Cl_3$ $[M]^+$, 323.0238; found, 323.0241; deviation: 0.9 ppm.

2,2,2-Trichloroethyl (4-phenylbutyl)carbamate-derived thianthrenium salt (24-TT)

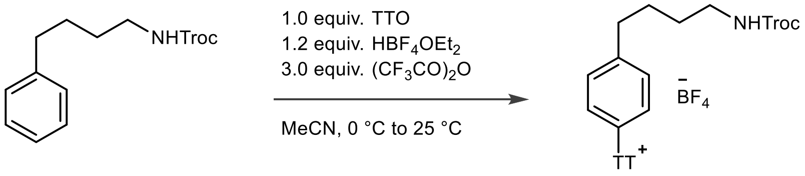

24 24-TT

Under an ambient atmosphere, a 20 mL glass vial was charged with **24** (64.9 mg, 0.200 mmol, 1.00 equiv.), thianthrenium-*S*-oxide (46.4 mg, 0.200 mmol, 1.00 equiv.), and dry MeCN (2.0 mL,

c = 0.10 M). After cooling to 0 °C, $HBF_4 \cdot OEt_2$ (38.9 mg, 32.7 µL, 0.240 mmol, 1.20 equiv.) was added to the vial while stirring the reaction mixture. Subsequently, trifluoroacetic anhydride (83.4 µL, 126 mg, 0.600 mmol, 3.00 equiv.) was added in one portion at 0 °C, resulting in a color change to deep purple. The vial was sealed with a screw-cap. The mixture was stirred at 0 °C for 10 min and then at 25 °C for 1 h. The solution was concentrated, and the residue was diluted with 5 mL dichloromethane and poured onto a mixture of 30 mL dichloromethane, 20 mL saturated aqueous $NaHCO_3$ solution, and 10 mL water. After stirring for 5 min at 25 °C, the mixture was poured into a separatory funnel, and the layers were separated. The dichloromethane layer was washed with aqueous $NaBF_4$ solution (4 × ca. 20 mL, 5 % w/w). The dichloromethane layer was dried over Na_2SO_4, filtered, and the solvent was removed under reduced pressure. The residue was purified by chromatography on silica gel eluting with CH_2Cl_2/MeOH 20:1 (v/v) to afford 100 mg (80% yield) of the title compound **24-TT** as a colorless solid.

R_f = 0.45 (pentane/MeOH 9:1 (v:v))

NMR Spectroscopy:

^{1}H NMR (500 MHz, $CDCl_3$, 298 K, δ) δ 8.46 (dd, *J* = 8.0, 1.3 Hz, 2H), 8.04 – 7.78 (m, 4H), 7.73 (ddd, *J* = 7.9, 6.8, 2.0 Hz, 2H), 7.21 (d, *J* = 8.7 Hz, 2H), 7.04 (d, *J* = 8.6 Hz, 2H), 5.27 (t, *J* = 6.1 Hz, 1H), 4.64 (s, 2H), 3.17 (q, *J* = 6.5 Hz, 2H), 2.58 (t, *J* = 7.5 Hz, 2H), 1.72 – 1.36 (m, 4H).

^{13}C NMR (126 MHz, $CDCl_3$, 298 K, δ) δ 154.8, 148.4, 136.6, 135.6, 134.9, 130.9, 130.4, 130.3, 128.3, 121.1, 119.3, 95.8, 74.6, 40.9, 35.1, 29.4, 27.9.

^{19}F NMR (471 MHz, $CDCl_3$, 298 K, δ): –151.1 (s).

HRMS-ESI POS (m/z) calc'd for $C_{25}H_{23}N_1O_2S_2Cl_3$ $[M\text{-}BF_4]^+$, 538.0237; found, 538.0230; deviation: –1.2 ppm.

LHVS-derived thianthrenium salt (26-TT)

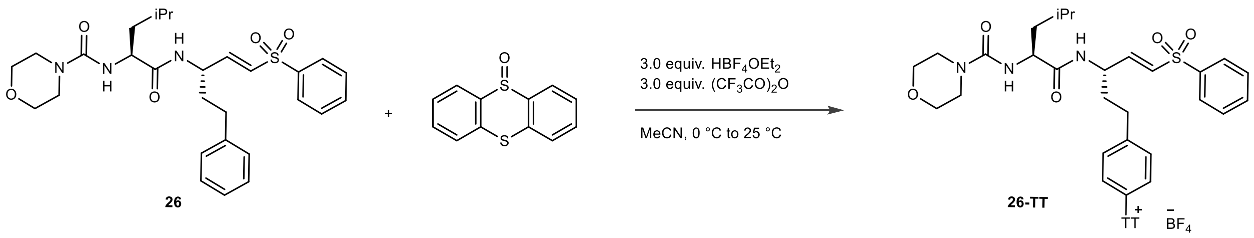

Under an ambient atmosphere, a 4 mL glass vial was charged with LHVS (**26**) (50 mg, 0.095 mmol, 1.0 equiv.), and dry MeCN (2.5 mL, c = 38 mM). After cooling to 0 °C, $HBF_4 \cdot OEt_2$ (47 mg, 39 µL, 0.29 mmol, 3.0 equiv.) was added to the vial while stirring the reaction mixture. Subsequently, thianthrenium-*S*-oxide (22 mg, 0.095 mmol, 1.0 equiv.) and trifluoroacetic anhydride (40 µL, 0.29 mmol, 3.0 equiv.) was added in one portion at 0 °C, resulting in a color change to deep purple. The vial was sealed with a screw-cap. The mixture was stirred at 0 °C for 1 h and then at 25 °C until the intensity of the purple color decreased. The solution was concentrated and the residue was diluted with 5 mL dichloromethane and poured onto a mixture of 30 mL dichloromethane, 20 mL saturated aqueous Na_2CO_3 solution, and 10 mL water. After stirring for 5 min at 25 °C, the mixture was poured into a separatory funnel, and the layers were separated. The dichloromethane layer was washed with aqueous $NaBF_4$ solution (2 × ca. 20 mL, 5 % w/w) and with water (2 × ca. 20 mL). The dichloromethane layer was dried over Na_2SO_4, filtered, and the solvent was removed under reduced pressure. The residue was purified by chromatography on silica gel eluting with CH_2Cl_2/MeOH 10:1 (v/v) to afford 39.6 mg (50% yield) of the title compound **26-TT** as a colorless solid. Further purification by preparative HPLC (YMC-Pack Pro C18 (150 × 30.0 mm, 5 µm), MeOH/water = 50:50, flow rate = 42.0 mL/min, 35 °C) provided **26-TT** as a colorless solid (32.5 mg, 41% yield).

R_f = 0.28 (CH_2Cl_2/MeOH 10:1 (v:v))

NMR Spectroscopy:

^{1}H NMR (500 MHz, CD_2Cl_2, 298 K, δ): 8.33 (ddd, *J* = 11.2, 7.9, 1.4 Hz, 1H), 7.91 (dd, *J* = 7.8, 1.4 Hz, 1H), 7.85 (td, *J* = 7.7, 1.4 Hz, 1H), 7.83 – 7.75 (m, 2H), 7.64 – 7.56 (m, 0H), 7.54 – 7.46 (m, 1H), 7.38 – 7.30 (m, 1H), 7.07 – 7.01 (m, 1H), 6.95 (d, *J* = 8.6 Hz, 1H), 6.76 (dd, *J* = 15.0, 3.8 Hz, 1H), 6.58 (dd, *J* = 15.0, 1.9 Hz, 0H), 5.52 (d, *J* = 6.9 Hz, 1H), 4.53 (ddd, *J* = 8.0, 4.1, 2.2 Hz, 0H), 4.07 (ddd, *J* = 9.8, 6.8, 5.0 Hz, 1H), 3.60 – 3.46 (m, 2H), 3.26 (dddd, *J* = 39.4, 13.0, 6.3, 3.4 Hz, 2H), 2.75 (dt, *J* = 14.5, 7.5 Hz, 1H), 2.58 (dt, *J* = 13.8, 8.1 Hz, 1H), 1.87 – 1.79 (m, 2H), 1.70 – 1.57 (m, 1H), 1.56 – 1.47 (m, 0H), 0.86 (dd, *J* = 26.6, 6.4 Hz, 3H).

^{13}C NMR (126 MHz, CD_2Cl_2, 298 K, δ): 174.4, 157.9, 148.7, 146.6, 140.8, 137.2, 135.5, 134.8, 134.8, 133.7, 131.8, 131.2, 131.2, 130.8, 130.6, 129.7, 128.4, 127.8, 120.5, 119.0, 118.9, 66.9, 54.9, 49.0, 44.4, 40.5, 35.1, 32.1, 25.3, 23.2, 21.8.

^{19}F NMR (471 MHz, CD_2Cl_2, 298 K, δ): –149.8 (s).

HRMS-ESI POS (m/z) calc'd for $C_{40}H_{44}N_3O_5S_3$ $[M-BF_4]^+$, 742.2436; found, 742.2438; deviation: 0.2 ppm.

(2-Bromoethyl)benzene-derived thianthrenium salt (27-TT1)

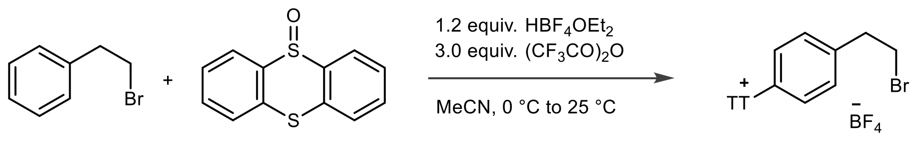

S-5 **27-TT1**

Under an ambient atmosphere, a 20 mL glass vial was charged with (2-bromoethyl)benzene **S-5** (273 µL, 370 mg, 2.00 mmol, 1.00 equiv.), thianthrenium-*S*-oxide (465 mg, 2.00 mmol, 1.00 equiv.), and dry MeCN (10 mL, c = 0.20 M). After cooling to 0 °C, $HBF_4{\cdot}OEt_2$ (0.39 g, 0.32 mL, 2.4 mmol, 1.2 equiv.) was added to the vial while stirring the reaction mixture. Subsequently, trifluoroacetic anhydride (1.3 g, 0.84 mL, 6.0 mmol, 3.0 equiv.) was added in one portion at 0 °C, resulting in a color change to deep purple. The vial was sealed with a screw-cap. The mixture was stirred at 0 °C for 1 h and then at 25 °C for 3 h. The solution was concentrated, and the residue was diluted with 5 mL dichloromethane and poured onto a mixture of 30 mL dichloromethane, 20 mL saturated aqueous $NaHCO_3$ solution, and 10 mL water. After stirring for 5 min at 25 °C, the mixture was poured into a separatory funnel, and the layers were separated. The dichloromethane layer was washed with aqueous $NaBF_4$ solution (4 × ca. 20 mL, 5 % w/w). The dichloromethane layer was dried over Na_2SO_4, filtered, and the solvent was removed under reduced pressure. The residue was purified by chromatography on silica gel eluting with CH_2Cl_2/MeOH 25:1 (v/v) to afford 720 mg (76% yield) of **27-TT1** as a colorless solid.

$\mathbf{R_f}$ = 0.50 (CH_2Cl_2/MeOH 9:1 (v:v))

NMR Spectroscopy:

^{1}H NMR (500 MHz, $CDCl_3$, 298 K, δ): 8.47 (dd, *J* = 7.5, 1.2 Hz, 2H), 7.89 – 7.80 (m, 4H), 7.78 – 7.69 (m, 2H), 7.40 – 7.21 (m, 2H), 7.09 (d, *J* = 8.6 Hz, 2H), 3.48 (t, *J* = 6.9 Hz, 2H), 3.11 (t, *J* = 7.0 Hz, 2H).

^{13}C NMR (126 MHz, $CDCl_3$, 298 K, δ): 144.9, 136.6, 135.2, 135.2, 131.2, 130.5, 130.4, 128.2, 122.1, 118.5, 38.3, 32.0.

^{19}F NMR (471 MHz, $CDCl_3$, 298 K, δ): –150.9 (s).

HRMS-ESI POS (m/z) calc'd for $C_{20}H_{16}S_2Br_1$ [M-BF_4]$^+$, 398.9871; found, 398.9871; deviation: 0.0 ppm.

Styrene-derived thianthrenium salt (27-TT)

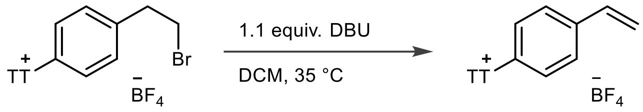

27-TT1 **27-TT**

Under an ambient atmosphere, a 20 mL glass vial was charged with **27-TT1** (97.4 mg, 0.200 mmol, 1.00 equiv.), DBU (32.9 µL, 33.5 mg, 0.220 mmol, 1.10 equiv.) and dry dichloromethane (2 mL, c = 0.1 M). The mixture was placed on a heating block preheated at 35 °C for 40 min. Then the solvent was removed under reduced pressure. The residue was purified by chromatography on silica gel eluting with CH_2Cl_2/MeOH 25:1 (v/v) to afford 71.2 mg (88% yield) of the title compound **27-TT** as a colorless solid.

R_f = 0.48 (CH_2Cl_2/MeOH 9:1 (v:v))

NMR Spectroscopy:

^{1}H NMR (500 MHz, MeOD, 298 K, δ): 8.47 (dd, J = 7.9, 1.4 Hz, 2H), 8.02 (dd, J = 7.9, 1.4 Hz, 2H), 7.93 (td, J = 7.7, 1.4 Hz, 2H), 7.86 (td, J = 7.7, 1.4 Hz, 2H), 7.57 (d, J = 8.8 Hz, 2H), 7.13 (d, J = 8.7 Hz, 2H), 6.73 (dd, J = 17.6, 11.0 Hz, 1H), 5.92 (d, J = 17.6 Hz, 1H), 5.43 (d, J = 11.0 Hz, 1H).

^{13}C NMR (126 MHz, MeOD, 298 K, δ): 143.8, 138.0, 136.3, 136.1, 135.9, 131.7, 131.1, 129.4, 129.2, 124.0, 120.6, 119.0.

^{19}F NMR (471 MHz, MeOD, 298 K, δ): –150.9 (s).

HRMS-ESI POS (m/z) calc'd for $C_{20}H_{15}S_2$ [M-BF_4]$^+$, 319.0612; found, 319.0610; deviation: –0.6 ppm.

Etofenprox-derived tetrafluorothianthrenium salt (29-TFT$_2$)

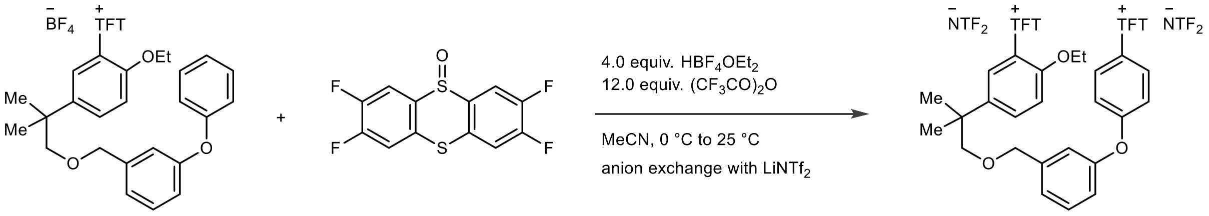

29-TFT$_1$ **29-TFT$_2$**

Under an ambient atmosphere, a 20 mL glass vial was charged with etofenprox-derived tetrafluorothianthrenium **29-TFT$_1$** (500 mg, 0.666 mmol, 1.00 equiv.), and dry MeCN (6.6 mL, c = 0.10 M). After cooling to 0 °C, $HBF_4·OEt_2$ (0.44 g, 0.36 mL, 2.7 mmol, 4.0 equiv.) was added to the vial while stirring the reaction mixture. Subsequently, (tetrafluoro)thianthrenium-*S*-oxide (208 mg, 0.666 mmol, 1.00 equiv.) and trifluoroacetic anhydride (1.7 g, 1.1 mL, 8.0 mmol, 12 equiv.) was added at 0 °C, resulting in a color change to deep purple. The vial was sealed with a screw-cap. The mixture was stirred at 0 °C for 1 h and then at 25 °C until the intensity of the purple color decreased. The solution was concentrated and the residue was diluted with 5 mL dichloromethane and poured onto a mixture of 30 mL dichloromethane, 20 mL saturated aqueous Na_2CO_3 solution, and 10 mL water. After stirring for 5 min at 25 °C, the mixture was poured into a separatory funnel, and the layers were separated. The dichloromethane layer was washed with aqueous $NaBF_4$ solution (2 × ca. 20 mL, 5 % w/w) and with water (2 × ca. 20 mL). The dichloromethane layer was dried over Na_2SO_4, filtered, and the solvent was removed under reduced pressure. In order to obtain analytically pure samples of thianthrenium salts, the residue was purified by chromatography on silica gel eluting with CH_2Cl_2/MeOH 20:1 (v/v) to afford 480 mg (64% yield) of the etofenprox-derived tetrafluorothianthrenium salt **23-TFT$_2$** with BF_4 as counterion. The etofenprox-derived tetrafluorothianthrenium salt **23-TFT$_2$** with BF_4 as counterion was then diluted with 5 mL dichloromethane, and the dichloromethane layer was washed with aqueous $LiNTF_2$ solution (2 × ca. 10 mL, 5 % w/w) and with water (2 × ca. 20 mL). The dichloromethane layer was dried over Na_2SO_4, filtered, and the solvent was removed under reduced pressure to afford 631 mg (63% yield) of the title compound **23-TFT$_2$** with NTf_2 as counterion as a colorless solid

R$_f$ = 0.33 (CH_2Cl_2/MeOH 9:1 (v:v))

NMR Spectroscopy:

^{1}H NMR (500 MHz, CD_2Cl_2, 298 K, δ): 8.29 (dd, *J* = 8.4, 6.9 Hz, 2H), 8.16 (dd, *J* = 8.5, 7.0 Hz, 2H), 7.80 (dd, *J* = 9.1, 6.7 Hz, 2H), 7.69 – 7.57 (m, 3H), 7.38 (t, *J* = 7.9 Hz, 1H), 7.33 – 7.24 (m, 2H), 7.14 – 7.05 (m, 4H), 7.01 – 6.96 (m, 1H), 6.81 (dd, *J* = 2.5, 1.6 Hz, 1H), 6.68 (d, *J* = 2.2 Hz, 1H), 4.34 (s, 2H), 4.21 (d, *J* = 7.0 Hz, 2H), 3.29 (s, 2H), 1.52 (t, *J* = 7.0 Hz, 3H), 1.16 (s, 6H).

^{13}C NMR (126 MHz, CD_2Cl_2, 298 K, δ): 163.4, 155.9, 155.4 (d, *J* = 13.2 Hz), 155.1 (d, *J* = 13.2 Hz), 154.7, 153.3 (d, *J* = 13.0 Hz), 153.0 (d, *J* = 13.0 Hz), 152.4 (d, *J* = 13.4 Hz), 152.1 (d, *J* = 13.4 Hz), 150.3 (d, *J* = 13.5 Hz), 150.0 (d, *J* = 13.3 Hz), 142.3, 141.9, 135.2, 135.1 (dd, *J* = 7.9, 4.1 Hz), 134.2 (dd, *J* = 8.0, 4.2 Hz), 131.3, 130.7, 127.3, 124.7 (dd, *J* = 21.7, 2.3 Hz), 124.5, 124.1 (dd, *J* = 21.8, 2.3 Hz), 121.5, 120.6 (d, *J* = 21.4 Hz), 120.0, 120.0 (d, *J* = 21.4 Hz), 119.8, 119.3, 118.9, 116.4, 115.2 (dd, *J* = 6.9, 3.3 Hz), 114.5, 113.6, 113.5 – 113.3 (m), 79.9, 72.6, 66.9, 39.2, 26.0, 14.6.

^{19}F NMR (471 MHz, CD_2Cl_2, 298 K, δ): –79.5 (s), –122.5 (m), –131.0 (m).

HRMS-ESI POS (m/z) calc'd for $C_{49}H_{34}F_8O_3S_4$ $[M-NTf_2]^+$, 475.0631; found, 475.0626; deviation: –0.9 ppm.

Benazepril methyl ester-derived thianthrenium salts (30-TT and 30-TT1)

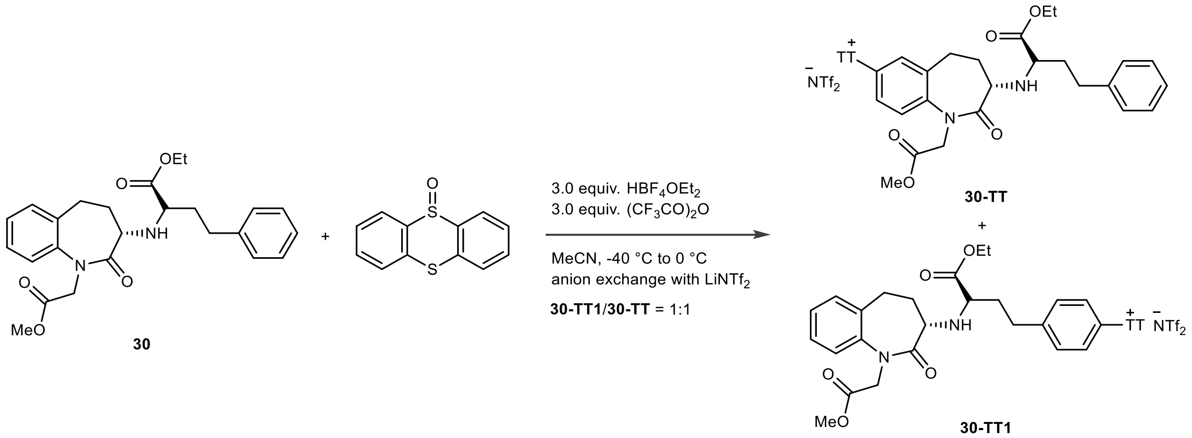

Under an ambient atmosphere, a 20 mL glass vial was charged with benazepril methyl ester **30** (50 mg, 0.11 mmol, 1.0 equiv.), thianthrenium-*S*-oxide (27 mg, 1.1 mmol, 1.0 equiv.), and dry MeCN (5 mL, c = 0.01 M). After cooling to –40 °C, $HBF_4{\cdot}OEt_2$ (55 mg, 47 µL, 0.34 mmol, 3.0 equiv.) was added to the vial while stirring the reaction mixture. Subsequently, trifluoroacetic anhydride (71 mg, 48 µL, 0.34 mmol, 3.0 equiv.) was added in one portion at –40 °C, resulting in a color change to deep purple. The vial was sealed with a screw-cap. The mixture was stirred at –40 °C for 3 h and then at 0 °C for 1 h. The solution was concentrated and the residue was

diluted with 5 mL dichloromethane and poured onto a mixture of 30 mL dichloromethane, 20 mL saturated aqueous $NaHCO_3$ solution, and 10 mL water. After stirring for 5 min at 25 °C, the mixture was poured into a separatory funnel, and the layers were separated. The dichloromethane layer was washed with aqueous $LiNTf_2$ solution (4 × ca. 20 mL, 5 % w/w). The dichloromethane layer was dried over Na_2SO_4, filtered, and the solvent was removed under reduced pressure. The residue was purified by chromatography on silica gel eluting with CH_2Cl_2/MeOH 25:1 (v/v) to afford 61.0 mg of the mixture of **30-TT** and **30-TT1** as a colorless solid. Further purification by preparative HPLC (YMC-Pack Triart C18 (250 × 4.60 mm: 5 µm), acetonitrile/0.1% TFA = 40:60, flow rate = 1.0 mL/min, 35 °C) provided **30-TT** as a colorless solid (28.1 mg, 30% yield) and **30-TT1** as a colorless solid (27.3 mg, 29% yield).

$\mathbf{R_f}$ = 0.41 (CH_2Cl_2/MeOH 9:1 (v:v))

The following data were obtained for the pure isomers:

Benazepril methyl ester-derived thianthrenium salt 30-TT

NMR Spectroscopy:

^{1}H NMR (600 MHz, CD_2Cl_2, 298 K, δ): 8.42 – 8.35 (m, 2H), 7.93 (dd, *J* = 7.9, 1.4 Hz, 2H), 7.89 (ddd, *J* = 8.0, 6.8, 1.4 Hz, 2H), 7.82 (td, *J* = 7.7, 1.5 Hz, 2H), 7.26 – 7.19 (m, 3H), 7.18 – 7.11 (m, 3H), 7.03 (d, J = 8.2 Hz, 2H), 4.52 (d, *J* = 17.4 Hz, 1H), 4.41 (d, *J* = 17.4 Hz, 1H), 4.07 – 3.96 (m, 2H), 3.70 – 3.65 (m, 3H), 3.15 (dt, *J* = 35.2, 6.4 Hz, 3H), 2.57 (d, *J* = 63.2 Hz, 3H), 2.29 (dd, *J* = 13.1, 7.0 Hz, 1H), 1.93 – 1.87 (m, 2H), 1.34 (t, J = 7.3 Hz, 1H), 1.12 (t, *J* = 7.1 Hz, 3H).

^{13}C NMR (151 MHz, CD_2Cl_2, 298 K, δ): 174.6, 174.0, 169.5, 146.7, 142.0, 141.9, 139.9, 137.4, 137.3, 136.5, 135.8, 135.2, 135.1, 131.3, 130.8, 129.8, 129.1, 128.8, 128.8, 128.7, 128.1, 128.0, 126.3, 126.3, 124.9, 123.5, 122.8, 121.4, 120.5, 119.2, 118.5, 118.3, 117.1, 61.1, 59.8, 57.0, 52.8, 50.6, 37.4, 35.4, 32.2, 29.1, 14.4.

^{19}F NMR (565 MHz, CD_2Cl_2, 298 K, δ): –79.4 (s).

HRMS-ESI POS (m/z) calc'd for $C_{37}H_{37}N_2S_2O_5$ [M-2NTf$_2$]$^+$, 653.2138; found, 653.2143; deviation: –0.7 ppm.

Benazepril methyl ester-derived thianthrenium salt 30-TT1

NMR Spectroscopy:

^{1}H NMR (600 MHz, CD_2Cl_2, 298 K, δ): 8.32 (dtd, *J* = 7.9, 1.3, 0.5 Hz, 2H), 7.93 (dtd, *J* = 7.9, 1.3, 0.4 Hz, 2H), 7.90 – 7.85 (m, 2H), 7.81 (ddt, *J* = 7.9, 7.4, 1.4 Hz, 2H), 7.35 – 7.31 (m, 2H), 7.27 (td, *J* = 7.6, 1.8 Hz, 1H), 7.22 (dd, *J* = 7.6, 1.8 Hz, 1H), 7.18 (td, *J* = 7.4, 1.2 Hz, 1H), 7.11 – 7.07 (m, 1H), 7.05 – 7.00 (m, 2H), 4.53 (d, *J* = 17.2 Hz, 1H), 4.44 (d, *J* = 17.2 Hz, 1H), 3.98 (q, *J* = 7.1 Hz, 2H), 3.27 – 3.21 (m, 1H), 3.14 (q, J = 5.6 Hz, 1H), 3.09 (dd, *J* = 7.1, 5.9 Hz, 1H), 2.72 (t, *J* = 8.0 Hz, 2H), 2.56 (dd, *J* = 13.5, 6.9 Hz, 1H), 2.30 (dq, *J* = 13.0, 6.8, 6.1 Hz, 1H), 1.95 – 1.75 (m, 5H), 1.36 – 1.29 (m, 1H), 1.07 (t, *J* = 7.1 Hz, 3H).

^{13}C NMR (151 MHz, CD_2Cl_2, 298 K, δ): 174.2, 174.1, 169.9, 149.1, 141.4, 137.3, 137.3, 136.4, 135.6, 134.8, 131.6, 131.2, 131.2, 130.7, 129.8, 128.2, 128.2, 127.2, 123.5, 122.7, 121.4, 120.4, 119.3, 118.8, 117.1, 61.1, 59.7, 57.1, 52.5, 50.7, 38.0, 34.6, 31.9, 28.7, 14.3.

^{19}F NMR (565 MHz, CD_2Cl_2, 298 K, δ): –79.4 (s).

HRMS-ESI POS (m/z) calc'd for $C_{37}H_{37}N_2S_2O_5$ [M-2NTf$_2$]$^+$, 653.2138; found, 653.2142; deviation: –0.7 ppm.

Hydrogenolysis of aryl (tetrafluoro)thianthrenium salts

4-[^{2}H]-Biphenyl ([^{2}H]1)

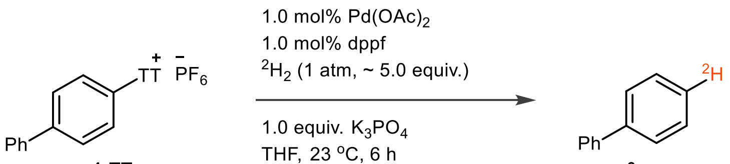

1-TT **[^{2}H]1**

Aryl thianthrenium salt **1-TT** (103 mg, 0.200 mmol, 1.00 equiv.), K_3PO_4 (42.5 mg, 0.200 mmol, 1.00 equiv.), and THF (0.5 mL, c = 0.2 M) were added to a 25-mL Schlenk tube containing a magnetic stir bar. Subsequently, a stock THF solution (0.5 mL) containing $Pd(OAc)_2$ (0.5 mg, 2 μmol, 1 mol%) and dppf (1.1 mg, 2.0 μmol, 1.0 mol%) was added to the reaction mixture. The Schlenk tube was then connected to a high vacuum line and a balloon containing $^2H_2$ (1 atm) via a T-bore glass stopcock adaptor (Figure S1). The reaction mixture was degassed via three freeze-pump-thaw cycles. After the third freeze-pump-thaw cycle, $^2H_2$ (1 atm) was introduced to the Schlenk tube while keeping the bottom of the Schlenk tube submersed in a water bath (23 °C). After the Schlenk tube was warmed to 23 °C, the tube was sealed, and the reaction mixture was stirred vigorously at 23 °C. After 12 hours, the reaction vessel was opened to air, and CH_2Cl_2 (5 mL) was added to the reaction mixture. The resulting mixture was concentrated by

rotary evaporation. The residue was purified by chromatography on silica gel, eluting with EtOAc/hexanes 1:50 (v/v) to afford 27.2 mg (87% yield) of the title compound as a colorless solid.

Deuterium incorporation: >0.99 ^{2}H/molecule (^{1}H NMR analysis).

R_f = 0.67 (hexanes/ether 20:1 (v:v))

NMR Spectroscopy:

^{1}H NMR (500 MHz, CD_2Cl_2, 298 K, δ): 7.65 – 7.60 (m, 4H), 7.49 – 7.42 (m, 4H), 7.39 – 7.33 (m, 1H).

^{2}H NMR (92 MHz, CH_2Cl_2, 298 K, δ): 7.40 (s).

^{13}C NMR (126 MHz, CD_2Cl_2, 298 K, δ): 141.5, 129.2, 127.7, 127.5, 127.4 (t, *J* = 24.6 Hz).

HRMS-EI (m/z) calc'd for $C_6H_3Cl_2D_1$ $[M]^+$, 155.0840; found, 155.0840; deviation: 0.0 ppm.

4-[^{2}H]-Fluorobenzene ([^{2}H]2)

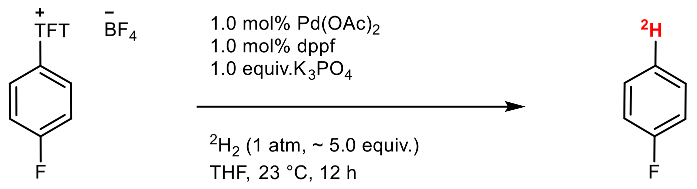

2-TFT **[^{2}H]2**

Aryl tetrafluorothianthrenium salt **2-TFT** (94.0 mg, 0.200 mmol, 1.00 equiv.), K_3PO_4 (42.5 mg, 0.200 mmol, 1.00 equiv.), and THF (0.5 mL) were added to a 25-mL Schlenk tube containing a magnetic stir bar. Subsequently, a THF solution (0.5 mL) containing $Pd(OAc)_2$ (0.5 mg, 2 μmol, 1 mol%) and dppf (1.1 mg, 2.0 μmol, 1.0 mol%) was added to the reaction mixture. The Schlenk tube was then connected to a high vacuum line and a balloon containing $^{2}H_2$ (1 atm) via a T-bore glass stopcock adaptor. The reaction mixture was degassed via three freeze-pump-thaw cycles. After the third freeze-pump-thaw cycle, $^{2}H_2$ (1 atm) was introduced to the Schlenk tube while keeping the bottom of the Schlenk tube submersed in a water bath (23 °C). The tube was sealed, and the reaction mixture was stirred vigorously at 23 °C. After being stirred for 12 hours, the reaction vessel was opened to air and pentane (5 mL) was added to the reaction mixture. 4-Fluorobenzotrifluoride (32.8 mg, 25.4 μL, 0.200 mmol, 1.00 equiv.) was added as an internal standard. Due to the volatility of the product, the yield of the known compounds **[^{2}H]2**[9] was

determined by ^{19}F NMR integration relative to the internal standard (71% yield, standard: δ –107.97 ppm, and 4-[^{2}H]-fluorobenzene: δ –113.04 (m) ppm), and the deuterium incorporation was determined by mass analysis (100.5 ± 0.8%). The identity of the product was further confirmed by HRMS analysis.

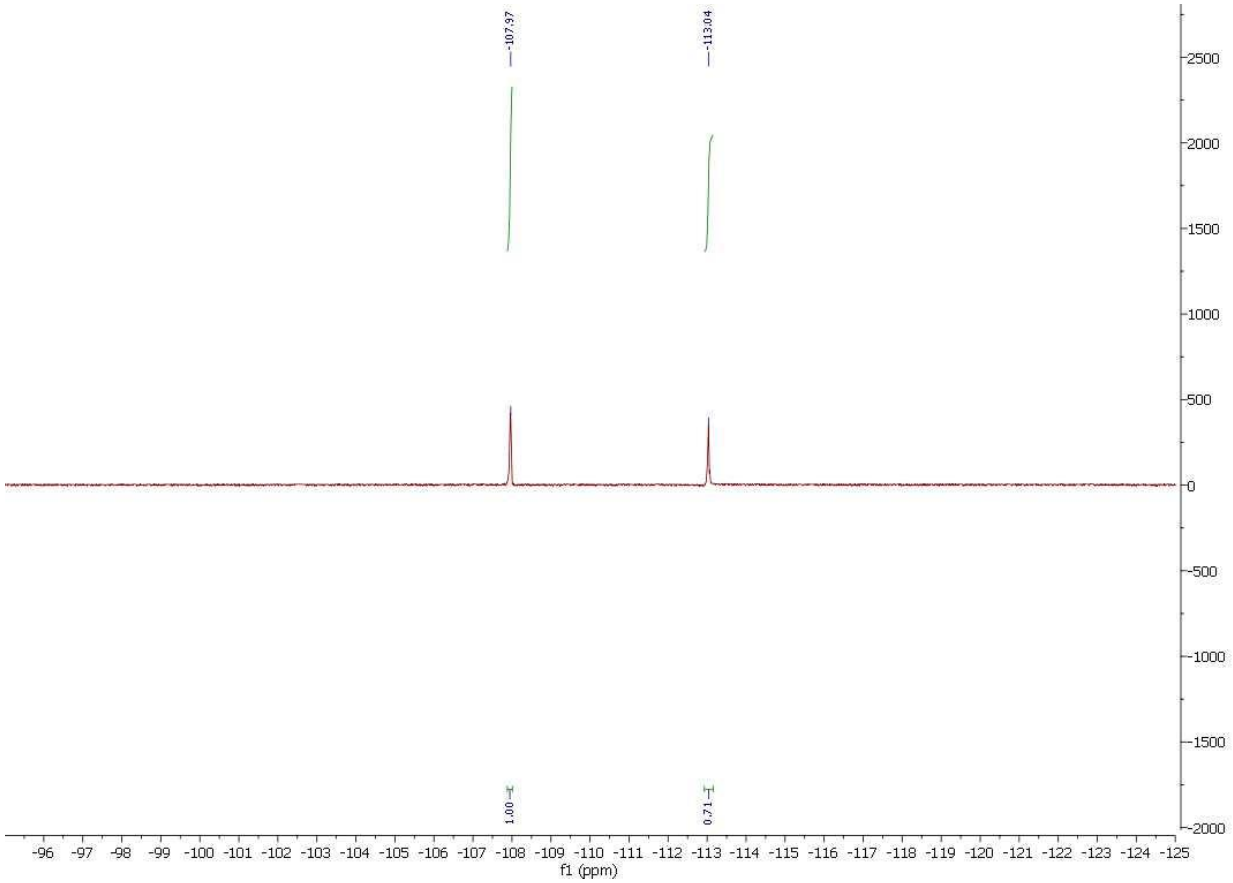

Figure S26. ^{19}F NMR spectrum of the reaction mixture of 4-fluorobenzene-derived tetrafluorothianthrenium salt with 4-fluorobenzotrifluoride as internal standard.

Deuterium incorporation: 100.5 ± 0.8 ^{2}H/molecule (mass analysis)

HRMS-EI (m/z) calc'd for $C_6H_4D_1F_1$ $[M]^+$, 97.0433; found, 97.0435; deviation: –2.0 ppm.

2-Fluoro-(*p*-[^{2}H]phenoxy)benzonitrile ([^{2}H]3)

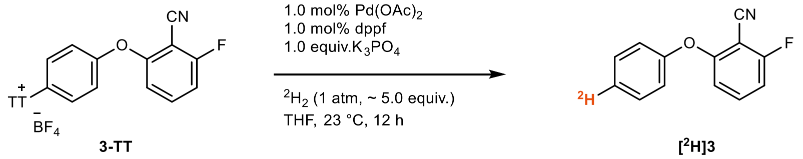

3-TT **[^{2}H]3**

Aryl thianthrenium salt **3-TT** (117 mg, 0.200 mmol, 1.00 equiv.), K_3PO_4 (42.5 mg, 0.200 mmol, 1.00 equiv.), and THF (0.5 mL, c = 0.2 M) were added to a 25-mL Schlenk tube containing a magnetic stir bar. Subsequently, a stock THF solution (0.5 mL) containing $Pd(OAc)_2$ (0.5 mg, 2 μmol, 1 mol%) and dppf (1.1 mg, 2.0 μmol, 1.0 mol%) was added to the reaction mixture. The Schlenk tube was then connected to a high vacuum line and a balloon containing $^2H_2$ (1 atm) via a T-bore glass stopcock adaptor (Figure S1). The reaction mixture was degassed via three freeze-pump-thaw cycles. After the third freeze-pump-thaw cycle, $^2H_2$ (1 atm) was introduced to the Schlenk tube while keeping the bottom of the Schlenk tube submersed in a water bath (23 °C). After the Schlenk tube was warmed to 23 °C, the tube was sealed, and the reaction mixture was stirred vigorously at 23 °C. After 12 hours, the reaction vessel was opened to air, and CH_2Cl_2 (5 mL) was added to the reaction mixture. The resulting mixture was concentrated by rotary evaporation. The residue was purified by chromatography on silica gel, eluting with EtOAc/hexanes 1:20 (v/v) to afford 39.7 mg (93% yield) of the title compound as a colorless solid.

Deuterium incorporation: 0.98 ^{2}H/molecule (^{1}H NMR)

R_f = 0.29 (hexanes/EtOAc, 5:1 (v:v))

NMR Spectroscopy:

^{1}H NMR (500 MHz, CD_3CN, 298 K, δ): 7.55 (td, *J* = 8.6, 6.7 Hz, 1H), 7.48 (dq, *J* = 7.4, 1.0 Hz, 2H), 7.20 – 7.13 (m, 2H), 7.02 (td, *J* = 8.6, 0.8 Hz, 1H), 6.67 (dt, *J* = 8.6, 0.9 Hz, 1H).

^{2}H NMR (92 MHz, CH_3CN, 298 K, δ): 7.36 (s).

^{13}C NMR (126 MHz, CD_3CN, 298 K, δ): 164.6 (d, *J* = 256.3 Hz), 161.8 (d, *J* = 4.1 Hz), 155.7, 136.6 (d, *J* = 10.7 Hz), 131.4, 126.5 (t, *J* = 23.9 Hz), 121.1, 113.6 (d, *J* = 3.5 Hz), 112.3, 110.6 (d, *J* = 19.6 Hz), 93.9 (d, *J* = 18.0 Hz).

^{19}F NMR (471 MHz, CD_3CN, 298 K, δ): –107.9 (dd, *J* = 9.1, 6.6 Hz).

HRMS-EI (m/z) calc'd for $C_{13}H_7O_1N_1D_1F_1$ $[M]^+$, 214.0647; found, 214.0647; deviation: –0.1 ppm.

3-Phenyl-3-(4-[^{2}H]-phenyl) propan-1-ol ([^{2}H]4)

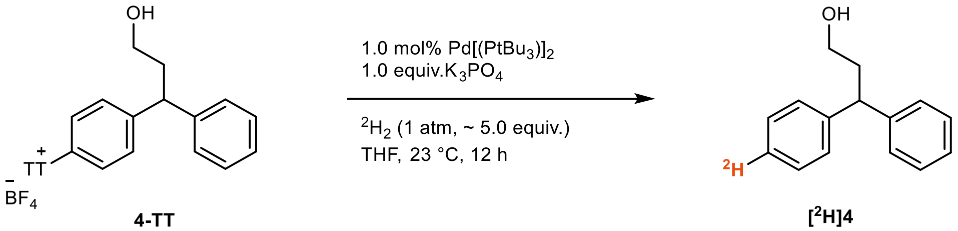

4-TT **[^{2}H]4**

Aryl thianthrenium salt **4-TT** (118 mg, 0.200 mmol, 1.00 equiv.), K_3PO_4 (42.5 mg, 0.200 mmol, 1.00 equiv.), and THF (0.5 mL, c = 0.2 M) were added to a 25-mL Schlenk tube containing a magnetic stir bar. Subsequently, a stock THF solution (0.5 mL) containing Pd[(P*t*Bu$_3$)]$_2$ (1.0 mg, 2.0 μmol, 1.0 mol%) was added to the reaction mixture. The Schlenk tube was then connected to a high vacuum line and a balloon containing $^2H_2$ (1 atm) via a T-bore glass stopcock adaptor (Figure S1). The reaction mixture was degassed via three freeze-pump-thaw cycles. After the third freeze-pump-thaw cycle, $^2H_2$ (1 atm) was introduced to the Schlenk tube while keeping the bottom of the Schlenk tube submersed in a water bath (23 °C). After the Schlenk tube was warmed to 23 °C, the tube was sealed, and the reaction mixture was stirred vigorously at 23 °C. After 12 hours, the reaction vessel was opened to air, and CH_2Cl_2 (5 mL) was added to the reaction mixture. The resulting mixture was concentrated by rotary evaporation. The residue was purified by chromatography on silica gel, eluting with EtOAc/hexanes 1:20 (v/v) to afford 35.4 mg (83% yield) of the title compound as a colorless oil.

Deuterium incorporation: >0.99 ^{2}H/molecule (^{1}H NMR)

R$_f$ = 0.18 (pentane/EtOAc, 5:1 (v:v))

NMR Spectroscopy:

^{1}H NMR (500 MHz, CD_2Cl_2, 298 K, δ): 7.31 – 7.28 (m, 9H), 7.20 – 7.17 (m, 1H), 4.13 (t, *J* = 7.9 Hz, 1H), 3.56 (td, *J* = 6.3, 4.0 Hz, 2H), 2.33 – 2.29 (m, 2H), 1.37 (d, *J* = 4.8 Hz, 1H).

^{2}H NMR (92 MHz, CH_2Cl_2, 298 K, δ): 7.23 (s).

^{13}C NMR (126 MHz, CD_2Cl_2, 298 K, δ): 145.2, 128.9, 128.8, 126.6, 126.3 (t, *J* = 24.6 Hz), 61.2, 47.8, 38.6.

HRMS-ESI POS (m/z) calc'd for $C_{15}H_{15}D_1O_1Na_1$ $[M+Na]^+$, 236.1156; found, 236.1159; deviation: –1.1 ppm.

4-[^{2}H]-Benzyloxazolidinone ([^{2}H]5)

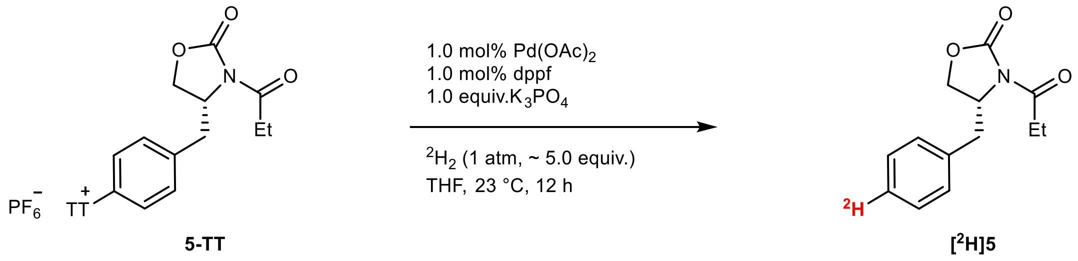

Aryl thianthrenium salt **5-TT** (119 mg, 0.200 mmol, 1.00 equiv.), K_3PO_4 (42.5 mg, 0.200 mmol, 1.00 equiv.), and THF (0.5 mL, c = 0.2 M) were added to a 25-mL Schlenk tube containing a magnetic stir bar. Subsequently, a stock THF solution (0.5 mL) containing $Pd(OAc)_2$ (0.5 mg, 2 µmol, 1 mol%) and dppf (1.1 mg, 2.0 µmol, 1.0 mol%) was added to the reaction mixture. The Schlenk tube was then connected to a high vacuum line and a balloon containing $^2H_2$ (1 atm) via a T-bore glass stopcock adaptor (Figure S1). The reaction mixture was degassed via three freeze-pump-thaw cycles. After the third freeze-pump-thaw cycle, $^2H_2$ (1 atm) was introduced to the Schlenk tube while keeping the bottom of the Schlenk tube submersed in a water bath (23 °C). After the Schlenk tube was warmed to 23 °C, the tube was sealed, and the reaction mixture was stirred vigorously at 23 °C. After 12 hours, the reaction vessel was opened to air, and CH_2Cl_2 (5 mL) was added to the reaction mixture. The resulting mixture was concentrated by rotary evaporation. The residue was purified by chromatography on silica gel, eluting with EtOAc/hexanes 1:10 (v/v) to afford 37.0 mg (79% yield) of the title compound as a colorless oil.

Deuterium incorporation: >0.99 ^{2}H/molecule (^{1}H NMR)

R$_f$ = 0.58 (hexanes/EtOAc, 2:1 (v:v))

NMR Spectroscopy:

^{1}H NMR (500 MHz, CD_2Cl_2, 298 K, δ): 7.34 (d, *J* = 8.1 Hz, 2H), 7.23 – 7.20 (d, *J* = 8.0 Hz, 2H), 4.68 (ddt, *J* = 9.1, 7.8, 3.1 Hz, 1H), 4.22 – 4.19 (m, 1H), 4.16 (dd, *J* = 9.1, 2.9 Hz, 1H), 3.23 (ddd, *J* = 13.4, 3.5, 0.7 Hz, 1H), 2.92 (dq, *J* = 13.0, 7.3 Hz, 2H), 2.84 (dd, *J* = 13.5, 9.1 Hz, 1H), 1.18 (t, *J* = 7.4 Hz, 3H).

^{2}H NMR (92 MHz, CH_2Cl_2, 298 K, δ): 7.33 (s).

^{13}C NMR (126 MHz, CD_2Cl_2, 298 K, δ): 174.2, 153.9, 136.0, 129.9, 129.1, 127.3 (t, *J* = 24.6 Hz), 66.7, 55.4, 38.1, 29.5, 8.5.

HRMS-EI (m/z) calc'd for $C_{13}H_{14}D_1N_1O_3$ $[M]^+$, 234.1109; found, 234.1111; deviation: –0.7 ppm.

[^{2}H]Amiodarone ([^{2}H]6)

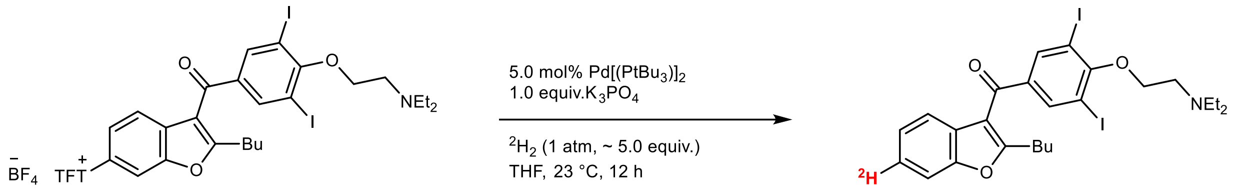

6-TFT [^{2}H]6

Aryl tetrafluorothianthrenium salt **6-TFT** (102 mg, 0.100 mmol, 1.00 equiv.), K_3PO_4 (21.3 mg, 0.100 mmol, 1.00 equiv.), and THF (0.25 mL, c = 0.20 M) were added to a 10-mL Schlenk tube containing a magnetic stir bar. Subsequently, a stock THF solution (0.25 mL) containing Pd[(P*t*Bu$_3$)]$_2$ (2.5 mg, 5.0 μmol, 5.0 mol%) was added to the reaction mixture. The Schlenk tube was then connected to a high vacuum line and a balloon containing $^2H_2$ (1 atm) via a T-bore glass stopcock adaptor (Figure S1). The reaction mixture was degassed via three freeze-pump-thaw cycles. After the third freeze-pump-thaw cycle, $^2H_2$ (1 atm) was introduced to the Schlenk tube while keeping the bottom of the Schlenk tube submersed in a water bath (23 °C). After the Schlenk tube was warmed to 23 °C, the tube was sealed, and the reaction mixture was stirred vigorously at 23 °C. After 12 hours, the reaction vessel was opened to air, and CH_2Cl_2 (5 mL) was added to the reaction mixture. The resulting mixture was concentrated by rotary evaporation. The residue was purified by chromatography on silica gel, eluting with DCM/MeOH 20:1 (v/v) to afford 44.2 mg (68% yield) of the title compound as a colorless solid.

Deuterium incorporation: >0.99 ^{2}H/molecule (^{1}H NMR)

R$_f$ = 0.21 (DCM/MeOH, 10:1 (v:v))

NMR Spectroscopy:

^{1}H NMR (500 MHz, CD_2Cl_2, 298 K, δ): 8.21 (s, 2H), 7.50 (s, 1H), 7.43 (d, *J* = 7.9 Hz, 1H), 7.24 (dd, *J* = 7.9, 1.0 Hz, 1H), 4.15 (t, *J* = 6.6 Hz, 2H), 3.09 (t, *J* = 6.6 Hz, 2H), 2.85 – 2.81 (m, 2H), 2.74 (q, *J* = 7.1 Hz, 4H), 1.75 (ddt, *J* = 9.0, 7.6, 3.7 Hz, 2H), 1.38 – 1.32 (m, 2H), 1.12 (t, *J* = 7.1 Hz, 6H), 0.91 (t, *J* = 7.4 Hz, 3H).

^{2}H NMR (92 MHz, CH_2Cl_2, 298 K, δ): 7.37 (s).

^{13}C NMR (126 MHz, CD_2Cl_2, 298 K, δ): 188.0, 166.4, 161.8, 154.2, 141.1, 138.8, 127.0, 124.0, 121.4, 116.3, 111.3, 91.1, 71.8, 52.5, 48.2, 30.4, 28.5, 22.9, 13.9, 12.1.

HRMS-ESI POS (m/z) calc'd for $C_{25}H_{29}D_1I_2N_1O_3$ $[M+H]^+$, 647.0372; found, 647.0376; deviation: –0.5 ppm.

4-[^{2}H]-Chlorobenzene ([^{2}H]7)

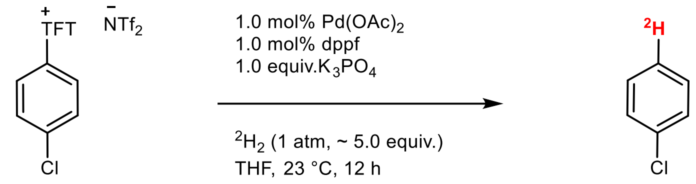

7-TFT **[^{2}H]7**

Aryl tetrafluorothianthrenium salt **7-TFT** (186 mg, 0.200 mmol, 1.00 equiv.), K_3PO_4 (42.5 mg, 0.200 mmol, 1.00 equiv.), and THF (0.5 mL, c = 0.2 M) were added to a 25-mL Schlenk tube containing a magnetic stir bar. Subsequently, a THF solution (0.5 mL) containing $Pd(OAc)_2$ (0.5 mg, 2 μmol, 1 mol%) and dppf (1.1 mg, 2.0 μmol, 1.0 mol%) was added to the reaction mixture. The Schlenk tube was then connected to a high vacuum line and a balloon containing $^2H_2$ (1 atm) via a T-bore glass stopcock adaptor. The reaction mixture was degassed via three freeze-pump-thaw cycles. After the third freeze-pump-thaw cycle, $^2H_2$ (1 atm) was introduced to the Schlenk tube while keeping the bottom of the Schlenk tube submersed in a water bath (23 °C). The tube was sealed, and the reaction mixture was stirred vigorously at 23 °C. After being stirred for 12 hours, the reaction vessel was opened to air and concentrated by rotary evaporation at 0 °C. The residue was diluted with benzene-d_6 (1 mL), and mesitylene (24.0 mg, 27.8 µL, 0.200 mmol, 1.0 equiv.) was added as an internal standard. Due to the volatility of the product, the yield of the known compounds **[^{2}H]7**[10] was determined by ^{1}H NMR integration relative to the internal standard (98% yield, standard: δ 6.72 ppm, and 4-[^{2}H]-chlorobenzene: δ 6.82 (m) ppm), and the deuterium incorporation was determined by mass analysis (97.7 ± 1.1%). The identity of the product was further confirmed by HRMS analysis.

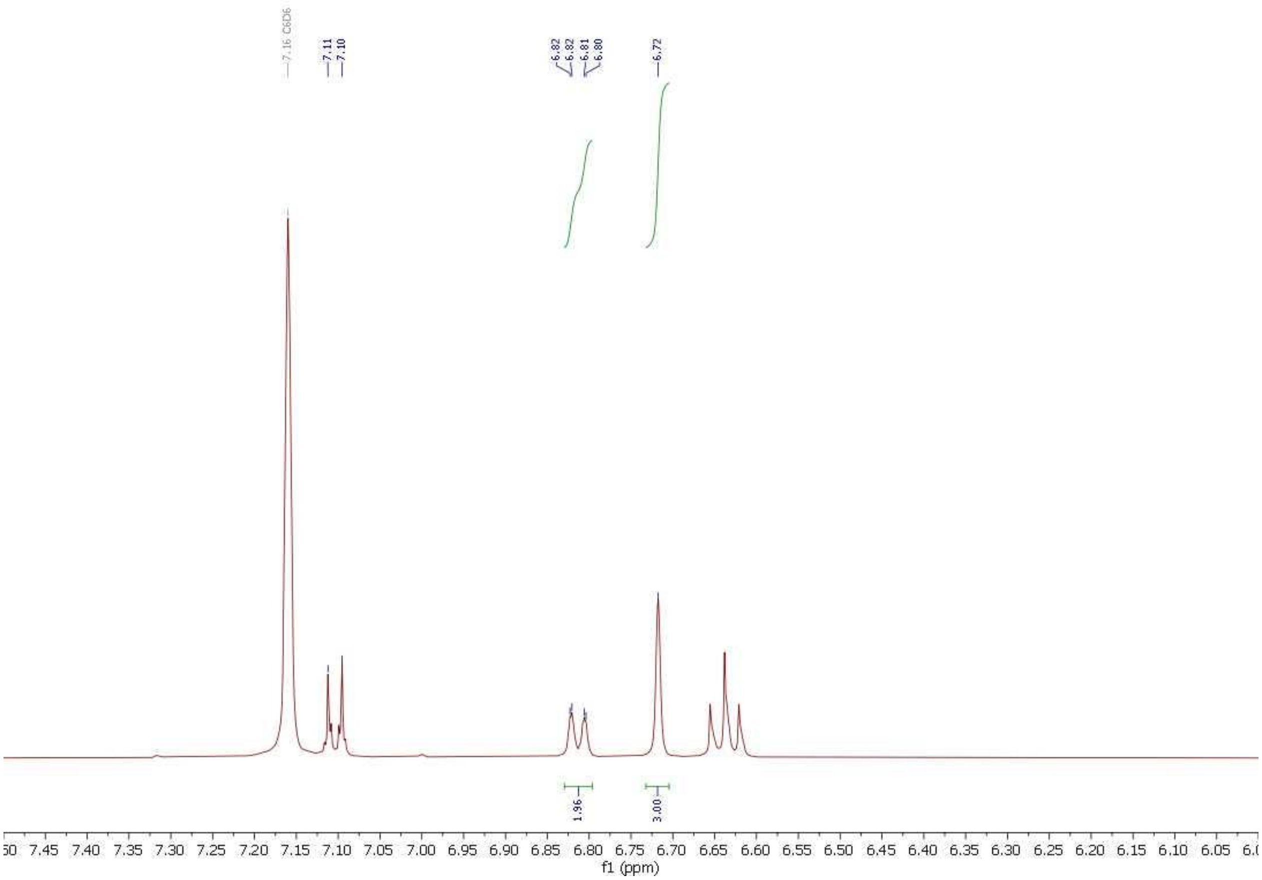

Figure S27. ^{1}H NMR spectrum of the reaction mixture of 4-chlorobenzene-derived tetrafluorothianthrenium salt with mesitylene as internal standard.

Deuterium incorporation: 97.7 ± 1.1 ^{2}H/molecule (mass analysis)

HRMS-EI (m/z) calc'd for $C_6H_4D_1Cl_1$ $[M]^+$, 112.0074; found, 112.0076; deviation: –1.2 ppm.

1-(4-Bromophenoxy)-4-[^{2}H]-benzene ([^{2}H]8)

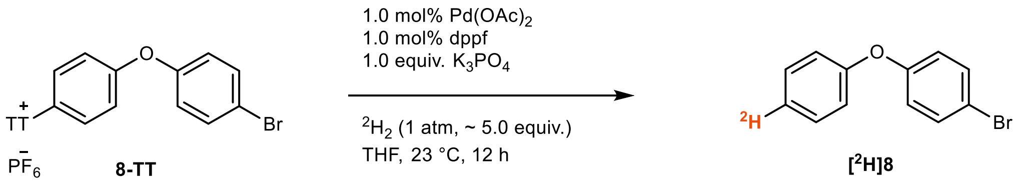

Aryl thianthrenium salt **8-TT** (121 mg, 0.200 mmol, 1.00 equiv.), K_3PO_4 (42.5 mg, 0.200 mmol, 1.00 equiv.) and THF (0.5 mL, c = 0.2 M) were added to a 25-mL Schlenk tube containing a magnetic stir bar. Subsequently, a stock THF solution (0.5 mL) containing $Pd(OAc)_2$ (0.5 mg, 2 μmol, 1 mol%) and dppf (1.1 mg, 2.0 μmol, 1.0 mol%) was added to the reaction mixture. The

Schlenk tube was then connected to a high vacuum line and a balloon containing $^{2}H_2$ (1 atm) via a T-bore glass stopcock adaptor (Figure S1). The reaction mixture was degassed via three freeze-pump-thaw cycles. After the third freeze-pump-thaw cycle, $^{2}H_2$ (1 atm) was introduced to the Schlenk tube while keeping the bottom of the Schlenk tube submersed in a water bath (23 °C). After the Schlenk tube was warmed to 23 °C, the tube was sealed, and the reaction mixture was stirred vigorously at 23 °C. After 12 hours, the reaction vessel was opened to air, and CH_2Cl_2 (5 mL) was added to the reaction mixture. The resulting mixture was concentrated by rotary evaporation. The residue was purified by chromatography on silica gel, eluting with ether/hexanes 1:20 (v/v) to afford 38.1 mg (76% yield) of the title compound as a colorless oil.

Deuterium incorporation: >0.99 ^{2}H/molecule (^{1}H NMR)

R$_f$ = 0.72 (hexanes/Ether, 20:1 (v:v))

NMR Spectroscopy:

^{1}H NMR (500 MHz, CD_3CN, 298 K, δ): 7.52 – 7.46 (m, 2H), 7.39 (dq, *J* = 7.8, 1.1 Hz, 2H), 7.04 – 7.00 (m, 2H), 6.96 – 6.89 (m, 2H).

^{2}H NMR (92 MHz, CH_3CN, 298 K, δ): 7.22 (s).

^{13}C NMR (126 MHz, CD_3CN, 298 K, δ): 157.8, 157.6, 133.7, 130.9, 121.6 (t, *J* = 25.2 Hz), 120.0, 116.1.

HRMS-EI (m/z) calc'd for $C_{12}H_8D_1O_1Br_1$ $[M]^+$, 248.9894; found, 248.9898; deviation: –1.6 ppm.

N-(4-[^{2}H]-Phenyl)benzamide ([^{2}H]9)

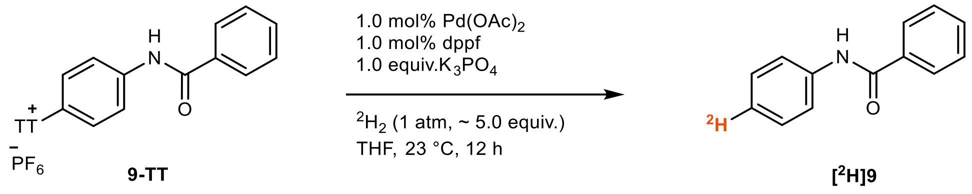

Aryl thianthrenium salt **9-TT** (111 mg, 0.200 mmol, 1.00 equiv.), K_3PO_4 (42.5 mg, 0.200 mmol, 1.00 equiv.), and THF (0.5 mL, c = 0.2 M) were added to a 25-mL Schlenk tube containing a magnetic stir bar. Subsequently, a stock THF solution (0.5 mL) containing $Pd(OAc)_2$ (0.5 mg, 2 μmol, 1 mol%) and dppf (1.1 mg, 2.0 μmol, 1.0 mol%) was added to the reaction mixture. The Schlenk tube was then connected to a high vacuum line and a balloon containing $^{2}H_2$ (1 atm) via

a T-bore glass stopcock adaptor (Figure S1). The reaction mixture was degassed via three freeze-pump-thaw cycles. After the third freeze-pump-thaw cycle, $^2H_2$ (1 atm) was introduced to the Schlenk tube while keeping the bottom of the Schlenk tube submersed in a water bath (23 °C). After the Schlenk tube was warmed to 23 °C, the tube was sealed, and the reaction mixture was stirred vigorously at 23 °C. After 12 hours, the reaction vessel was opened to air, and CH_2Cl_2 (5 mL) was added to the reaction mixture. The resulting mixture was concentrated by rotary evaporation. The residue was purified by chromatography on silica gel, eluting with EtOAc/hexanes 1:30 (v/v) to afford 34.6 mg (87% yield) of the title compound as a colorless solid.

Deuterium incorporation: >0.99 ^{2}H/molecule (^{1}H NMR)

R_f = 0.23 (hexanes/EtOAc, 5:1 (v:v))

NMR Spectroscopy:

^{1}H NMR (500 MHz, CD_2Cl_2, 298 K, δ): 7.94 (br, 1H, N*H*), 7.88 – 7.86 (m, 2H), 7.66 – 7.64 (m, 2H), 7.60 – 7.55 (m, 1H), 7.53 – 7.46 (m, 2H), 7.39 – 7.36 (m, 2H).

^{2}H NMR (92 MHz, CH_2Cl_2, 298 K, δ): 7.21 (s).

^{13}C NMR (126 MHz, CD_2Cl_2, 298 K, δ): 166.0, 138.6, 135.5, 132.2, 129.3, 129.1, 127.4, 120.6.

HRMS-EI (m/z) calc'd for $C_{13}H_{10}D_1O_1N_1$ $[M]^+$, 198.0898; found, 198.0901; deviation: –1.7 ppm.

[^{2}H]Nefiracetam ([^{2}H]10)

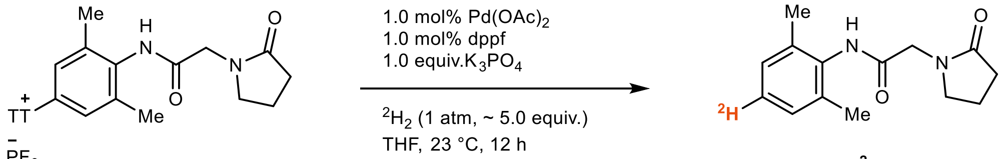

10-TT **[^{2}H]10**

Aryl thianthrenium salt **10-TT** (121 mg, 0.200 mmol, 1.00 equiv.), K_3PO_4 (42.5 mg, 0.200 mmol, 1.00 equiv.), and THF (0.5 mL, c = 0.2 M) were added to a 25-mL Schlenk tube containing a magnetic stir bar. Subsequently, a stock THF solution (0.5 mL) containing $Pd(OAc)_2$ (0.5 mg, 2 µmol, 1 mol%) and dppf (1.1 mg, 2.0 µmol, 1.0 mol%) was added to the reaction mixture. The Schlenk tube was then connected to a high vacuum line and a balloon containing $^2H_2$ (1 atm) via a T-bore glass stopcock adaptor (Figure S1). The reaction mixture was degassed via three

freeze-pump-thaw cycles. After the third freeze-pump-thaw cycle, $^2H_2$ (1 atm) was introduced to the Schlenk tube while keeping the bottom of the Schlenk tube submersed in a water bath (23 °C). After the Schlenk tube was warmed to 23 °C, the tube was sealed, and the reaction mixture was stirred vigorously at 23 °C. After 12 hours, the reaction vessel was opened to air, and CH_2Cl_2 (5 mL) was added to the reaction mixture. The resulting mixture was concentrated by rotary evaporation. The residue was purified by chromatography on silica gel, eluting with DCM/MeOH 20:1 (v/v) to afford 36.7 mg (74% yield) of the title compound as a colorless solid.

Deuterium incorporation: 0.98 D/molecule (^{1}H NMR)

R_f = 0.21 (DCM/MeOH, 10:1 (v:v))

NMR Spectroscopy:

^{1}H NMR (500 MHz, CD_3CN, 298 K, δ): 7.97 (br, 1H, N*H*), 7.08 (s, 2H), 4.01 (s, 2H), 3.51 – 3.48 (m, 2H), 2.31 (dd, *J* = 8.7, 7.6 Hz, 2H), 2.16 (s, 6H), 2.08 – 2.01 (m, 2H).

^{2}H NMR (92 MHz, CH_3CN, 298 K, δ): 7.15 (s).

^{13}C NMR (126 MHz, CD_3CN, 298 K, δ): 176.5, 167.9, 136.8, 135.3, 128.7, 127.8 (t, *J* = 23.9 Hz), 48.8, 47.0, 30.8, 18.5, 18.4.

HRMS-EI (m/z) calc'd for $C_{14}H_{17}D_1N_2O_2$ $[M]^+$, 247.1426; found, 247.1426; deviation: –0.3 ppm.

[^{2}H](±)-Pyriproxyfen ([^{2}H]11)

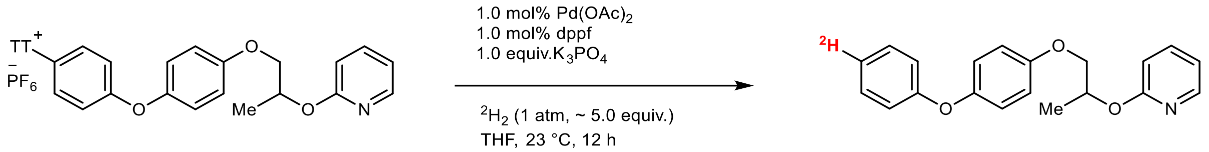

Aryl thianthrenium salt **11-TT** (136 mg, 0.200 mmol, 1.00 equiv.), K_3PO_4 (42.5 mg, 0.200 mmol, 1.00 equiv.), and THF (0.5 mL, c = 0.2 M) were added to a 25-mL Schlenk tube containing a magnetic stir bar. Subsequently, a stock THF solution (0.5 mL) containing $Pd(OAc)_2$ (0.5 mg, 2 μmol, 1 mol%) and dppf (1.1 mg, 2.0 μmol, 1.0 mol%) was added to the reaction mixture. The Schlenk tube was then connected to a high vacuum line and a balloon containing $^2H_2$ (1 atm) via a T-bore glass stopcock adaptor (Figure S1). The reaction mixture was degassed via three freeze-pump-thaw cycles. After the third freeze-pump-thaw cycle, $^2H_2$ (1 atm) was introduced to the Schlenk tube while keeping the bottom of the Schlenk tube submersed in a water bath (23

°C). After the Schlenk tube was warmed to 23 °C, the tube was sealed, and the reaction mixture was stirred vigorously at 23 °C. After 12 hours, the reaction vessel was opened to air, and CH_2Cl_2 (5 mL) was added to the reaction mixture. The resulting mixture was concentrated by rotary evaporation. The residue was purified by chromatography on silica gel, eluting with EtOAc/hexanes 1:20 (v/v) to afford 63.5 mg (98% yield) of the title compound as a colorless solid.

Deuterium incorporation: >0.99 ^{2}H/molecule (^{1}H NMR)

R_f = 0.43 (hexanes/EtOAc, 5:1 (v:v))

NMR Spectroscopy:

^{1}H NMR (500 MHz, CD_3CN, 298 K, δ): 8.13 (ddd, *J* = 5.0, 2.1, 0.8 Hz, 1H), 7.65 – 7.60 (m, 1H), 7.31 (d, *J* = 8.3 Hz, 2H), 6.95 – 6.90 (m, 6H), 6.74 – 6.70 (m, 1H), 5.56 (td, *J* = 6.2, 4.1 Hz, 1H), 4.16 (dd, *J* = 10.3, 5.9 Hz, 1H), 4.09 (dd, *J* = 10.3, 4.1 Hz, 1H), 2.19 (s, 1H), 1.40 (d, *J* = 6.4 Hz, 3H).

^{2}H NMR (92 MHz, CH_3CN, 298 K, δ): 7.12 (s).

^{13}C NMR (126 MHz, CD_3CN, 298 K, δ): 164.1, 159.4, 156.2, 151.2, 147.8, 140.1, 130.6, 123.3 (t, *J* = 24.6 Hz), 121.7, 118.4, 117.9, 116.7, 112.2, 71.9, 70.3, 17.0.

HRMS-ESI POS (m/z) calc'd for $C_{20}H_{19}D_1N_1O_3$ $[M+H]^+$, 323.1500; found, 323.1501; deviation: –0.3 ppm.

4'-[^{2}H]-[1,1'-Biphenyl]-4-yl trifluoromethanesulfonate ([^{2}H]12)

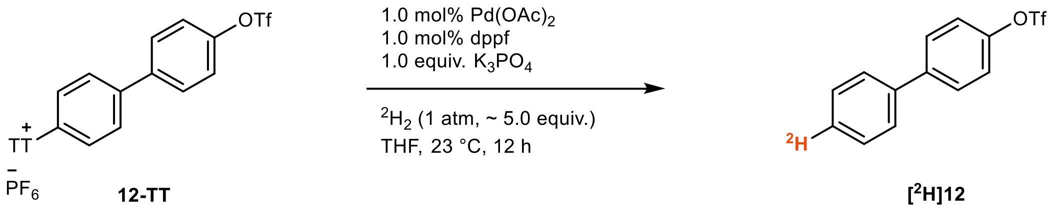

Aryl thianthrenium salt **12-TT** (133 mg, 0.200 mmol, 1.00 equiv.), K_3PO_4 (42.5 mg, 0.200 mmol, 1.00 equiv.), and THF (0.5 mL, c = 0.2 M) were added to a 25-mL Schlenk tube containing a magnetic stir bar. Subsequently, a stock THF solution (0.5 mL) containing $Pd(OAc)_2$ (0.5 mg, 2 μmol, 1 mol%) and dppf (1.1 mg, 2.0 μmol, 1.0 mol%) was added to the reaction mixture. The Schlenk tube was then connected to a high vacuum line and a balloon containing $^2H_2$ (1 atm) via

a T-bore glass stopcock adaptor (Figure S1). The reaction mixture was degassed via three freeze-pump-thaw cycles. After the third freeze-pump-thaw cycle, $^{2}H_2$ (1 atm) was introduced to the Schlenk tube while keeping the bottom of the Schlenk tube submersed in a water bath (23 °C). After the Schlenk tube was warmed to 23 °C, the tube was sealed, and the reaction mixture was stirred vigorously at 23 °C. After 12 hours, the reaction vessel was opened to air, and CH_2Cl_2 (5 mL) was added to the reaction mixture. The resulting mixture was concentrated by rotary evaporation. The residue was purified by chromatography on silica gel, eluting with EtOAc/hexanes 1:20 (v/v) to afford 52.8 mg (87% yield) of the title compound as a colorless solid.

Deuterium incorporation: >0.99 ^{2}H/molecule (^{1}H NMR)

R_f = 0.21 (hexanes)

NMR Spectroscopy:

^{1}H NMR (500 MHz, CD_2Cl_2, 298 K, δ): 7.71 – 7.67 (m, 2H), 7.61 – 7.57 (m, 2H), 7.50 – 7.44 (m, 2H), 7.40 – 7.34 (m, 2H).

^{2}H NMR (92 MHz, CH_2Cl_2, 298 K, δ): 7.46 (s).

^{13}C NMR (126 MHz, CD_2Cl_2, 298 K, δ): 149.4, 142.1, 139.7, 129.3, 129.3, 128.2 (t, J = 25.2 Hz), 127.6, 122.0, 119.2 (q, J = 241.0 Hz).

^{19}F NMR (471 MHz, CD_2Cl_2, 298 K, δ): –73.2 (s).

HRMS-EI (m/z) calc'd for $C_{13}H_8D_1S_1O_3F_3$ $[M]^+$, 303.0282; found, 303.0285; deviation: –1.0 ppm.

[^{2}H]-Tetrahydrobenzofuranone ([^{2}H]13)

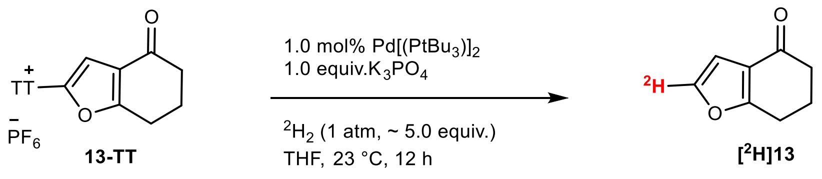

Aryl thianthrenium salt **13-TT** (99.3 mg, 0.200 mmol, 1.00 equiv.), K_3PO_4 (42.5 mg, 0.200 mmol, 1.00 equiv.), and THF (0.5 mL, c = 0.2 M) were added to a 25-mL Schlenk tube containing a magnetic stir bar. Subsequently, a stock THF solution (0.5 mL) containing Pd[(P*t*Bu$_3$)]$_2$ (1.0 mg, 2.0 µmol, 1.0 mol%) was added to the reaction mixture. The Schlenk tube was then connected

to a high vacuum line and a balloon containing $^2H_2$ (1 atm) via a T-bore glass stopcock adaptor (Figure S1). The reaction mixture was degassed via three freeze-pump-thaw cycles. After the third freeze-pump-thaw cycle, $^2H_2$ (1 atm) was introduced to the Schlenk tube while keeping the bottom of the Schlenk tube submersed in a water bath (23 °C). After the Schlenk tube was warmed to 23 °C, the tube was sealed, and the reaction mixture was stirred vigorously at 23 °C. After 12 hours, the reaction vessel was opened to air, and CH_2Cl_2 (5 mL) was added to the reaction mixture. The resulting mixture was concentrated by rotary evaporation. The residue was purified by chromatography on silica gel, eluting with EtOAc/hexanes 1:20 (v/v) to afford 22.0 mg (80% yield) of the title compound as a colorless oil.

Deuterium incorporation: >0.99 ^{2}H/molecule (^{1}H NMR)

R_f = 0.20 (hexanes/EtOAc, 5:1 (v:v))

NMR Spectroscopy:

^{1}H NMR (500 MHz, CD_3CN, 298 K, δ): 6.62 (s, 1H), 2.86 (t, *J* = 6.3 Hz, 2H), 2.45 (dd, *J* = 7.2, 5.8 Hz, 2H), 2.17 – 2.14 (m, 2H).

^{2}H NMR (92 MHz, CH_3CN, 298 K, δ): 7.39 (s).

^{13}C NMR (126 MHz, CD_3CN, 298 K, δ): 194.5, 167.6, 142.8 (t, *J* = 31.5 Hz), 121.4, 106.4, 38.1, 23.4, 23.0.

HRMS-EI (m/z) calc'd for $C_8H_7D_1O_2$ $[M]^+$, 137.0582; found, 137.0584; deviation: –1.9 ppm.

4-[^{2}H]-Acetylmethylalanate ([^{2}H]14)

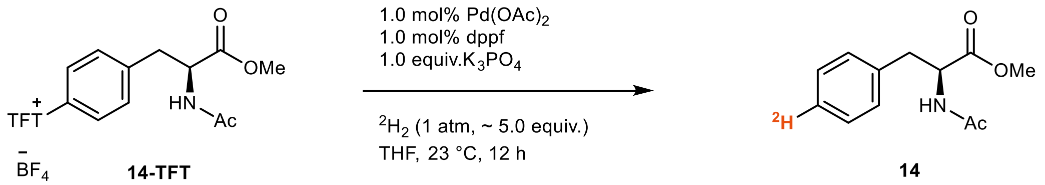

Aryl thianthrenium salt **14-TFT** (59.5 mg, 0.100 mmol, 1.00 equiv.), K_3PO_4 (21.3 mg, 0.100 mmol, 1.00 equiv.), and THF (0.25 mL, c = 0.20 M) were added to a 10-mL Schlenk tube containing a magnetic stir bar. Subsequently, a stock THF solution (0.25 mL) containing $Pd(OAc)_2$ (0.25 mg, 1.0 μmol, 1.0 mol%) and dppf (0.55 mg, 1.0 μmol, 1.0 mol%) was added to the reaction mixture. The Schlenk tube was then connected to a high vacuum line and a balloon

containing $^2H_2$ (1 atm) via a T-bore glass stopcock adaptor (Figure S1). The reaction mixture was degassed via three freeze-pump-thaw cycles. After the third freeze-pump-thaw cycle, $^2H_2$ (1 atm) was introduced to the Schlenk tube while keeping the bottom of the Schlenk tube submersed in a water bath (23 °C). After the Schlenk tube was warmed to 23 °C, the tube was sealed, and the reaction mixture was stirred vigorously at 23 °C. After 12 hours, the reaction vessel was opened to air, and CH_2Cl_2 (5 mL) was added to the reaction mixture. The resulting mixture was concentrated by rotary evaporation. The residue was purified by chromatography on silica gel, eluting with EtOAc/hexanes 1:1 (v/v) to afford 18.0 mg (81% yield) of the title compound as a colorless solid.

Deuterium incorporation: >0.99 D/molecule (^{1}H NMR)

R_f = 0.14 (hexanes/EtOAc, 1:2 (v:v))

NMR Spectroscopy:

^{1}H NMR (500 MHz, CD_2Cl_2, 298 K, δ): 7.30 (d, *J* = 7.6 Hz, 2H), 7.13 – 7.09 (m, 2H), 5.91 (br, 1H, N*H*), 4.81 (dtd, *J* = 7.0, 5.8, 1.0 Hz, 1H), 3.70 (d, *J* = 1.1 Hz, 3H), 3.13 (dd, *J* = 13.9, 5.8 Hz, 1H), 3.05 (dd, *J* = 13.8, 6.0 Hz, 1H), 1.93 (d, *J* = 1.0 Hz, 3H).

^{2}H NMR (92 MHz, CH_2Cl_2, 298 K, δ): 7.30 (s).

^{13}C NMR (126 MHz, CD_2Cl_2, 298 K, δ): 172.4, 169.7, 136.6, 129.7, 128.8, 127.1 (t, *J* = 23.9 Hz), 52.6, 38.2, 23.3.

HRMS-CI (m/z) calc'd for $C_{12}H_{15}D_1N_1O_3$ $[M+H]^+$, 223.1187; found, 223.1187; deviation: +0.3 ppm.

[^{2}H]Indomethacin methylester ([^{2}H]15)

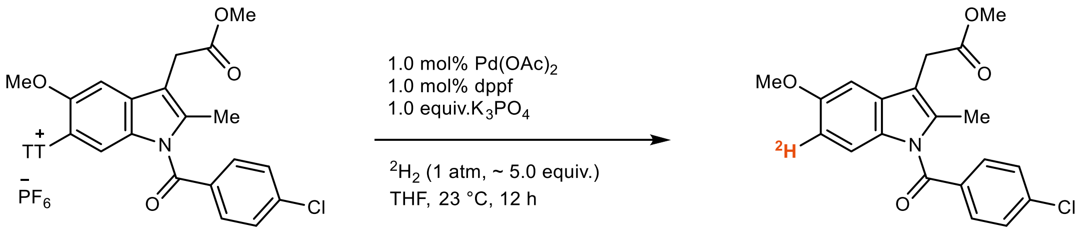

15-TT **[^{2}H]15**

Aryl thianthrenium salt **15-TT** (110 mg, 0.150 mmol, 1.00 equiv.), K_3PO_4 (31.9 mg, 0.150 mmol, 1.00 equiv.) and THF (0.4 mL, c = 0.2 M) were added to a 25-mL Schlenk tube containing a

magnetic stir bar. Subsequently, a stock THF solution (0.4 mL) containing $Pd(OAc)_2$ (0.38 mg, 1.5 µmol, 1.0 mol%) and dppf (0.8 mg, 1.5 µmol, 1.0 mol%) was added to the reaction mixture. The Schlenk tube was then connected to a high vacuum line and a balloon containing $^2H_2$ (1 atm) via a T-bore glass stopcock adaptor (Figure S1). The reaction mixture was degassed via three freeze-pump-thaw cycles. After the third freeze-pump-thaw cycle, $^2H_2$ (1 atm) was introduced to the Schlenk tube while keeping the bottom of the Schlenk tube submersed in a water bath (23 °C). After the Schlenk tube was warmed to 23 °C, the tube was sealed, and the reaction mixture was stirred vigorously at 23 °C. After 12 hours, the reaction vessel was opened to air, and CH_2Cl_2 (5 mL) was added to the reaction mixture. The resulting mixture was concentrated by rotary evaporation. The residue was purified by chromatography on silica gel, eluting with EtOAc/hexanes 1:20 (v/v) to afford 62.6 mg (84% yield) of the title compound as a light yellow oil.

Deuterium incorporation: >0.99 ^{2}H/molecule (^{1}H NMR)

R_f = 0.11 (hexanes/EtOAc, 5:1 (v:v))

NMR Spectroscopy:

^{1}H NMR (500 MHz, CD_3CN, 298 K, δ): 7.63 – 7.60 (m, 2H), 7.53 – 7.49 (m, 2H), 6.98 (s, 1H), 6.94 (s, 1H), 3.78 (s, 3H), 3.69 (s, 2H), 3.65 (s, 3H), 2.24 (s, 3H).

^{2}H NMR (92 MHz, CH_3CN, 298 K, δ): 6.72 (s).

^{13}C NMR (126 MHz, CD_3CN, 298 K, δ): 172.2, 169.2, 157.0, 139.3, 136.8, 135.4, 132.0, 131.8, 131.6, 129.9, 115.8, 113.8, 112.0 (t, J = 24.6 Hz) 102.3, 56.2, 52.5, 30.2, 13.6.

HRMS-ESI POS (m/z) calc'd for $C_{20}H_{17}D_1Cl_1N_1O_4Na_1$ $[M+Na]^+$, 395.0879; found, 395.0882; deviation: –0.8 ppm.

[^{2}H](±)Famoxadone ([^{2}H]16)

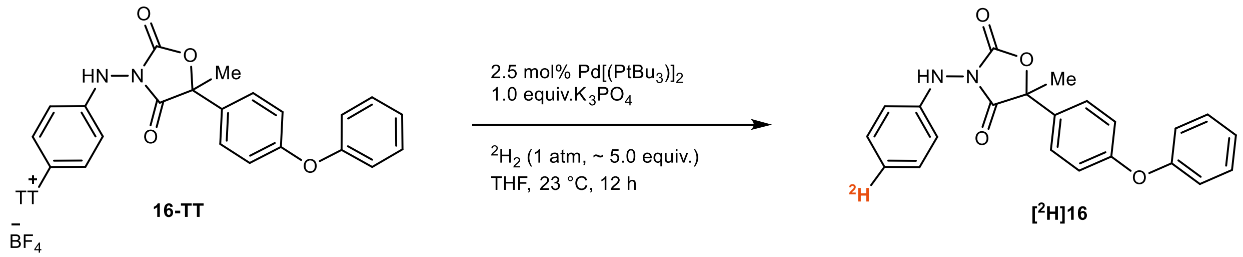

Aryl fluorothianthrenium salt **16-TT** (135 mg, 0.200 mmol, 1.00 equiv.), K_3PO_4 (42.5 mg, 0.200 mmol, 1.00 equiv.), and THF (0.5 mL, c = 0.2 M) were added to a 25-mL Schlenk tube containing a magnetic stir bar. Subsequently, a stock THF solution (0.5 mL) containing Pd[(P*t*Bu$_3$)]$_2$ (2.5 mg, 2.0 µmol, 2.5 mol%) was added to the reaction mixture. The Schlenk tube was then connected to a high vacuum line and a balloon containing $^2H_2$ (1 atm) via a T-bore glass stopcock adaptor (Figure S1). The reaction mixture was degassed via three freeze-pump-thaw cycles. After the third freeze-pump-thaw cycle, $^2H_2$ (1 atm) was introduced to the Schlenk tube while keeping the bottom of the Schlenk tube submersed in a water bath (23 °C). After the Schlenk tube was warmed to 23 °C, the tube was sealed, and the reaction mixture was stirred vigorously at 23 °C. After 12 hours, the reaction vessel was opened to air, and CH_2Cl_2 (5 mL) was added to the reaction mixture. The resulting mixture was concentrated by rotary evaporation. The residue was purified by chromatography on silica gel, eluting with CH_2Cl_2/hexanes 2:1 (v/v) to afford 64.8 mg (87% yield) of the title compound as a colorless solid.

Deuterium incorporation: >0.99 ^{2}H/molecule (^{1}H NMR)

R$_f$ = 0.40 (DCM)

NMR Spectroscopy:

^{1}H NMR (500 MHz, CD_3CN, 298 K, δ): 7.60 – 7.53 (m, 2H), 7.46 – 7.37 (m, 2H), 7.27 – 7.16 (m, 2H), 7.10 – 7.02 (m, 4H), 6.94 (br, 1H, N*H*), 6.81 – 6.70 (m, 2H), 2.00 (s, 3H).

^{2}H NMR (92 MHz, CH_3CN, 298 K, δ): 7.00 (s)

^{13}C NMR (126 MHz, CD_3CN, 298 K, δ): 173.3, 159.4, 157.2, 154.0, 146.1, 131.8, 131.1, 130.3, 127.6, 125.5 (t, *J* = 24.6 Hz),120.4, 119.4, 114.0, 85.7, 24.7.

HRMS-ESI NEG (m/z) calc'd for $C_{22}H_{16}D_1N_2O_4$ [M-H]$^-$, 374.1258; found, 374.1257; deviation: –0.4 ppm.

2-[^{2}H]-3-Methyl-5-acetyl thiophene ([^{2}H]17)

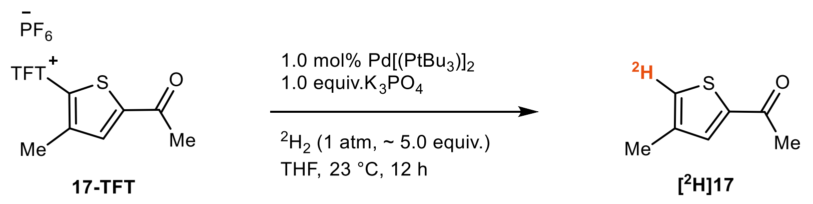

Aryl tetrafluorothianthrenium salt **17-TFT** (115 mg, 0.200 mmol, 1.00 equiv.), K_3PO_4 (42.5 mg, 0.200 mmol, 1.00 equiv.), and THF (0.5 mL, c = 0.2 M) were added to a 25-mL Schlenk tube containing a magnetic stir bar. Subsequently, a stock THF solution (0.5 mL) containing Pd[(P*t*Bu_3)]$_2$ (1.0 mg, 1.0 μmol, 1.0 mol%) was added to the reaction mixture. The Schlenk tube was then connected to a high vacuum line and a balloon containing $^2H_2$ (1 atm) via a T-bore glass stopcock adaptor (Figure S1). The reaction mixture was degassed via three freeze-pump-thaw cycles. After the third freeze-pump-thaw cycle, $^2H_2$ (1 atm) was introduced to the Schlenk tube while keeping the bottom of the Schlenk tube submersed in a water bath (23 °C). After the Schlenk tube was warmed to 23 °C, the tube was sealed, and the reaction mixture was stirred vigorously at 23 °C. After 12 hours, the reaction vessel was opened to air, and CH_2Cl_2 (5 mL) was added to the reaction mixture. The resulting mixture was concentrated by rotary evaporation. The residue was purified by chromatography on silica gel, eluting with EtOAc/hexanes 1:20 (v/v) to afford 22.6 mg (80% yield) of the title compound as a colorless oil.

Deuterium incorporation: >0.99 ^{2}H/molecule (^{1}H NMR)

R_f = 0.43 (hexanes/EtOAc, 5:1 (v:v))

NMR Spectroscopy:

^{1}H NMR (500 MHz, CD_2Cl_2, 298 K, δ): 7.51 (s, 1H), 2.50 (s, 3H), 2.29 (s, 3H).

^{2}H NMR (92 MHz, CH_2Cl_2, 298 K, δ): 7.30 (s).

^{13}C NMR (126 MHz, CD_2Cl_2, 298 K, δ): 190.9, 144.5, 139.3, 134.9, 129.5 (t, J = 28.4 Hz) 27.0, 15.6.

HRMS-EI (m/z) calc'd for $C_7H_7D_1S_1O_1$ $[M]^+$, 141.0353; found, 141.0355; deviation: –1.6 ppm.

1-Phenyl-4-[^{2}H]-1H-pyrazole ([^{2}H]18)

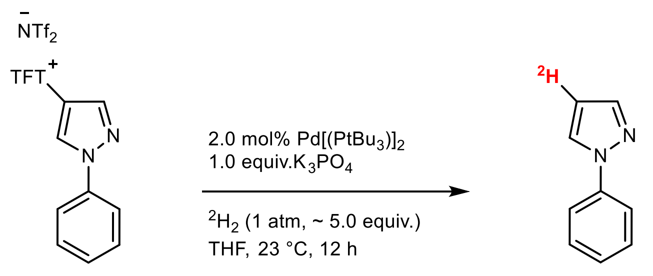

18-TFT **[^{2}H]18**

Aryl tetrafluorothianthrenium salt **18-TFT** (115 mg, 0.200 mmol, 1.00 equiv.), K_3PO_4 (42.5 mg, 0.200 mmol, 1.00 equiv.), and THF (0.5 mL, c = 0.2 M) were added to a 25-mL Schlenk tube containing a magnetic stir bar. Subsequently, a stock THF solution (0.5 mL) containing Pd[(P*t*Bu$_3$)]$_2$ (2.0 mg, 2.0 μmol, 2.0 mol%) was added to the reaction mixture. The Schlenk tube was then connected to a high vacuum line and a balloon containing $^2H_2$ (1 atm) via a T-bore glass stopcock adaptor (Figure S1). The reaction mixture was degassed via three freeze-pump-thaw cycles. After the third freeze-pump-thaw cycle, $^2H_2$ (1 atm) was introduced to the Schlenk tube while keeping the bottom of the Schlenk tube submersed in a water bath (23 °C). After the Schlenk tube was warmed to 23 °C, the tube was sealed, and the reaction mixture was stirred vigorously at 23 °C. After 12 hours, the reaction vessel was opened to air, and CH_2Cl_2 (5 mL) was added to the reaction mixture. The resulting mixture was concentrated by rotary evaporation. The residue was purified by chromatography on silica gel, eluting with EtOAc/hexanes 1:20 (v/v) to afford 23.5 mg (81% yield) of the title compound as a colorless solid.

Deuterium incorporation: >0.99 ^{2}H/molecule (^{1}H NMR)

R$_f$ = 0.47 (hexanes/EtOAc, 5:1 (v:v))

NMR Spectroscopy:

^{1}H NMR (500 MHz, CD_2Cl_2, 298 K, δ): 7.98 (s, 1H), 7.72 (p, *J* = 1.4 Hz, 1H), 7.71 – 7.67 (m, 2H), 7.50 – 7.42 (m, 2H), 7.35 – 7.25 (m, 1H).

^{2}H NMR (92 MHz, CH_2Cl_2, 298 K, δ): 6.53 (s).

^{13}C NMR (101 MHz, CD_2Cl_2, 298 K, δ): 141.2, 140.7, 129.8, 127.0, 126.7, 119.4, 107.7 (t, *J* = 34.0 Hz).

HRMS-ESI POS (m/z) calc'd for $C_9H_8N_2D_1$ $[M+H]^+$, 146.0823; found, 146.0823; deviation: –0.2 ppm.

2-Nitro-4'-[^{2}H]-biphenyl ([^{2}H]19)

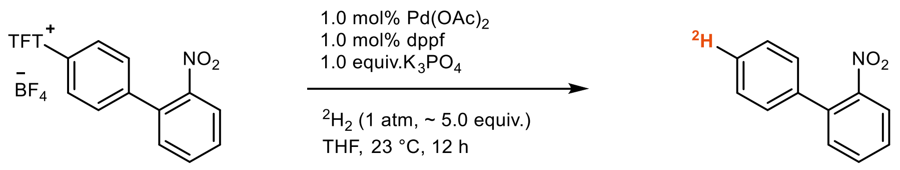

19-TFT **[^{2}H]19**

Aryl tetrafluorothianthrenium salt **19-TFT** (115 mg, 0.200 mmol, 1.00 equiv.), K_3PO_4 (42.5 mg, 0.200 mmol, 1.00 equiv.), and THF (0.5 mL, c = 0.2 M) were added to a 25-mL Schlenk tube containing a magnetic stir bar. Subsequently, a stock THF solution (0.5 mL) containing $Pd(OAc)_2$ (0.5 mg, 2 μmol, 1 mol%) and dppf (1.1 mg, 2.0 μmol, 1.0 mol%) was added to the reaction mixture. The Schlenk tube was then connected to a high vacuum line and a balloon containing $^2H_2$ (1 atm) via a T-bore glass stopcock adaptor (Figure S1). The reaction mixture was degassed via three freeze-pump-thaw cycles. After the third freeze-pump-thaw cycle, $^2H_2$ (1 atm) was introduced to the Schlenk tube while keeping the bottom of the Schlenk tube submersed in a water bath (23 °C). After the Schlenk tube was warmed to 23 °C, the tube was sealed, and the reaction mixture was stirred vigorously at 23 °C. After 12 hours, the reaction vessel was opened to air, and CH_2Cl_2 (5 mL) was added to the reaction mixture. The resulting mixture was concentrated by rotary evaporation. The residue was purified by chromatography on silica gel, eluting with EtOAc/hexanes 1:30 (v/v) to afford 35.1 mg (87% yield) of the title compound as a colorless solid.

Deuterium incorporation: >0.99 ^{2}H/molecule (^{1}H NMR)

R_f = 0.46 (hexanes/EtOAc, 5:1 (v:v))

NMR Spectroscopy:

^{1}H NMR (500 MHz, CD_2Cl_2, 298 K, δ) 7.86 (dd, *J* = 8.1, 1.4 Hz, 1H), 7.65 (td, *J* = 7.6, 1.3 Hz, 1H), 7.51 (td, *J* = 7.8, 1.4 Hz, 1H), 7.49 – 7.41 (m, 3H), 7.33 (d, *J* = 8.2 Hz, 2H).

^{2}H NMR (92 MHz, CH_2Cl_2, 298 K, δ): 7.48 (s).

^{13}C NMR (126 MHz, CD_2Cl_2, 298 K, δ): 149.7, 138.0, 136.6, 132.8, 132.4, 128.9, 128.7, 128.3, 124.4.

HRMS-EI (m/z) calc'd for $C_{12}H_8D_1Cl_1N_1O_2$ $[M]^+$, 200.0691; found, 200.0691; deviation: –0.2 ppm.

[^{2}H]Strychnine ([^{2}H]20)

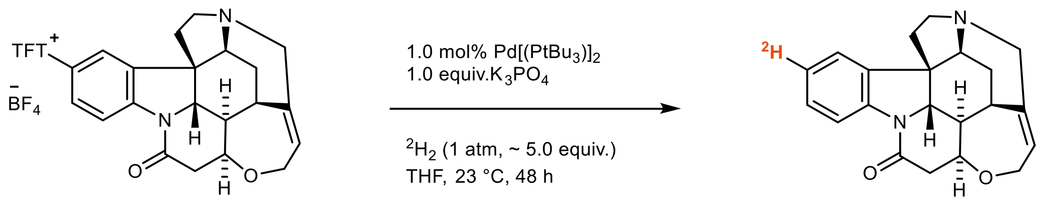

20-TFT **[^{2}H]20**

Aryl tetrafluorothianthrenium salt **20-TFT** (53 mg, 0.075 mmol, 1.0 equiv.), K_3PO_4 (16 mg, 75 µmol, 1.0 equiv.), and THF (1.0 mL, c = 38 mM) were added to a 25-mL Schlenk tube containing a magnetic stir bar. Subsequently, a stock THF solution (1.0 mL) containing Pd[(P*t*Bu$_3$)]$_2$ (0.40 mg, 0.38 µmol, 1.0 mol%) was added to the reaction mixture. The Schlenk tube was then connected to a high vacuum line and a balloon containing $^2H_2$ (1 atm) via a T-bore glass stopcock adaptor (Figure S1). The reaction mixture was degassed via three freeze-pump-thaw cycles. After the third freeze-pump-thaw cycle, $^2H_2$ (1 atm) was introduced to the Schlenk tube while keeping the bottom of the Schlenk tube submersed in a water bath (23 °C). After the Schlenk tube was warmed to 23 °C, the tube was sealed, and the reaction mixture was stirred vigorously at 23 °C. After 48 hours, the reaction vessel was opened to air, and CH_2Cl_2 (5 mL) was added to the reaction mixture. The resulting mixture was concentrated by rotary evaporation. The residue was purified by chromatography on silica gel, eluting with CH_2Cl_2/NEt_3 10:1 (v/v) to afford 14.9 mg (59% yield) of the title compound as a colorless solid.

Deuterium incorporation: 0.90 ^{2}H/molecule (^{1}H NMR); Higher ^{2}H-incorporation can be achieved with shorter reaction time (95% deuterium enrichment, 6 h).

R$_f$ = 0,30 CH_2Cl_2/NEt_3, 9:1 (v:v))

NMR Spectroscopy:

^{1}H NMR (500 MHz, CD_2Cl_2, 298 K, δ): 8.02 (dd, *J* = 7.9, 0.6 Hz, 1H), 7.24 – 7.16 (m, 2H), 7.08 (td, *J* = 7.5, 1.1 Hz, 0.1 H), 5.87 (dq, *J* = 6.4, 4.5, 3.4 Hz, 1H), 4.27 (dt, *J* = 8.5, 3.4 Hz, 1H), 4.13 – 4.03 (m, 2H), 3.90 (dd, *J* = 4.1, 2.2 Hz, 1H), 3.84 (d, *J* = 10.5 Hz, 1H), 3.66 (dq, *J* = 14.8, 1.6 Hz, 1H), 3.13 (td, *J* = 5.6, 4.9, 2.0 Hz, 2H), 3.05 (dd, *J* = 17.3, 8.4 Hz, 1H), 2.87 – 2.80 (m, 1H), 2.69 – 2.59 (m, 2H), 2.34 (dt, *J* = 14.3, 4.3 Hz, 1H), 1.87 – 1.82 (m, 2H), 1.41 (d, *J* = 14.4 Hz, 1H).

^{2}H NMR (92 MHz, CH_2Cl_2, 298 K, δ): 7.13 (s).

^{13}C NMR (151 MHz, CD_2Cl_2, 298 K, δ): 169.6, 142.9, 141.3, 133.7, 128.5, 127.3, 124.3, 122.7, 116.2, 78.0, 65.0, 60.6, 60.5, 53.0, 52.4, 50.6, 48.6, 43.3, 42.97, 32.1, 27.3.

HRMS-EI (m/z) calc'd for $C_{21}H_{21}D_1N_2O_2$ $[M]^+$, 335.1739; found, 335.1745; deviation: –1.8 ppm.

[^{2}H]Salicin pentahydrate ([^{2}H]21)

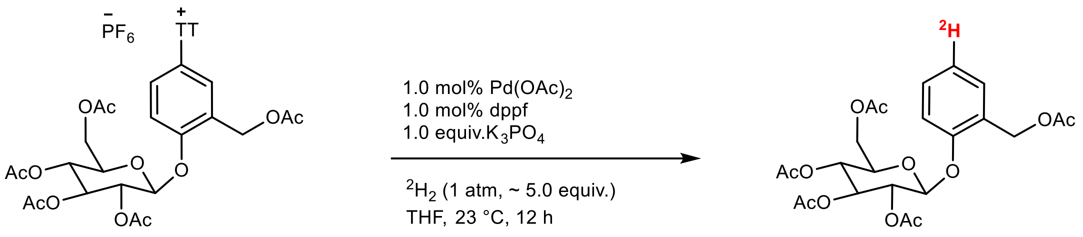

21-TT **[^{2}H]21**

Aryl thianthrenium salt **21-TT** (171 mg, 0.200 mmol, 1.00 equiv.), K_3PO_4 (42.5 mg, 0.200 mmol, 1.00 equiv.), and THF (0.5 mL, c = 0.2 M) were added to a 25-mL Schlenk tube containing a magnetic stir bar. Subsequently, a stock THF solution (0.5 mL) containing $Pd(OAc)_2$ (0.5 mg, 2 µmol, 1 mol%) and dppf (1.1 mg, 2.0 µmol, 1.0 mol%) was added to the reaction mixture. The Schlenk tube was then connected to a high vacuum line and a balloon containing $^2H_2$ (1 atm) via a T-bore glass stopcock adaptor (Figure S1). The reaction mixture was degassed via three freeze-pump-thaw cycles. After the third freeze-pump-thaw cycle, $^2H_2$ (1 atm) was introduced to the Schlenk tube while keeping the bottom of the Schlenk tube submersed in a water bath (23 °C). After the Schlenk tube was warmed to 23 °C, the tube was sealed, and the reaction mixture was stirred vigorously at 23 °C. After 12 hours, the reaction vessel was opened to air, and CH_2Cl_2 (5 mL) was added to the reaction mixture. The resulting mixture was concentrated by rotary evaporation. The residue was purified by chromatography on silica gel, eluting with EtOAc/hexanes 1:1 (v/v) to afford 93.3 mg (94% yield) of the title compound as a colorless solid.

Deuterium incorporation: 0.98 ^{2}H/molecule (^{1}H NMR)

R_f = 0.42 (hexanes/EtOAc, 1:1 (v:v))

NMR Spectroscopy:

^{1}H NMR (500 MHz, CD_3CN, 298 K, δ): 7.38 – 7.31 (m, 2H), 7.15 (d, *J* = 8.3 Hz, 1H), 5.36 (t, *J* = 9.5 Hz, 1H), 5.27 (d, *J* = 7.8 Hz, 1H), 5.22 (dd, *J* = 9.6, 7.9 Hz, 1H), 5.12 (dd, *J* = 10.1, 9.4 Hz, 1H), 5.00 (d, *J* = 3.4 Hz, 2H), 4.25 (dd, *J* = 12.3, 5.6 Hz, 1H), 4.14 (dd, *J* = 12.3, 2.5 Hz, 1H), 4.03 (ddd, *J* = 10.1, 5.6, 2.5 Hz, 1H), 2.05 – 1.98 (m, 15H).

^{2}H NMR (92 MHz, CH_3CN, 298 K, δ): 7.16 (s).

^{13}C NMR (126 MHz, CD_3CN, 298 K, δ): 171.5, 171.2, 170.9, 170.5, 170.4, 155.5, 130.4, 130.3, 126.8, 116.3, 99.5, 73.0, 72.4, 71.7, 69.2, 62.6, 61.7, 21.0, 20.9, 20.8.

HRMS-ESI POS (m/z) calc'd for $C_{23}H_{27}D_1O_{12}Na_1$ $[M]^+$, 520.1536; found, 520.1541; deviation: –1.0 ppm.

2-Cyano-5-[^{2}H]-6-methoxyquinoline ([^{2}H]22)

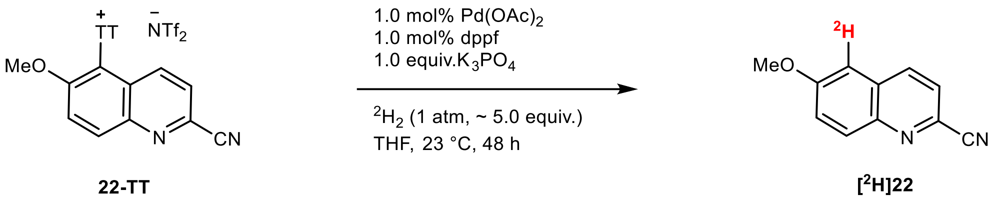

Aryl thianthrenium salt **22-TT** (134 mg, 0.200 mmol, 1.00 equiv.), K_3PO_4 (42.5 mg, 0.200 mmol, 1.00 equiv.), and THF (0.5 mL, c = 0.2 M) were added to a 25-mL Schlenk tube containing a magnetic stir bar. Subsequently, a stock THF solution (0.5 mL) containing $Pd(OAc)_2$ (0.5 mg, 2 μmol, 1 mol%) and dppf (1.1 mg, 2.0 μmol, 1.0 mol%) was added to the reaction mixture. The Schlenk tube was then connected to a high vacuum line and a balloon containing $^2H_2$ (1 atm) via a T-bore glass stopcock adaptor (Figure S1). The reaction mixture was degassed via three freeze-pump-thaw cycles. After the third freeze-pump-thaw cycle, $^2H_2$ (1 atm) was introduced to the Schlenk tube while keeping the bottom of the Schlenk tube submersed in a water bath (23 °C). After the Schlenk tube was warmed to 23 °C, the tube was sealed, and the reaction mixture was stirred vigorously at 23 °C. After 48 hours, the reaction vessel was opened to air, and CH_2Cl_2 (5 mL) was added to the reaction mixture. The resulting mixture was concentrated by rotary evaporation. The residue was purified by chromatography on silica gel, eluting with EtOAc/hexanes 1:5 (v/v) to afford 25.7 mg (70% yield) of the title compound as a colorless solid.

Deuterium incorporation: >0.99 ^{2}H/molecule (^{1}H NMR)

R_f = 0.13 (hexanes/EtOAc, 5:1 (v:v))

NMR Spectroscopy:

^{1}H NMR (500 MHz, CD_2Cl_2, 298 K, δ): 8.18 (dd, J = 8.4, 0.9 Hz, 1H), 8.00 (dd, J = 9.2, 0.8 Hz, 1H), 7.64 (d, J = 8.5 Hz, 1H), 7.46 (d, J = 9.3 Hz, 1H), 3.95 (s, 3H).

^{2}H NMR (92 MHz, CH_2Cl_2, 298 K, δ): 7.19 (s).

^{13}C NMR (126 MHz, CD_2Cl_2, 298 K, δ) 160.3, 144.9, 136.1, 131.6, 131.1, 130.7, 124.8, 124.2, 118.3, 104.9 (t, J = 24.6 Hz), 56.2.

HRMS-EI (m/z) calc'd for $C_{11}H_7D_1O_1N_2$ $[M]^+$, 185.0694; found, 185.0697; deviation: –1.4 ppm.

5-[^{2}H]-2-Methoxybenzaldehyde ([^{2}H]23)

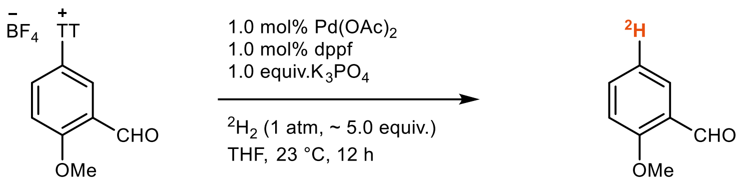

23-TT **[^{2}H]23**

Aryl thianthrenium salt **23-TT** (87.6 mg, 0.200 mmol, 1.00 equiv.), K_3PO_4 (42.5 mg, 0.200 mmol, 1.00 equiv.), and THF (1.0 mL, c = 0.2 M) were added to a 25-mL Schlenk tube containing a magnetic stir bar. Subsequently, a stock THF solution (0.5 mL) containing $Pd(OAc)_2$ (0.5 mg, 2 μmol, 1 mol%) and dppf (1.1 mg, 2.0 μmol, 1.0 mol%) was added to the reaction mixture. The Schlenk tube was then connected to a high vacuum line and a balloon containing $^2H_2$ (1 atm) via a T-bore glass stopcock adaptor (Figure S1). The reaction mixture was degassed via three freeze-pump-thaw cycles. After the third freeze-pump-thaw cycle, $^2H_2$ (1 atm) was introduced to the Schlenk tube while keeping the bottom of the Schlenk tube submersed in a water bath (23 °C). After the Schlenk tube was warmed to 23 °C, the tube was sealed, and the reaction mixture was stirred vigorously at 23 °C. After 12 hours, the reaction vessel was opened to air, and CH_2Cl_2 (5 mL) was added to the reaction mixture. The resulting mixture was concentrated by rotary evaporation. The residue was purified by chromatography on silica gel, eluting with EtOAc/hexanes 1:30 (v/v) to afford 23.5 mg (86% yield) of the title compound as a colorless oil.

Deuterium incorporation: >0.99 ^{2}H/molecule (^{1}H NMR)

R_f = 0.24 (hexanes/EtOAc, 9:1 (v:v))

NMR Spectroscopy:

^{1}H NMR (600 MHz, CD_2Cl_2, 298 K, δ): 10.45 (s, 1H), 7.78 (t, J = 1.0 Hz, 1H), 7.58 (ddt, J = 8.4, 2.0, 1.0 Hz, 1H), 7.03 (d, J = 8.4 Hz, 1H), 3.93 (s, 3H).

^{2}H NMR (92 MHz, CH_2Cl_2, 298 K, δ): 7.07 (s).

^{13}C NMR (126 MHz, CD_2Cl_2, 298 K, δ): 189.8, 162.3, 136.2, 128.4, 120.7 (t, *J* = 23.9 Hz), 112.2, 56.1.

HRMS-ESI POS (m/z) calc'd for $C_8H_8D_1O_2^+$, 138.0660; found, 138.0661; deviation: –1.0 ppm.

2,2,2-Trichloroethyl (4-(4'-[^{2}H]phenyl)butyl)carbamate ([^{2}H]24)

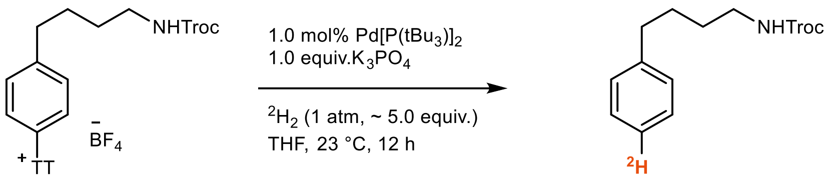

24-TT **[^{2}H]24**

Aryl thianthrenium salt **24-TT** (30.0 mg, 48.0 µmol, 1.00 equiv.) and K_3PO_4 (10.2 mg, 48.0 µmol mmol, 1.00 equiv.) were added to a 25-mL Schlenk tube containing a magnetic stir bar. Subsequently, a stock THF solution (0.5 mL, c = 0.01 M) containing Pd[P(*t*Bu)$_3$] (0.24 mg, 0.48 µmol, 1.0 mol%) was added to the reaction mixture. The Schlenk tube was then connected to a high vacuum line and a balloon containing $^2H_2$ (1 atm) via a T-bore glass stopcock adaptor (Figure S1). The reaction mixture was degassed via three freeze-pump-thaw cycles. After the third freeze-pump-thaw cycle, $^2H_2$ (1 atm) was introduced to the Schlenk tube while keeping the bottom of the Schlenk tube submersed in a water bath (23 °C). After the Schlenk tube was warmed to 23 °C, the tube was sealed, and the reaction mixture was stirred vigorously at 23 °C. After 12 hours, the reaction vessel was opened to air, and CH_2Cl_2 (5 mL) was added to the reaction mixture. The resulting mixture was concentrated by rotary evaporation. The residue was purified by chromatography on silica gel, eluting with EtOAc/hexanes 1:20 (v/v) to afford 13.9 mg (89% yield) of the title compound as a colorless oil.

Deuterium incorporation: >0.99 ^{2}H/molecule (^{1}H NMR)

R$_f$ = 0.19 (hexanes/EtOAc, 9:1 (v:v))

NMR Spectroscopy:

^{1}H NMR (600 MHz, CD_2Cl_2, 298 K, δ): 7.27 (d, *J* = 8.0 Hz, 2H), 7.18 (d, *J* = 8.0 Hz, 2H), 5.05 (s, 1H, N*H*), 4.72 (s, 2H), 3.23 (td, *J* = 7.0, 6.0 Hz, 2H), 2.64 (t, *J* = 7.6 Hz, 2H), 1.68 – 1.63 (m, 2H), 1.59 – 1.55 (m, 2H).

^{2}H NMR (92 MHz, CH_2Cl_2, 298 K, δ): 7.22 (s).

^{13}C NMR (151 MHz, CD_2Cl_2, 298 K, δ): 154.9, 142.7, 128.8, 128.6, 128.6, 125.9 (t, *J* = 24.2 Hz), 96.3, 74.7, 41.5, 35.8, 29.8, 28.9.

HRMS-ESI POS (m/z) calc'd for $C_{13}H_{15}D_1Cl_3Na_1O_2^+$ $[M+Na]^+$, 347.0206; found, 347.0202; deviation: –1.3 ppm.

[^{2}H]Boscalid ([^{2}H]25)

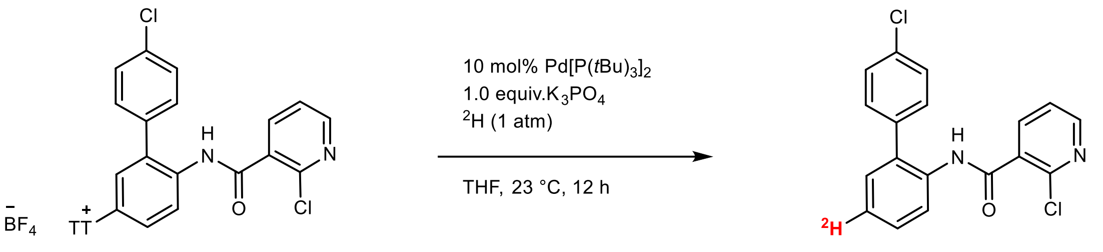

Aryl thianthrenium salt **25-TT** (32 mg, 0.050 mmol, 1.0 equiv.), K_3PO_4 (11 mg, 0.050 mmol, 1.0 equiv.), and THF (1.0 mL, c = 25 mM) were added to a 25-mL Schlenk tube containing a magnetic stir bar. Subsequently, a stock THF solution (1.0 mL) containing Pd[(P*t*Bu$_3$)]$_2$ (0.25 mg, 5.0 µmol, 10 mol%) was added to the reaction mixture. The Schlenk tube was then connected to a high vacuum line and a balloon containing $^2H_2$ (1 atm) via a T-bore glass stopcock adaptor (Figure S1). The reaction mixture was degassed via three freeze-pump-thaw cycles. After the third freeze-pump-thaw cycle, $^2H_2$ (1 atm) was introduced to the Schlenk tube while keeping the bottom of the Schlenk tube submersed in a water bath (23 °C). After the Schlenk tube was warmed to 23 °C, the tube was sealed, and the reaction mixture was stirred vigorously at 23 °C. After 48 hours, the reaction vessel was opened to air, and CH_2Cl_2 (5 mL) was added to the reaction mixture. The resulting mixture was concentrated by rotary evaporation. The residue was purified by chromatography on silica gel, eluting with EtOAc/pentane 1:3 (v/v) to afford 17.3 mg (99% yield) of the title compound as a colorless solid.

Deuterium incorporation: 1.01 ± 0.01 ^{2}H/molecule (mass analysis); >0.99 ^{2}H/molecule (^{1}H NMR).

R$_f$ = 0.35 (pentane/EtOAc, 5:1 (v:v))

NMR Spectroscopy:

^{1}H NMR (500 MHz, CD_2Cl_2, 298 K, δ): 8.42 (dd, J = 4.7, 2.0 Hz, 1H), 8.33 (d, J = 8.2 Hz, 1H), 8.04 (dd, J = 7.6, 2.0 Hz, 1H), 8.00 (br, 1H, N*H*), 7.48 – 7.41 (m, 3H), 7.39 – 7.33 (m, 3H), 7.30 (d, J = 1.6 Hz, 1H).

^{2}H NMR (92 MHz, CH_2Cl_2, 298 K, δ): 7.33 (s).

^{13}C NMR (126 MHz, CD_2Cl_2, 298 K, δ): 163.1, 151.6, 147.1, 139.9, 136.9, 134.8, 134.6, 133.0, 131.8, 131.3, 130.6, 129.6, 125.5 (t, J = 23.9 Hz), 123.3, 122.8.

HRMS-EI (m/z) calc'd for $C_{18}H_{11}D_1N_2O_1Cl_2$ $[M]^+$, 343.0384; found, 343.0384; deviation: –0.1 ppm.

[^{2}H]LHVS ([^{2}H]26)

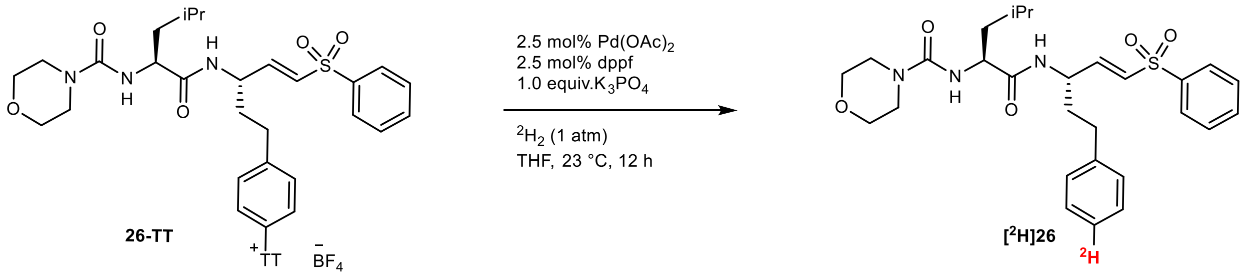

Aryl thianthrenium salt **26-TT** (23 mg, 0.028 mmol, 1.0 equiv.), K_3PO_4 (5.9 mg, 0.028 mmol, 1.0 equiv.), and THF (0.25 mL, c = 55 mM) were added to a 25-mL Schlenk tube containing a magnetic stir bar. Subsequently, a stock THF solution (0.25 mL) containing $Pd(OAc)_2$ (0.17 mg, 0.69 μmol, 2.5 mol%) and dppf (3.8 mg, 0.69 μmol, 2.5 mol%) was added to the reaction mixture. The Schlenk tube was then connected to a high vacuum line and a balloon containing $^2H_2$ (1 atm) via a T-bore glass stopcock adaptor (Figure S1). The reaction mixture was degassed via three freeze-pump-thaw cycles. After the third freeze-pump-thaw cycle, $^2H_2$ (1 atm) was introduced to the Schlenk tube while keeping the bottom of the Schlenk tube submersed in a water bath (23 °C). After the Schlenk tube was warmed to 23 °C, the tube was sealed, and the reaction mixture was stirred vigorously at 23 °C. After 48 hours, the reaction vessel was opened to air, and CH_2Cl_2 (5 mL) was added to the reaction mixture. The resulting mixture was concentrated by rotary evaporation. The residue was purified by chromatography on silica gel, eluting with CH_2Cl_2/MeOH 20:1 (v/v) to afford 14.1 mg (96% yield) of the title compound as a colorless solid.

Deuterium incorporation: >0.99 ^{2}H/molecule (^{1}H NMR)

R_f = 0.60 (CH_2Cl_2/MeOH, 10:1 (v:v))

NMR Spectroscopy:

^{1}H NMR (600 MHz, CD_2Cl_2, 298 K, δ): 7.96 (dt, J = 8.5, 1.1 Hz, 2H), 7.78 – 7.72 (m, 1H), 7.67 (t, J = 7.9 Hz, 2H), 7.37 (d, J = 7.7 Hz, 2H), 7.23 (d, J = 7.9 Hz, 2H), 6.98 (dd, J = 15.7, 4.9 Hz),

6.93 (d, J = 8.6 Hz, 1H), 6.61 – 6.56 (m, 1H), 4.74 – 4.62 (m, 1H), 4.42 – 4.29 (m, 1H), 3.81 – 3.60 (m, 4H), 3.49 – 3.30 (m, 4H), 2.80 – 2.66 (m, 2H), 2.11 – 2.02 (m, 1H), 1.95 (ddt, J = 13.8, 8.9, 4.9 Hz, 1H), 1.76 – 1.66 (m, 2H), 1.65 – 1.56 (m, 1H), 1.03 (d, J = 6.4 Hz, 3H), 0.99 (d, J = 6.3 Hz, 3H).

^{2}H NMR (92 MHz, CH_2Cl_2, 298 K, δ): 7.25 (s).

^{13}C NMR (151 MHz, CD_2Cl_2, 298 K, δ): 172.7, 157.6, 145.9, 140.8, 140.4, 133.5, 130.7, 129.3, 128.4, 127.5, 66.3, 53.1, 49.1, 44.1, 40.3, 35.5, 31.8, 29.7, 24.9, 22.7, 22.3, 21.8.

HRMS-ESI POS (m/z) calc'd for $C_{28}H_{36}D_1S_1N_3O_5Na_1$ $[M+Na]^+$, 551.2409; found, 551.2410; deviation: –0.2 ppm.

[^{2}H]Styrene ([^{2}H]27)

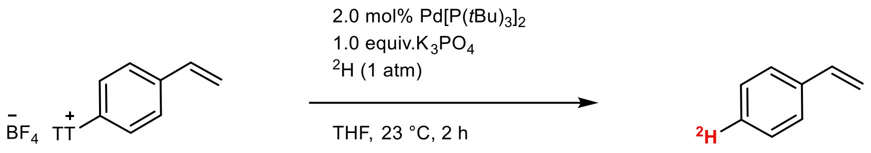

27-TT [^{2}H]27

Aryl thianthrenium salt **27-TT** (20 mg, 0.050 mmol, 1.0 equiv.) and K_3PO_4 (11 mg, 0.050 mmol, 1.0 equiv.) were added to a 10-mL Schlenk tube containing a magnetic stir bar. Subsequently, a stock THF solution (1.0 mL) containing Pd[(PtBu$_3$)]$_2$ (50 µg, 0.50 µmol, 2.0 mol%) was added to the reaction mixture. The Schlenk tube was then connected to a high vacuum line and a balloon containing $^2H_2$ (1 atm) via a T-bore glass stopcock adaptor (Figure S1). The reaction mixture was degassed via three freeze-pump-thaw cycles. After the third freeze-pump-thaw cycle, $^2H_2$ (1 atm) was introduced to the Schlenk tube while keeping the bottom of the Schlenk tube submersed in a water bath (23 °C). After the Schlenk tube was warmed to 23 °C, the tube was sealed, and the reaction mixture was stirred vigorously at 23 °C. After 2 hours, the NMR tube was opened to air and internal standard mesitylene (12 mg, 14 µL, 0.10 mmol, 2.0 equiv.) was added to the reaction mixture. The resulting mixture was diluted with CD_2Cl_2, and the ^{1}H NMR of the crude product mixture was recorded. The yield was determined by ^{1}H NMR by integrating the resonance at 7.31 ppm of the product and comparison to the internal standard mesitylene with a resonance at 6.78 ppm. The deuterium incorporation was determined by mass analysis (94.4 ± 4.3%). The identity of the product was further confirmed by HRMS analysis.

Note: Longer reaction time leads to the over-reduction of the product.

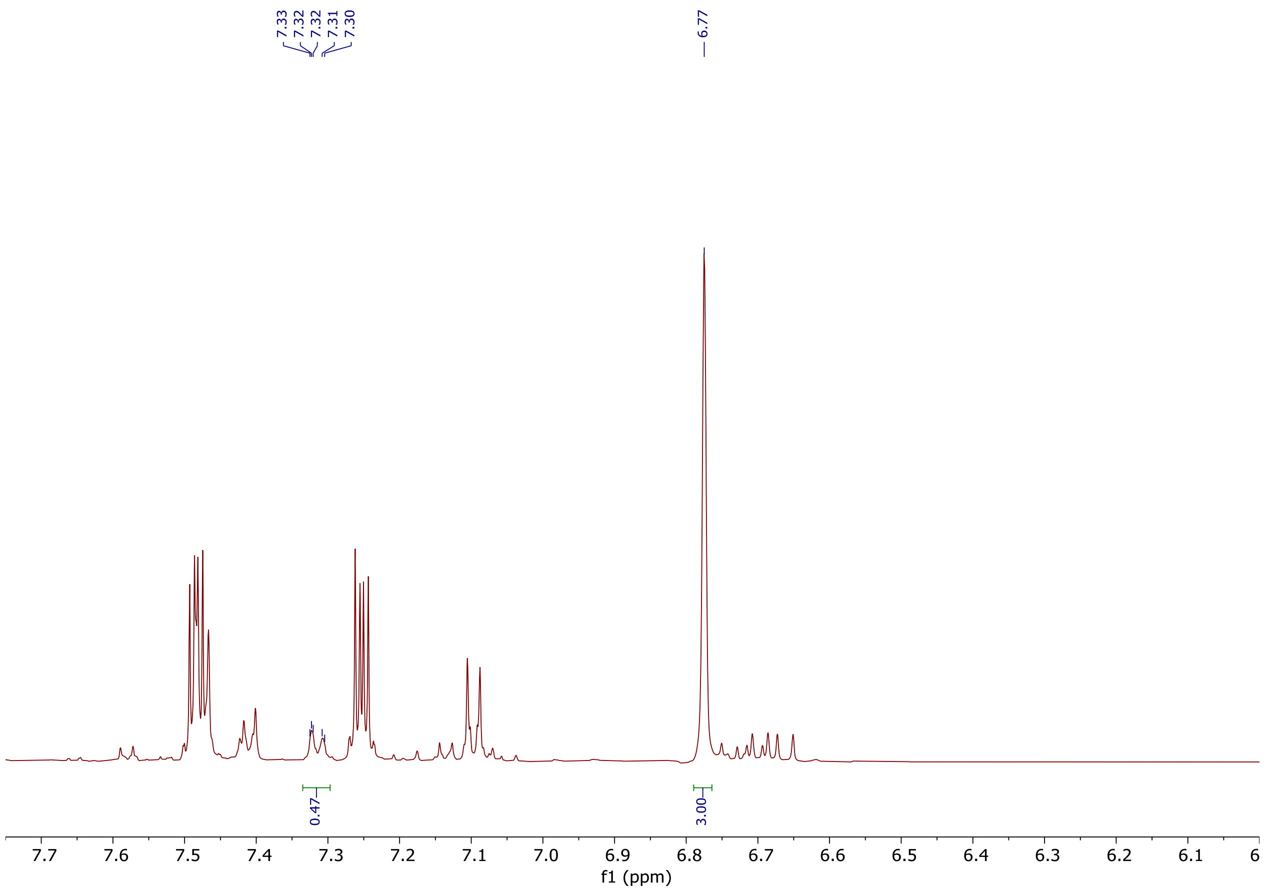

Figure S28. ^{1}H NMR spectrum of the reaction mixture of styrene-derived thianthrenium salt with mesitylene as internal standard.

Deuterium incorporation: 94.4 ± 4.3% ^{2}H/molecule (mass analysis).

HRMS-EI (m/z) calc'd for $C_8H_7D_1$ [M]$^+$, 105.0683; found, 105.0686; deviation: –2.4 ppm.

[^{2}H]Fenofibrate ([^{2}H]28)

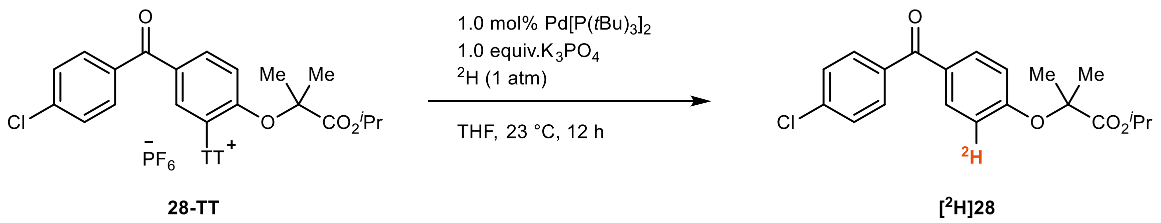

Aryl thianthrenium salt **28-TT** (74 mg, 0.10 mmol, 1.0 equiv.), K_3PO_4 (21 mg, 0.10 mmol, 1.0 equiv.), and THF (1.0 mL, c = 0.10 M) were added to a 25-mL Schlenk tube containing a

magnetic stir bar. Subsequently, a stock THF solution (1.0 mL) containing Pd[(P*t*Bu$_3$)]$_2$ (0.51 mg, 1.0 μmol, 1.0 mol%) was added to the reaction mixture. The Schlenk tube was then connected to a high vacuum line and a balloon containing $^2H_2$ (1 atm) via a T-bore glass stopcock adaptor (Figure S1). The reaction mixture was degassed via three freeze-pump-thaw cycles. After the third freeze-pump-thaw cycle, $^2H_2$ (1 atm) was introduced to the Schlenk tube while keeping the bottom of the Schlenk tube submersed in a water bath (23 °C). After the Schlenk tube was warmed to 23 °C, the tube was sealed, and the reaction mixture was stirred vigorously at 23 °C. After 12 hours, the reaction vessel was opened to air, and CH_2Cl_2 (5 mL) was added to the reaction mixture. The resulting mixture was concentrated by rotary evaporation. The residue was purified by chromatography on silica gel, eluting with EtOAc/pentane 1:20 (v/v) to afford 32.0 mg (89% yield) of the title compound as a colorless solid.

Deuterium incorporation: 1.02 ± 0.02 ^{2}H/molecule (mass analysis); >0.99 ^{2}H/molecule (^{1}H NMR).

R$_f$ = 0.38 (pentane/EtOAc, 9:1 (v:v))

NMR Spectroscopy:

^{1}H NMR (600 MHz, $CDCl_3$, 298 K, δ): 7.77 – 7.68 (m, 4H), 7.50 – 7.42 (m, 2H), 6.87 (d, J = 9.3 Hz, 1H), 5.09 (h, *J* = 6.3 Hz, 1H), 1.66 (s, 6H), 1.20 (d, *J* = 6.3 Hz, 6H).

^{2}H NMR (92 MHz, $CHCl_3$, 298 K, δ): 9.23 (s).

^{13}C NMR (151 MHz, $CDCl_3$, 298 K, δ): 194.4, 173.3, 159.9, 138.5, 136.6, 132.1, 132.0, 131.3, 130.4, 128.7, 117.4, 117.1 (t, *J* = 24.2 Hz), 79.6, 69.5, 25.5, 21.7.

HRMS-EI (m/z) calc'd for $C_{20}H_{20}D_1O_4Cl_1$ [M]$^+$, 361.1186; found, 361.1185; deviation: 0.2 ppm.

[^{2}H]Etofenprox ([^{2}H]29)

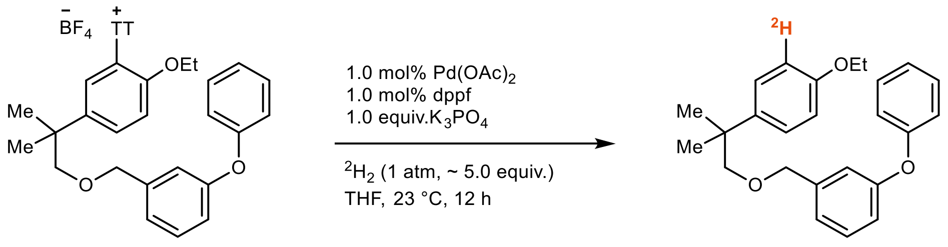

29-TT **[^{2}H]29**

Aryl thianthrenium salt **29-TT** (147 mg, 0.200 mmol, 1.00 equiv.), K_3PO_4 (42.5 mg, 0.200 mmol, 1.00 equiv.), and THF (0.5 mL, c = 0.2 M) were added to a 25-mL Schlenk tube containing a magnetic stir bar. Subsequently, a stock THF solution (0.5 mL) containing $Pd(OAc)_2$ (0.5 mg, 2 μmol, 1 mol%) and dppf (1.1 mg, 2.0 μmol, 1.0 mol%) was added to the reaction mixture. The Schlenk tube was then connected to a high vacuum line and a balloon containing $^2H_2$ (1 atm) via a T-bore glass stopcock adaptor (Figure S1). The reaction mixture was degassed via three freeze-pump-thaw cycles. After the third freeze-pump-thaw cycle, $^2H_2$ (1 atm) was introduced to the Schlenk tube while keeping the bottom of the Schlenk tube submersed in a water bath (23 °C). After the Schlenk tube was warmed to 23 °C, the tube was sealed, and the reaction mixture was stirred vigorously at 23 °C. After 12 hours, the reaction vessel was opened to air, and CH_2Cl_2 (5 mL) was added to the reaction mixture. The resulting mixture was concentrated by rotary evaporation. The residue was purified by chromatography on silica gel, eluting with EtOAc/hexanes 1:30 (v/v) to afford 69.7 mg (93% yield) of the title compound as a colorless oil.

Deuterium incorporation: >0.99 ^{2}H/molecule (^{1}H NMR)

R_f = 0.58 (hexanes/EtOAc, 5:1 (v:v))

NMR Spectroscopy:

^{1}H NMR (500 MHz, CD_3CN, 298 K, δ): 7.39 – 7.34 (m, 2H), 7.31 – 7.24 (m, 3H), 7.14 (tt, *J* = 7.3, 1.1 Hz, 1H), 7.02 – 6.97 (m, 3H), 6.89 (ddt, *J* = 4.7, 2.5, 0.9 Hz, 2H), 6.79 (d, *J* = 9.4 Hz, 1H), 4.40 (s, 2H), 3.98 (q, *J* = 7.0 Hz, 2H), 3.43 (s, 2H), 1.33 (t, *J* = 7.0 Hz, 3H), 1.26 (s, 6H).

^{2}H NMR (92 MHz, CH_3CN, 298 K, δ): 6.84 (s).

^{13}C NMR (126 MHz, CD_3CN, 298 K, δ): 158.3, 158.1, 157.9, 142.3, 140.5, 130.8, 130.7, 128.1, 128.0, 123.1, 119.8, 118.5, 114.8, 114.5 (t, *J* = 23.9 Hz), 81.0, 73.1, 64.1, 39.1, 26.5, 15.1.

HRMS-ESI POS (m/z) calc'd for $C_{25}H_{27}D_1O_3Na_1$ $[M+Na]^+$, 400.1993; found, 400.1999; deviation: –1.3 ppm.

[$^2H_2$]Etofenprox ([$^2H_2$]29)

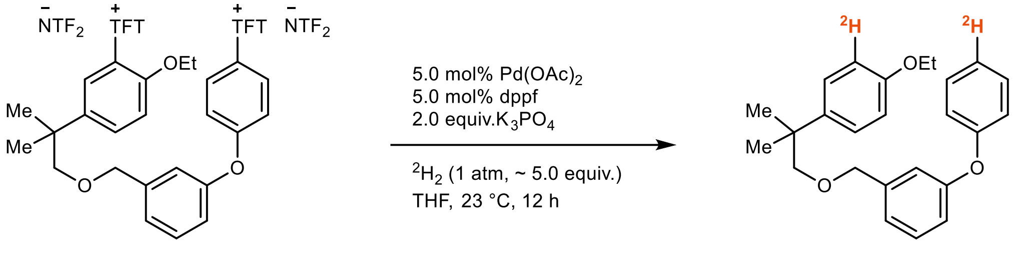

29-TFT$_2$ [$^2H_2$]29

Aryl tetrafluorothianthrenium salt **23-TFT$_2$** (75.6 mg, 50.0 μmol, 1.00 equiv.), K_3PO_4 (21.3 mg, 100 μmol, 2.00 equiv.), and THF (0.25 mL, c = 0.10 M) were added to a 25-mL Schlenk tube containing a magnetic stir bar. Subsequently, a stock THF solution (0.25 mL) containing Pd(OAc)$_2$ (0.60 mg, 2.5 μmol, 5.0 mol%) and dppf (1.4 mg, 2.5 μmol, 1.0 mol%) was added to the reaction mixture. The Schlenk tube was then connected to a high vacuum line and a balloon containing $^2H_2$ (1 atm) via a T-bore glass stopcock adaptor (Figure S1). The reaction mixture was degassed via three freeze-pump-thaw cycles. After the third freeze-pump-thaw cycle, $^2H_2$ (1 atm) was introduced to the Schlenk tube while keeping the bottom of the Schlenk tube submersed in a water bath (23 °C). After the Schlenk tube was warmed to 23 °C, the tube was sealed, and the reaction mixture was stirred vigorously at 23 °C. After 12 hours, the reaction vessel was opened to air, and CH_2Cl_2 (5 mL) was added to the reaction mixture. The resulting mixture was concentrated by rotary evaporation. The residue was purified by chromatography on silica gel, eluting with EtOAc/hexanes 1:30 (v/v) to afford 14.3 mg (83% yield) of the title compound as a colorless oil.

Deuterium incorporation: >1.98 ^{2}H/molecule (^{1}H NMR).

R_f = 0.58 (hexanes/EtOAc, 5:1 (v:v))

NMR Spectroscopy:

^{1}H NMR (500 MHz, CD_3CN, 298 K, δ): 7.38 (dq, J = 7.7, 1.0 Hz, 2H), 7.30 (t, J = 7.8 Hz, 1H), 7.26 (ddd, J = 5.6, 2.8, 1.6 Hz, 2H), 7.02 – 6.97 (m, 3H), 6.91 – 6.86 (m, 2H), 6.80 – 6.77 (m, 1H), 4.41 (s, 2H), 3.99 (q, J = 7.0 Hz, 2H), 3.43 (d, J = 1.2 Hz, 2H), 1.35 – 1.31 (m, 3H), 1.25 (s, 6H).

^{2}H NMR (92 MHz, CH_3CN, 298 K, δ): 7.20 (s), 6.84 (s).

^{13}C NMR (126 MHz, CD_3CN, 298 K, δ): 158.3, 158.1, 157.9, 142.3, 140.5, 130.8, 130.7, 128.1, 128.0, 124.2 (t, J = 24.6 Hz), 123.1, 119.8, 118.5, 114.8, 114.4 (t, J = 23.9 Hz), 81.0, 73.1, 64.1, 39.1, 26.5, 15.1.

HRMS-ESI POS (m/z) calc'd for $C_{25}H_{26}D_2O_3Na_1$ $[M+Na]^+$, 401.2056; found, 401.2057; deviation: –0.1 ppm.

[^{2}H]Benazepril methylester triflimide adduct ([^{2}H]30)

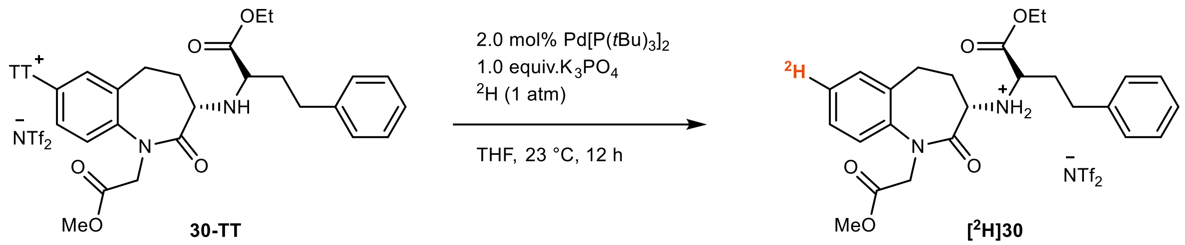

Aryl thianthrenium salt **30-TT** (24 mg, 0.025 mmol, 1.0 equiv.) and K_3PO_4 (6.0 mg, 0.025 mmol, 1.0 equiv.) were added to a 10-mL Schlenk tube containing a magnetic stir bar. Subsequently, a stock THF solution (1.0 mL, c = 50 mM) containing Pd[(P*t*Bu$_3$)]$_2$ (0.25 mg, 1.0 μmol, 2.0 mol%) was added to the reaction mixture. The Schlenk tube was then connected to a high vacuum line and a balloon containing $^2H_2$ (1 atm) via a T-bore glass stopcock adaptor (Figure S1). The reaction mixture was degassed via three freeze-pump-thaw cycles. After the third freeze-pump-thaw cycle, $^2H_2$ (1 atm) was introduced to the Schlenk tube while keeping the bottom of the Schlenk tube submersed in a water bath (23 °C). After the Schlenk tube was warmed to 23 °C, the tube was sealed, and the reaction mixture was stirred vigorously at 23 °C. After 12 hours, the reaction vessel was opened to air, and CH_2Cl_2 (5 mL) was added to the reaction mixture. The resulting mixture was concentrated by rotary evaporation. The residue was purified by chromatography on silica gel, eluting with EtOAc/hexanes 1:10 (v/v) to afford 14.6 mg (81% yield) of the title compound as a colorless solid.

Deuterium incorporation: >0.99 ^{2}H/molecule (^{1}H NMR)

R$_f$ = 0.15 (hexanes/EtOAc, 4:1 (v:v))

NMR Spectroscopy:

^{1}H NMR (600 MHz, CD_3CN, 298 K, δ): 7.32 – 7.29 (m, 1H), 7.26 (ddt, J = 8.9, 5.5, 1.8 Hz, 3H), 7.22 – 7.15 (m, 4H), 4.56 (d, J = 17.4 Hz, 1H), 4.46 (d, J = 17.3 Hz, 1H), 4.03 – 3.93 (m, 2H),

3.67 (s, 3H), 3.25 (dd, J = 11.4, 7.7 Hz, 1H), 3.14 (q, J = 8.2, 7.3 Hz, 2H), 2.62 (d, J = 7.4 Hz, 3H), 1.93 – 1.77 (m, 5H), 1.08 (s, 3H).

^{2}H NMR (92 MHz, CH_3CN, 298 K, δ): 7.29 (s).

^{13}C NMR (151 MHz, CD_3CN, 298 K, δ): 174.5, 174.4, 170.7, 142.7, 130.2, 130.1, 129.4, 129.4, 129.3, 129.2, 128.6, 128.5, 126.8, 123.5, 121.9, 119.5, 61.3, 60.3, 57.4, 52.7, 51.0, 38.2, 35.6, 32.4, 28.8, 14.4.

^{19}F NMR (565 MHz, CD_3CN, 298 K, δ): –80.3 (s).

HRMS-EI (m/z) calc'd for $C_{25}H_{30}D_1O_5N_2$ $[M]^+$, 440.2290; found, 440.2296; deviation: –1.2 ppm.

Reductive tritiation of aryl (tetrafluoro)thianthrenium salts

General information

Tritium gas was obtained from RC Tritec AG (Teufen, Switzerland), and all handling of tritium gas was performed with a manifold from RC Tritec. The radioactivity was measured by liquid scintillation counting using a HIDEX 300 SL and ULTIMATE GOLD™ cocktail (PerkinElmer Inc., Waltham, MA, USA). Radio-HPLC and HPLC-UV comparison was conducted with an Agilent 1200 series HPLC connected in series to an Elysia-Raytest Ramona Star with liquid scintillation. LC-MS analysis was conducted with an Agilent 1260 Infinity LC connected to an Agilent 6125C quadrupole MS.

Reagents for radiochemical experiments: potassium phosphate tribasic (≥ 98%, reagent grade), palladium(II)acetate (98%, reagent grade) and 1,1'bis(diphenylphosphino)ferrocene (97%) were purchased from SigmaAldrich®. Tetrahydrofuran (99.5%, extra dry over molecular sieves) was purchased from Acros, methanol and ethanol (absolute for analysis) from Merck Supelco. All solvents and reagents were used as received. Water was obtained from a Millipore Milli-Q Integral Water Purification System.

HPLC Method: Sunfire C18, 3.5 µm, 4.6 × 150 mm, 1.0 ml/min, 45°C, 230 nm, mobile phases: 0.5% TFA in water (A), acetonitrile (B), water + 5% acetonitrile (C); conditions: 0 ‑ 12 mins (A: 10%, B: 10% to 90%, C: 80% to 0%), then linear to 16 min, 16.0 – 16.1 min (A: 10%, B: 90% to 10%, C: 0% to 80%) then linear till 20 min.

LC-MS Analysis: Agilent Zorbax Eclipse Plus C18 1.8 µm, 2.1 x 50 mm, 0.6 ml/min DAD (190-400 nm), 50°C, mobile phases: water + 0.1% formic acid (A), acetonitrile + 0.1% formic acid (B); conditions: 0 – 0.2 min A: 95% 0.2 – 4 min A: 95 to 5%.

All ^{3}H-labeled molecules were characterized by comparing the HPLC radio-trace of the isolated compound to the HPLC UV-trace of an authentic reference sample.

The **radiochemical purity** was determined by dividing the integrated area under the tritium-labeled arene peak on radio-HPLC chromatogram by the total integrated area of all tritium-containing species peaks on the radio-HPLC chromatogram.

[^{3}H]Nefiracetam ([^{3}H]10)

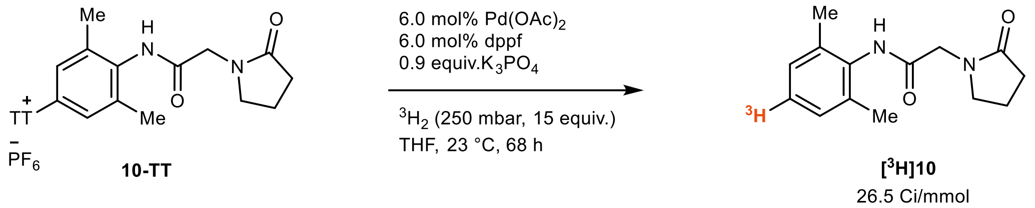

Nefiracetam-derived thianthrenium salt **10-TT** (2.9 mg, 4.7 µmol, 1.0 equiv.) and K_3PO_4 (0.9 mg, 4 µmol, 0.9 equiv.) were added to a 2-mL tritiation flask containing a magnetic stir bar. Subsequently, a THF stock-solution (45 µL) containing $Pd(OAc)_2$ (68 µg, 0.30 µmol, 6.0 mol%), a THF stock-solution (80 µL) containing dppf (160 µg, 0.280 µmol, 6.00 mol%), and THF (375 µL) were added to the reaction mixture. The flask was attached to the tritium manifold and degassed by 3 freeze-thaw cycles. Tritium gas was introduced and the reaction mixture was stirred for 68 h at 23 °C under an atmosphere of tritium at 250 mbar (15 equiv.). The solution was cooled by liquid nitrogen, and the excess tritium gas in the reaction vessel was reabsorbed on an uranium-trap for waste-tritium. The solvent was lyophilized off and labile tritium was removed by lyophilization with methanol (3 × 0.3 mL). The remaining solid was dissolved in 5 mL of ethanol. Solid-phase extraction with a Phenomenex StrataX cartridge (33 µm polymeric reversed phase, 100 mg, 3 mL, 8B-S100EB) and elution with ethanol (approx. 6 mL) provided the desired product as ethanolic solution. 4.6 GBq (125 mCi) (99% based on the thianthrenium starting material) were obtained with a radiochemical purity of 99% (determined by radio-HPLC analysis). A molar activity of 981 GBq/mmol (26.5 Ci/mmol) was achieved (determined by LC-MS analysis).

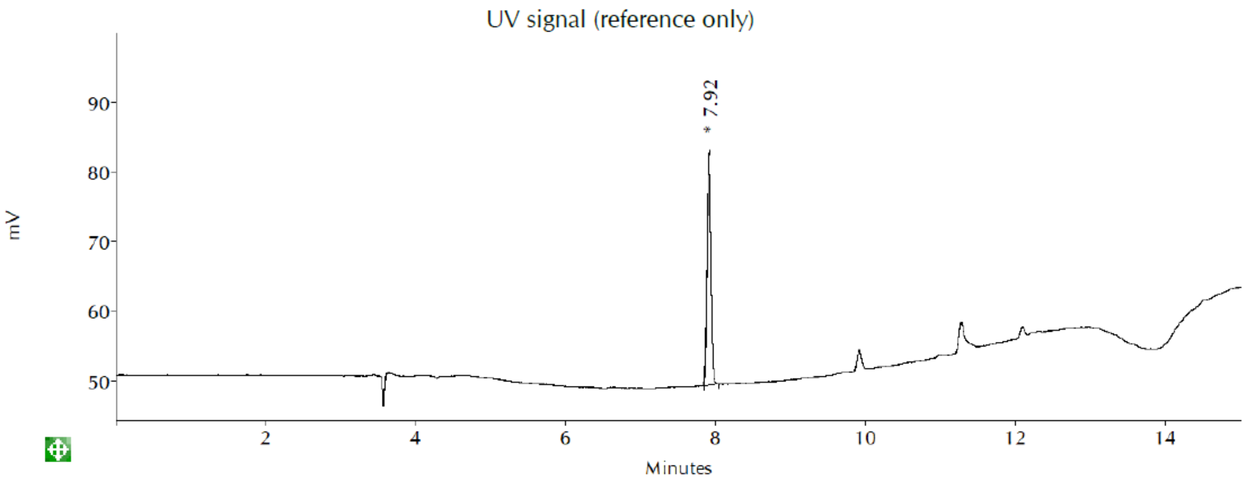

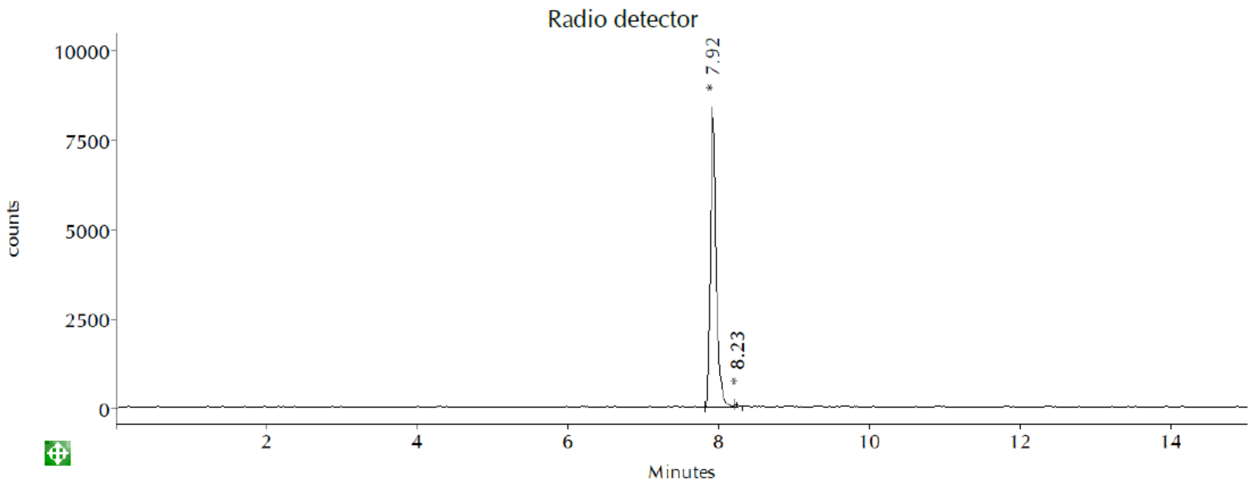

Figure S29. HPLC UV-trace of nefiracetam as the reference (top) and radio-HPLC trace of the tritiation reaction of nefiracetam-derived thianthrenium salt **10-TT** (bottom).

[^{3}H](±)-Pyriproxyfen ([^{3}H]11)

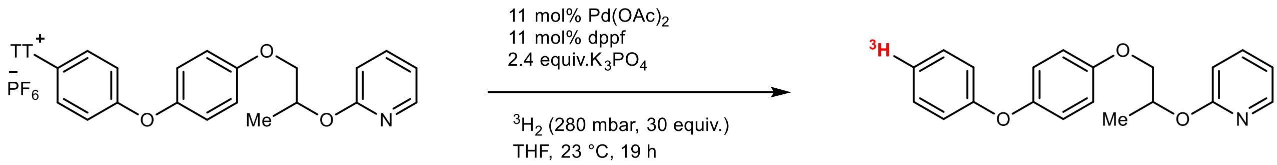

11-TT

[^{3}H]11
26.6 Ci/mmol

Pyriproxyfen-derived thianthrenium salt **11-TT** (1.5 mg, 2.5 µmol, 1.0 equiv.) and K_3PO_4 (1.3 mg, 6.0 µmol, 2.4 equiv.) were added to a 2-mL tritiation flask containing a magnetic stir bar. Subsequently, a THF stock-solution (0.05 mL) containing $Pd(OAc)_2$ (65 µg, 0.29 µmol, 11 mol%), a THF stock-solution (120 µL) containing dppf (160 µg, 0.280 µmol, 11.0 mol%), and THF (330 µL) were added to the reaction mixture. The flask was attached to the tritium manifold and degassed by 3 freeze-thaw cycles. Tritium gas was introduced and the reaction mixture was stirred for 19 h at 23 °C under an atmosphere of tritium at 280 mbar (30 equiv.). The solution was cooled by liquid nitrogen and the excess tritium gas in the reaction vessel was reabsorbed on an uranium-trap for waste-tritium. The solvent was lyophilized off and labile tritium was removed by lyophilization with methanol (3 × 1 mL). The remaining solid was dissolved in water (0.1 mL), transferred onto a solid phase extraction cartridge (Phenomenex StrataX, 33 µm polymeric reversed phase, 100 mg, 3 mL, 8B-S100EB) and eluted with ethanol (5 mL). 2.0 GBq (55 mCi) (83% based on the thianthrenium starting material) were obtained with a radiochemical purity of 99% (determined by radio-HPLC analysis). A molar activity of 984 GBq/mmol (26.6 Ci/mmol) was achieved (determined by LC-MS analysis).

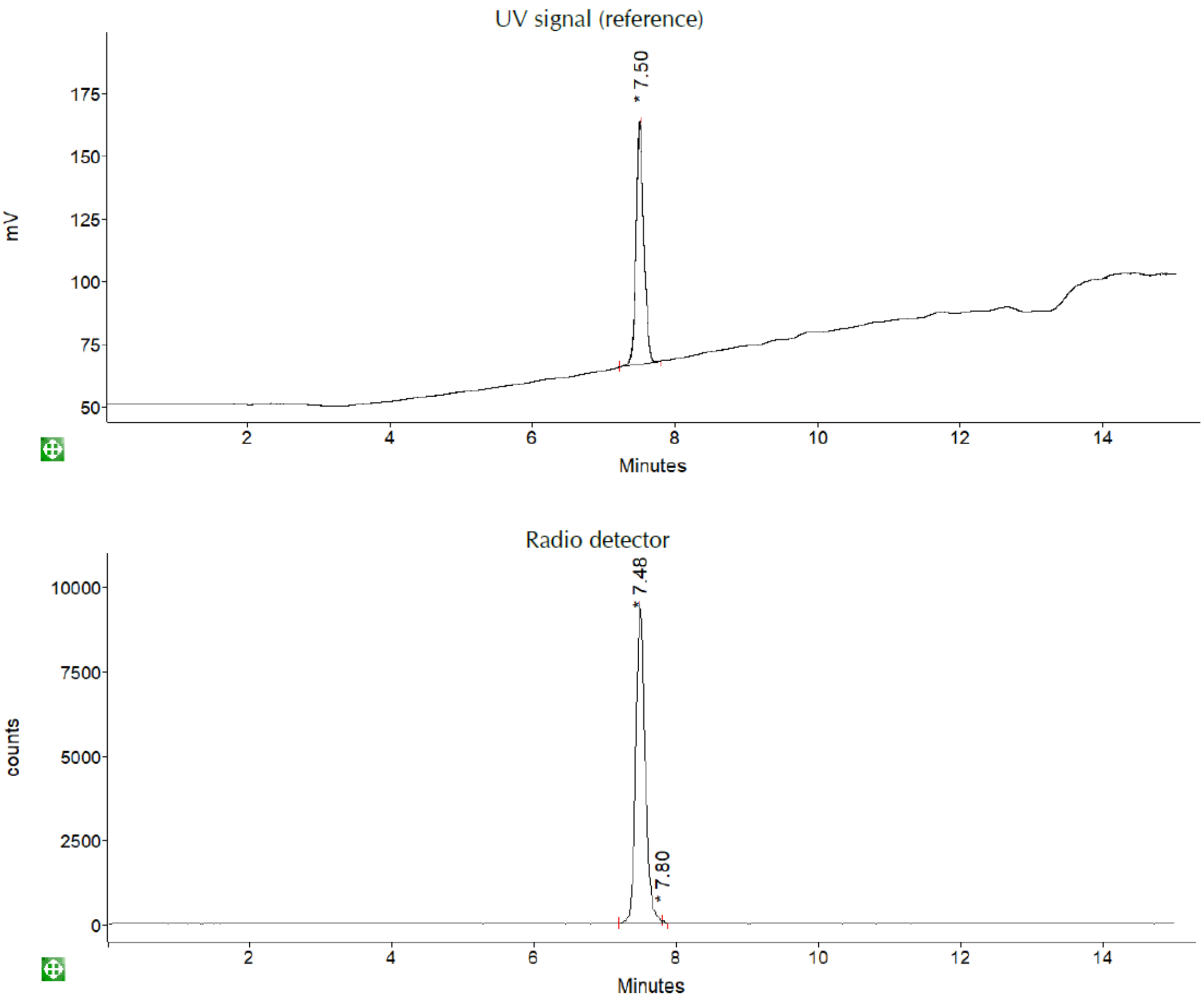

Figure S30. HPLC UV-trace of (±)pyriproxyfen as the reference (top) and radio-HPLC trace of the tritiation reaction of pyriproxyfen-derived thianthrenium salt **11-TT** (bottom).

[³H]Indomethacin methylester ([³H]15)

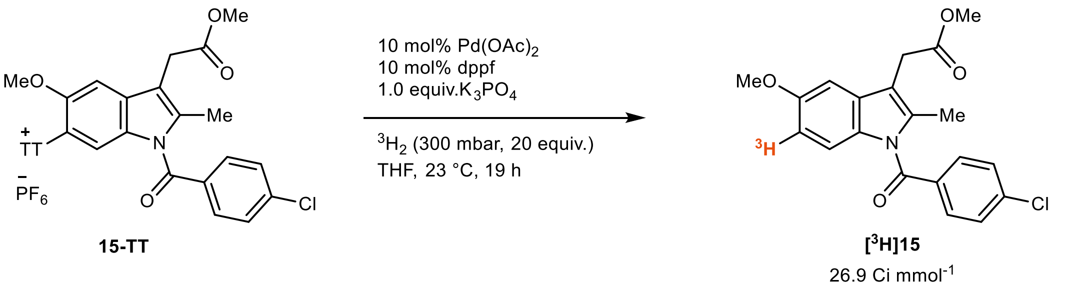

Indomethacin methylester-derived thianthrenium salt **15-TT** (2.2 mg, 3.0 µmol, 1.0 equiv.) and K_3PO_4 (0.6 mg, 3 µmol, 1 equiv.) were added to a 2-mL tritiation flask containing a magnetic stir bar. Subsequently, a stock THF solution (50 µL) containing of $Pd(OAc)_2$ (65 µg, 0.29 µmol, 10

mol%) and a stock THF solution (120 µL) containing dppf (160 µg, 0.280 µmol, 10.0 mol%) were added to the reaction mixture. The flask was attached to the tritium manifold and degassed by 3 freeze-thaw cycles. Tritium gas was introduced and the reaction mixture was stirred for 19 h at 23 °C under an atmosphere of tritium at 300 mbar (20 equiv.). The solution was cooled by liquid nitrogen and the excess tritium gas in the reaction vessel was reabsorbed on an uranium-trap for waste-tritium. The solvent was lyophilized off and labile tritium was removed by lyophilization with methanol (3 × 1 mL). The remaining solid was dissolved in 10 mL of ethanol. 2.7 GBq (73 mCi) (90% based on the thiantrenium starting material) was obtained with a radiochemical purity of 93% (determined by radio-HPLC analysis). A molar activity of 995 GBq/mmol (26.9 Ci/mmol) was achieved (determined by LC-MS analysis).

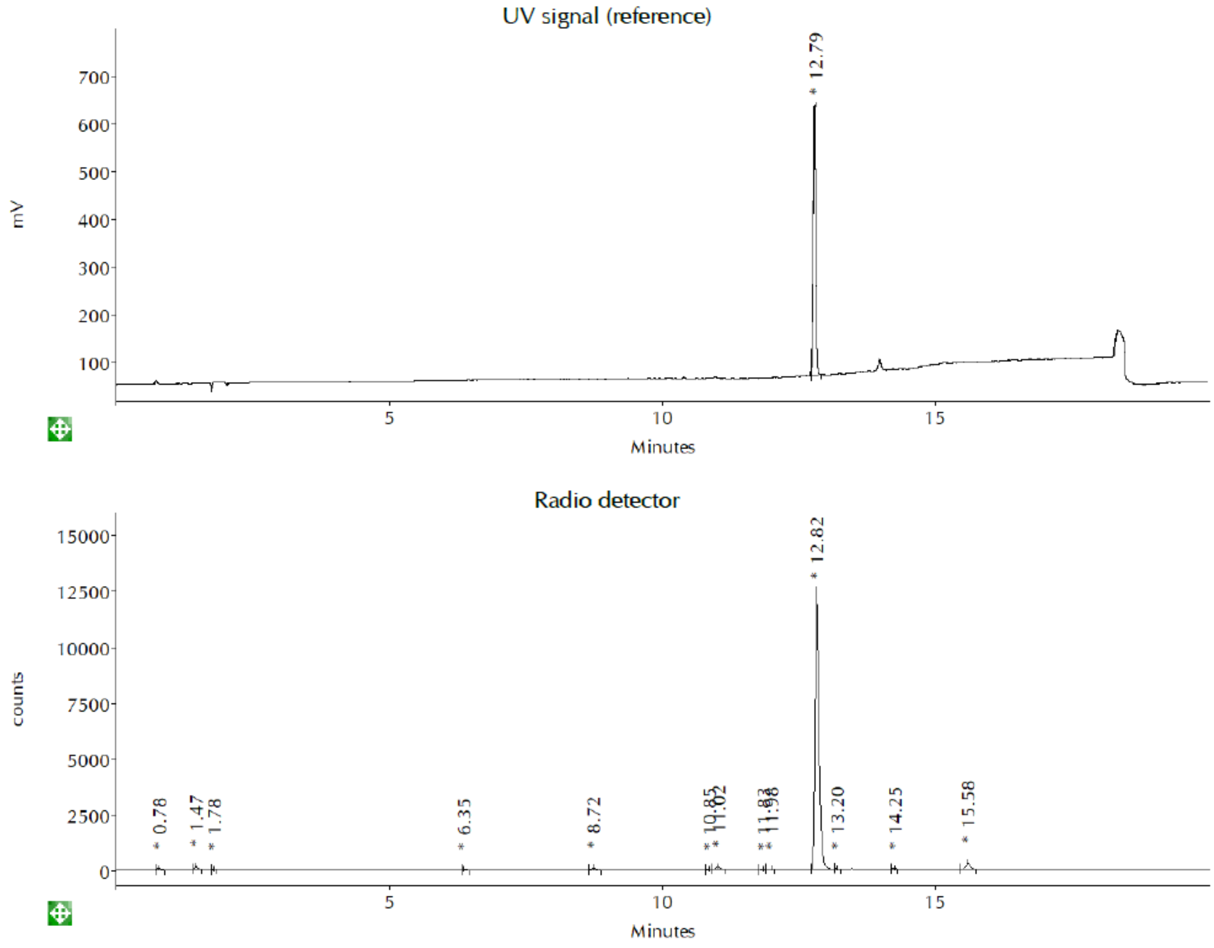

Figure S31. HPLC UV-trace of indomethacin methylester as the reference (top) and radio-HPLC trace of the reductive tritiation reaction of indomethacin methylester-derived thianthrenium salt **15-TT** (bottom).

[^{3}H]LHVS ([^{3}H]26)

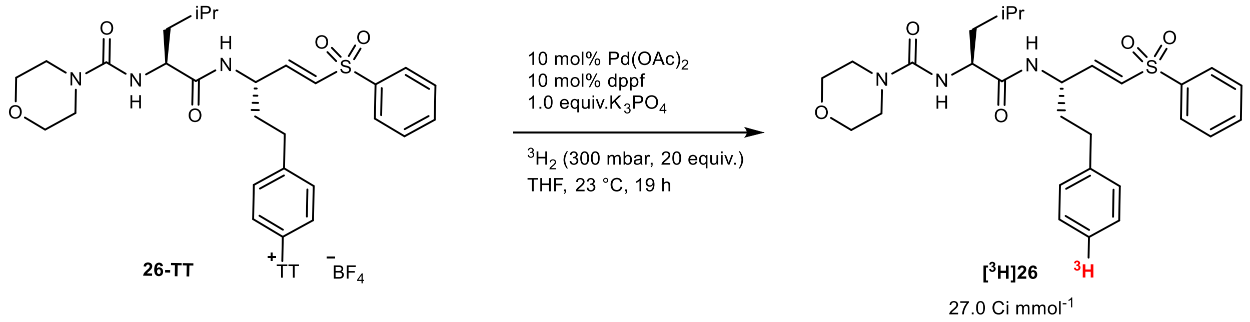

LHVS-derived thianthrenium salt **26-TT** (2.2 mg, 2.6 µmol, 1.0 equiv.) and K_3PO_4 (0.68 mg, 3.2 µmol, 1.2 equiv.) were added to a 2-mL tritiation flask containing a magnetic stir bar. Subsequently, a stock THF solution (50 µL) containing of $Pd(OAc)_2$ (65 µg, 0.29 µmol, 0.10 equiv.) and a stock THF solution (120 µL) containing dppf (160 µg, 0.280 µmol, 10.0 mol%) were added to the reaction mixture. The flask was attached to the tritium manifold and degassed by 3 freeze-thaw cycles. Tritium gas was introduced and the reaction mixture was stirred for 19 h at 23 °C under an atmosphere of tritium at 300 mbar (20 equiv.). The solution was cooled by liquid nitrogen and the excess tritium gas in the reaction vessel was reabsorbed on an uranium-trap for waste-tritium. The solvent was lyophilized off and labile tritium was removed by lyophilization with methanol (3 × 1 mL). The remaining solid was dissolved in 10 mL of ethanol. 2.6 GBq (70 mCi) (99% based on the thiantrenium starting material) was obtained with a radiochemical purity of 81% (determined by radio-HPLC analysis). A molar activity of 999 GBq/mmol (27.0 Ci/mmol) was achieved (determined by LC-MS analysis).

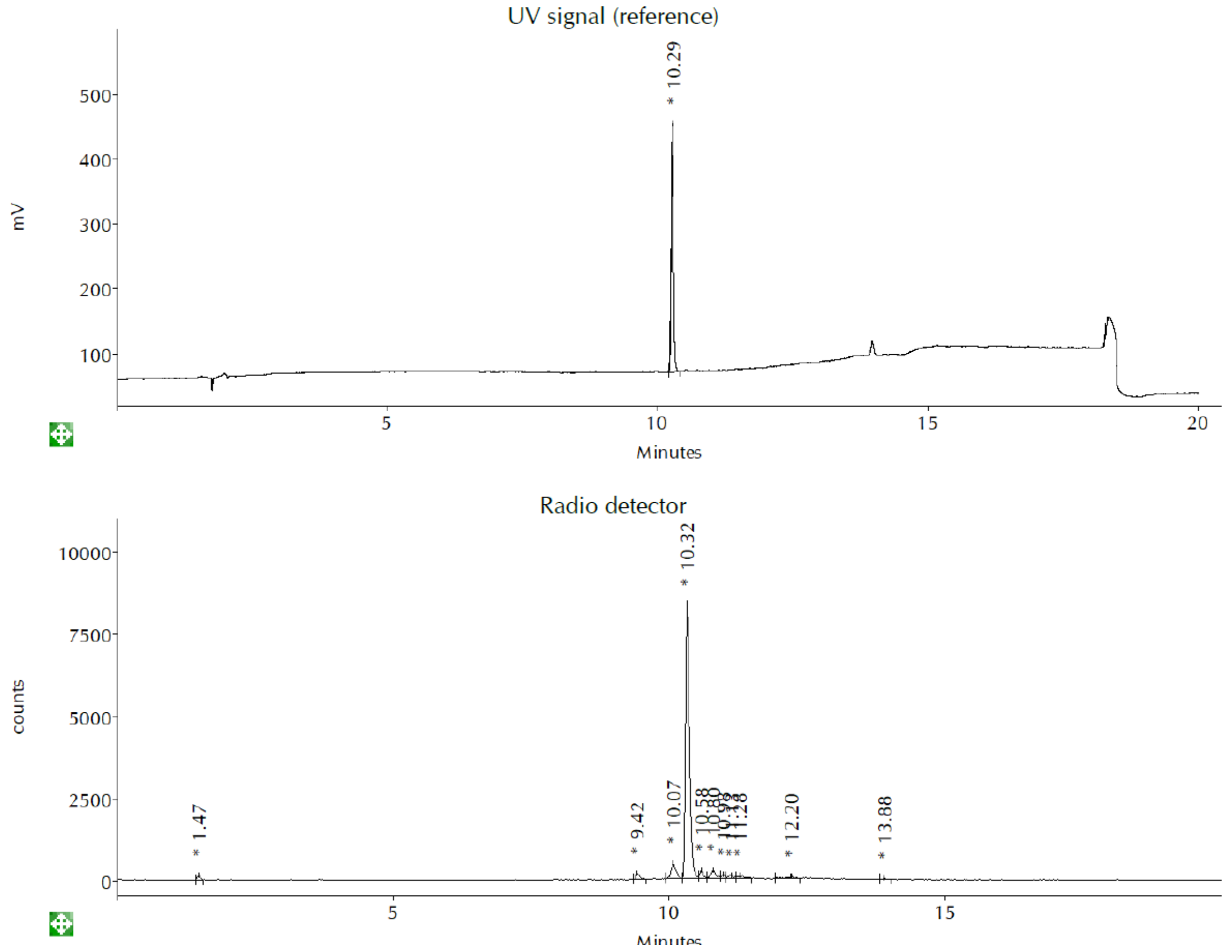

Figure S32. HPLC UV-trace of LHVS as the reference (top) and radio-HPLC trace of the reductive tritiation reaction of LHVS-derived thianthrenium salt **26-TT** (bottom).

[$^3H_2$]Etofenprox ([$^3H_2$]29)

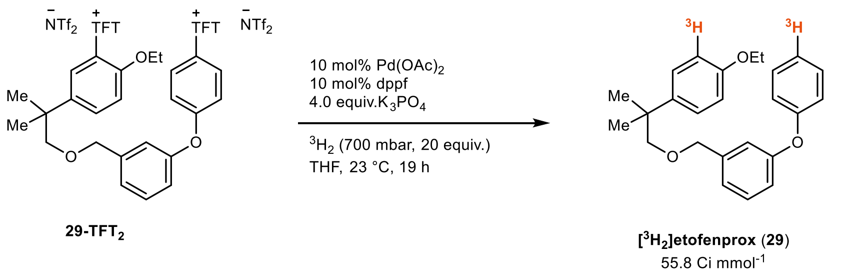

Etofenprox-derived double tetrafluorothianthrenium salt **29-TFT$_2$** (4.0 mg, 2.6 µmol, 1.0 equiv.) and K_3PO_4 (2.3 mg, 11 µmol, 4.0 equiv.) were added to a 2-mL tritiation flask containing a magnetic stir bar. Subsequently, a stock THF solution (0.5 mL) containing of $Pd(OAc)_2$ (59.4 µg, 0.260 µmol, 10.0 mol%) and dppf (149 µg, 0.260 µmol, 10.0 mol%) were added to the reaction mixture. The flask was attached to the tritium manifold and degassed by 3 freeze-thaw cycles.

Tritium gas was introduced and the reaction mixture was stirred for 19 h at 23 °C under an atmosphere of tritium at 700 mbar (20 equiv.). The solution was cooled by liquid nitrogen and the excess tritium gas in the reaction vessel was reabsorbed on an uranium-trap for waste-tritium. The solvent was lyophilized off and labile tritium was removed by lyophilization with methanol (3 × 1 mL). The remaining solid was dissolved in 10 mL of ethanol. 4.8 GBq (131 mCi) (90% based on the thiantrenium starting material) was obtained with a radiochemical purity of 91% (determined by radio-HPLC analysis). A molar activity of 2.06 TBq/mmol (55.8 Ci/mmol) was achieved (determined by LC-MS analysis).

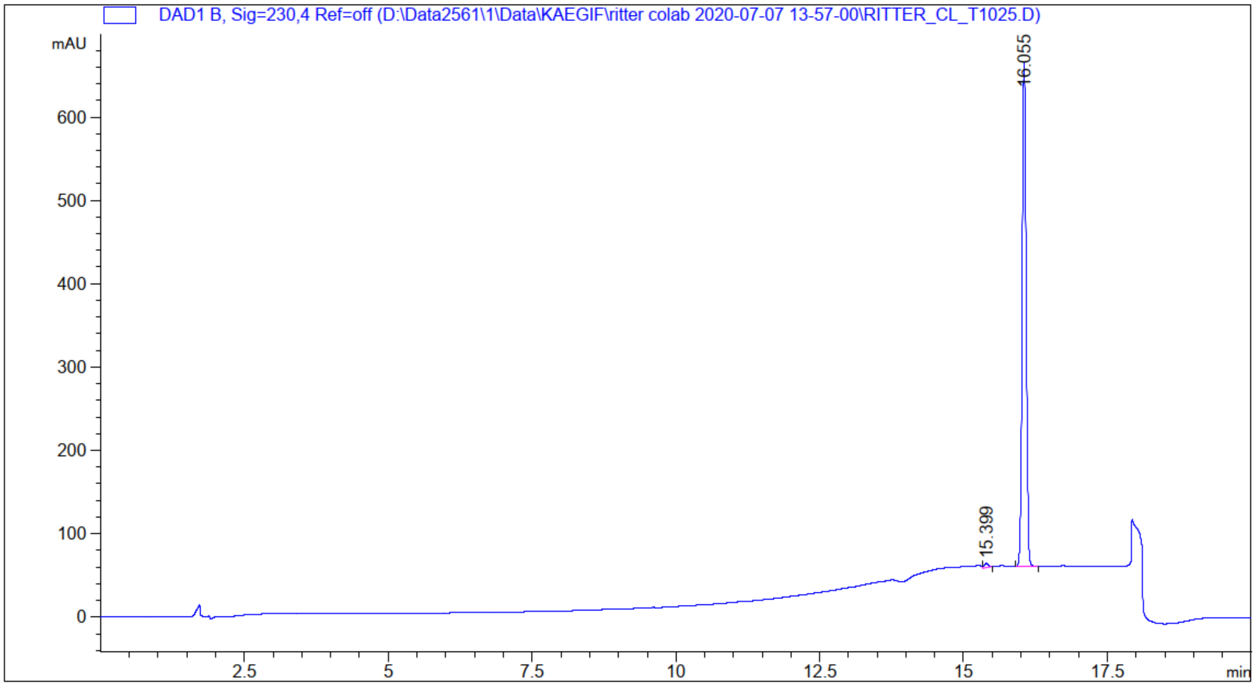

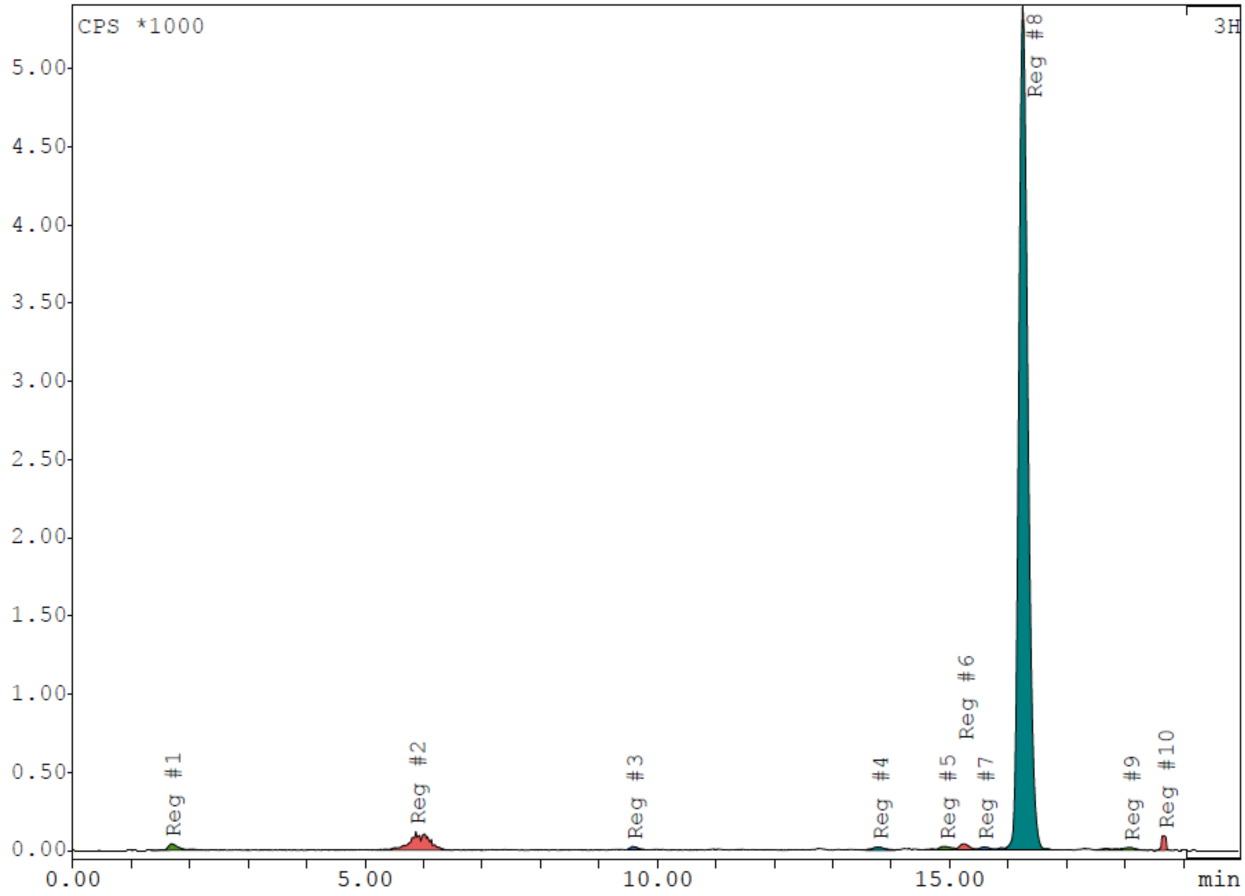

Figure S33. HPLC UV-trace of etofenprox as the reference (top) and radio-HPLC trace of the reductive tritiation reaction of etofenprox-derived double tetrafluorothianthrenium salt **29-TFT$_2$**.

SPECTROSCOPIC DATA

^{1}H NMR of 2-nitro-biphenyl-derived tetrafluorothianthrenium salt (19-TFT)

CD_3CN, 23 °C

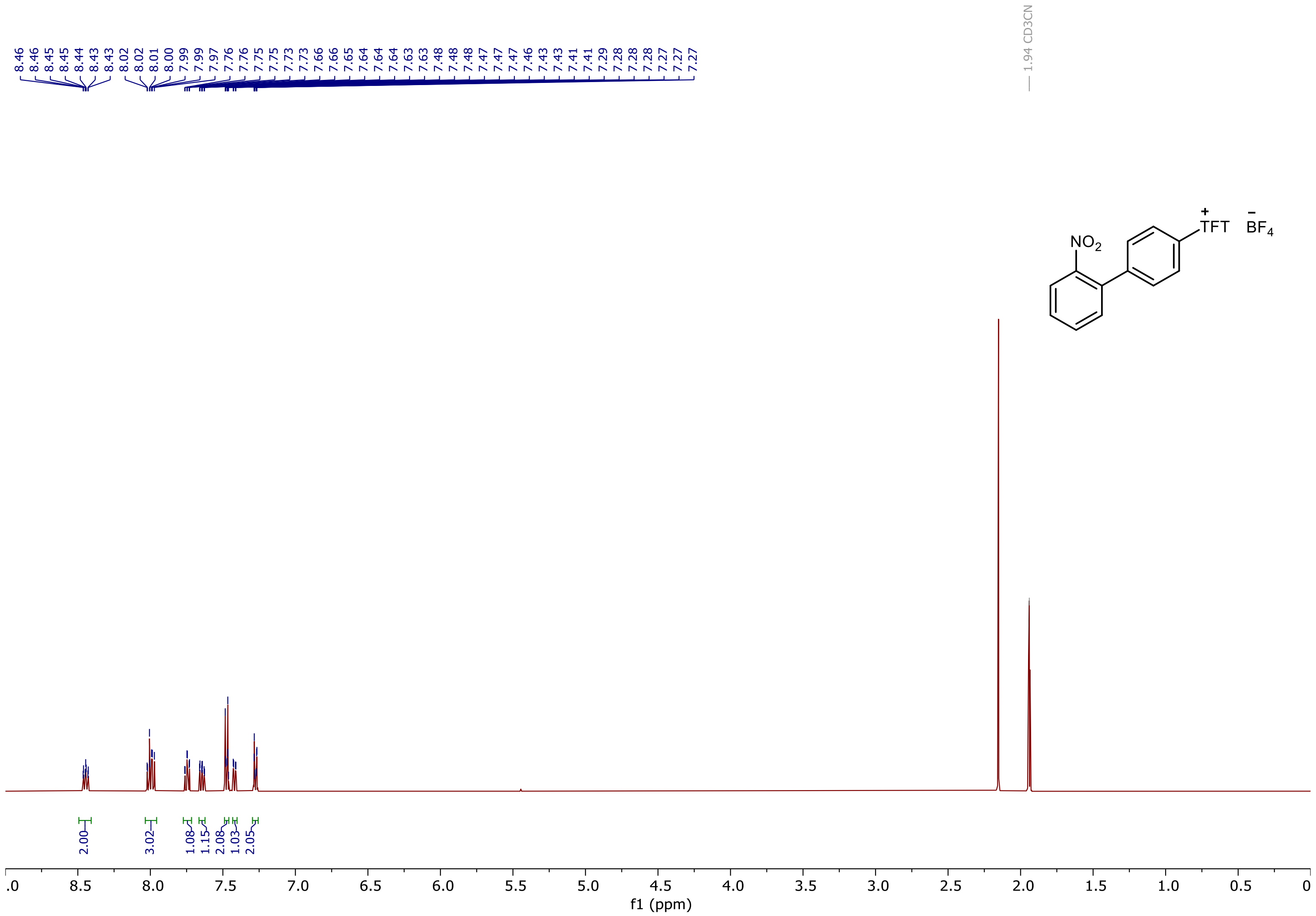

^{13}C NMR of 2-nitro-biphenyl-derived tetrafluorothianthrenium salt (19-TFT)

CD_3CN, 23 °C

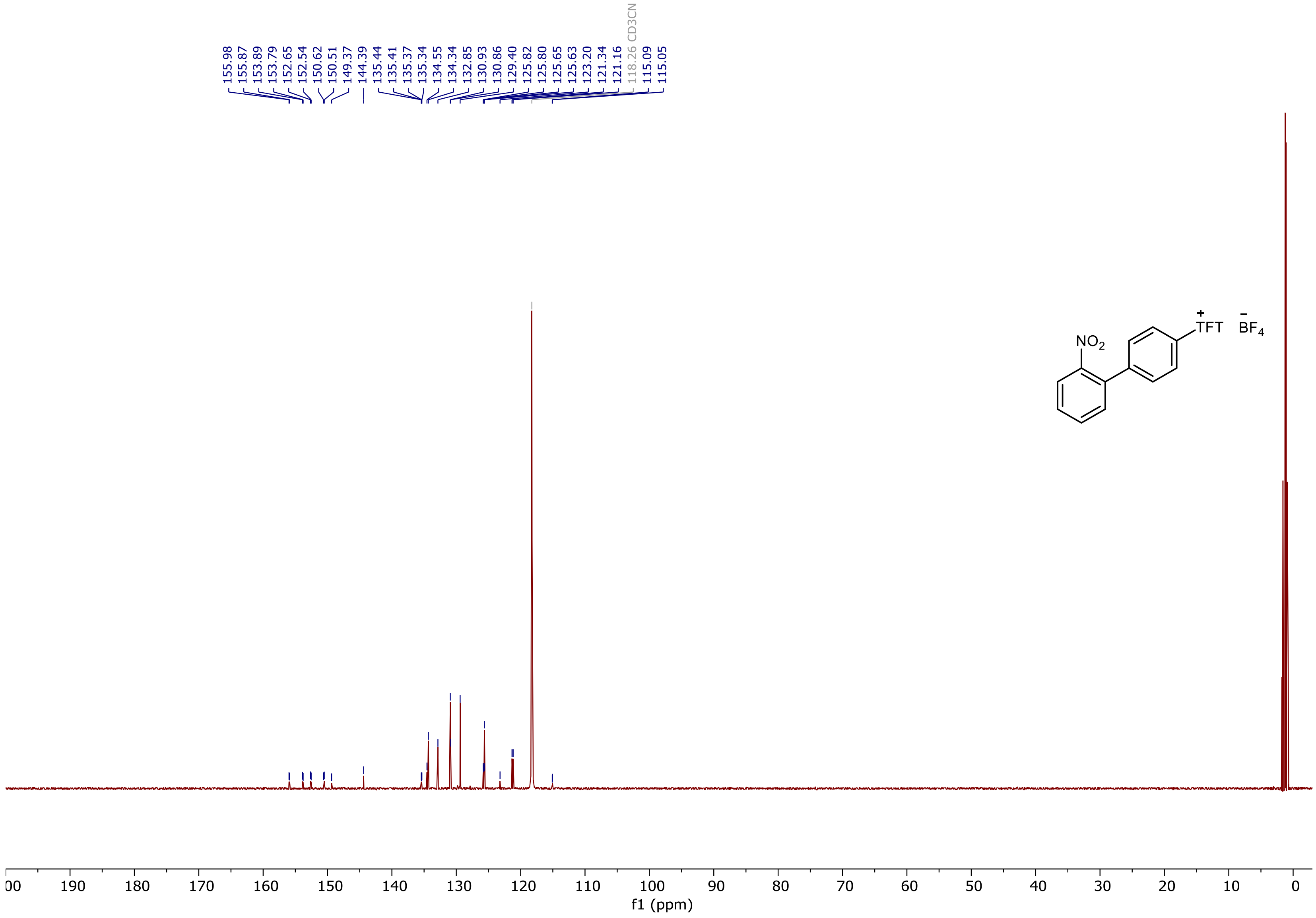

^{19}F NMR of 2-nitro-biphenyl-derived tetrafluorothianthrenium salt (19-TFT)

CD_3CN, 23 °C

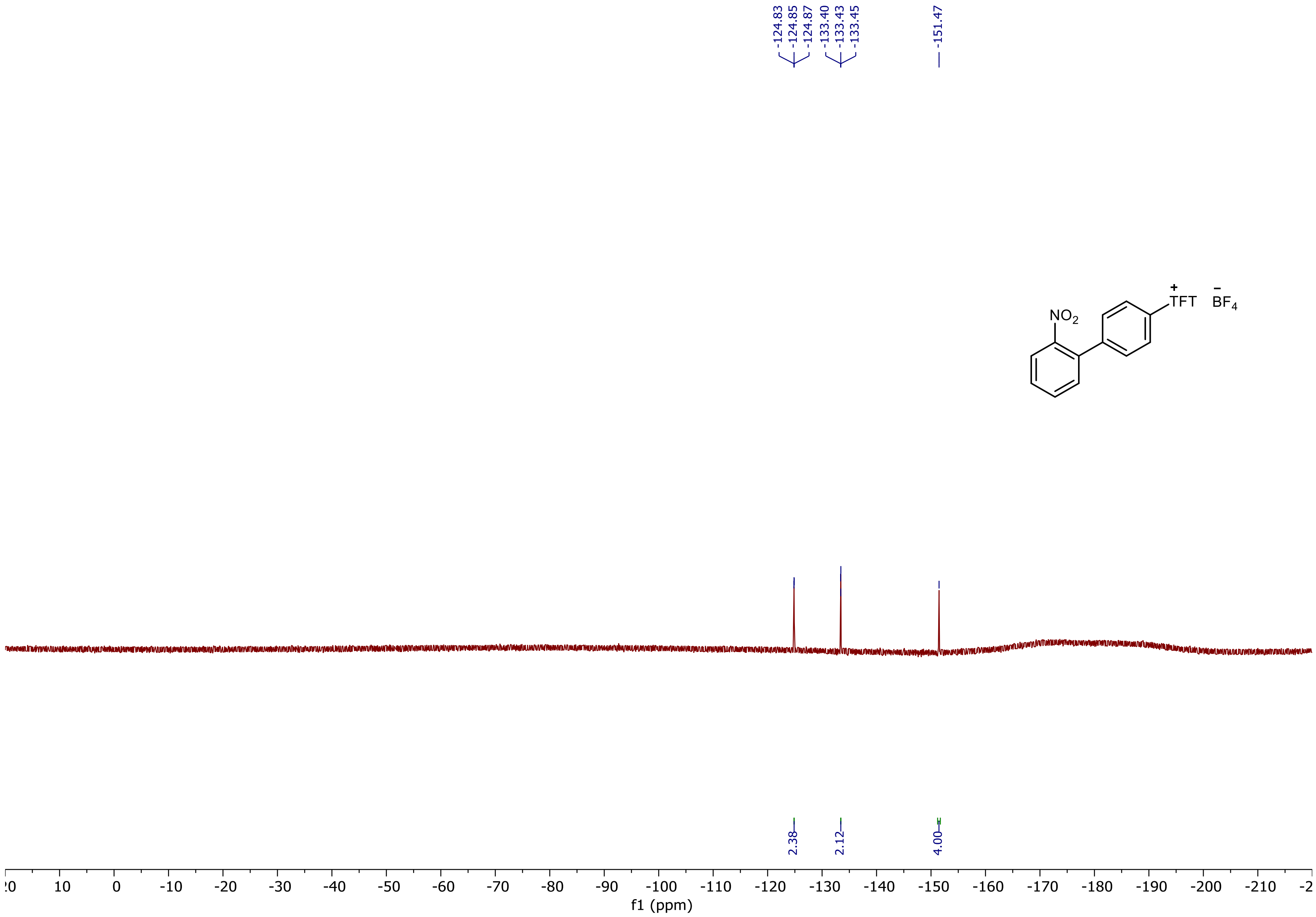

^{1}H NMR of 2,2,2-trichloroethyl (4-phenylbutyl)carbamate (24)

$CDCl_3$, 23 °C

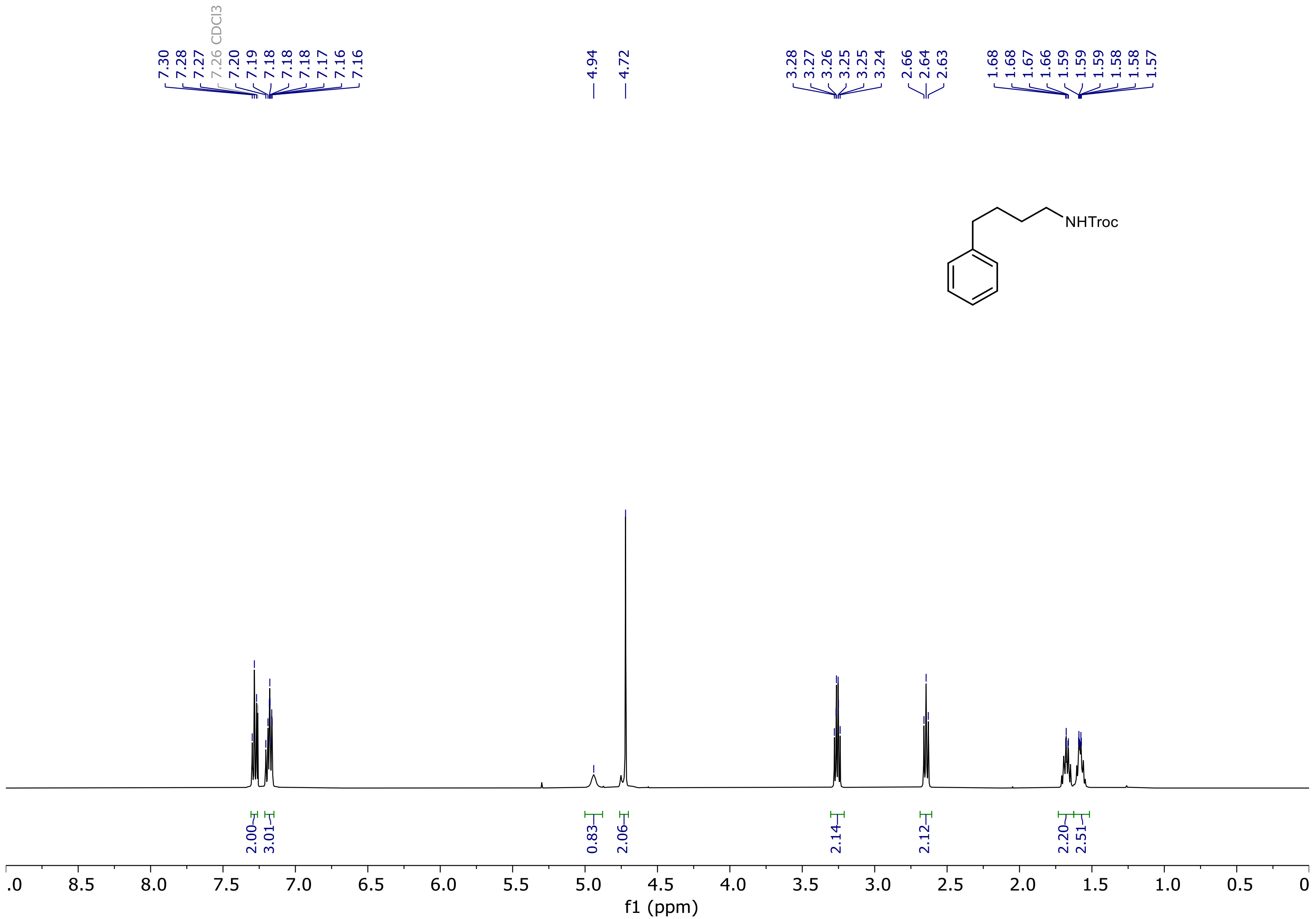

^{13}C NMR of 2,2,2-trichloroethyl (4-phenylbutyl)carbamate (24)

$CDCl_3$, 23 °C

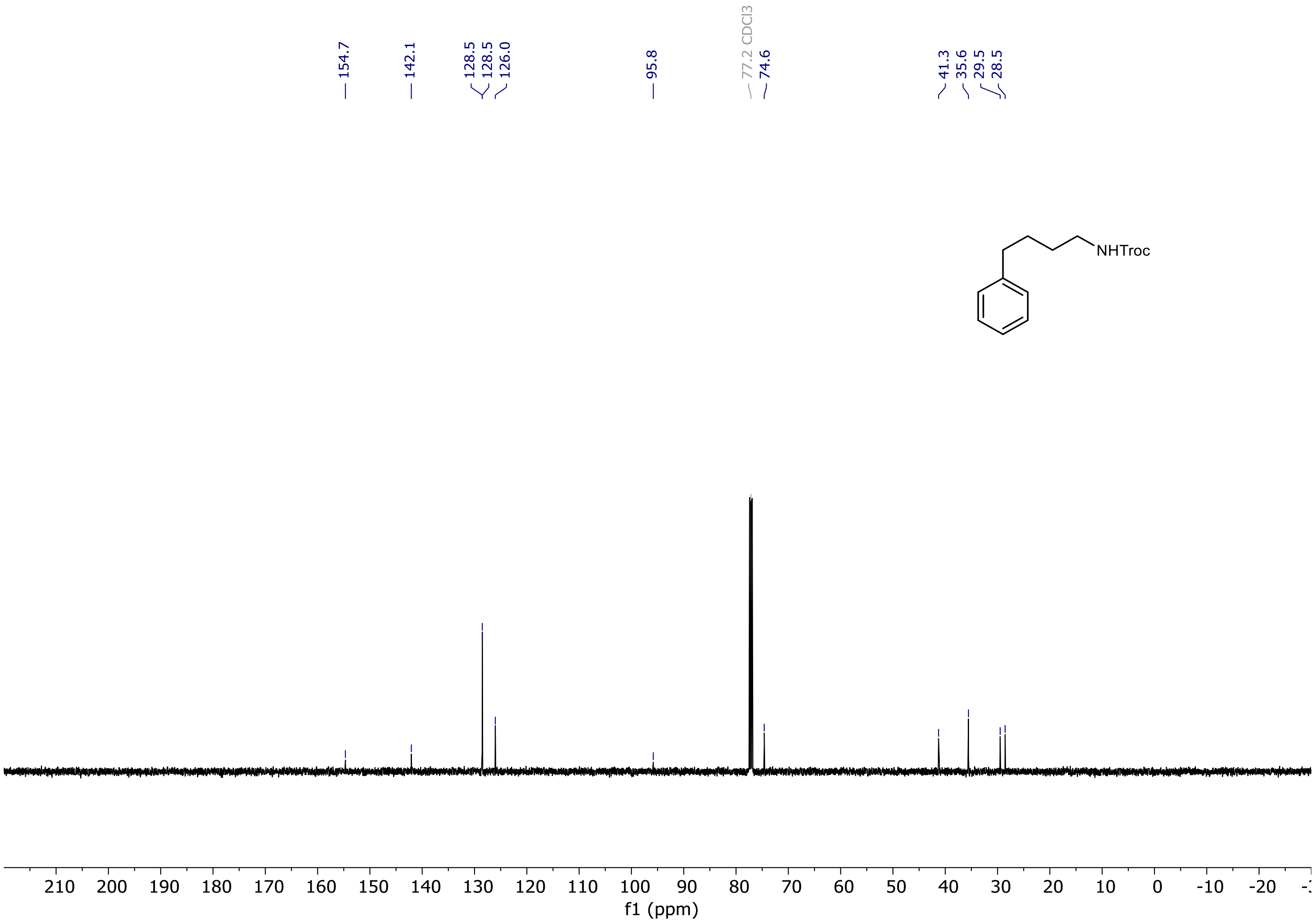

^{1}H NMR of 2,2,2-trichloroethyl (4-phenylbutyl)carbamate-derived thianthrenium salt (24-TT)

$CDCl_3$, 23 °C

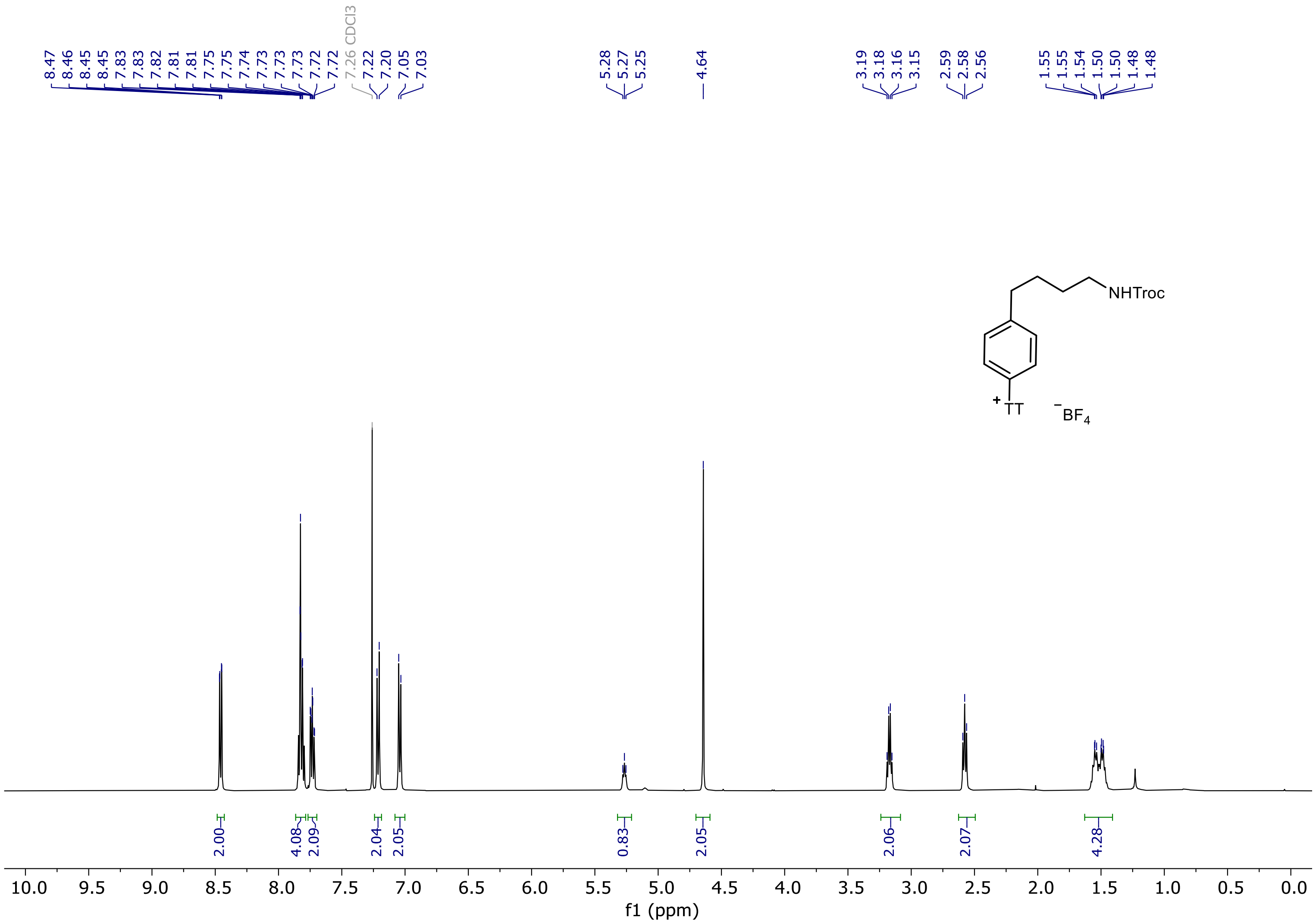

^{13}C NMR of 2,2,2-trichloroethyl (4-phenylbutyl)carbamate-derived thianthrenium salt (24-TT)

$CDCl_3$, 23 °C

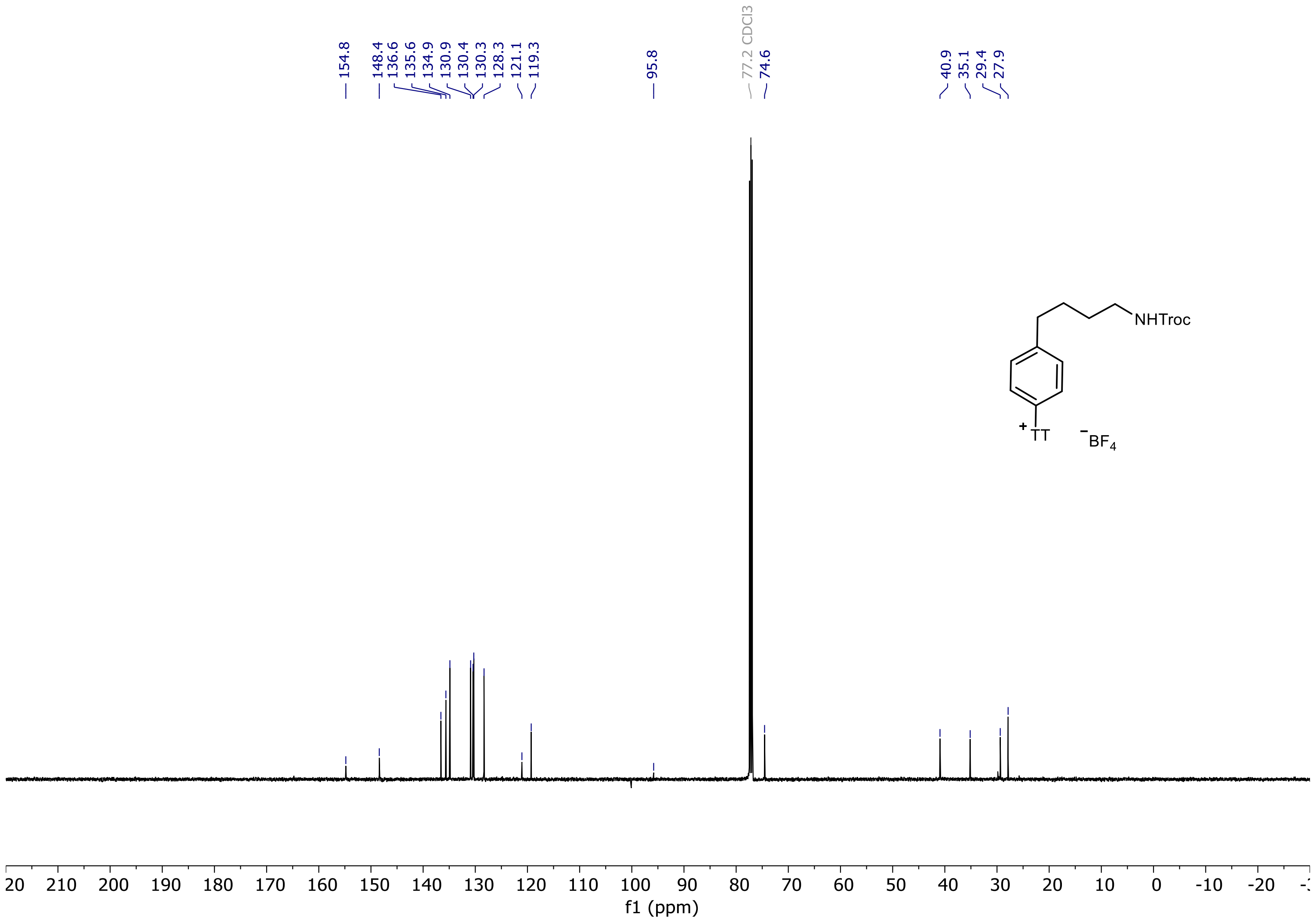

^{19}F NMR of 2,2,2-trichloroethyl (4-phenylbutyl)carbamate-derived thianthrenium salt (24-TT)

CDCl$_3$, 23 °C

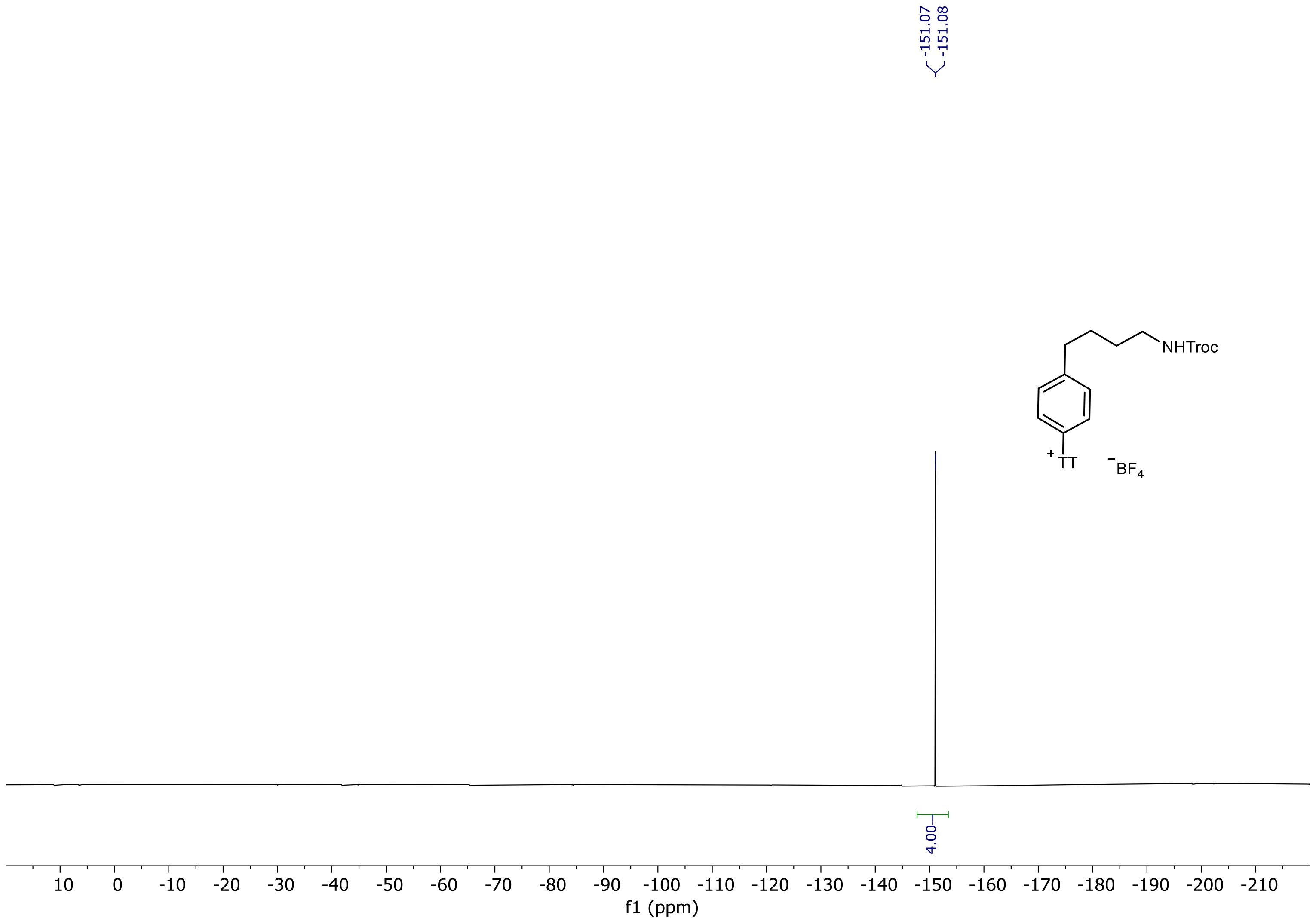

^{1}H NMR of LHVS-derived thianthrenium salt (26-TT)

CD_2Cl_2, 23 °C

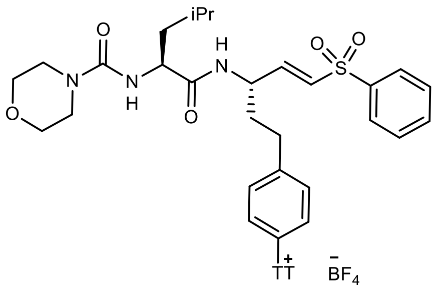

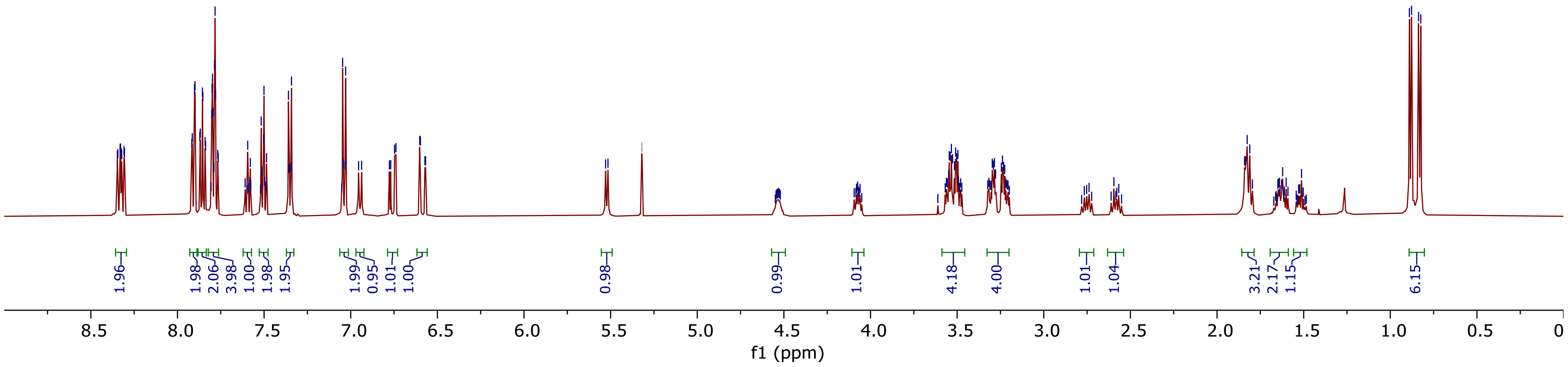

^{13}C NMR of LHVS-derived thianthrenium salt (26-TT)

CD_2Cl_2, 23 °C

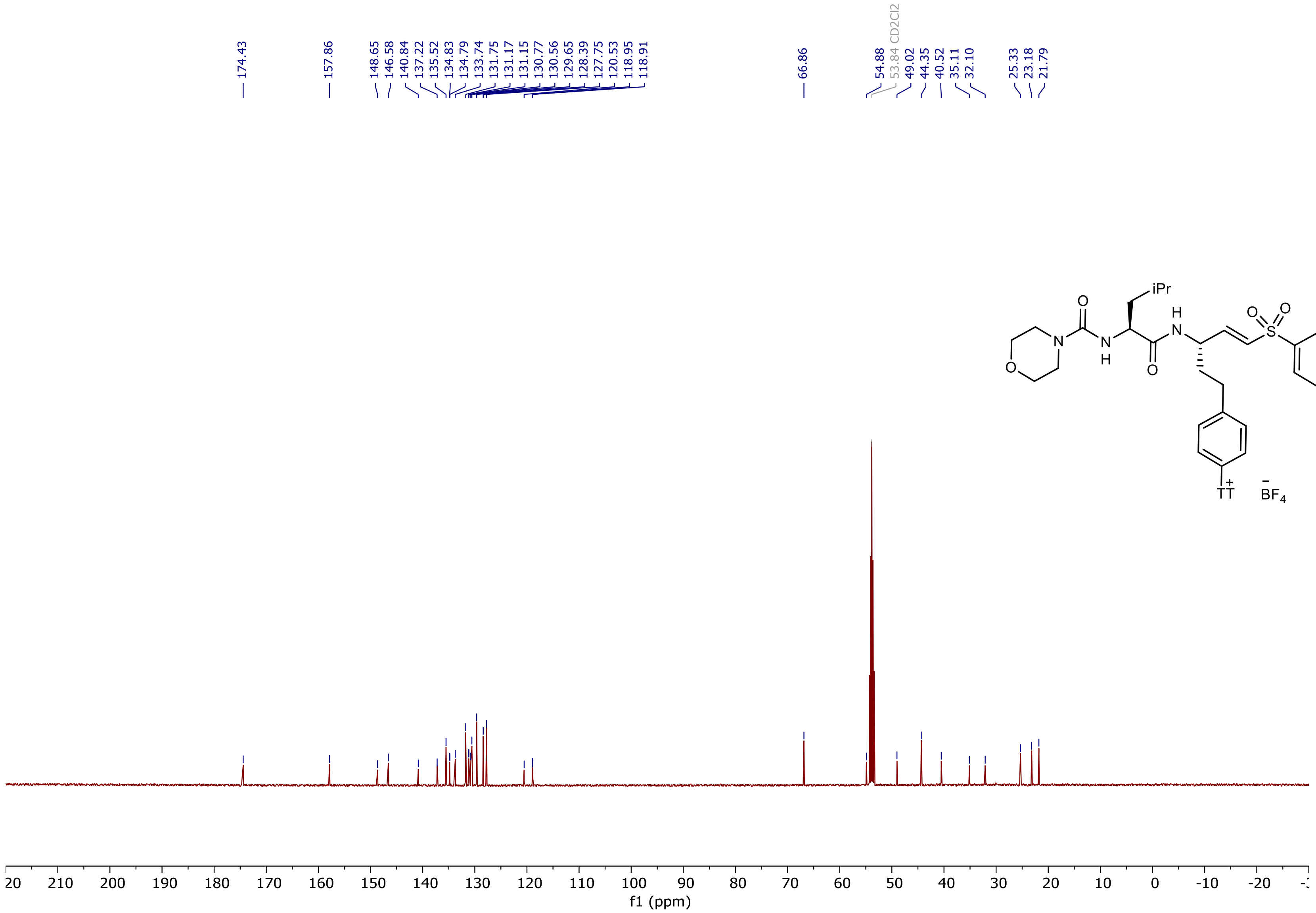

^{19}F NMR of LHVS-derived thianthrenium salt (26-TT)

CD_2Cl_2, 23 °C

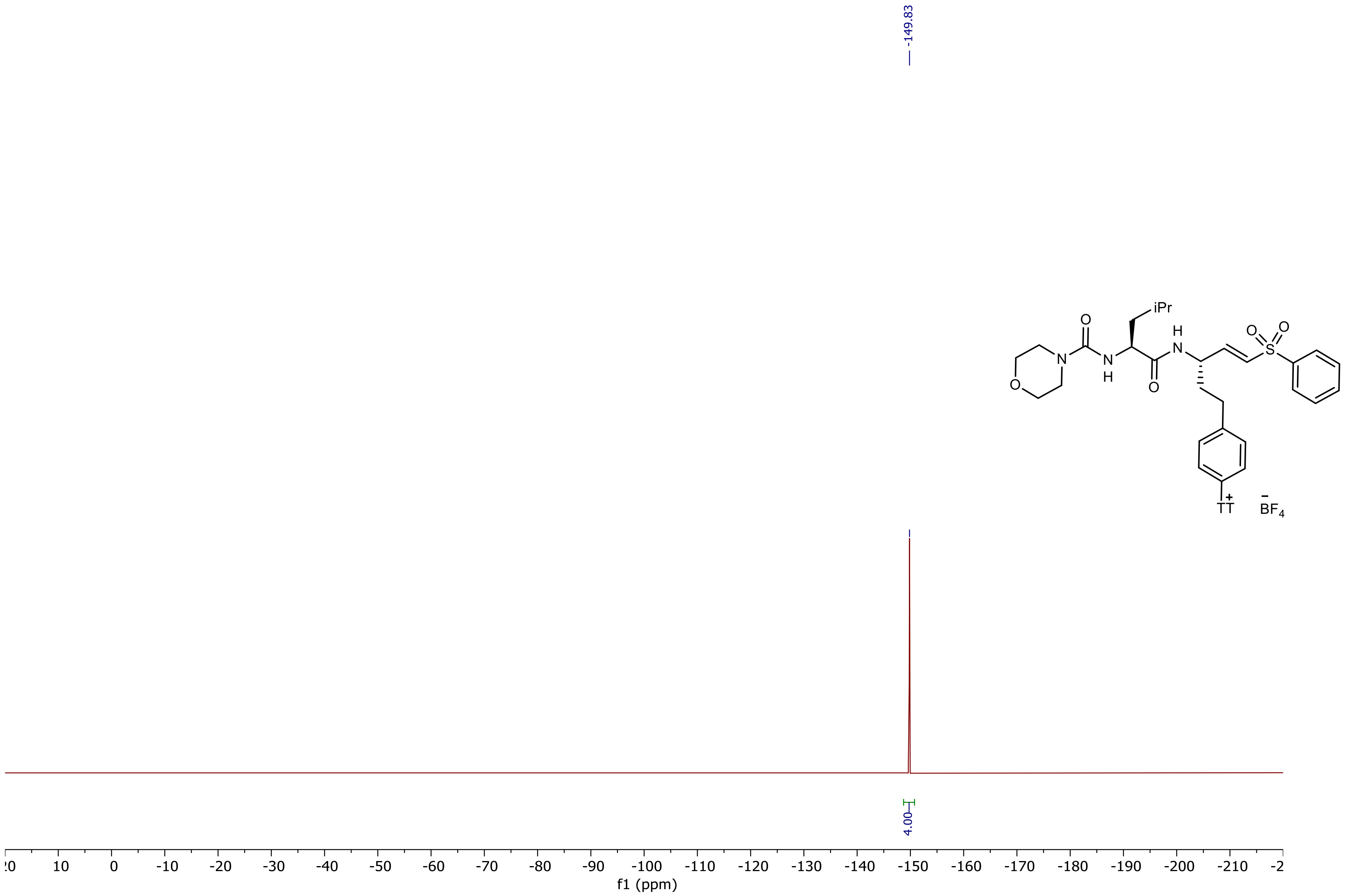

^{1}H NMR of (2-bromoethyl)benzene-derived thianthrenium salt (27-TT1)

$CDCl_3$, 23 °C

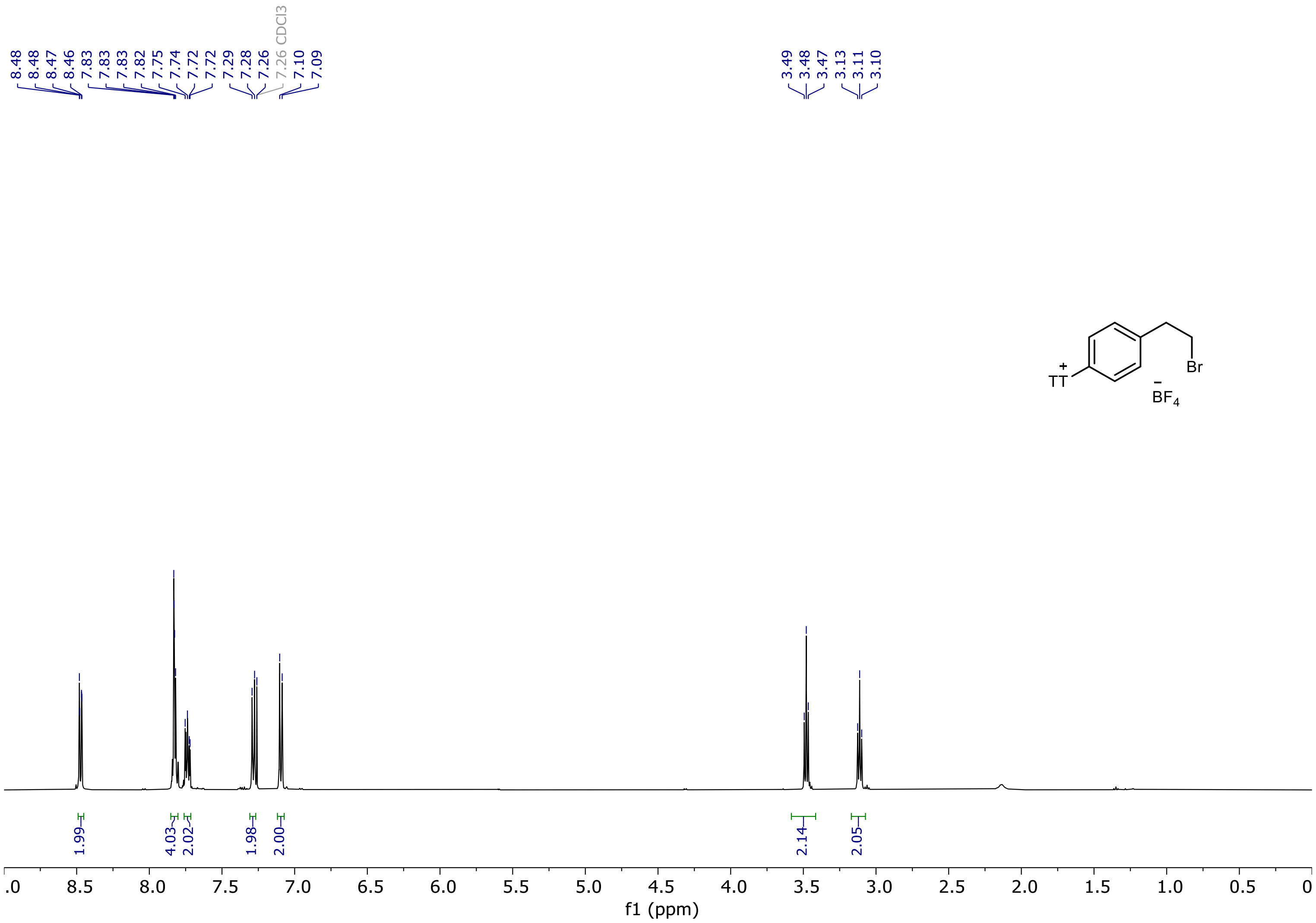

^{13}C NMR of (2-bromoethyl)benzene-derived thianthrenium salt (27-TT1)

$CDCl_3$, 23 °C

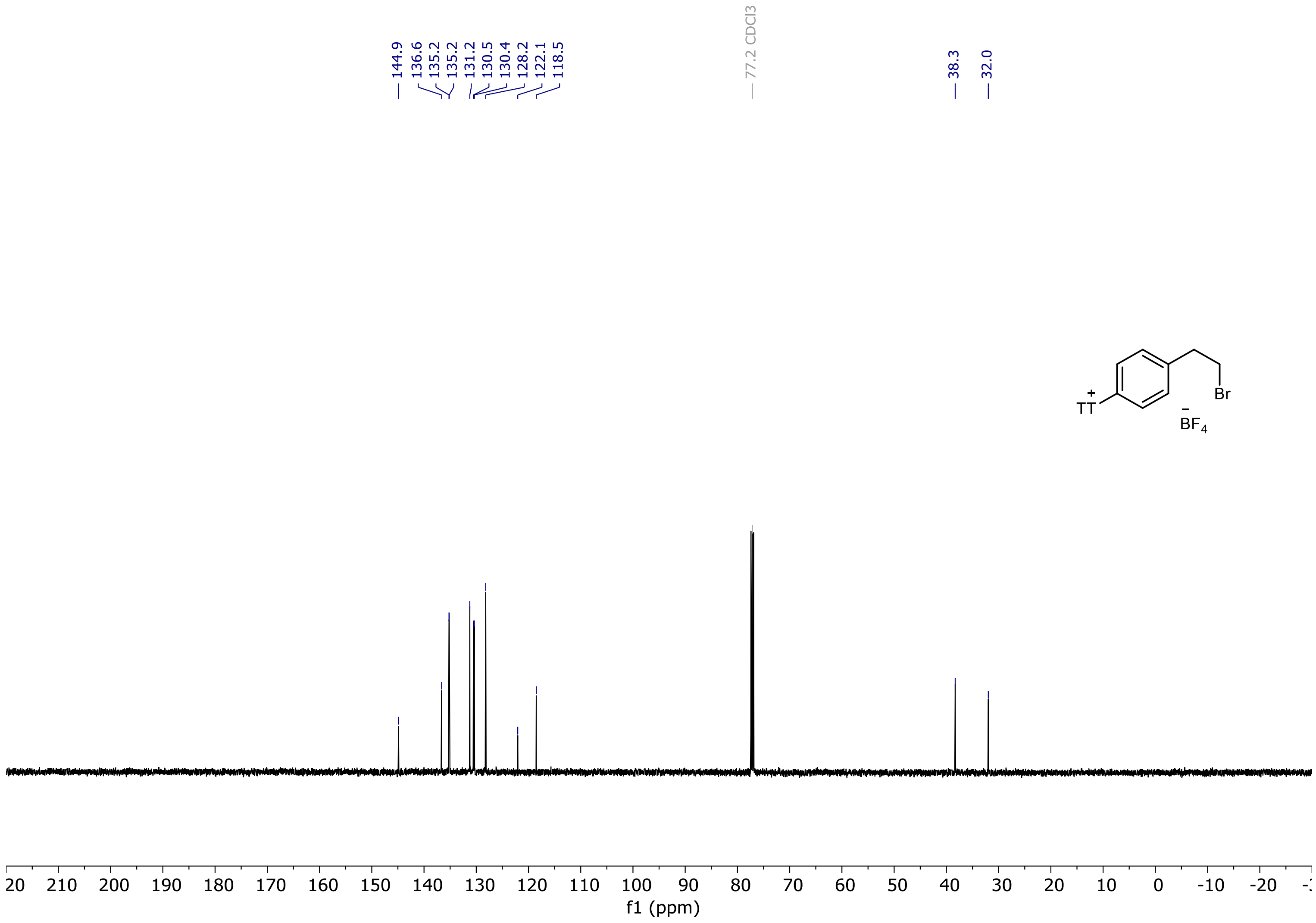

^{19}F NMR of (2-bromoethyl)benzene-derived thianthrenium salt (27-TT1)

$CDCl_3$, 23 °C

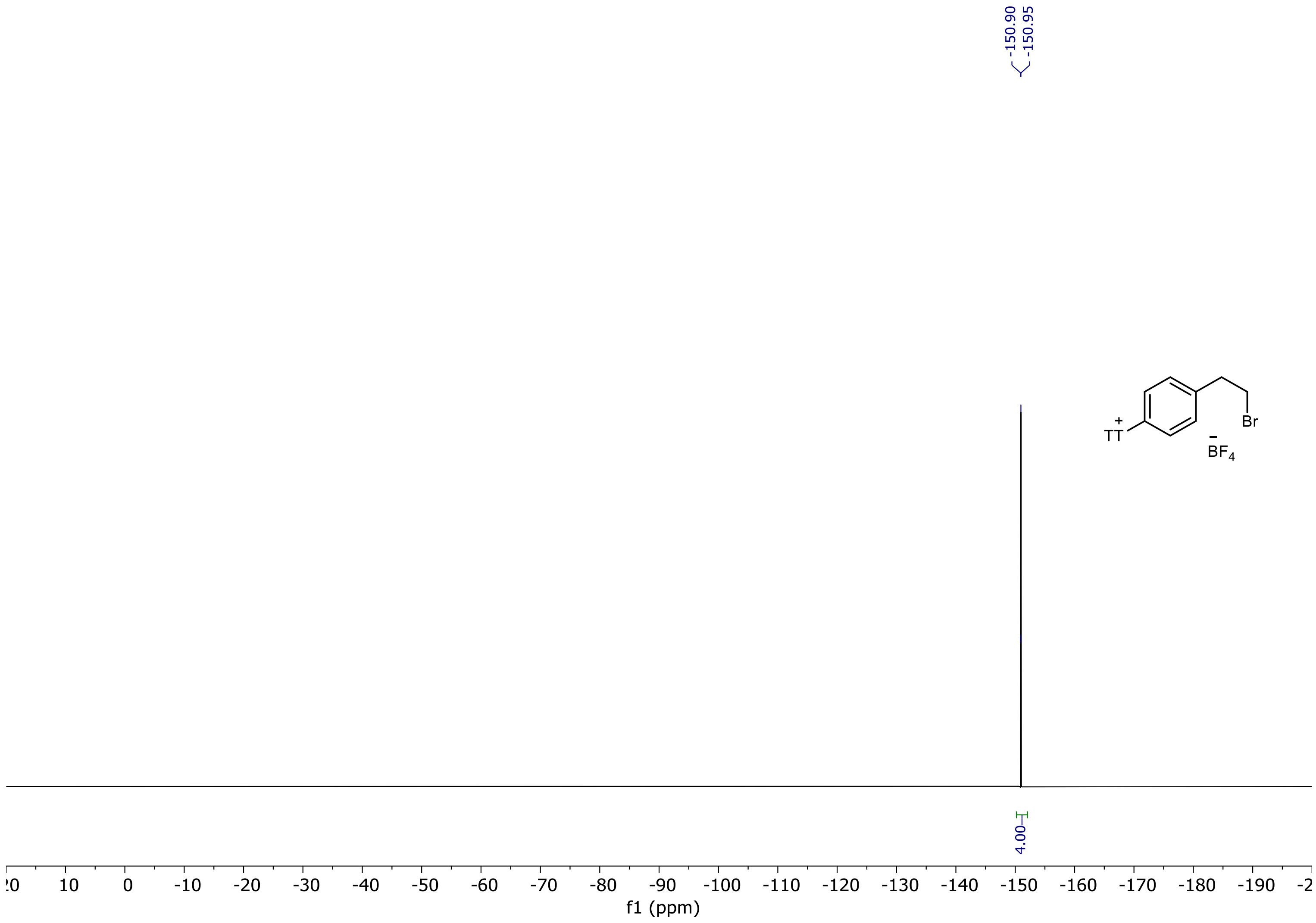

^{1}H NMR of styrene-derived thianthrenium salt (27-TT)

CD_3OD, 23 °C

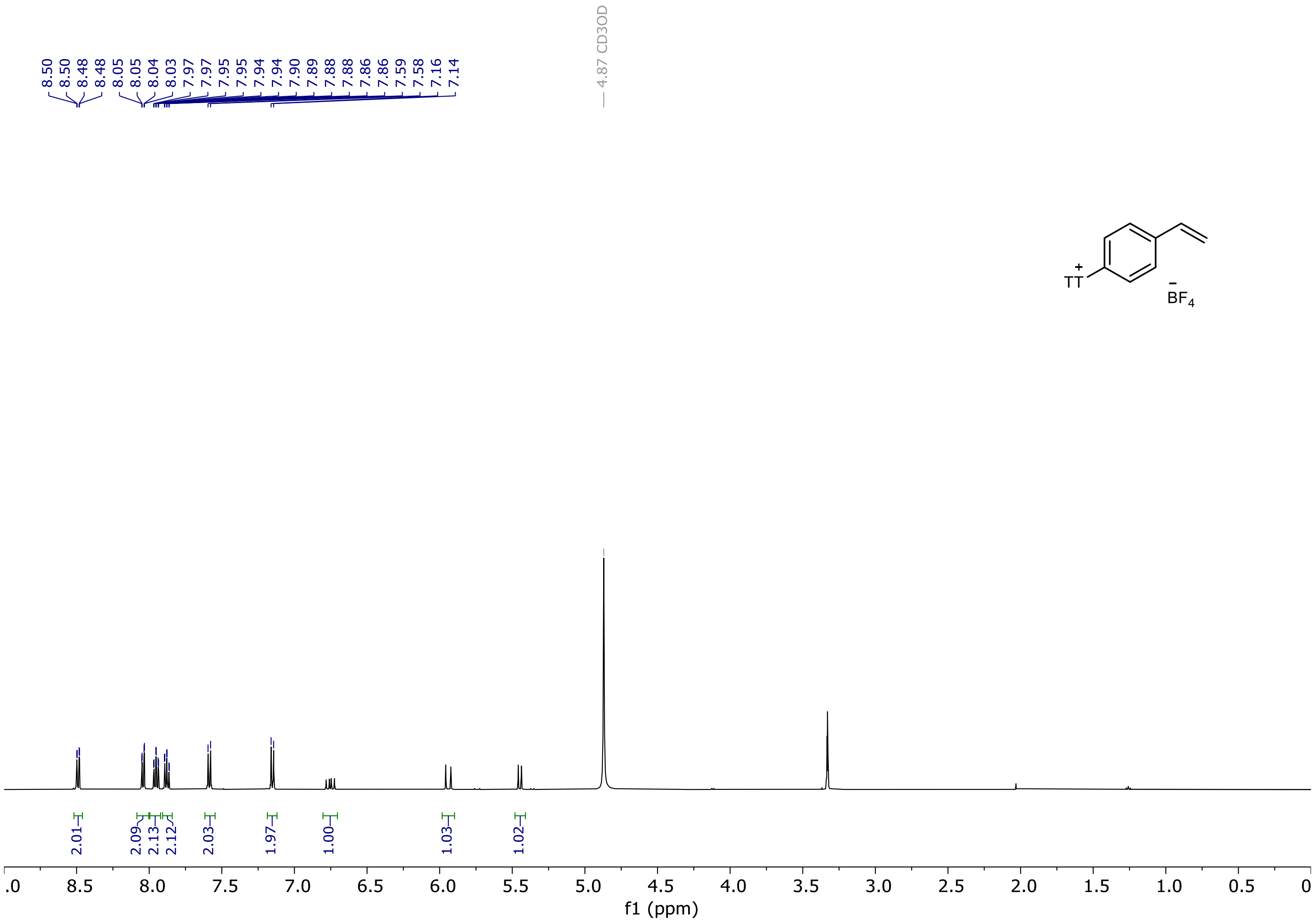

^{13}C NMR of styrene-derived thianthrenium salt (27-TT)

CD_3OD, 23 °C

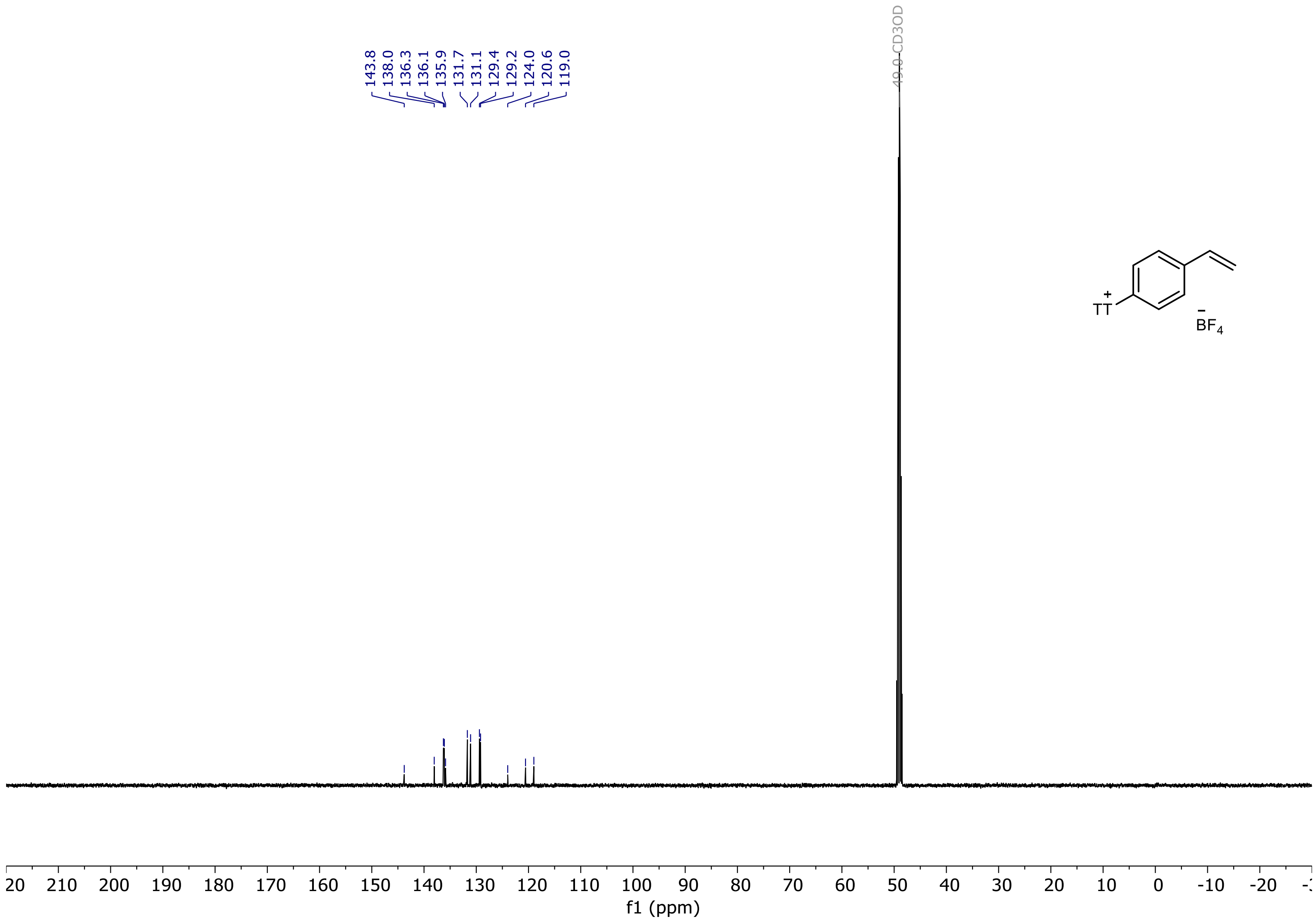

^{19}F NMR of styrene-derived thianthrenium salt (27-TT)

CD_3OD, 23 °C

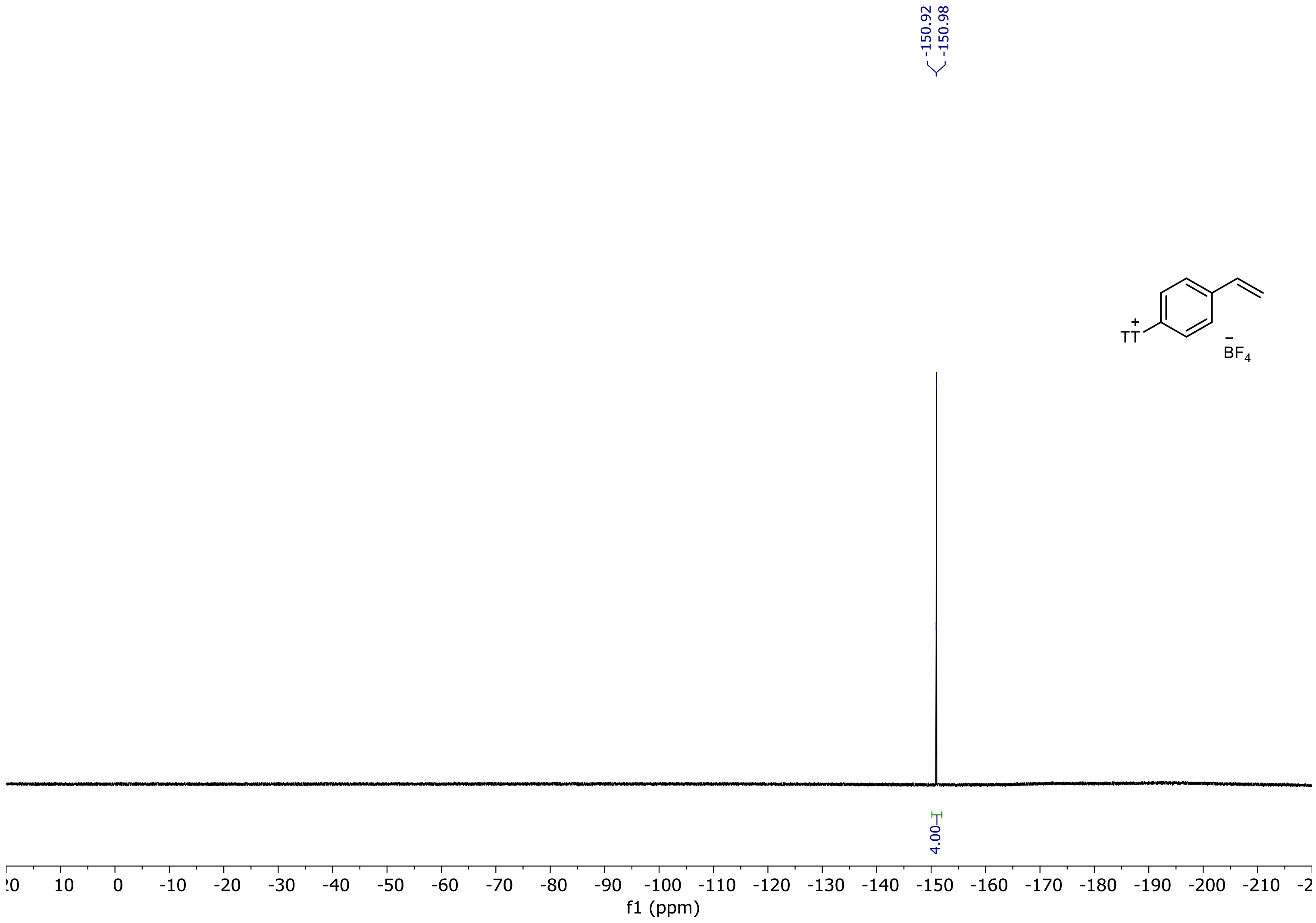

^{1}H NMR of etofenprox-derived tetrafluorothianthrenium salt (29-TFT$_2$)

CD_2Cl_2, 23 °C

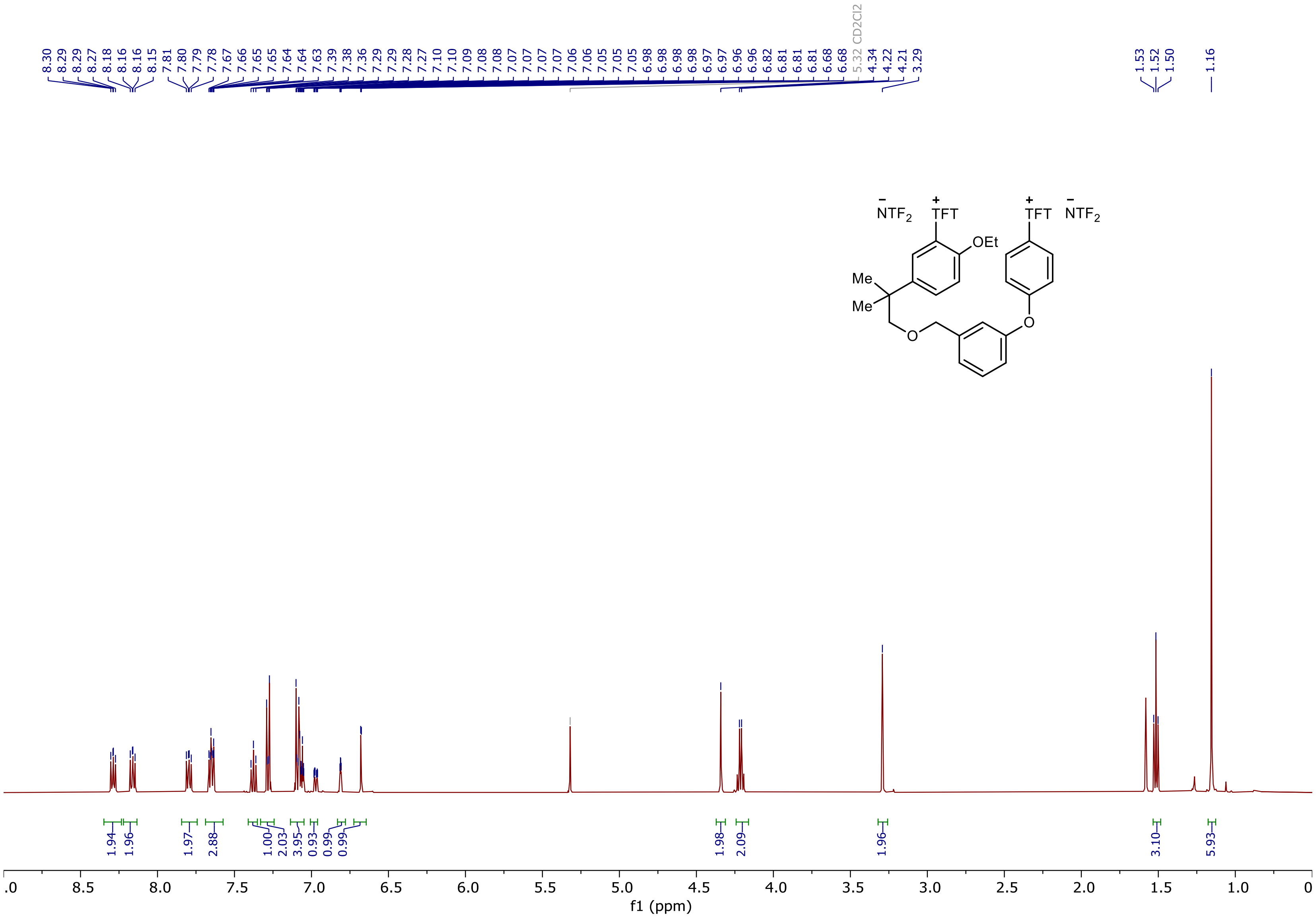

^{13}C NMR of etofenprox-derived tetrafluorothianthrenium salt (29-TFT$_2$)

CD_2Cl_2, 23 °C

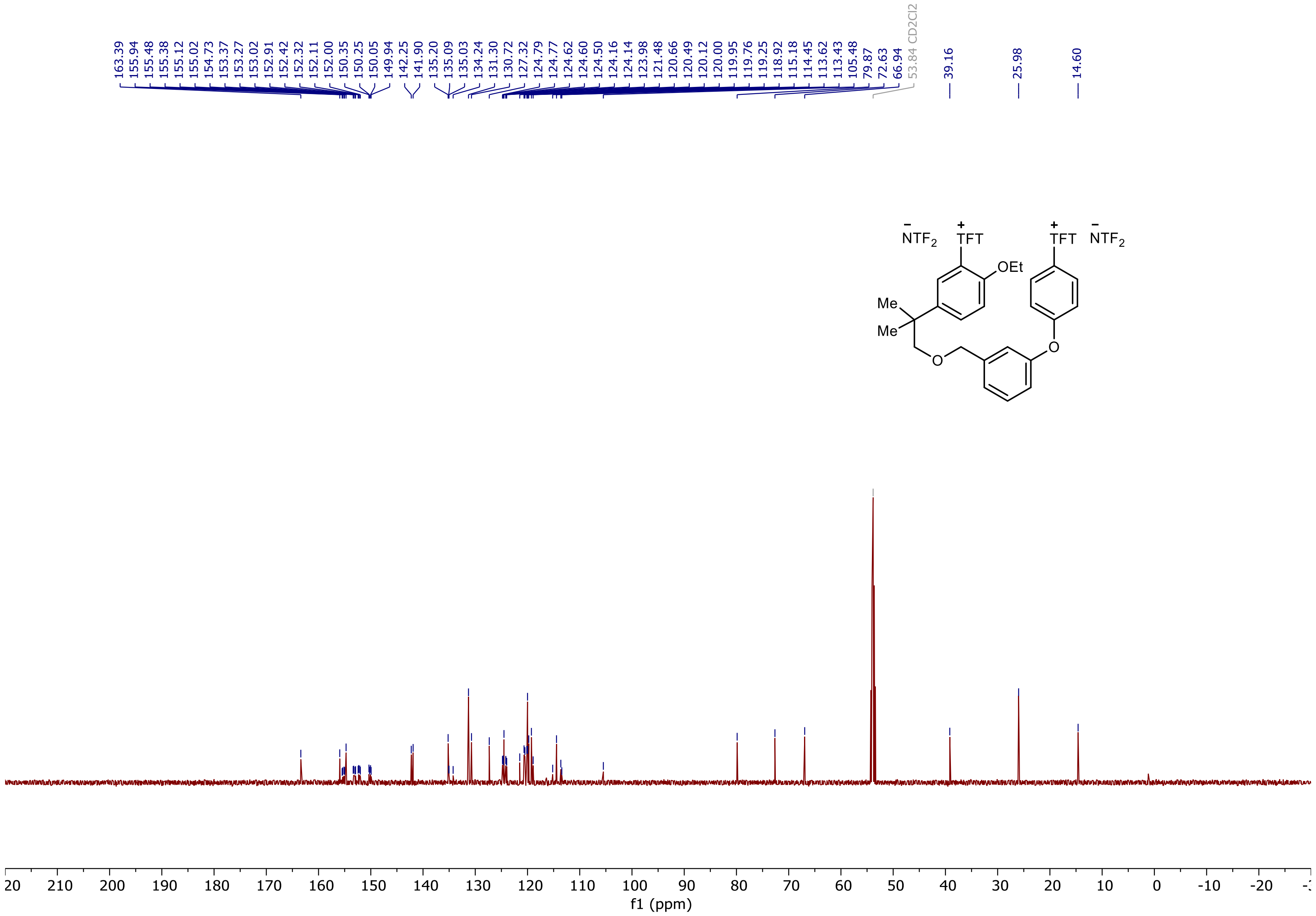

^{19}F NMR of etofenprox-derived tetrafluorothianthrenium salt (29-TFT$_2$)

CD_2Cl_2, 23 °C

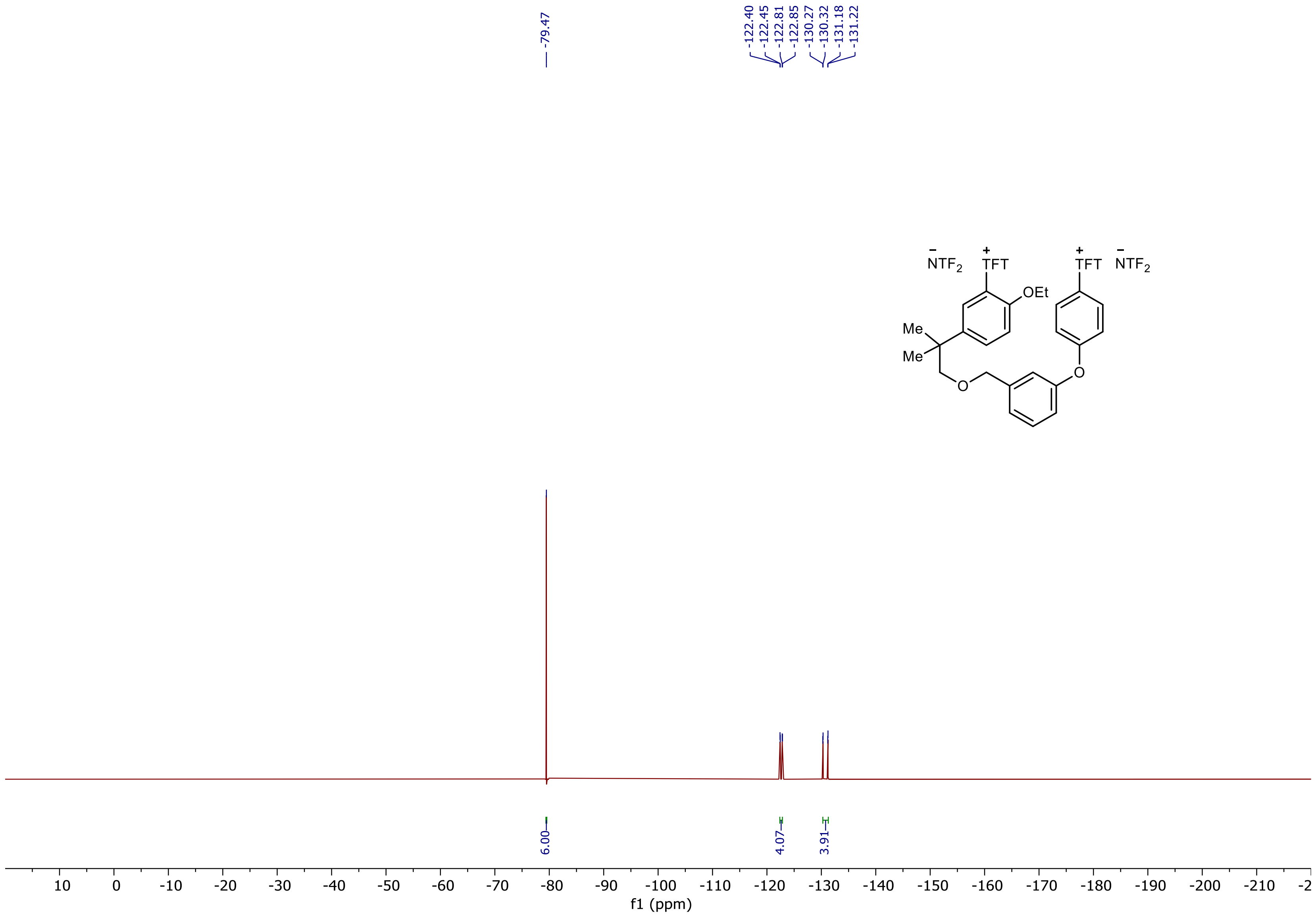

^{1}H NMR of benazepril methyl ester-derived thianthrenium salt (30-TT)

CD_2Cl_2, 23 °C

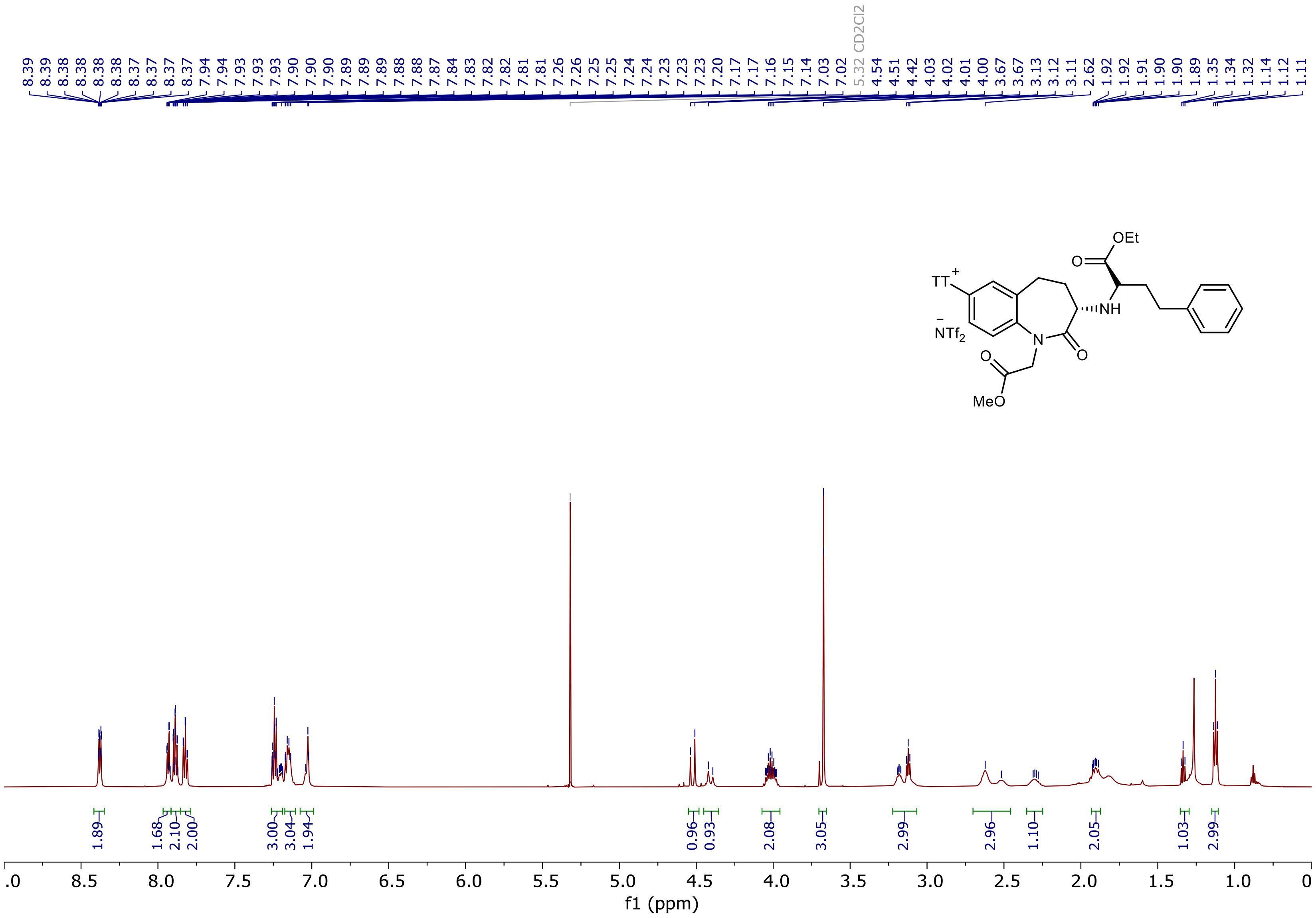

^{13}C NMR of benazepril methyl ester-derived thianthrenium salt (30-TT)

CD_2Cl_2, 23 °C

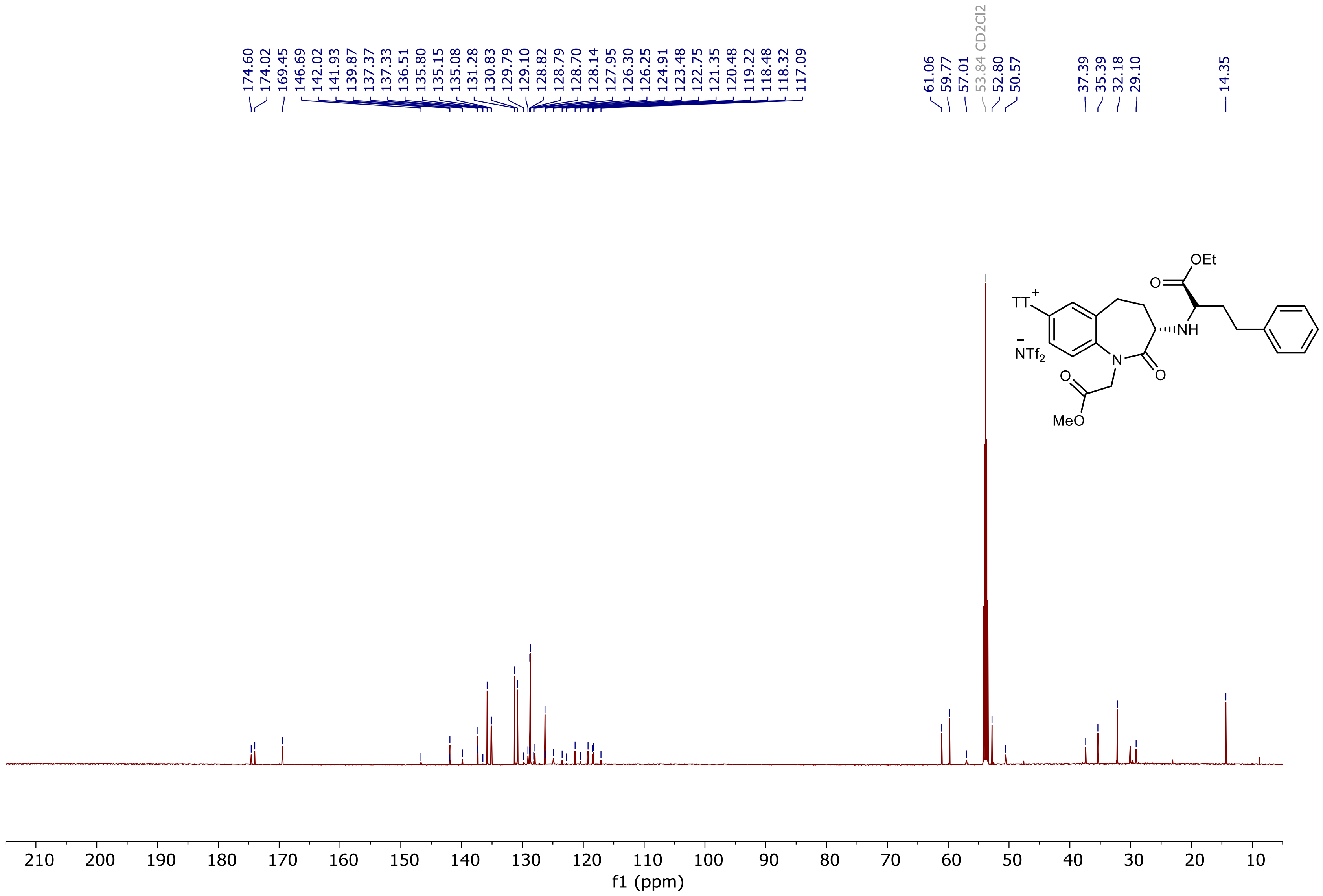

^{19}F NMR of benazepril methyl ester-derived thianthrenium salt (30-TT)

CD_2Cl_2, 23 °C

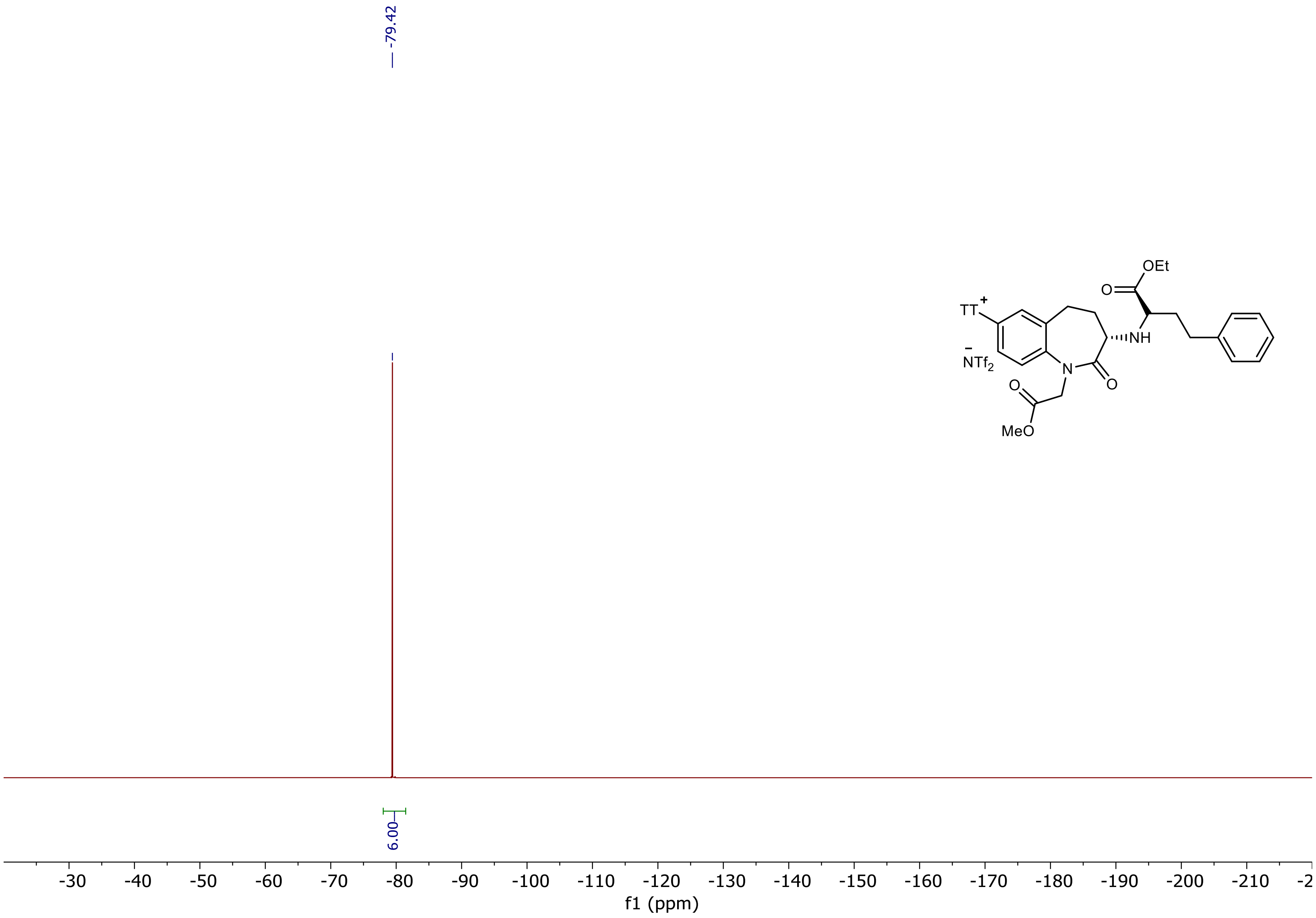

NOESY spectrum of benazepril methyl ester-derived thianthrenium salt (30-TT)

CD_2Cl_2, 23 °C

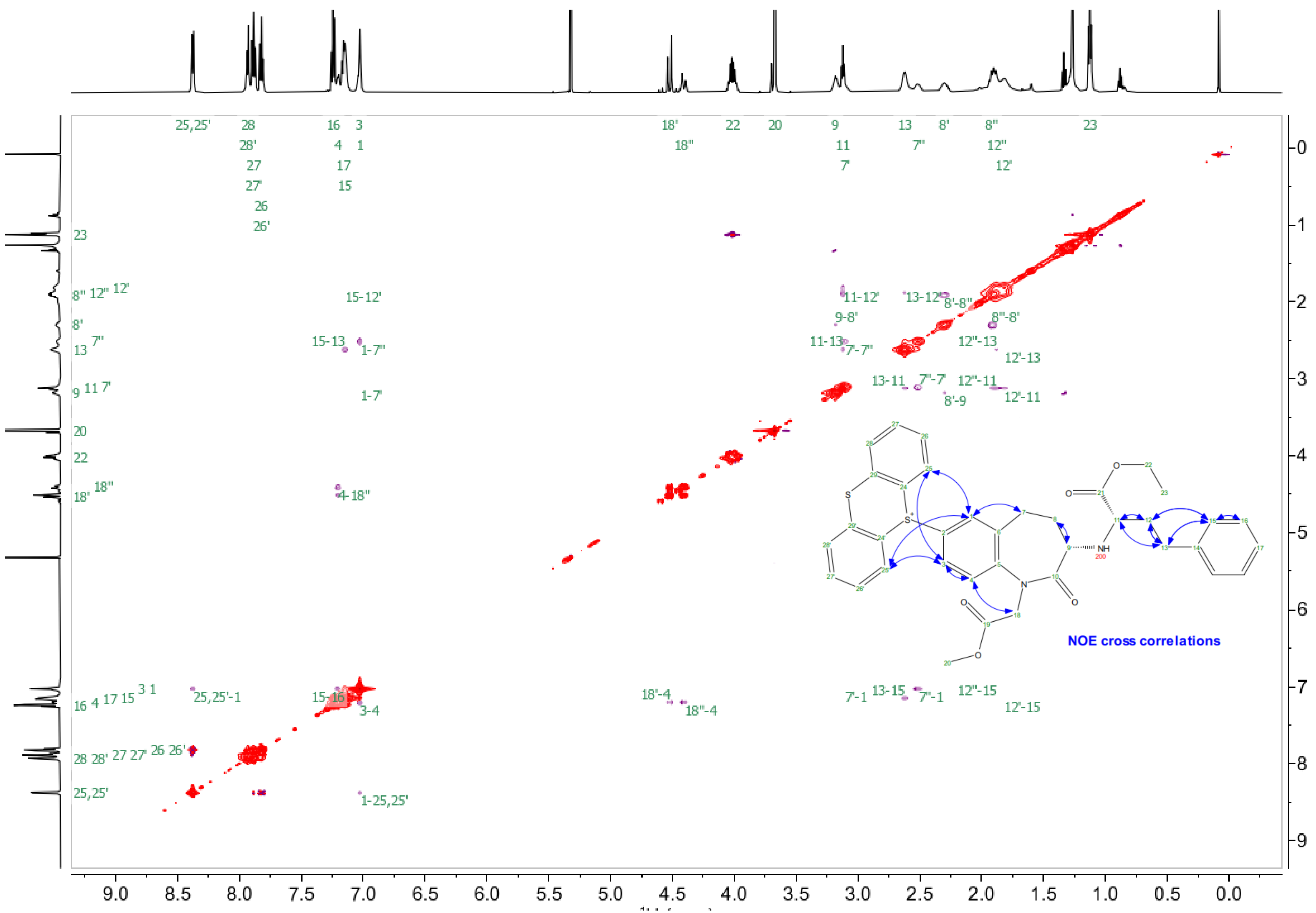

^{1}H NMR of benazepril methyl ester-derived thianthrenium salt (30-TT1)

CD_2Cl_2, 23 °C

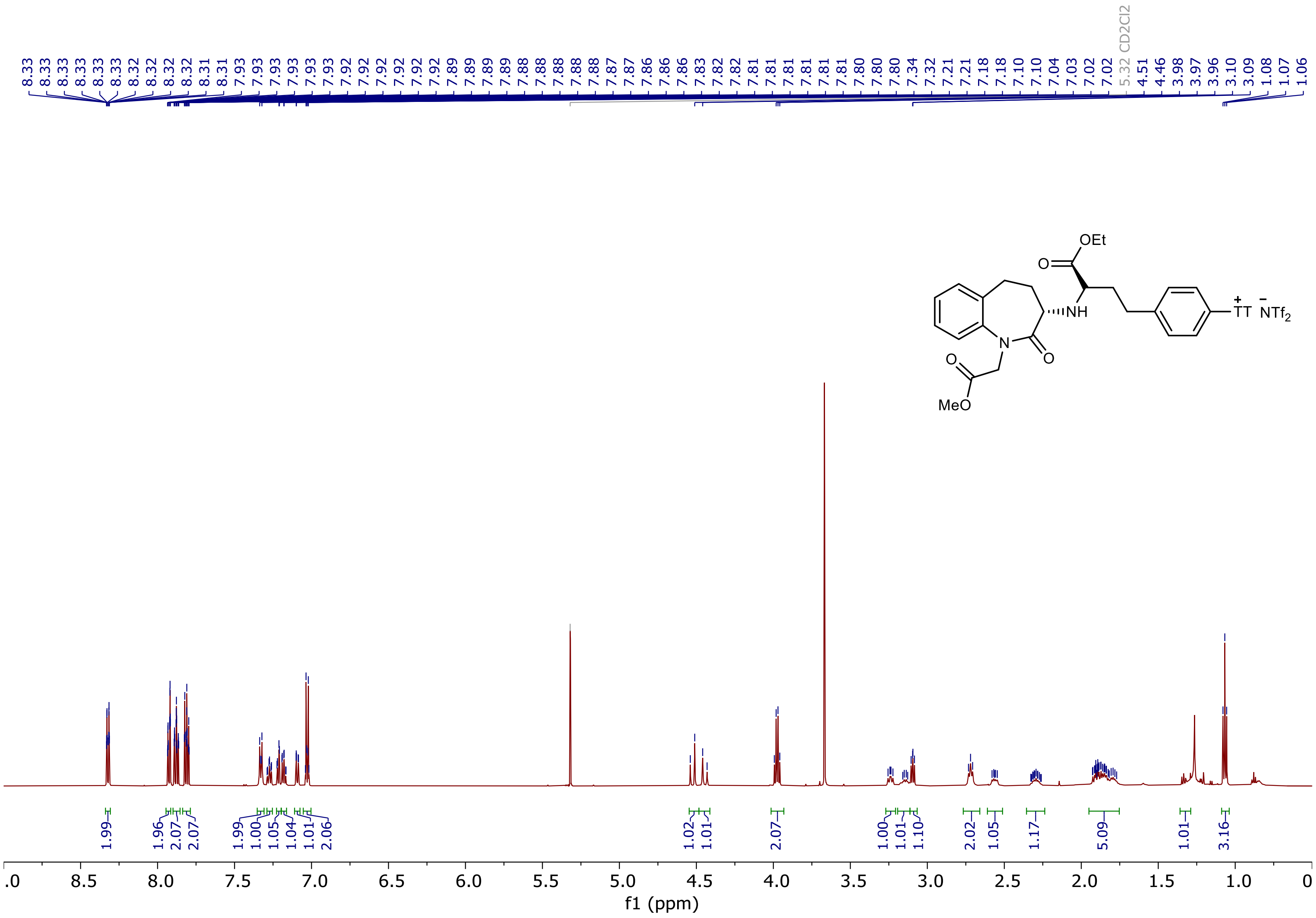

^{13}C NMR of benazepril methyl ester-derived thianthrenium salt (30-TT1)

CD_2Cl_2, 23 °C

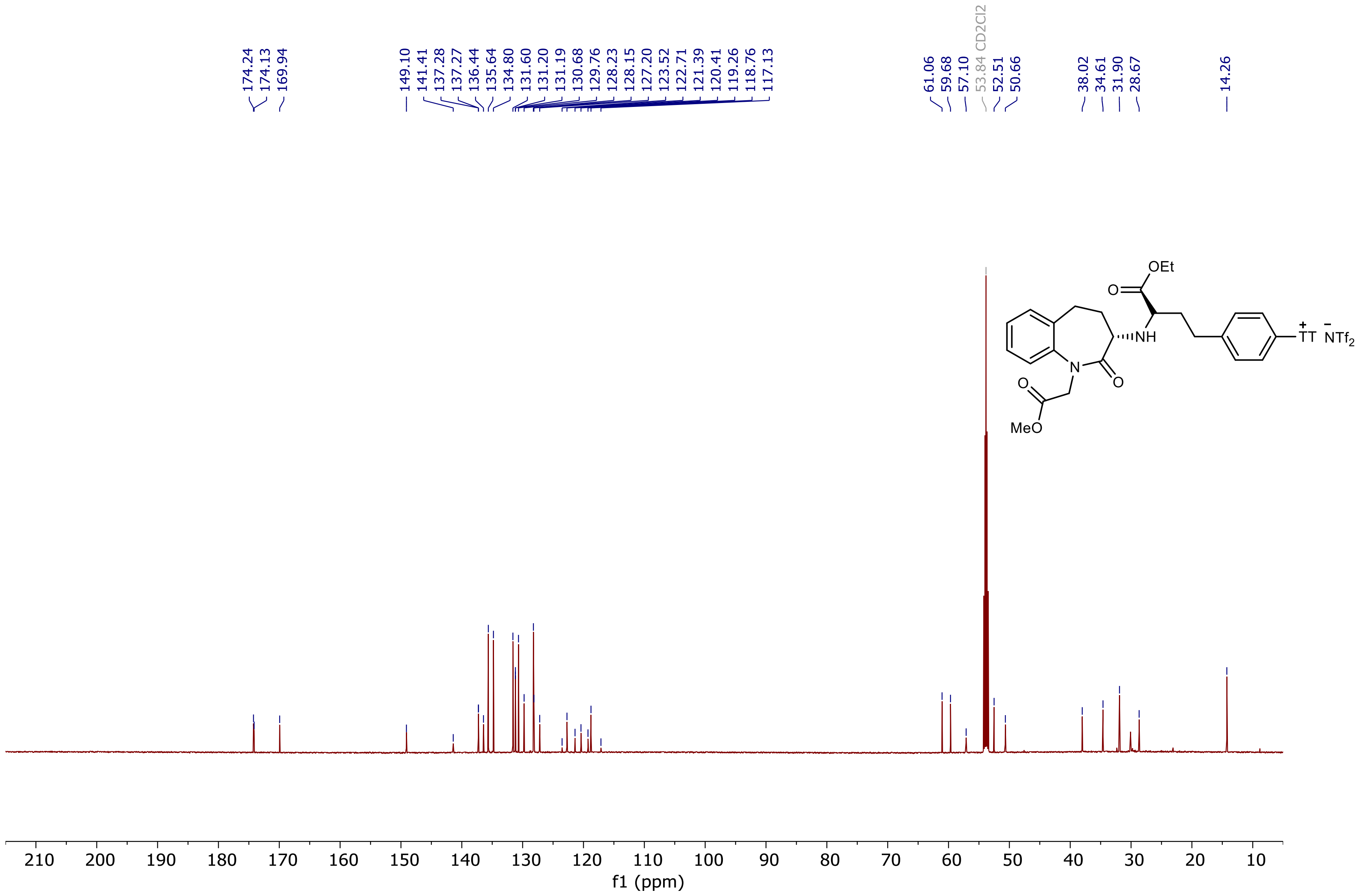

^{19}F NMR of benazepril methyl ester-derived thianthrenium salt (30-TT1)

CD_2Cl_2, 23 °C

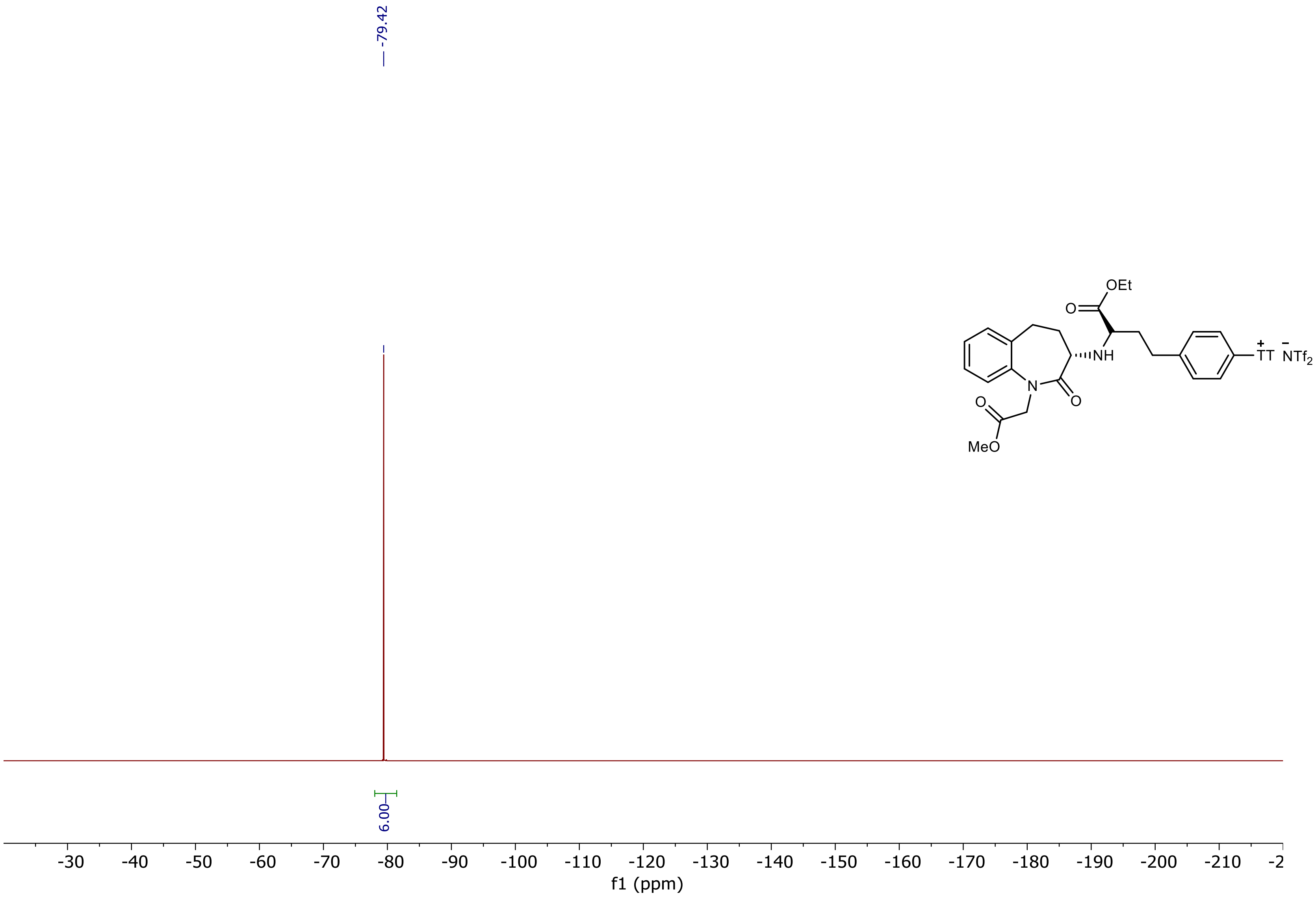

NOESY spectrum of benazepril methyl ester-derived thianthrenium salt (30-TT1)

CD_2Cl_2, 23 °C

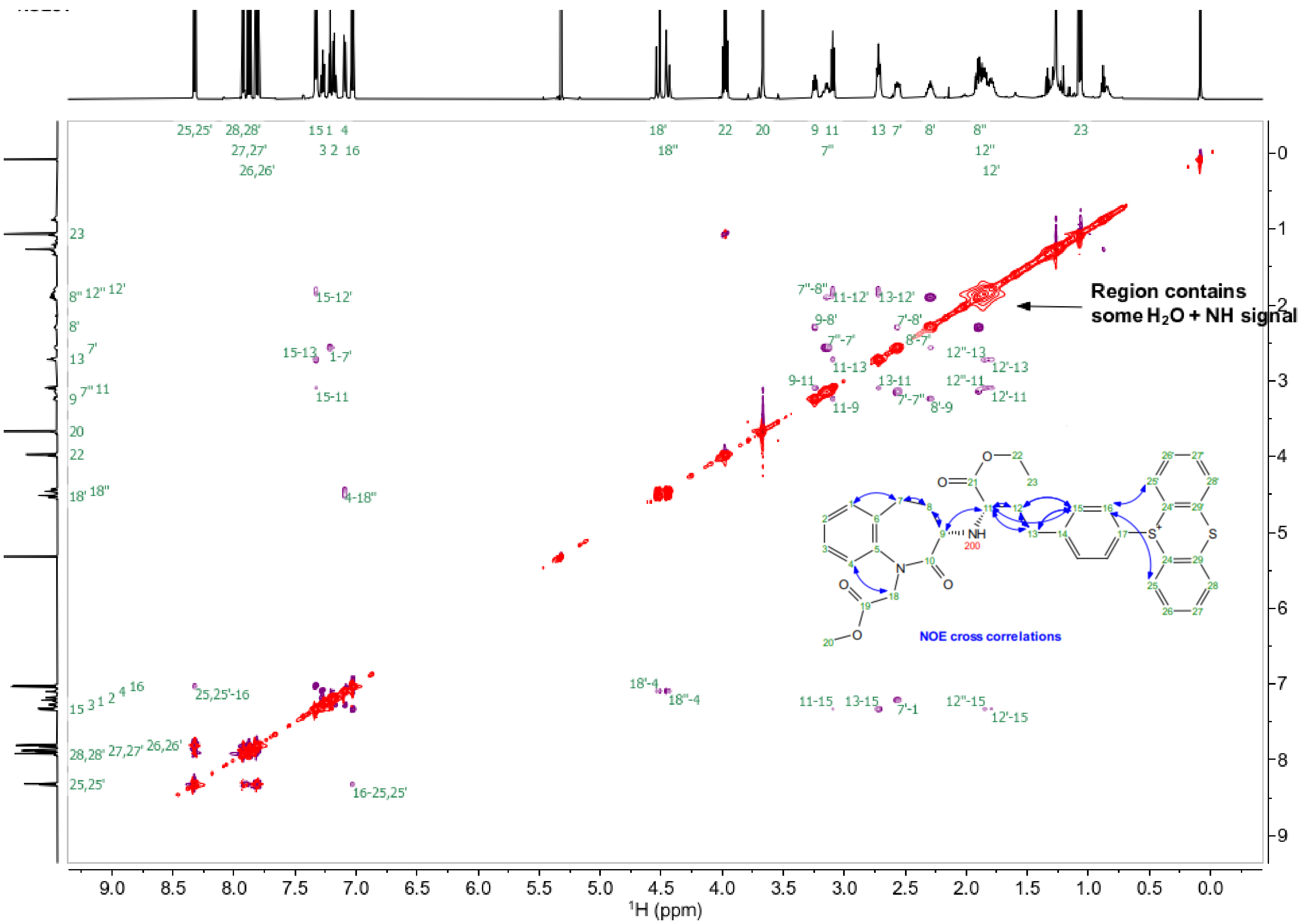

^{1}H NMR of 4-[^{2}H]-biphenyl ([^{2}H]1)

CD_2Cl_2, 23 °C

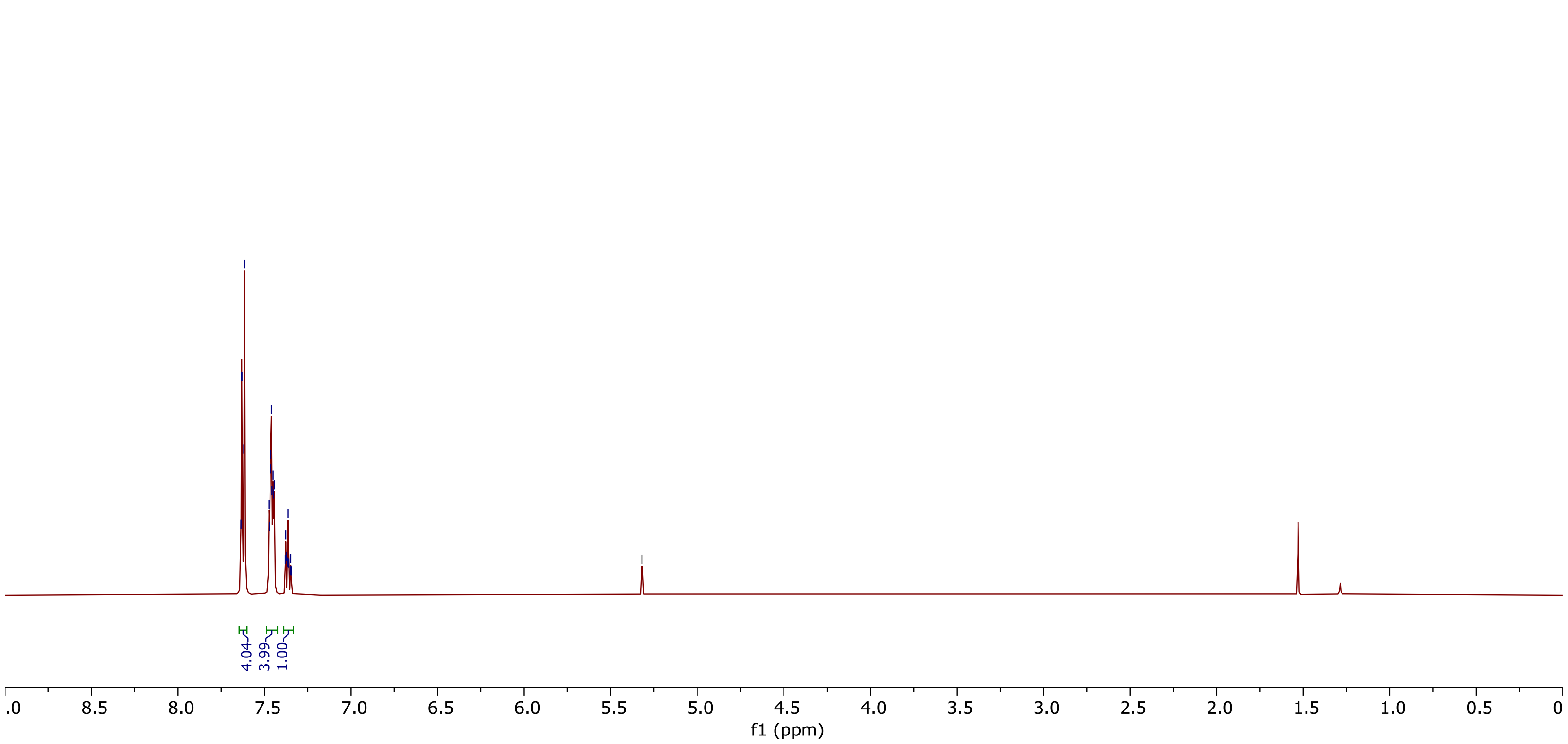

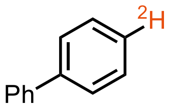

^{2}H NMR of 4-[^{2}H]-biphenyl ([^{2}H]1)

CH_2Cl_2, 23 °C

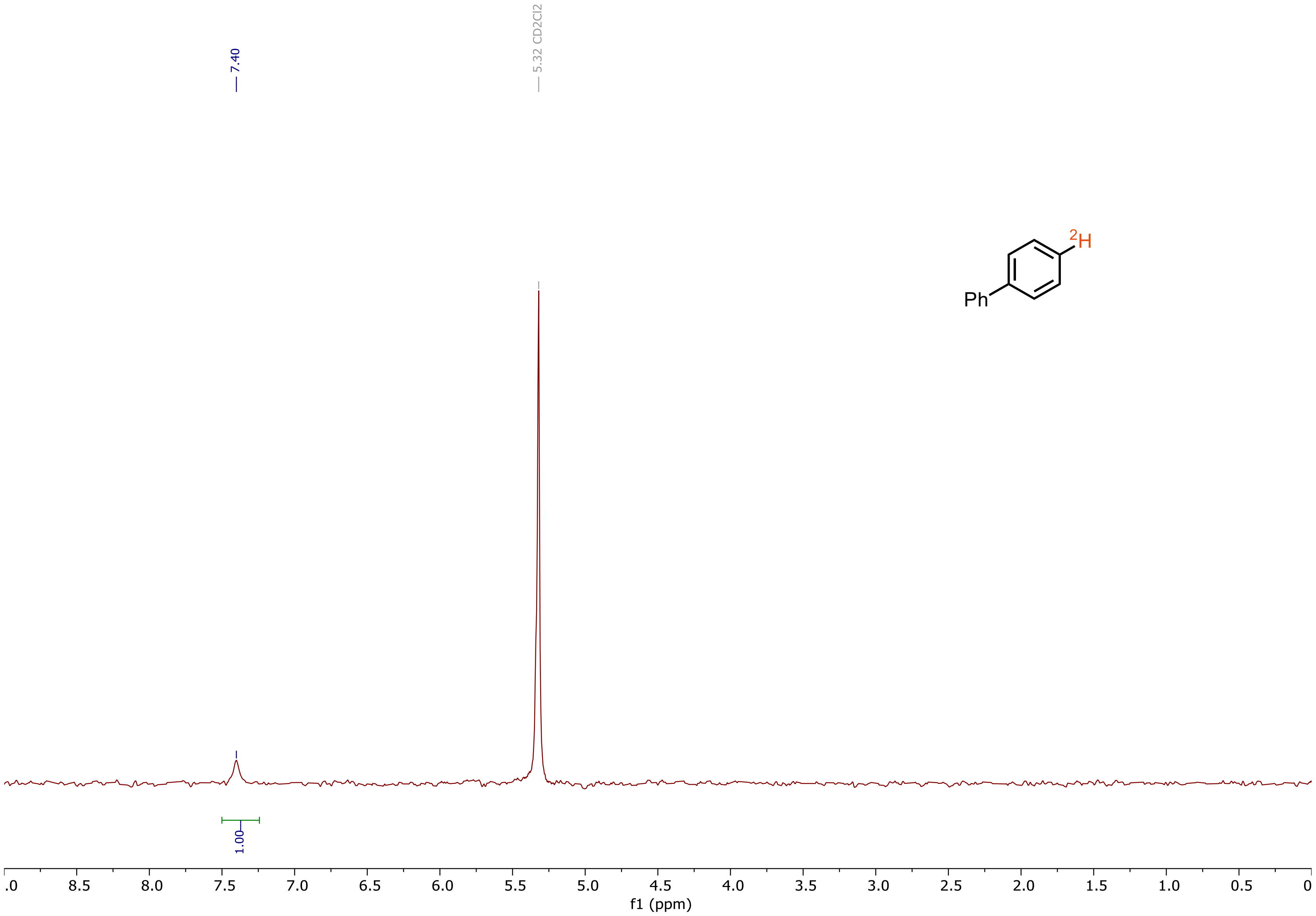

^{13}C NMR of 4-[^{2}H]-biphenyl ([^{2}H]1)

CD_2Cl_2, 23 °C

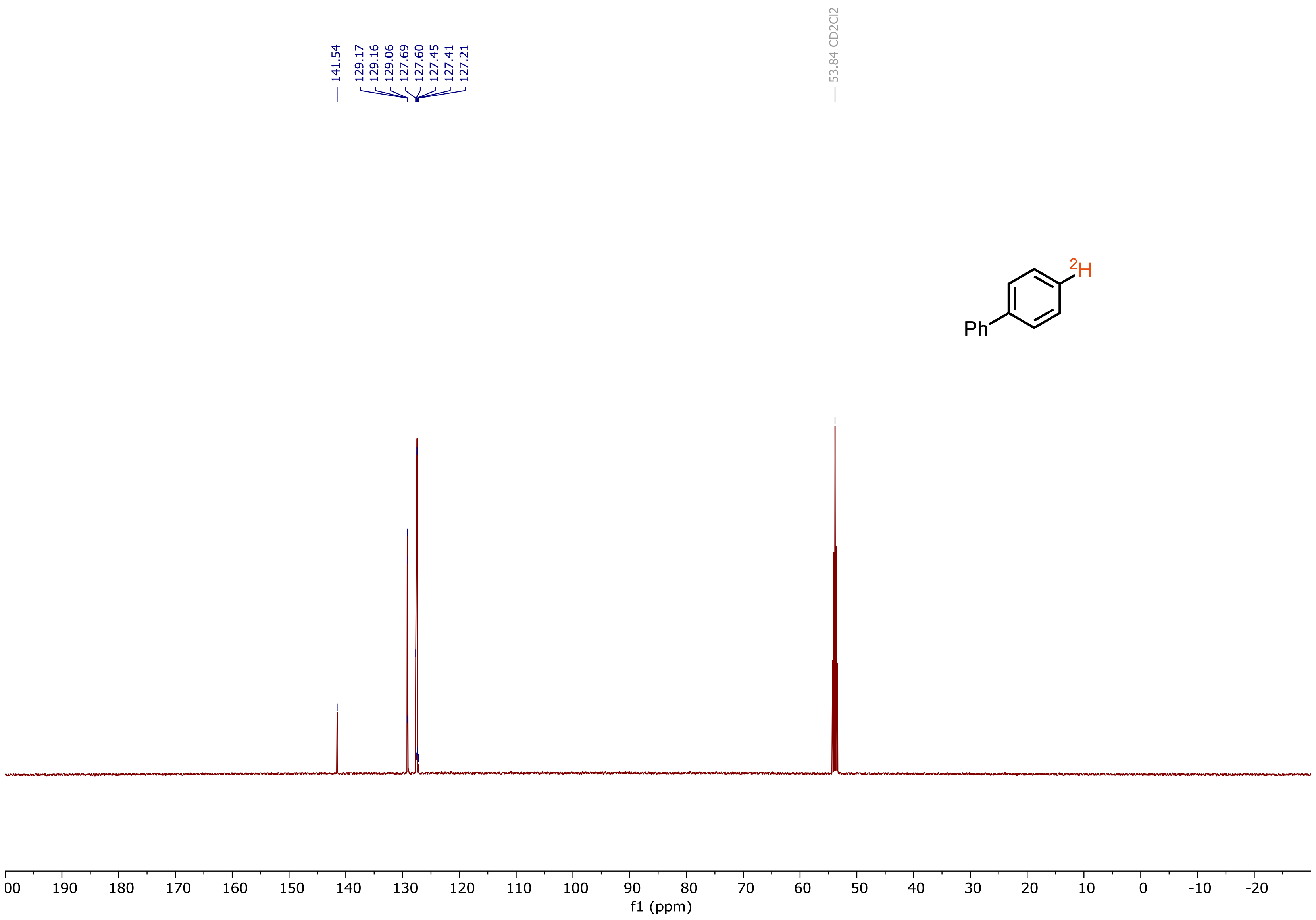

^{1}H NMR of 2-fluoro-(*p*-[^{2}H]phenoxy)benzonitrile ([^{2}H]3)

CD_3CN, 23 °C

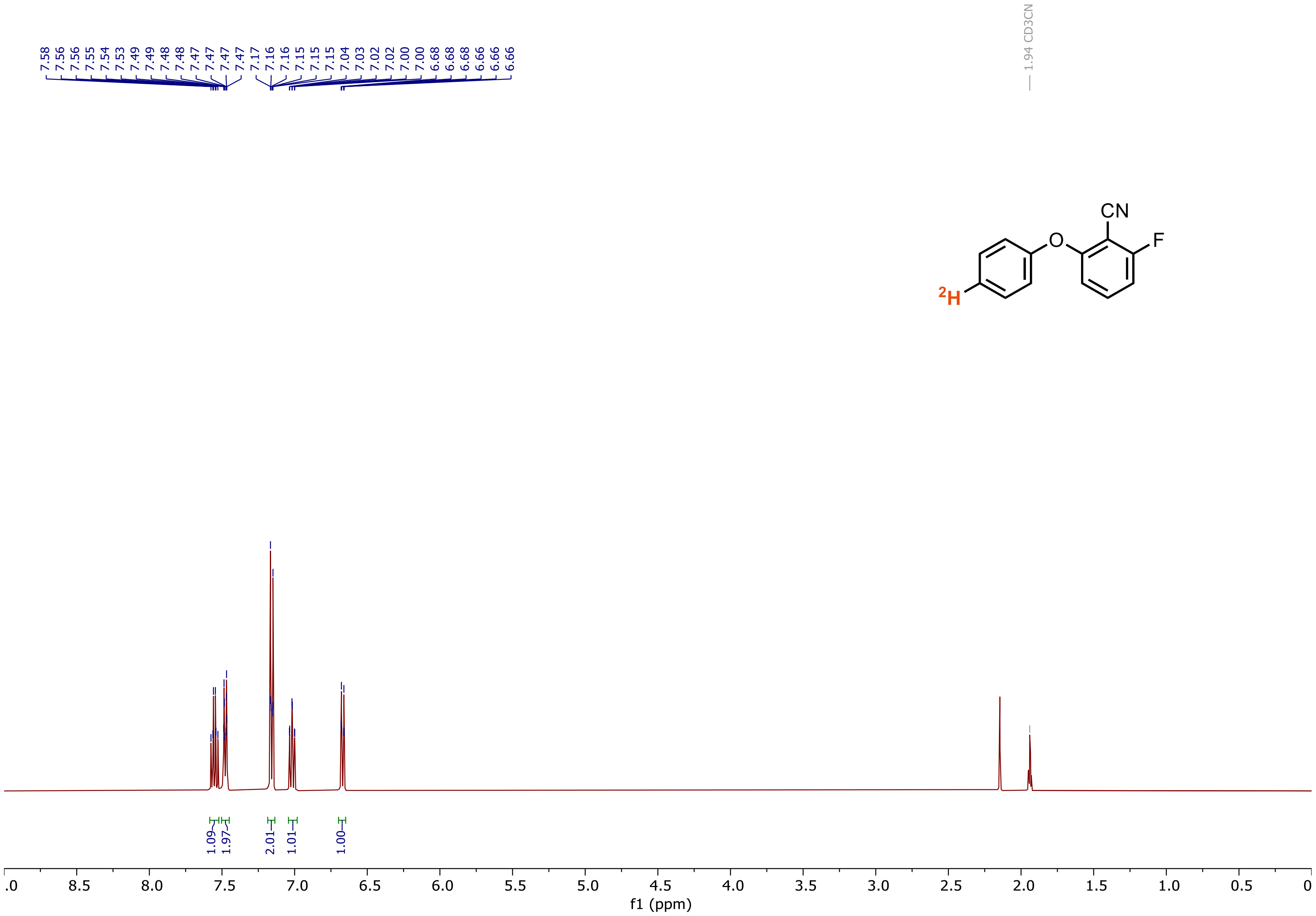

^{2}H NMR of 2-fluoro-(*p*-[^{2}H]phenoxy)benzonitrile ([^{2}H]3)

CH_3CN, 23 °C

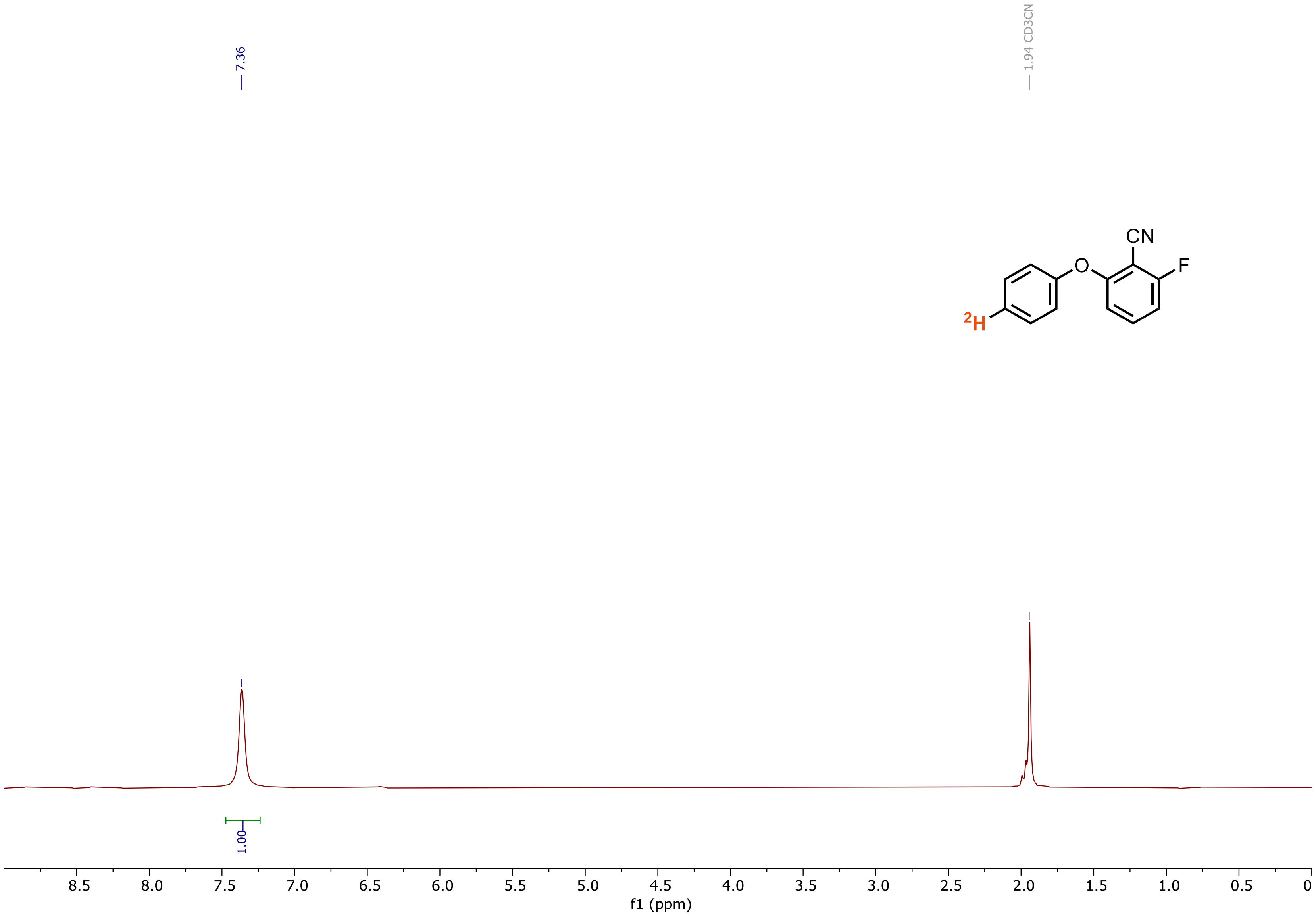

^{13}C NMR of 2-fluoro-(*p*-[^{2}H]phenoxy)benzonitrile ([^{2}H]3)

CD_3CN, 23 °C

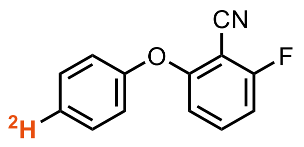

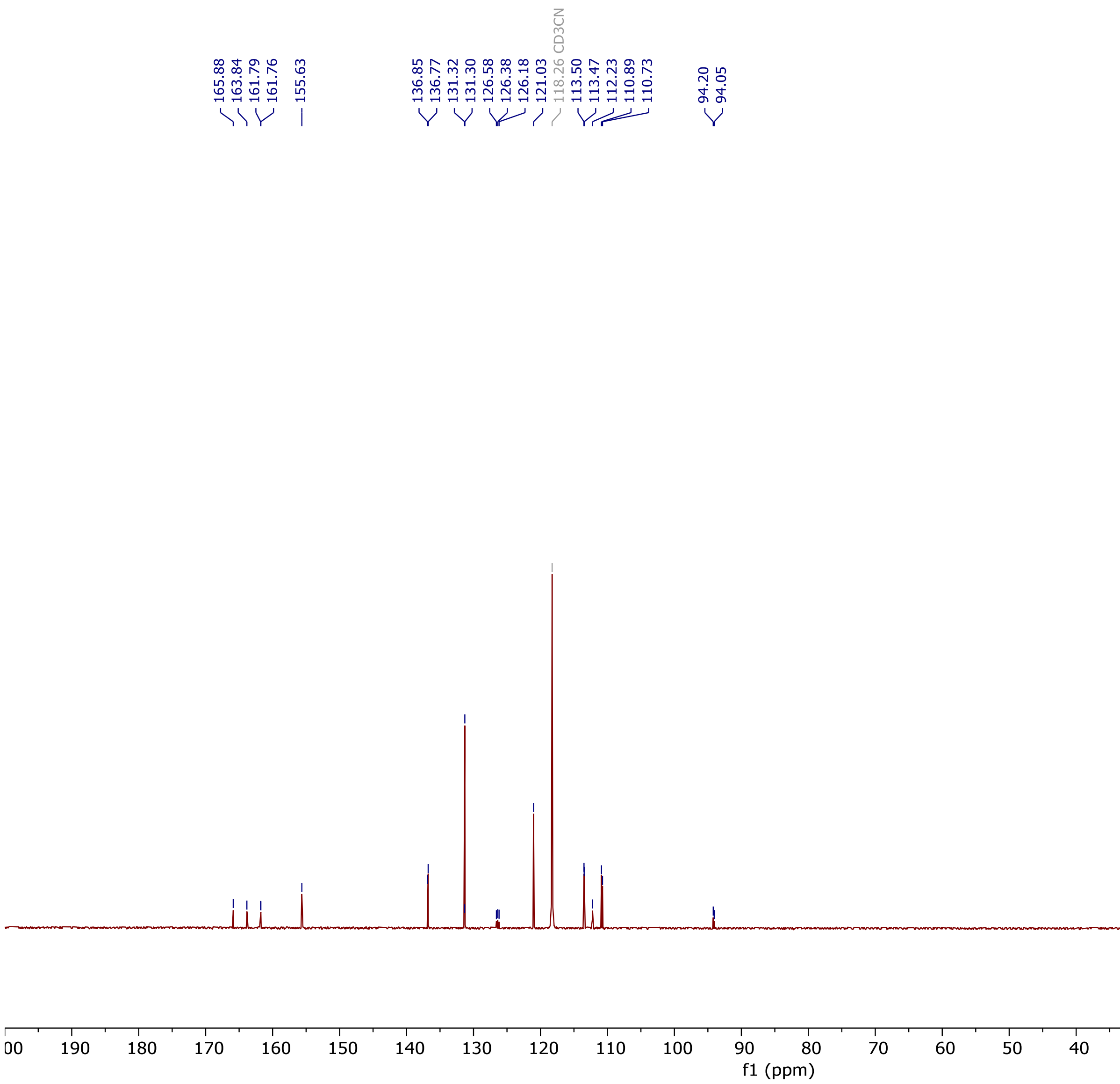

^{19}F NMR of 2-fluoro-(*p*-[^{2}H]phenoxy)benzonitrile ([^{2}H]3)

CD_3CN, 23 °C

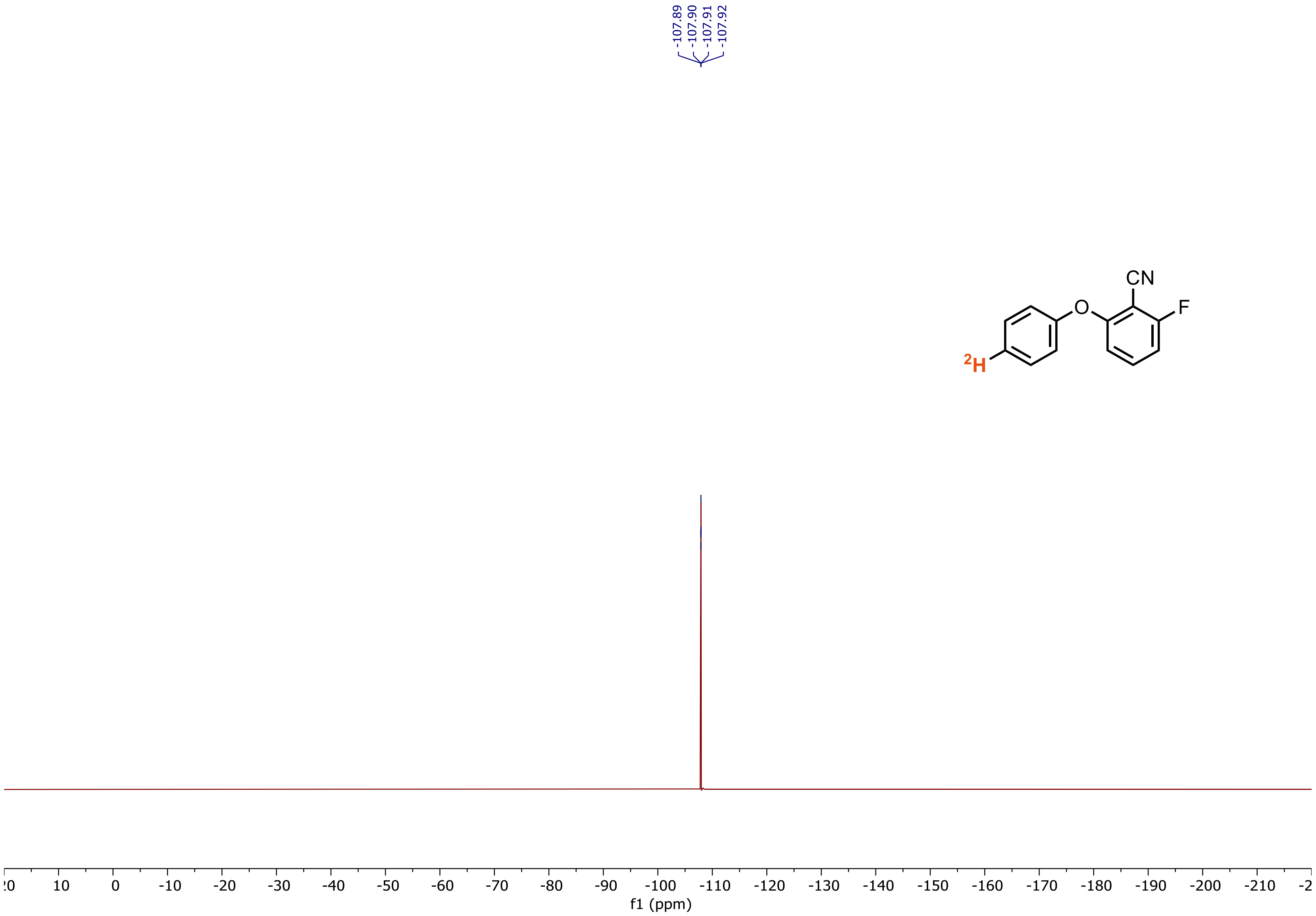

^{1}H NMR of 3-phenyl-3-(4-[^{2}H]-phenyl) propan-1-ol ([^{2}H]4)

CD_2Cl_2, 23 °C

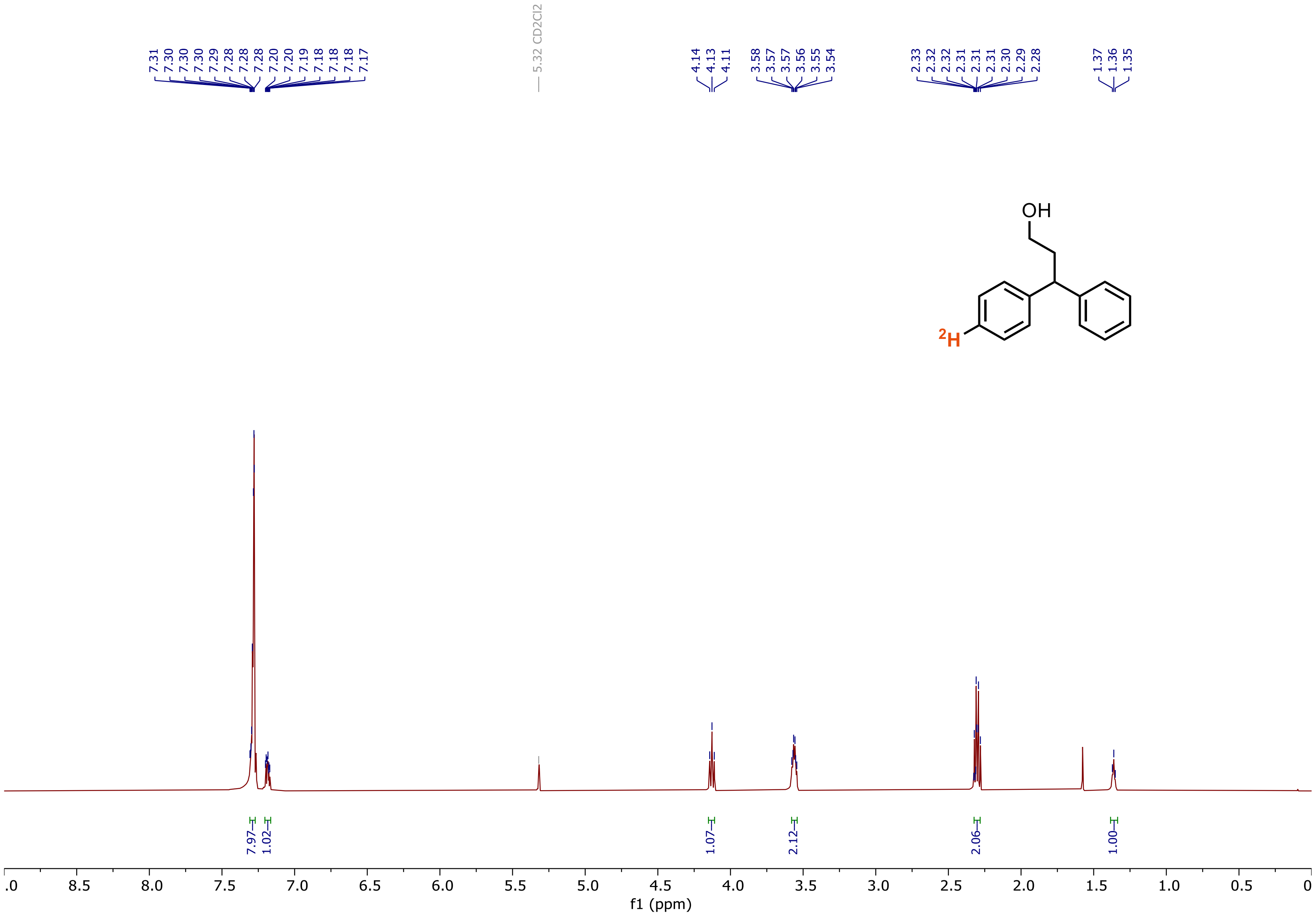

^{2}H NMR of 3-phenyl-3-(4-[^{2}H]-phenyl) propan-1-ol ([^{2}H]4)

CH_2Cl_2, 23 °C

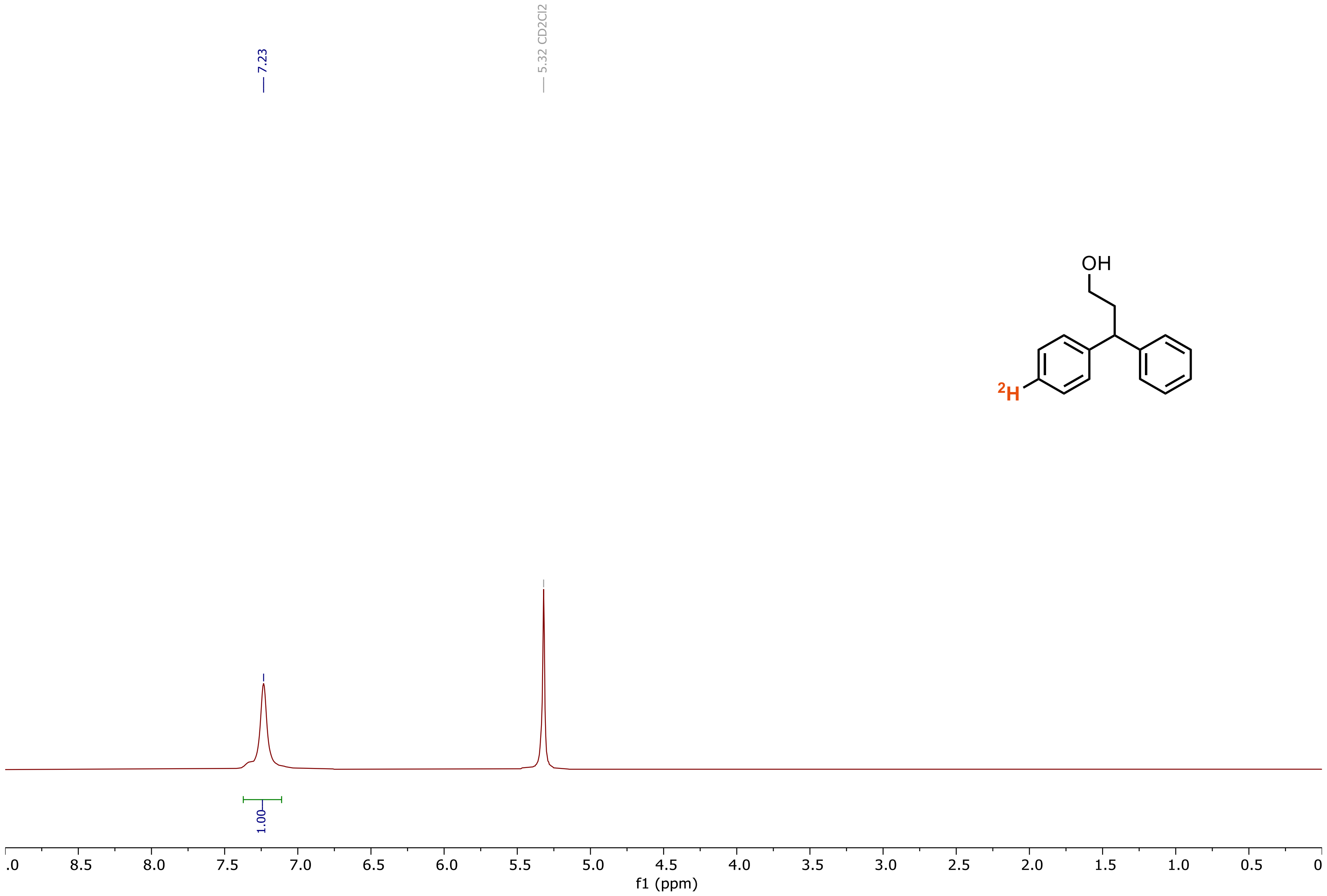

^{13}C NMR of 3-phenyl-3-(4-[^{2}H]-phenyl) propan-1-ol ([^{2}H]4)

CD_2Cl_2, 23 °C

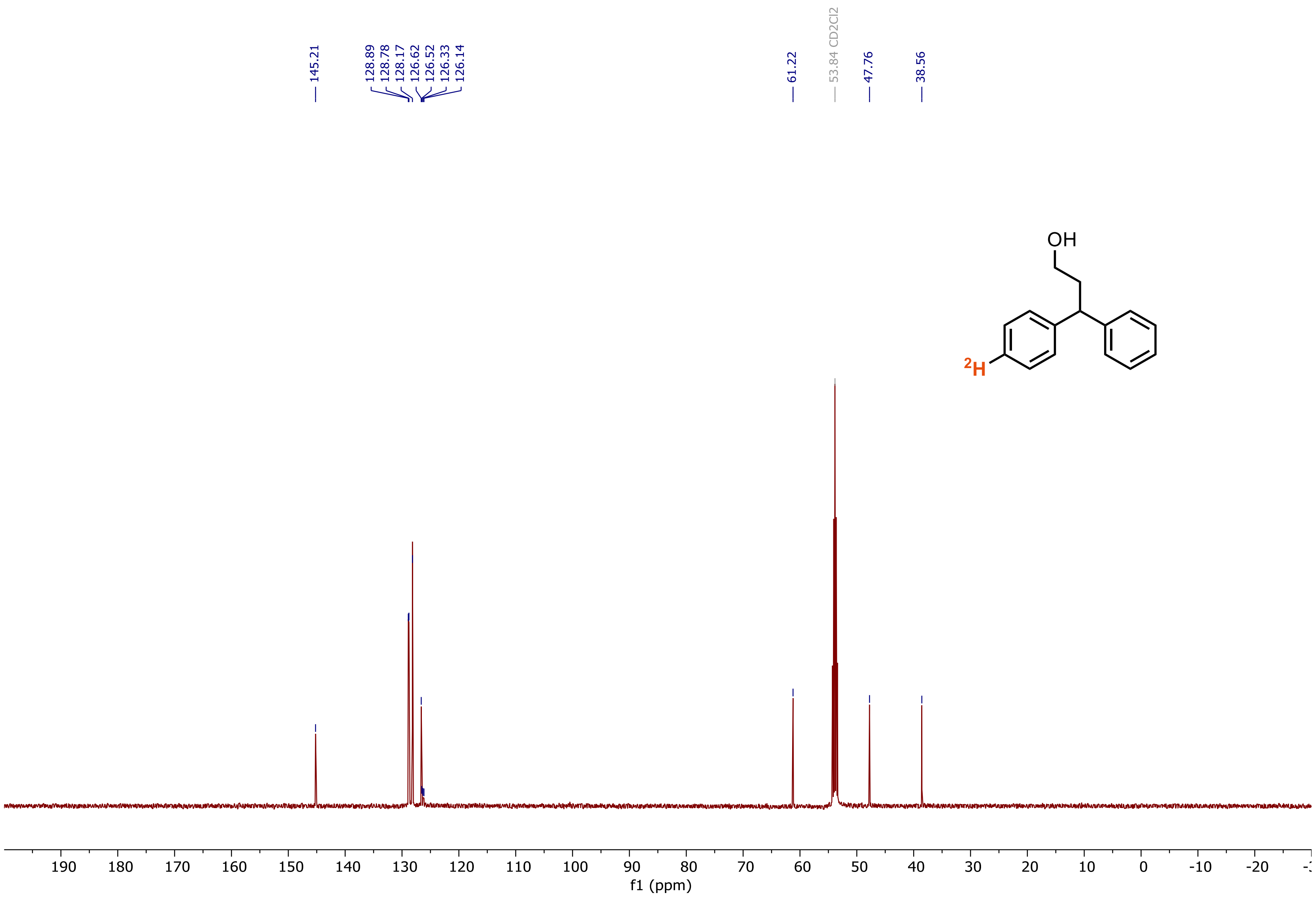

^{1}H NMR of 4-[^{2}H]-benzyloxazolidinone ([^{2}H]5)

CD_2Cl_2, 23 °C

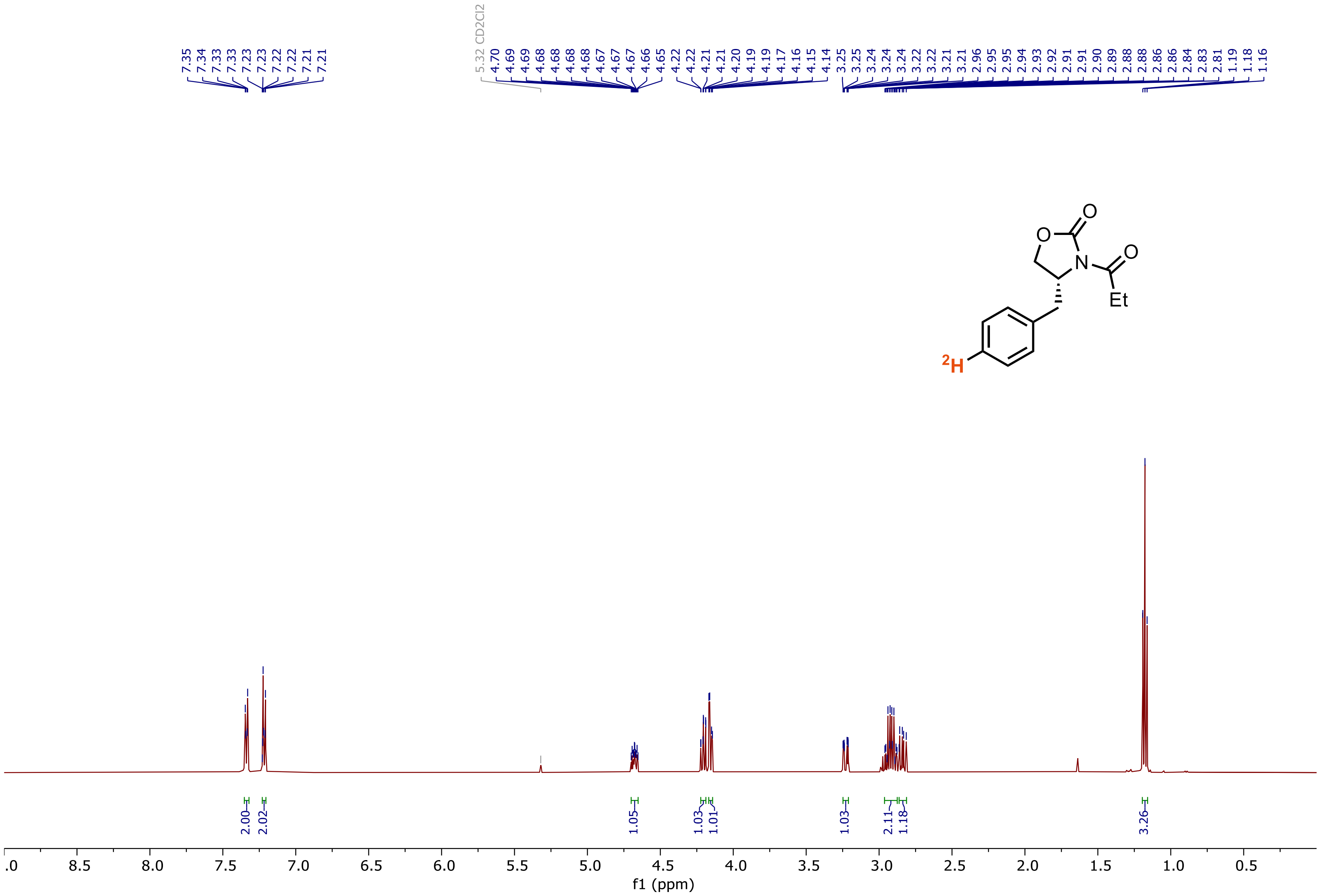

^{2}H NMR of 4-[^{2}H]-benzyloxazolidinone ([^{2}H]5)

CH_2Cl_2, 23 °C

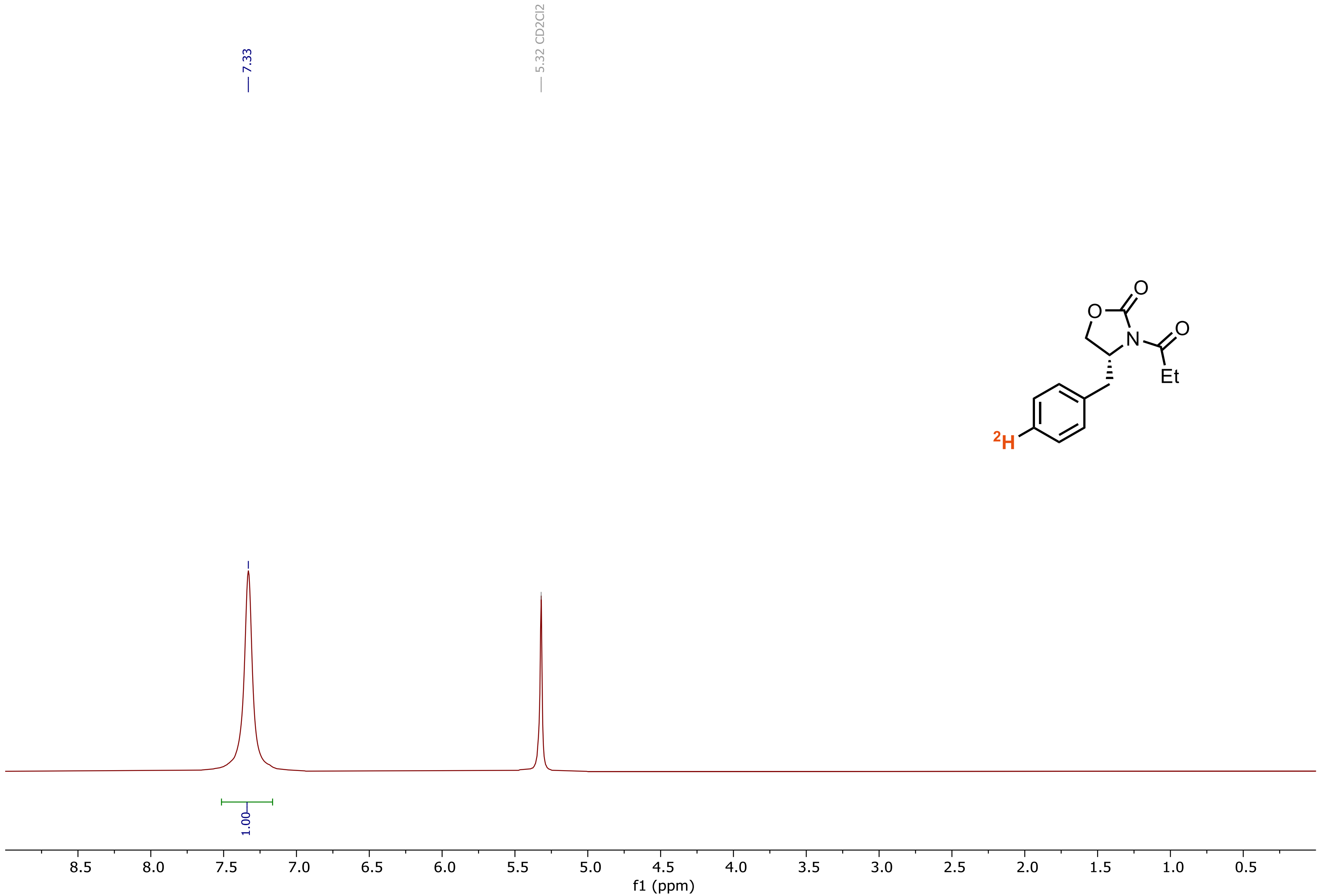

^{13}C NMR of 4-[^{2}H]-benzyloxazolidinone ([^{2}H]5)

CD_2Cl_2, 23 °C

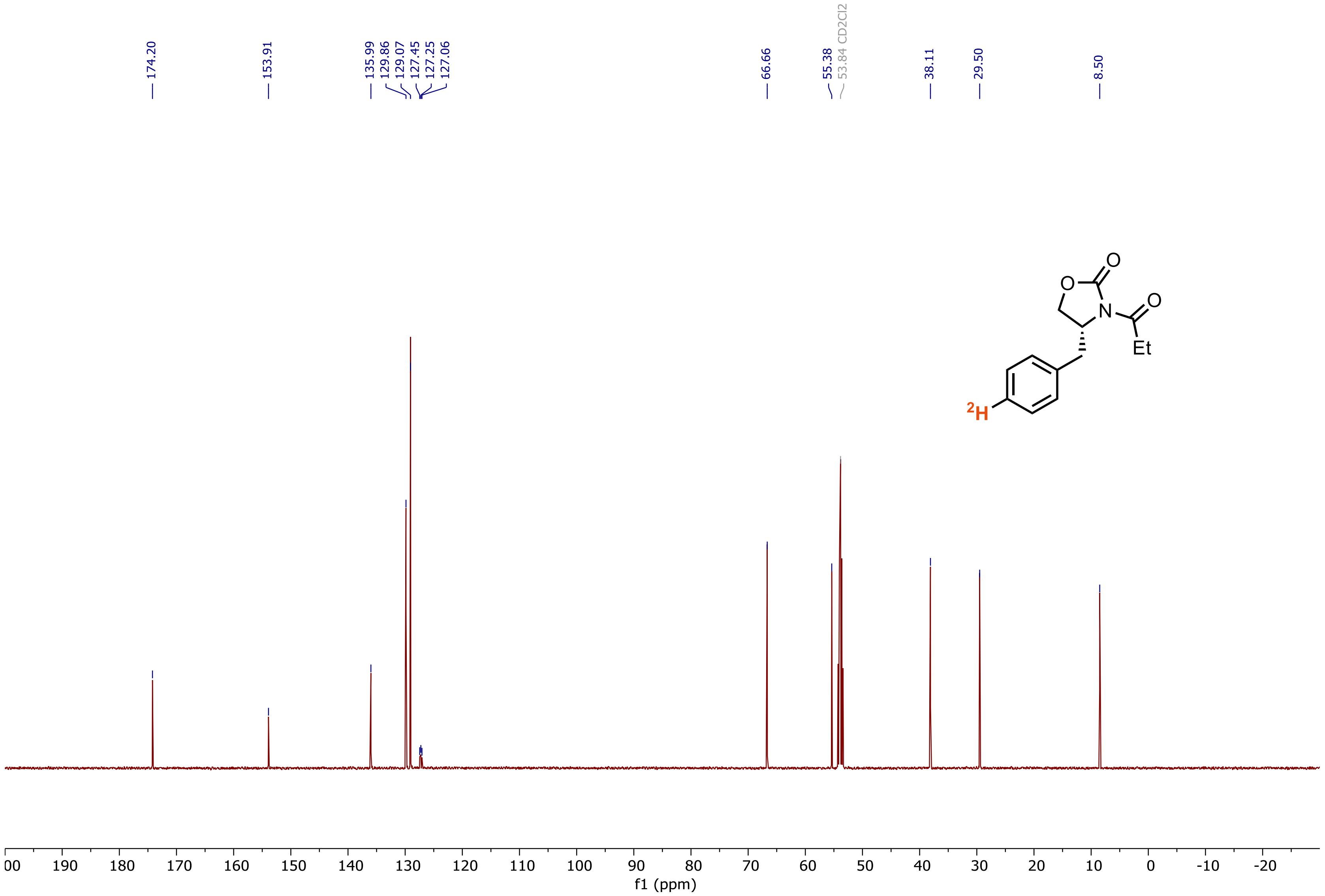

^1H NMR of [^2H]amiodarone ([^2H]6)

CD_2Cl_2, 23 °C

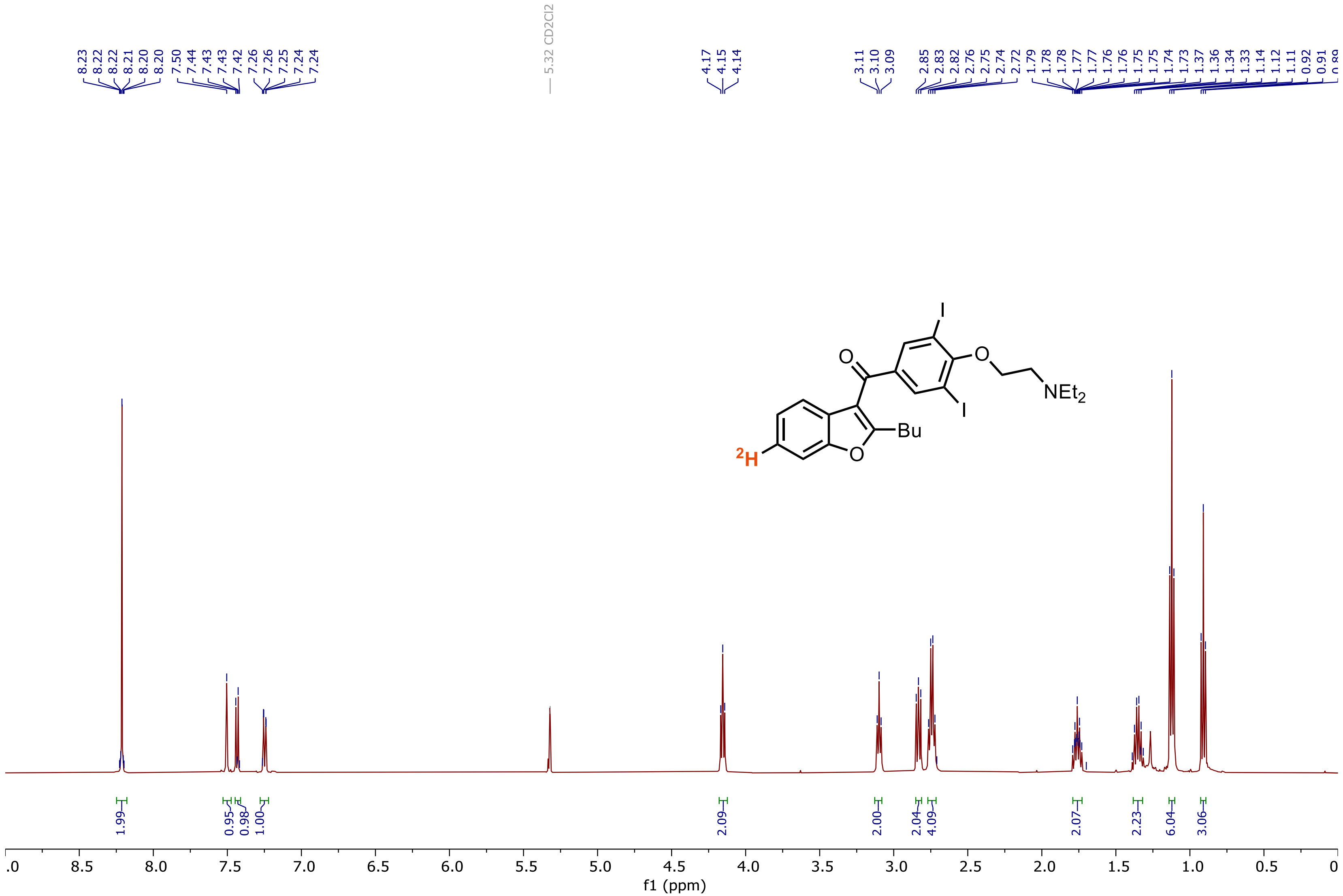

^{2}H NMR of [^{2}H]amiodarone ([^{2}H]6)

CH_2Cl_2, 23 °C

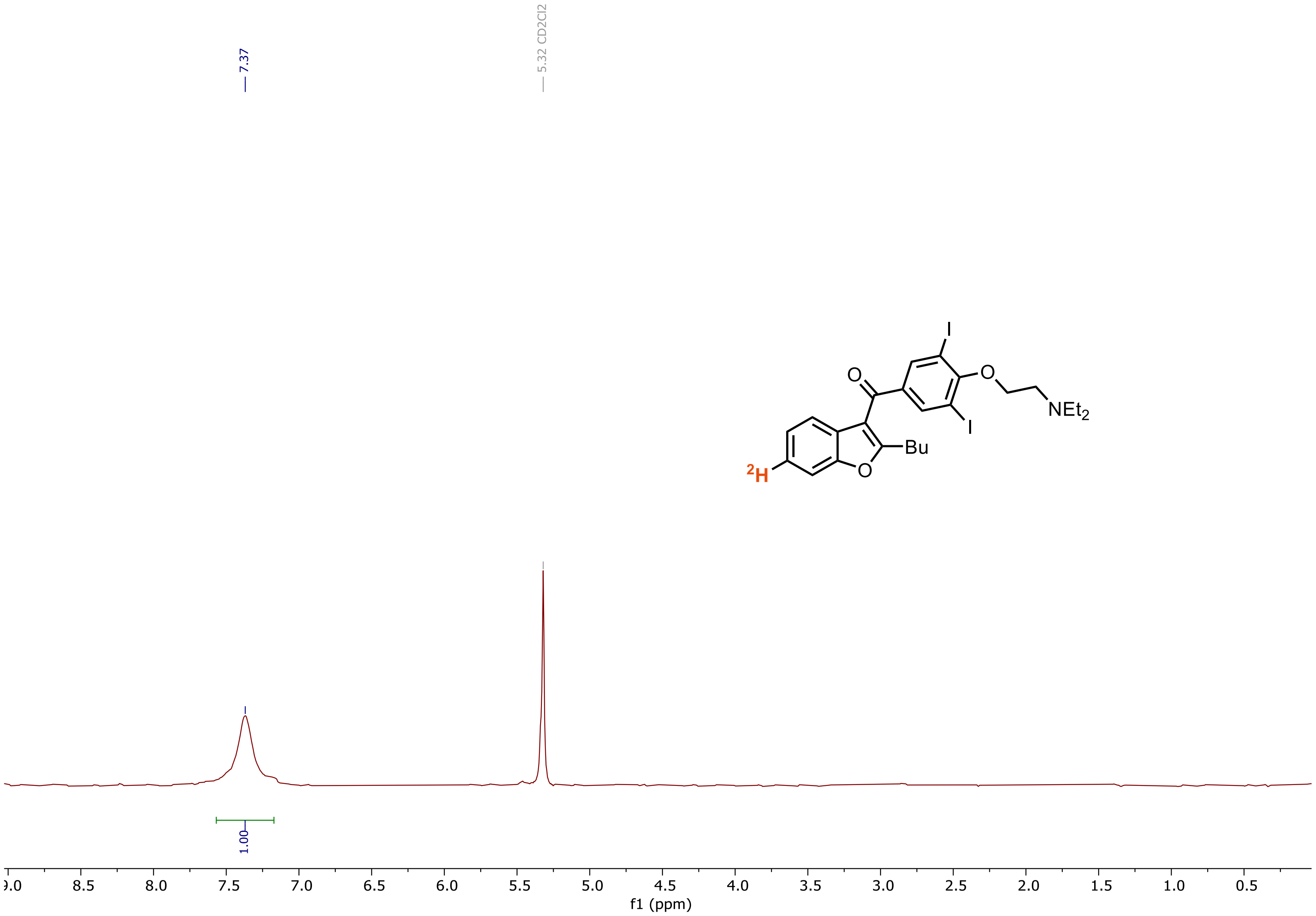

^{13}C NMR of [^{2}H]amiodarone ([^{2}H]6)

CD_2Cl_2, 23 °C

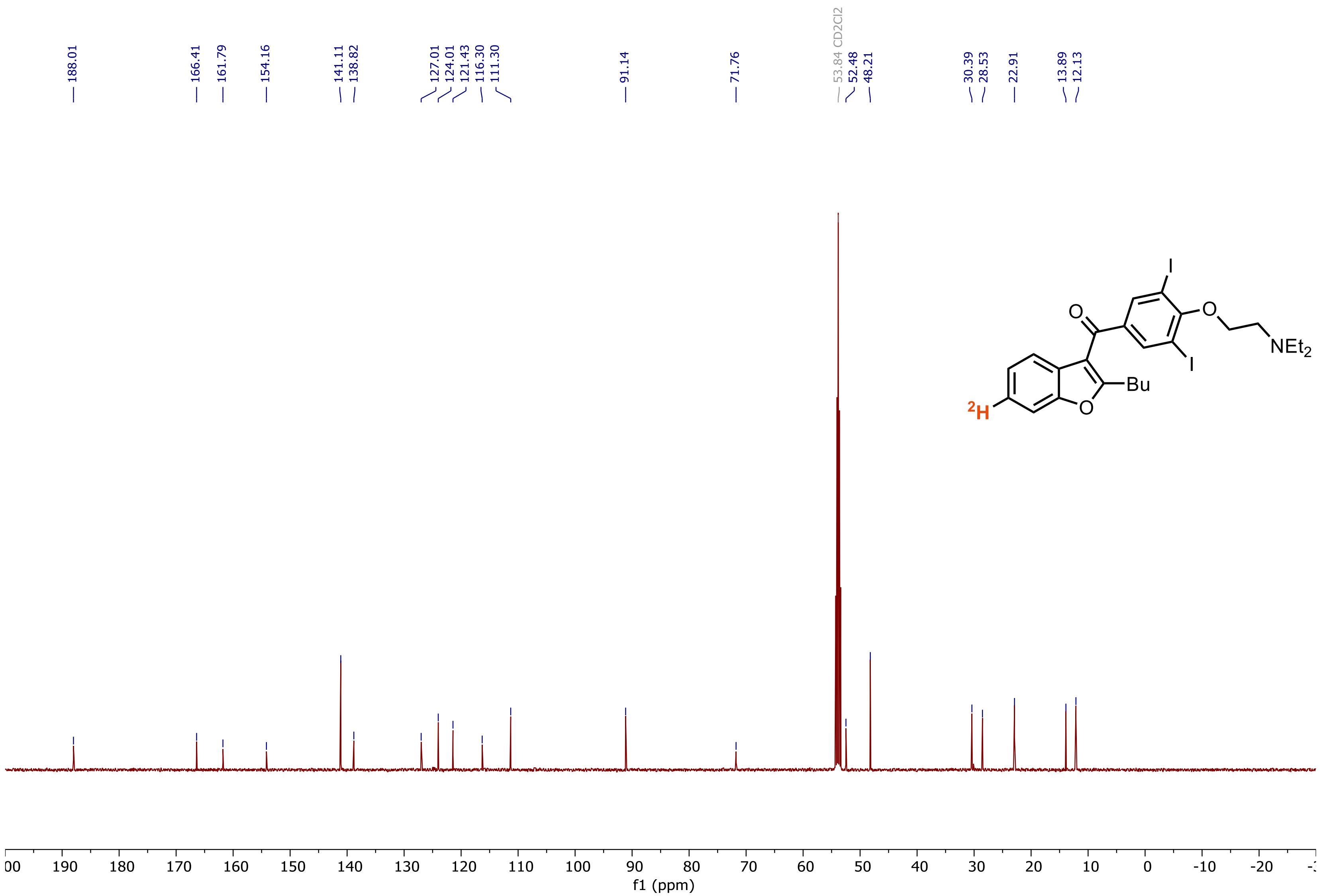

^{1}H NMR of 1-(4-bromophenoxy)-4-[^{2}H]-benzene ([^{2}H]8)

CD_3CN, 23 °C

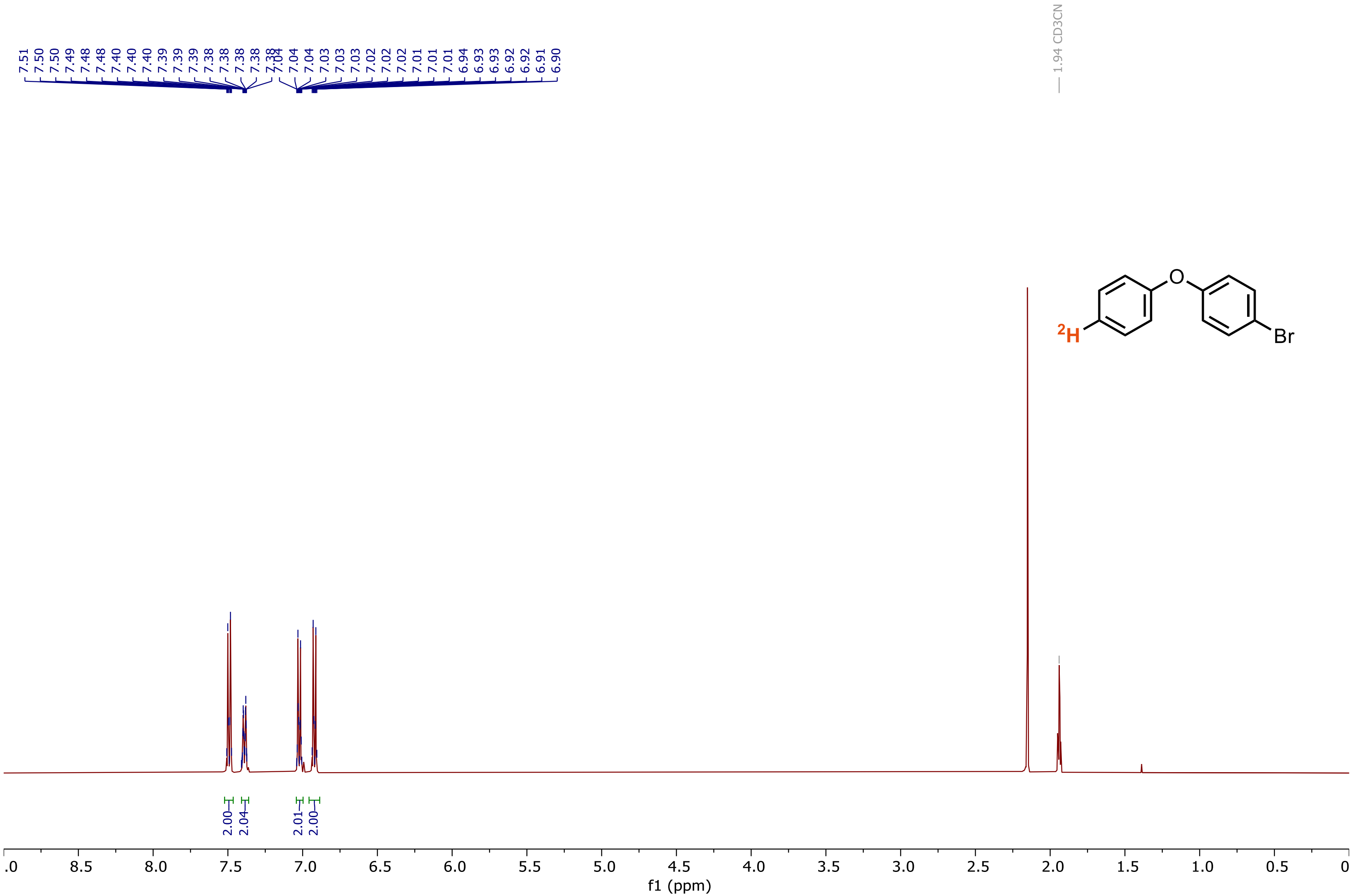

^{2}H NMR of 1-(4-bromophenoxy)-4-[^{2}H]-benzene ([^{2}H]8)

CH_3CN, 23 °C

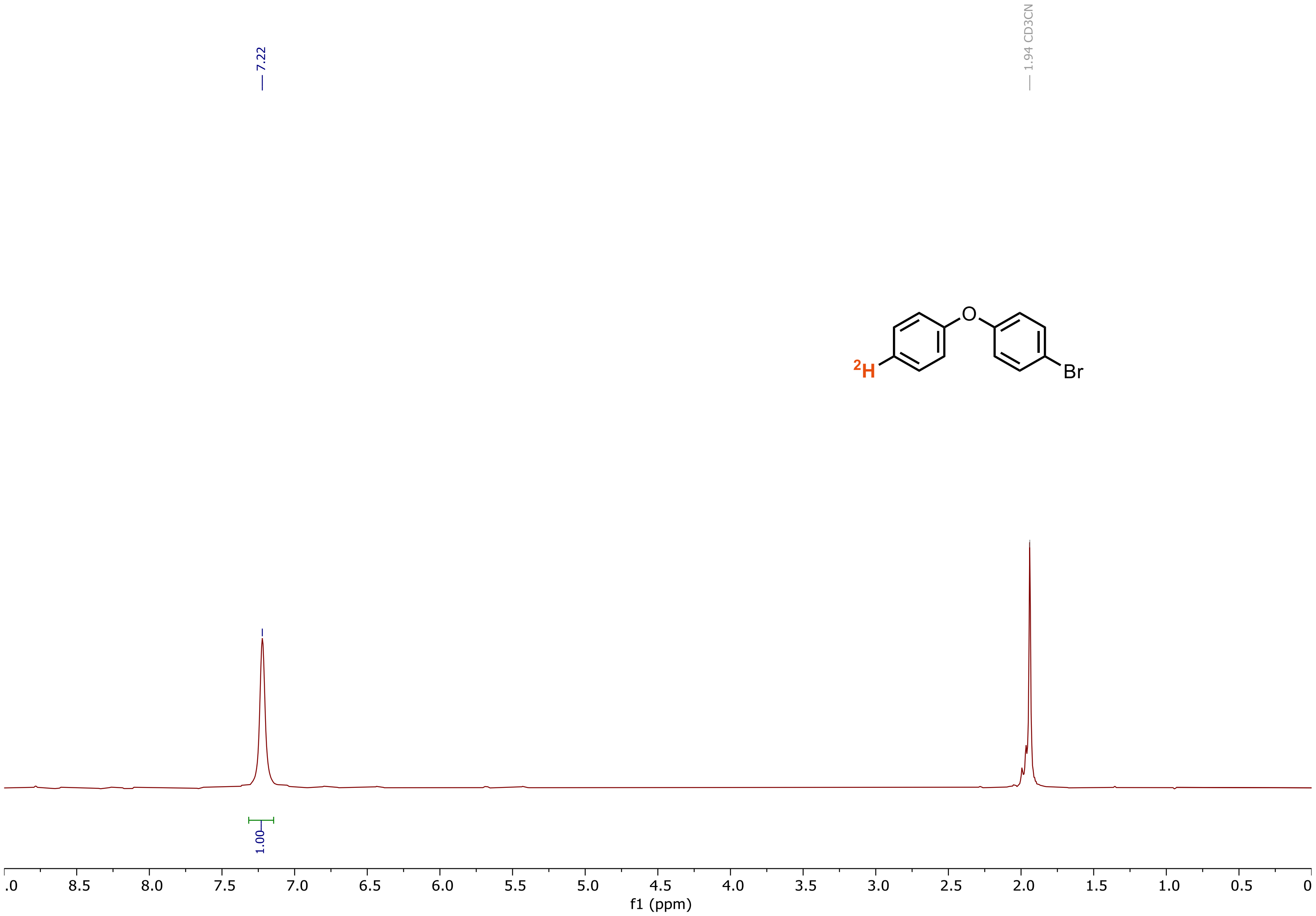

^{13}C NMR of 1-(4-bromophenoxy)-4-[^{2}H]-benzene ([^{2}H]8)

CD_3CN, 23 °C

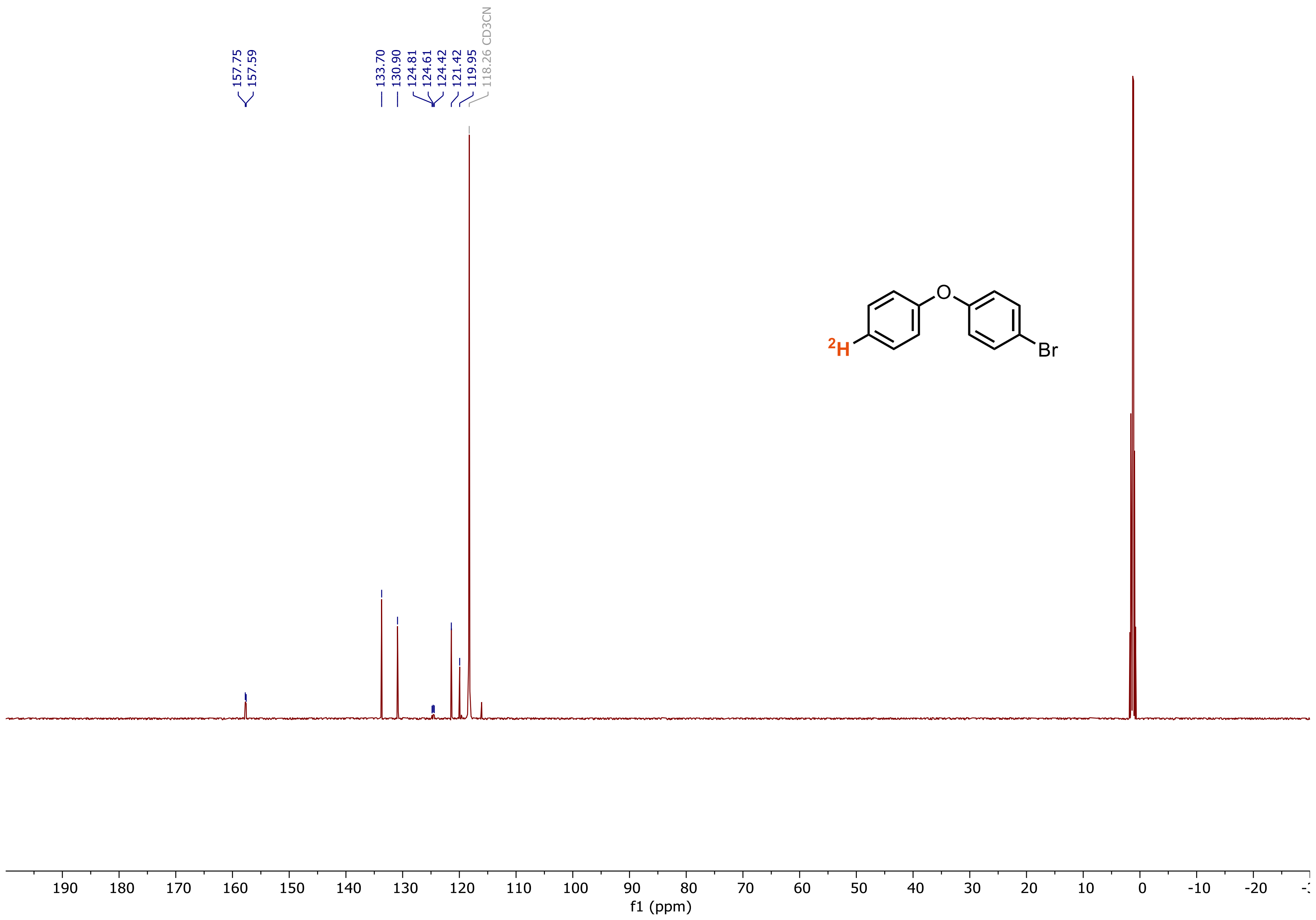

^{1}H NMR of *N*-(4-[^{2}H]-phenyl)benzamide ([^{2}H]9)

CD_2Cl_2, 23 °C

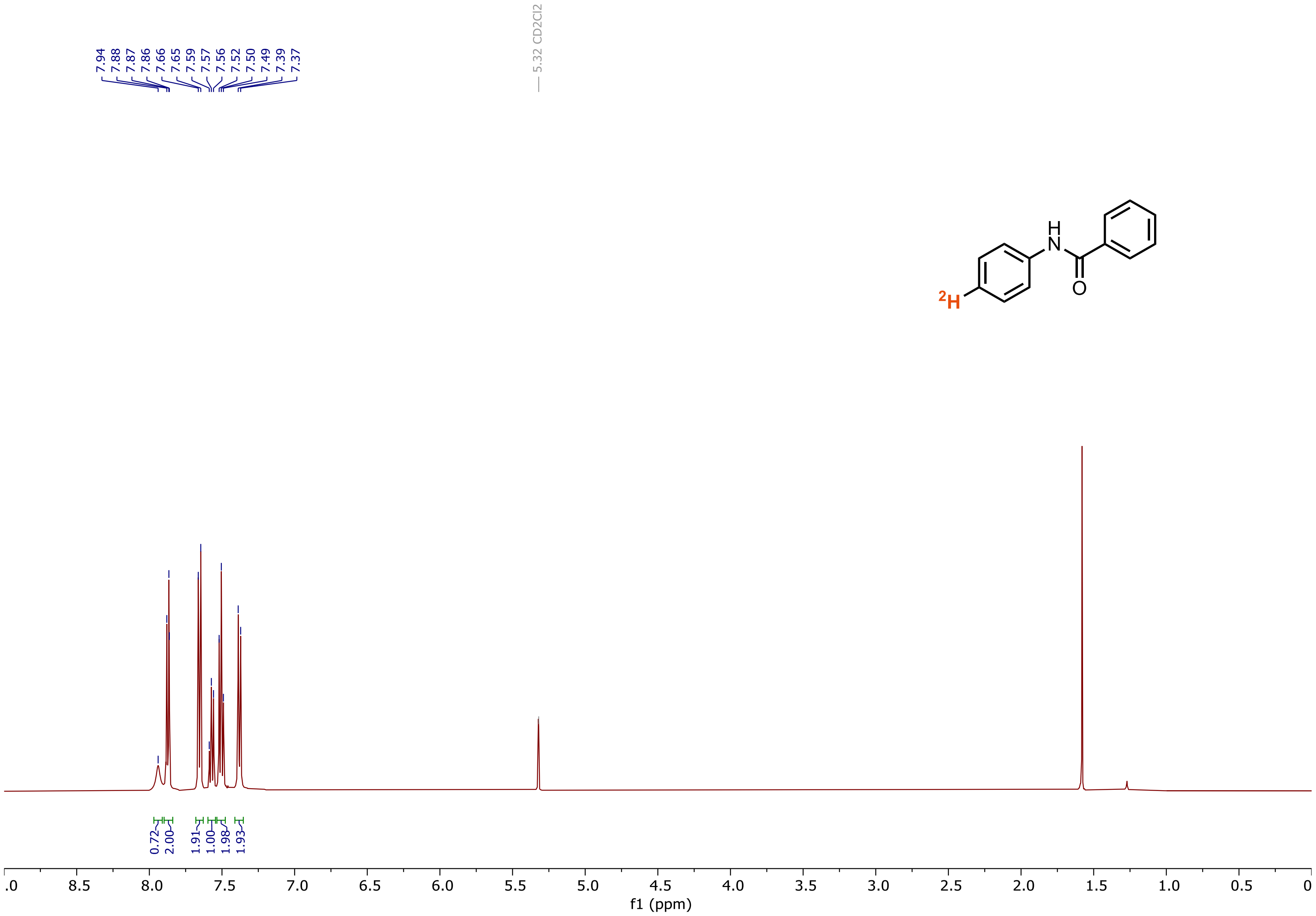

^{2}H NMR of *N*-(4-[^{2}H]-phenyl)benzamide ([^{2}H]9)

CH_2Cl_2, 23 °C

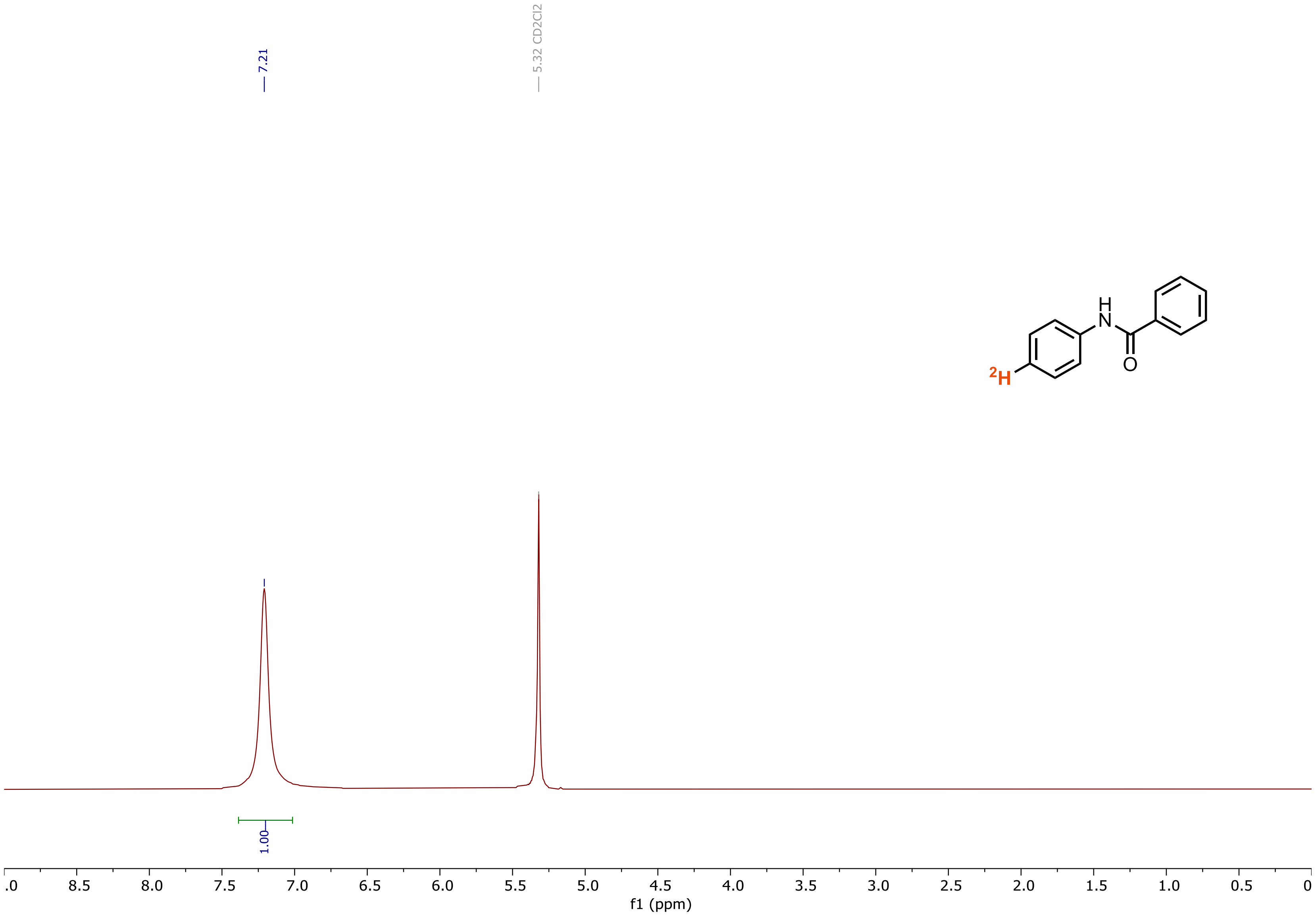

^{13}C NMR of *N*-(4-[^{2}H]-phenyl)benzamide ([^{2}H]9)

CD_2Cl_2, 23 °C

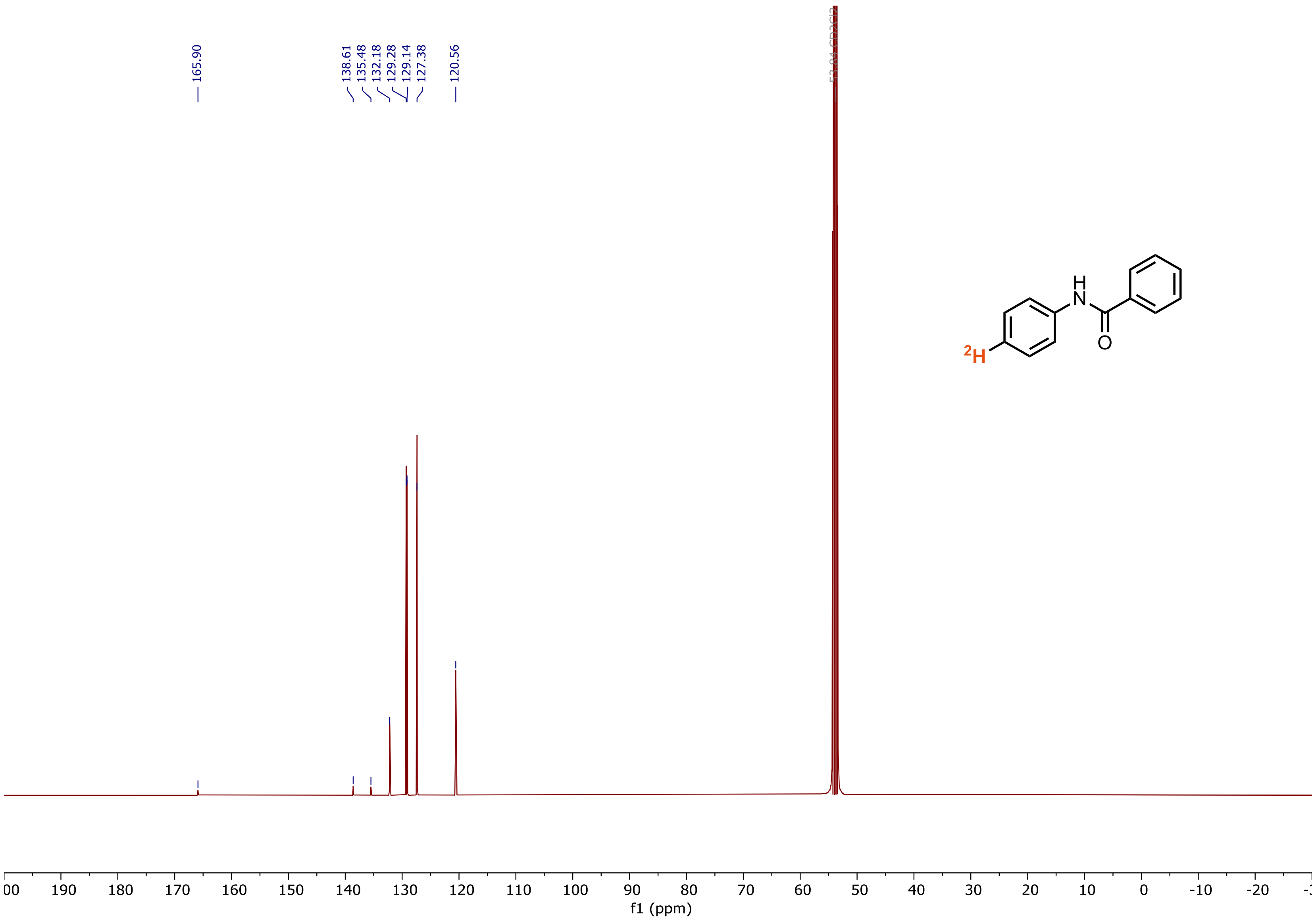

^{1}H NMR of [^{2}H]nefiracetam ([^{2}H]10)

CD_3CN, 23 °C

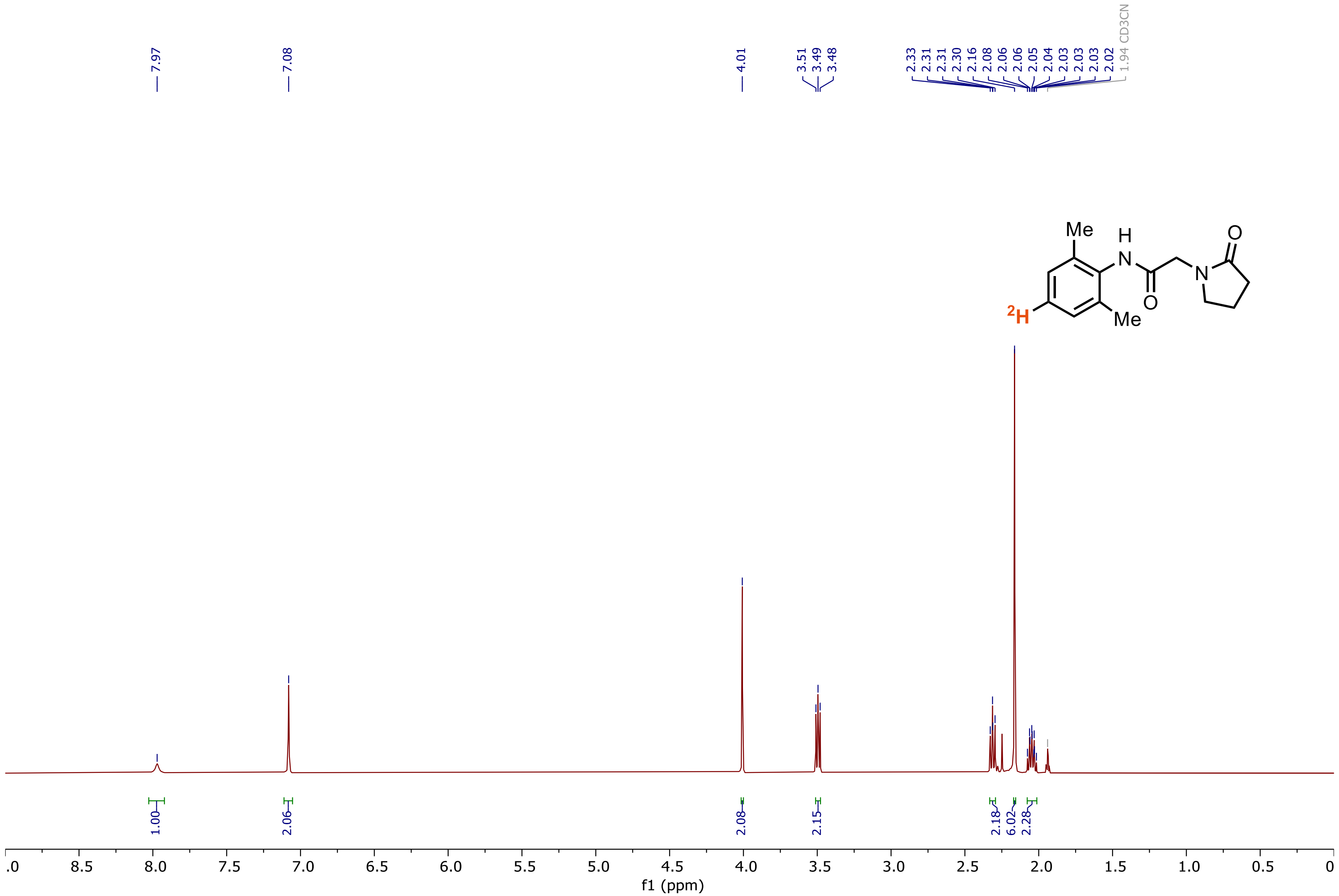

^{2}H NMR of [^{2}H]nefiracetam ([^{2}H]10)

CH_3CN, 23 °C

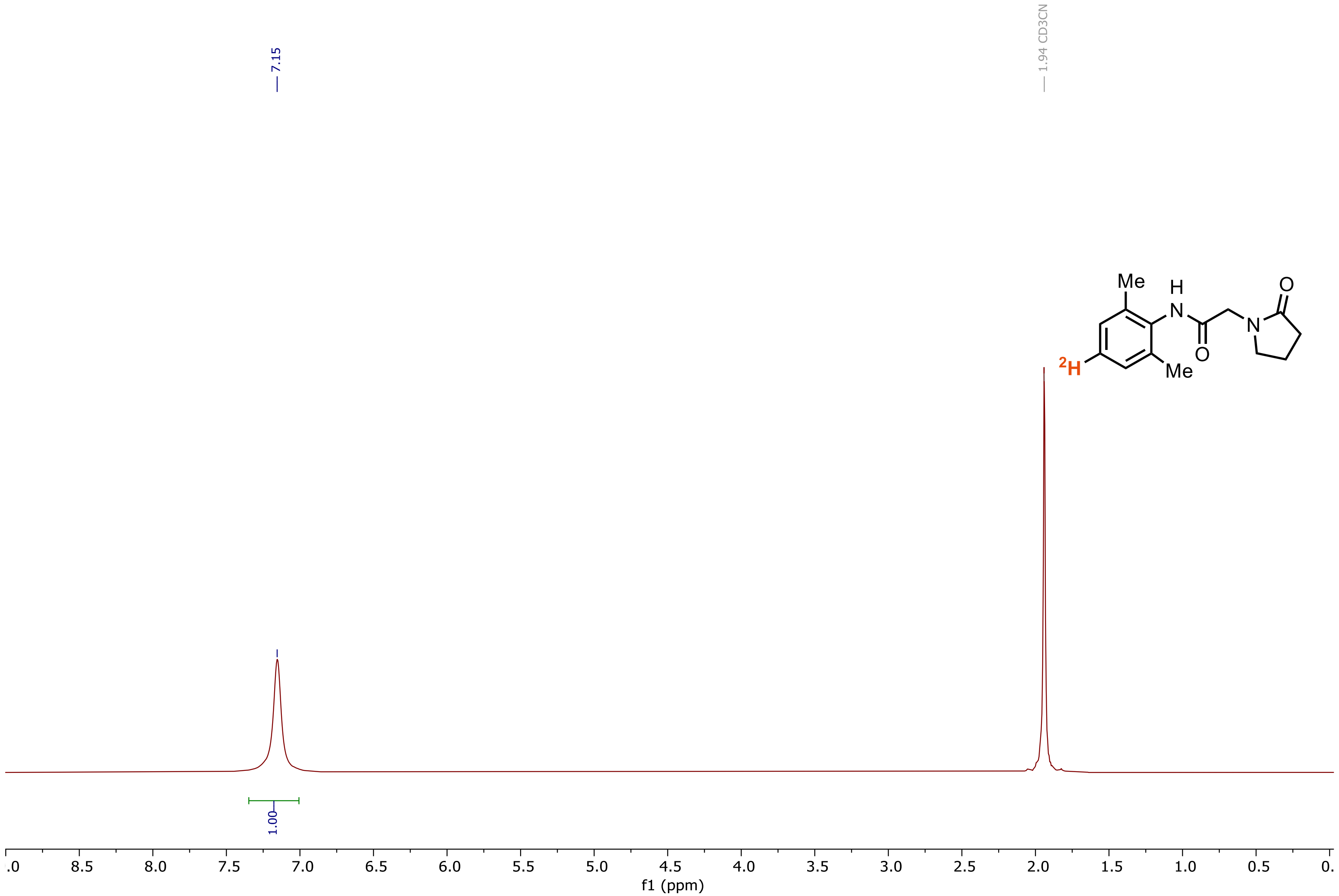

^{13}C NMR of [^{2}H]nefiracetam ([^{2}H]10)

CD_3CN, 23 °C

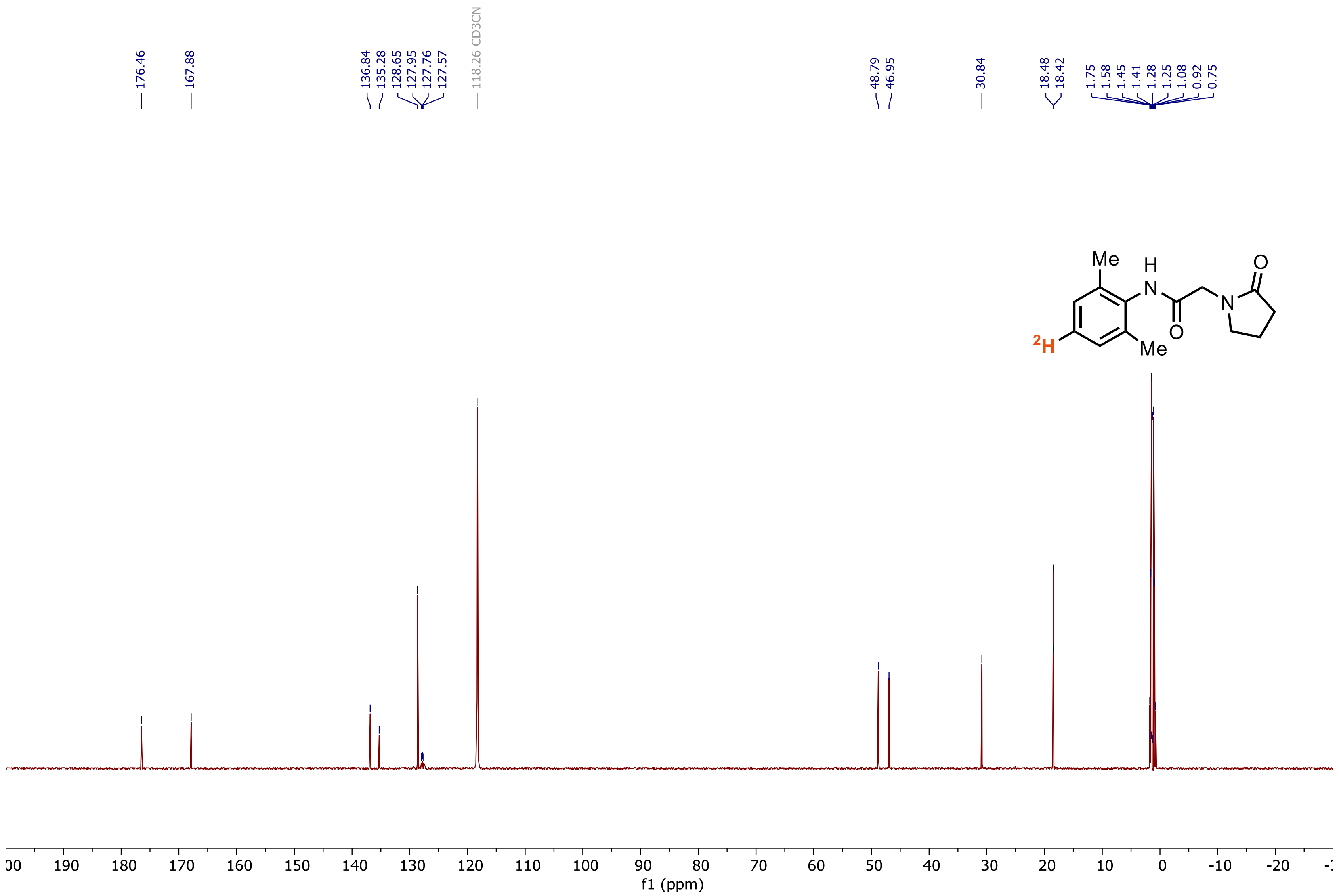

^{1}H NMR of [^{2}H](±)-pyriproxyfen ([^{2}H]11)

CD_3CN, 23 °C

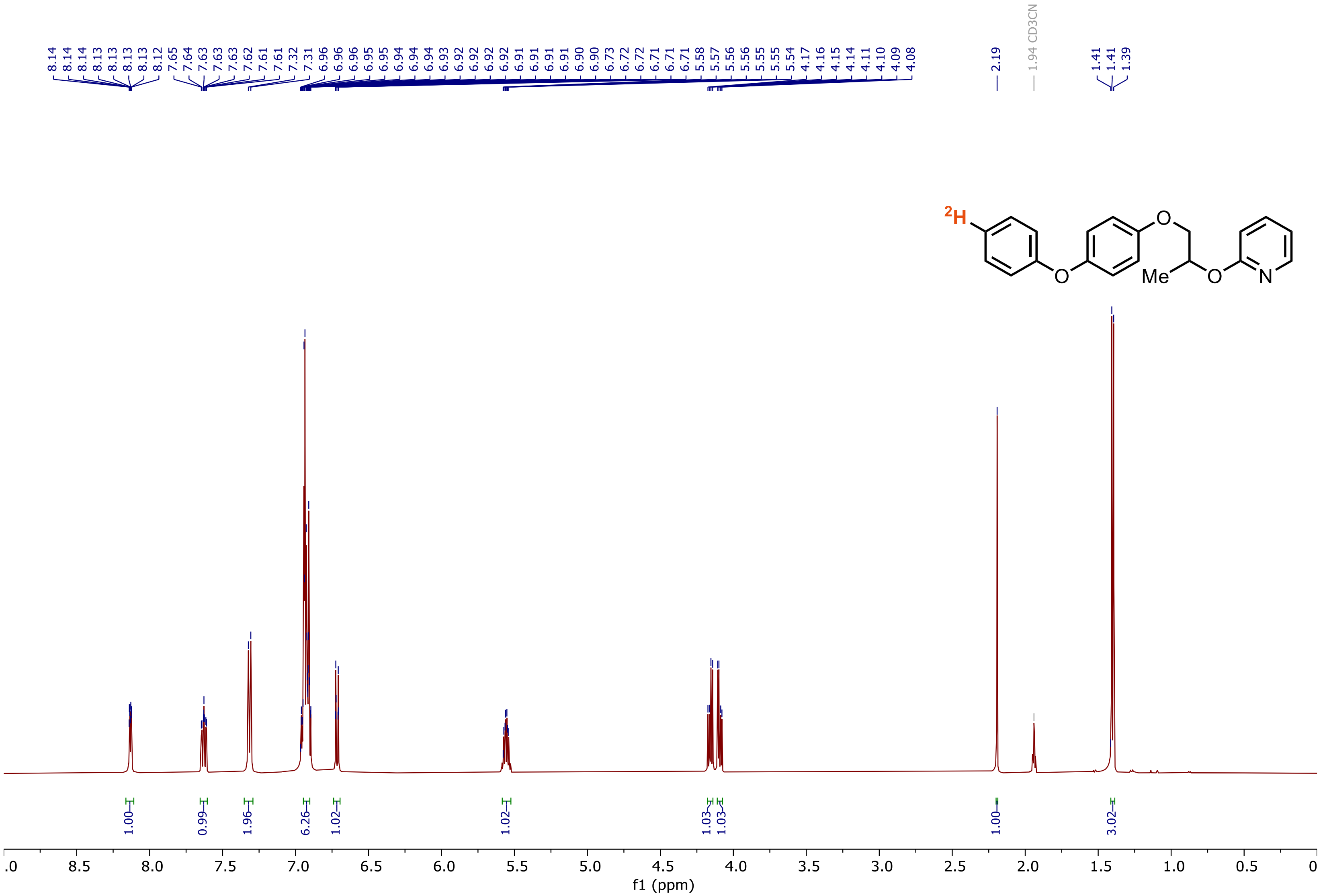

^{2}H NMR of [^{2}H](±)-pyriproxyfen ([^{2}H]11)

CH_3CN, 23 °C

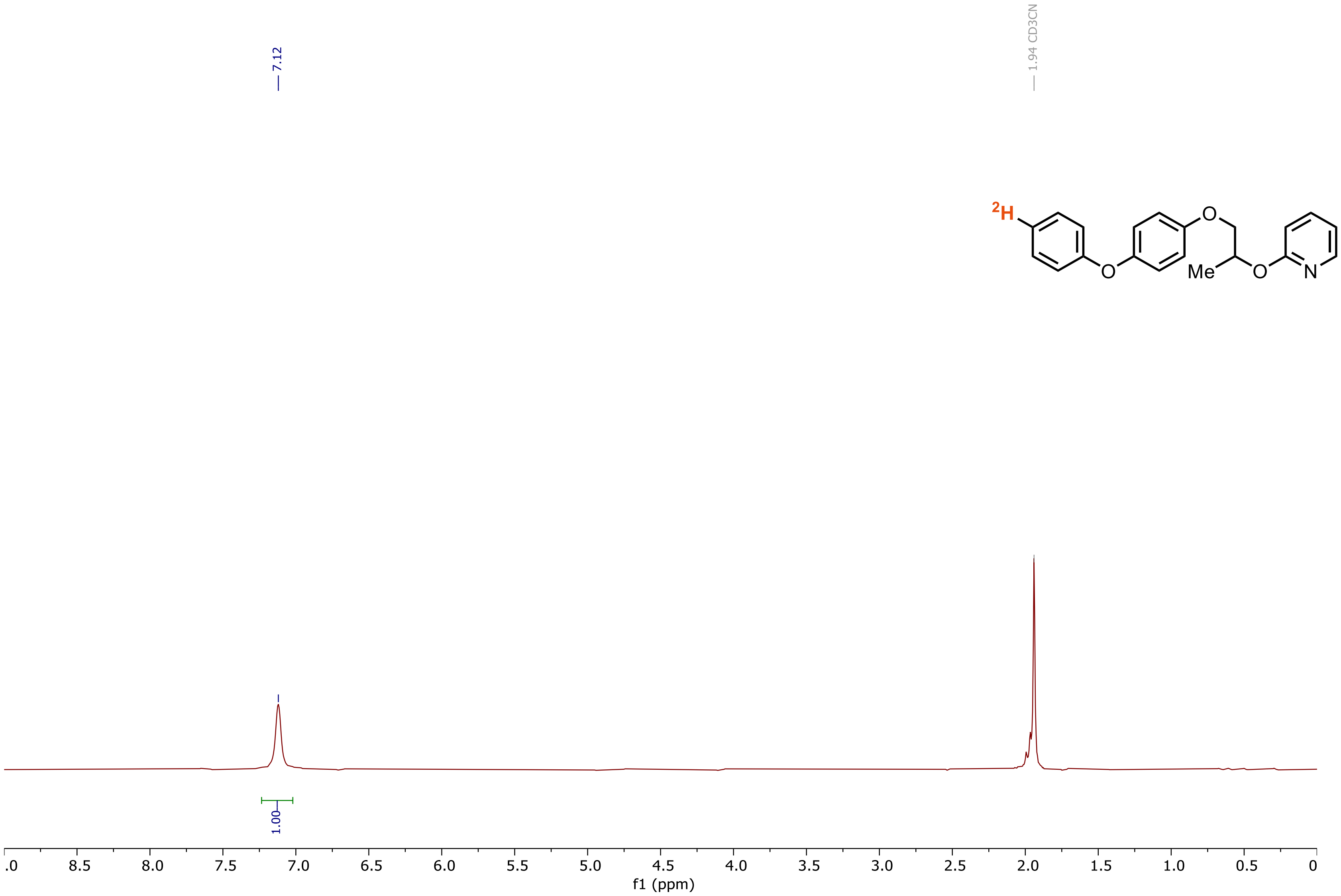

^{13}C NMR of [^2H](±)-pyriproxyfen ([^2H]11)

CD_3CN, 23 °C

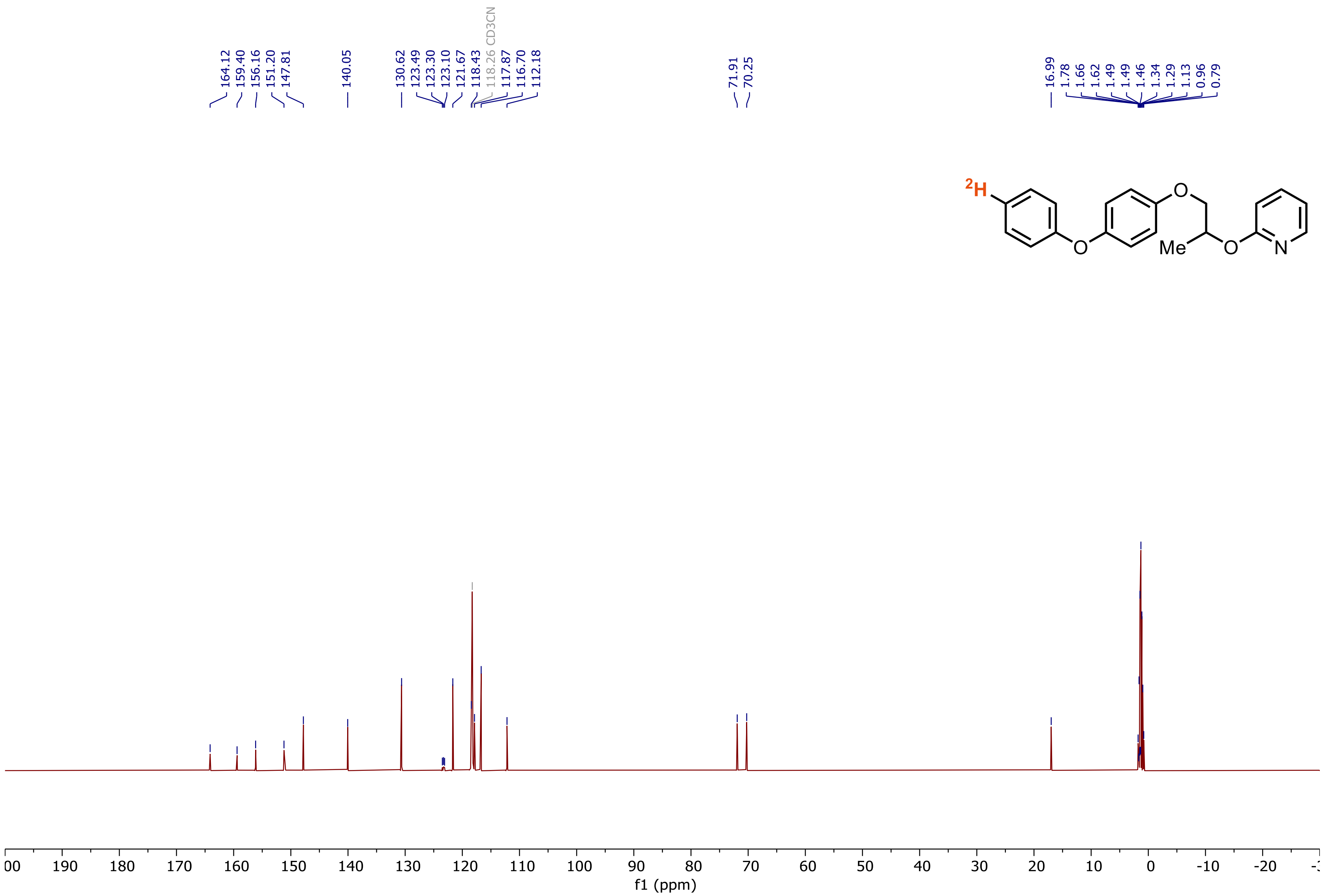

^{1}H NMR of 4'-[^{2}H]-[1,1'-biphenyl]-4-yl trifluoromethanesulfonate ([^{2}H]12)

CD_2Cl_2, 23 °C

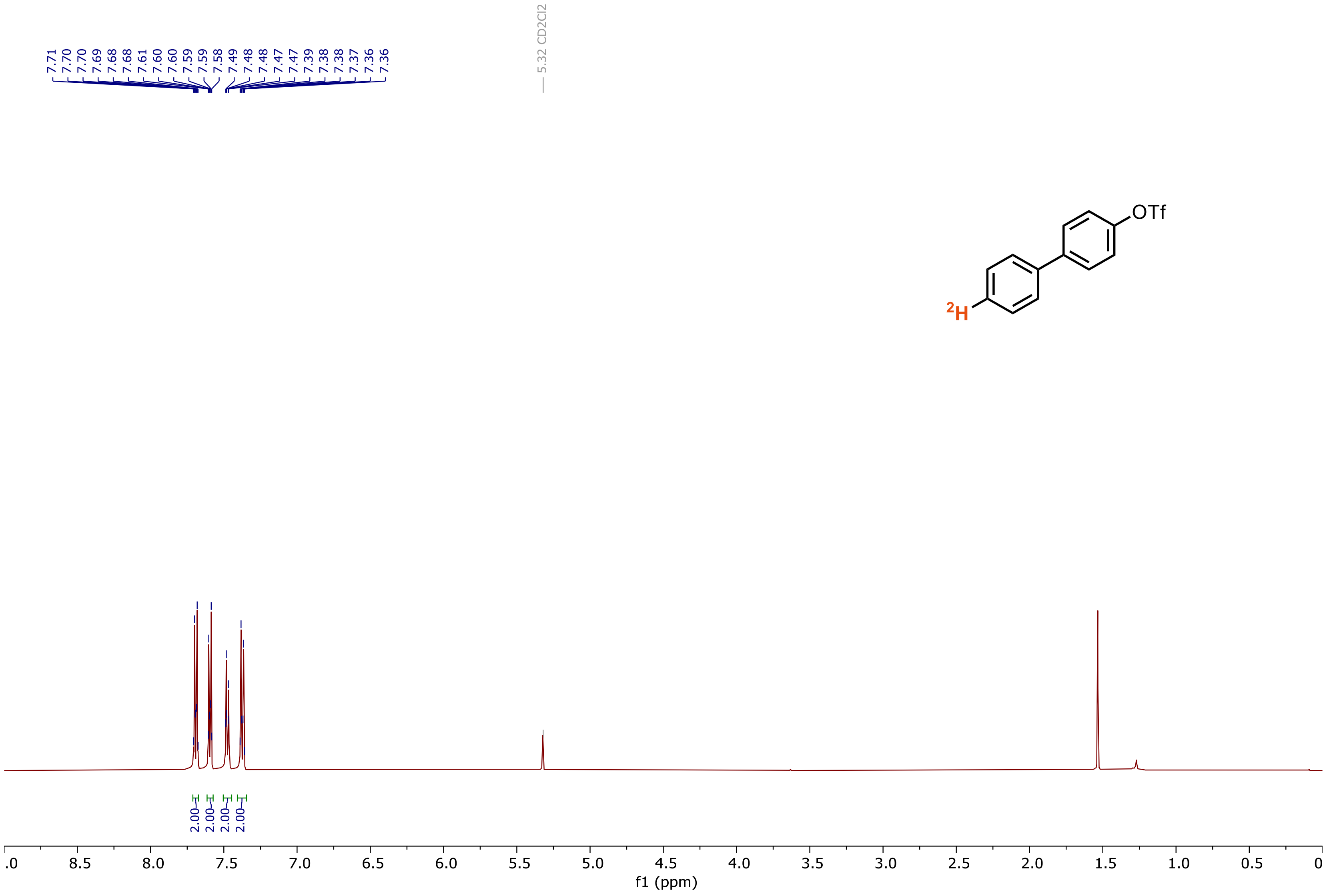

^2H NMR of 4'-[^2H]-[1,1'-biphenyl]-4-yl trifluoromethanesulfonate ([^2H]12)

CH_2Cl_2, 23 °C

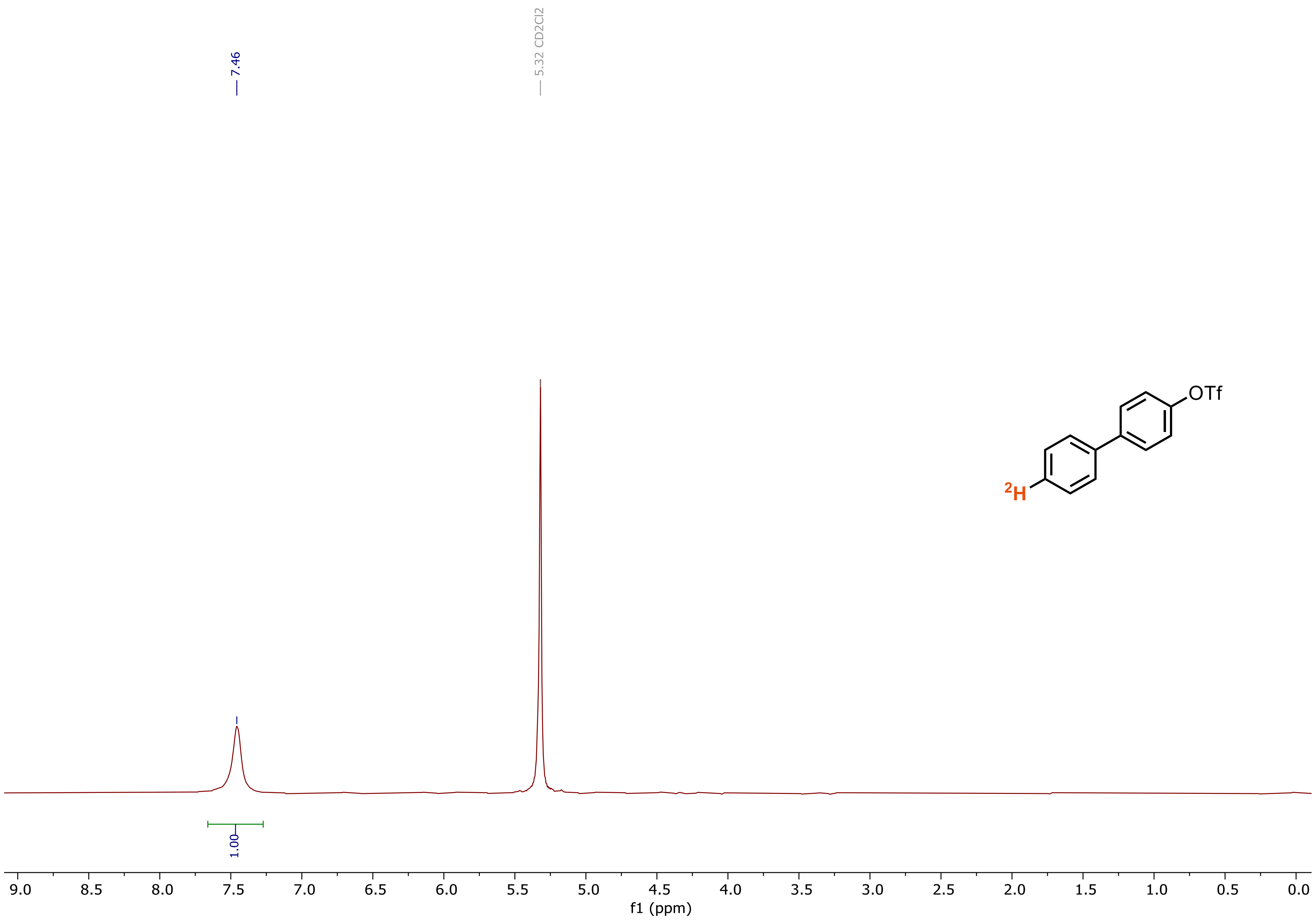

^{13}C NMR of 4'-[^{2}H]-[1,1'-biphenyl]-4-yl trifluoromethanesulfonate ([^{2}H]12)

CD_2Cl_2, 23 °C

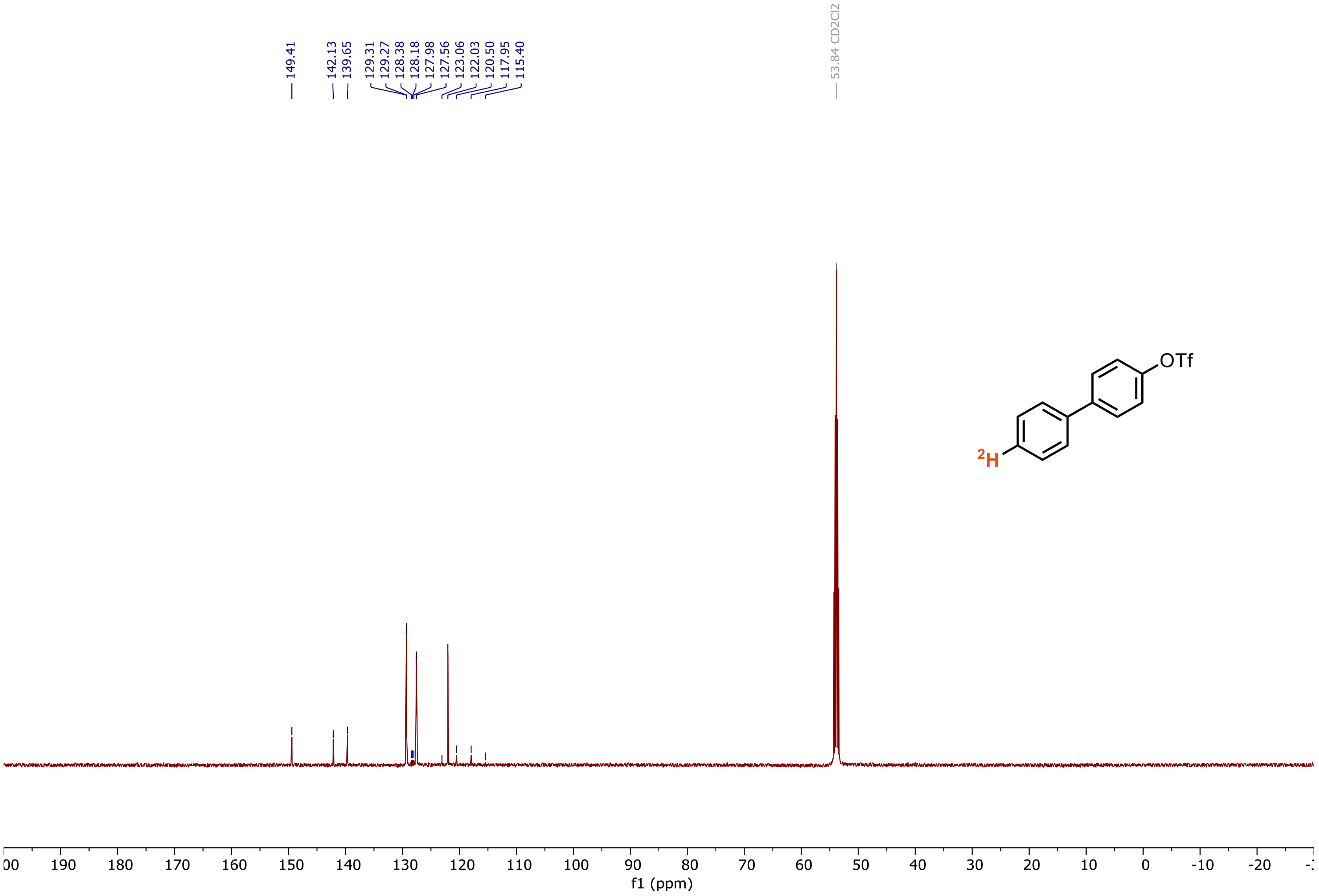

^{19}F NMR of 4'-[^2H]-[1,1'-biphenyl]-4-yl trifluoromethanesulfonate ([^2H]12)

CD_2Cl_2, 23 °C

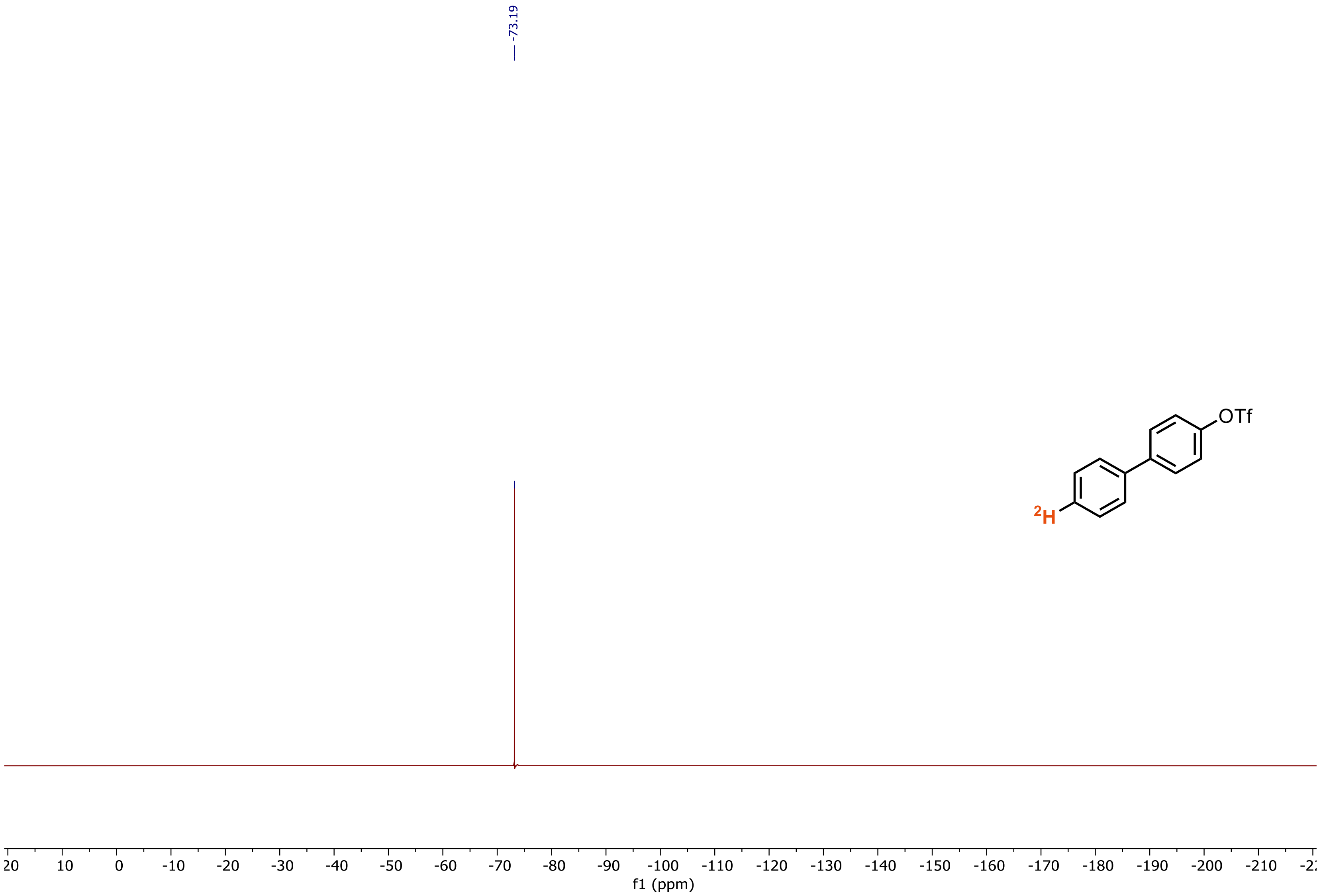

^{1}H NMR of [^{2}H]-tetrahydrobenzofuranone ([^{2}H]13)

CD_3CN, 23 °C

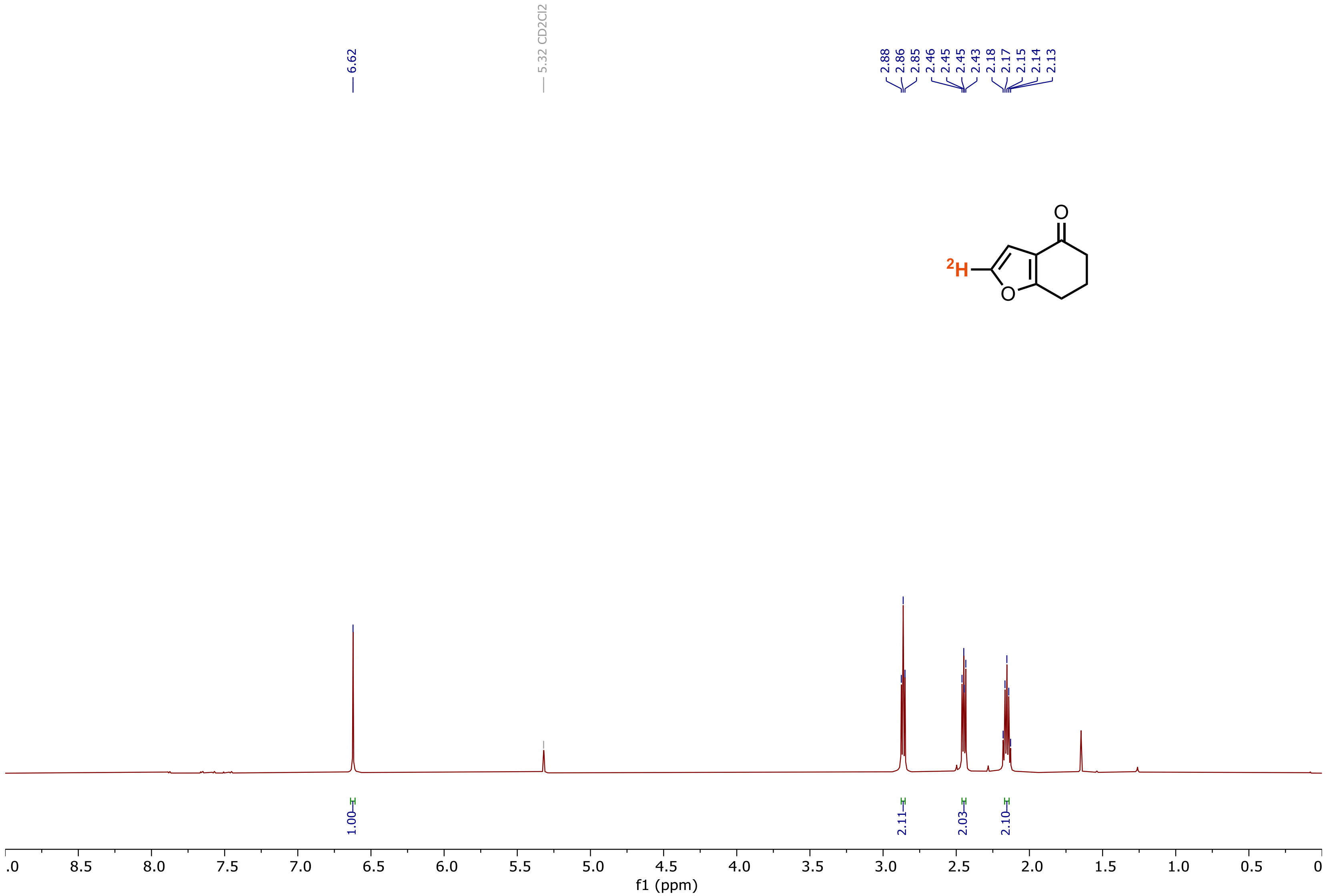

^{2}H NMR of [^{2}H]-tetrahydrobenzofuranone ([^{2}H]13)

CH_3CN, 23 °C

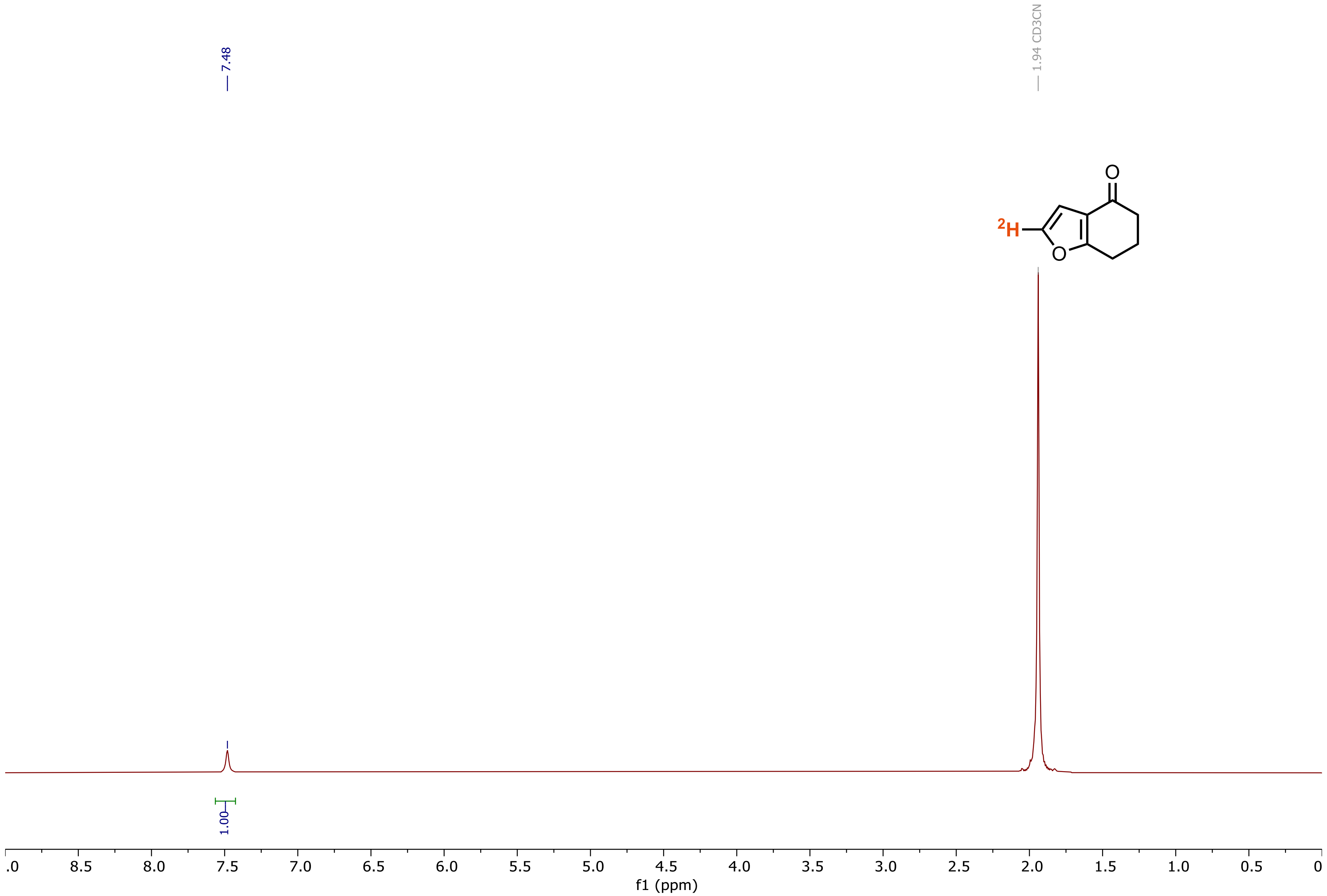

^{13}C NMR of [^{2}H]-tetrahydrobenzofuranone ([^{2}H]13)

CD_3CN, 23 °C

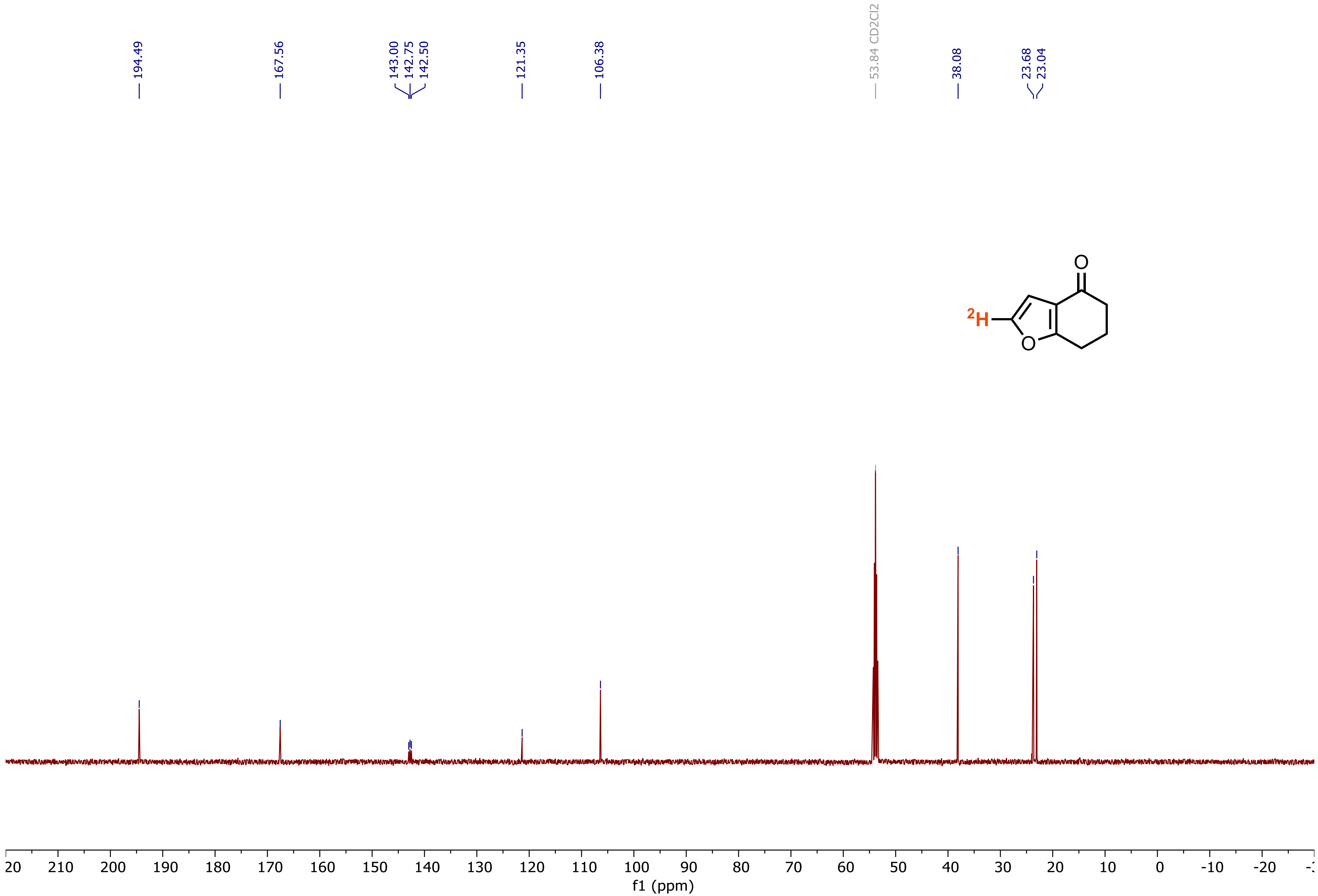

^{1}H NMR of 4-[^{2}H]-acetylmethylalanate ([^{2}H]14)

CD_2Cl_2, 23 °C

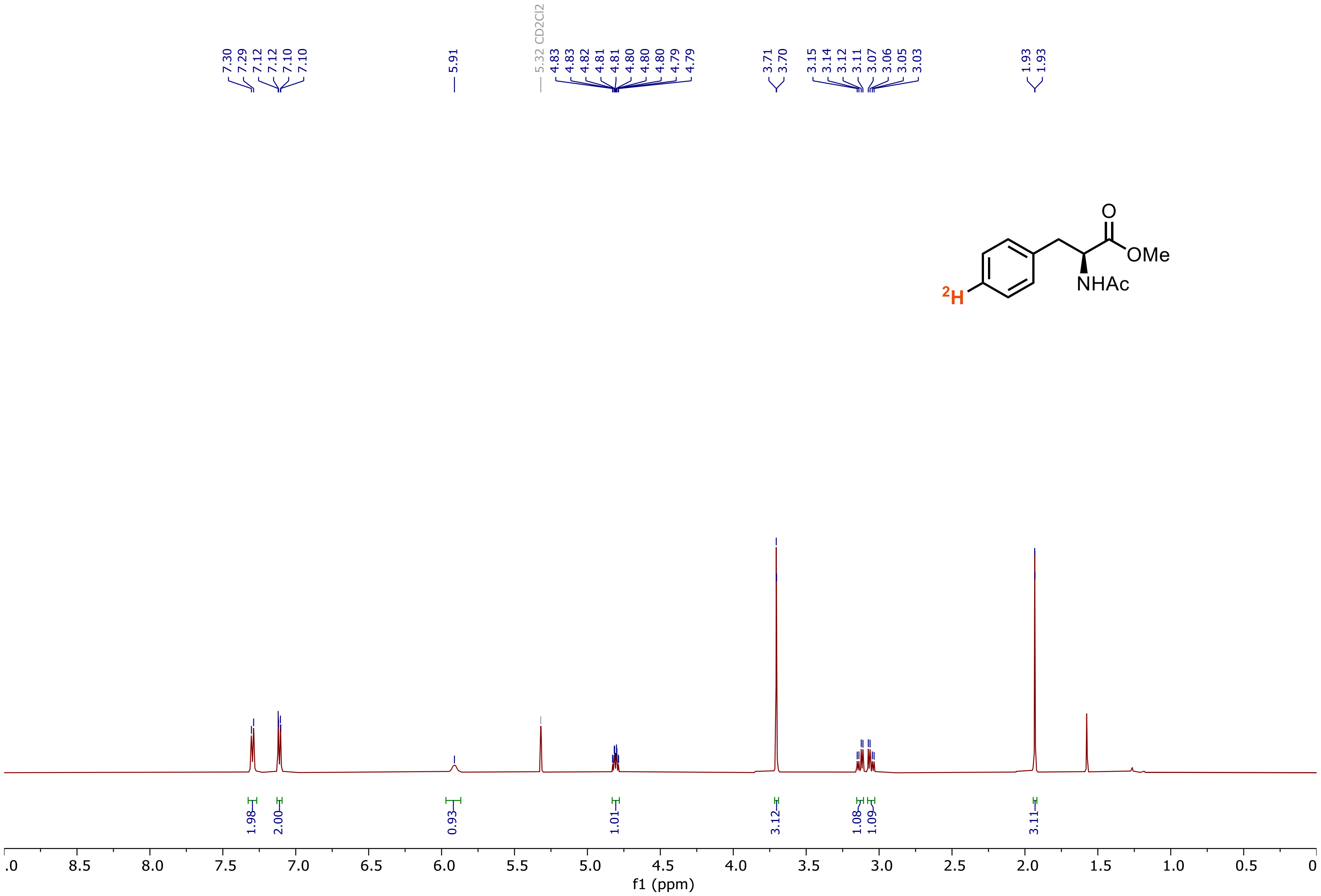

^{2}H NMR of 4-[^{2}H]-acetylmethylalanate ([^{2}H]14)

CH_2Cl_2, 23 °C

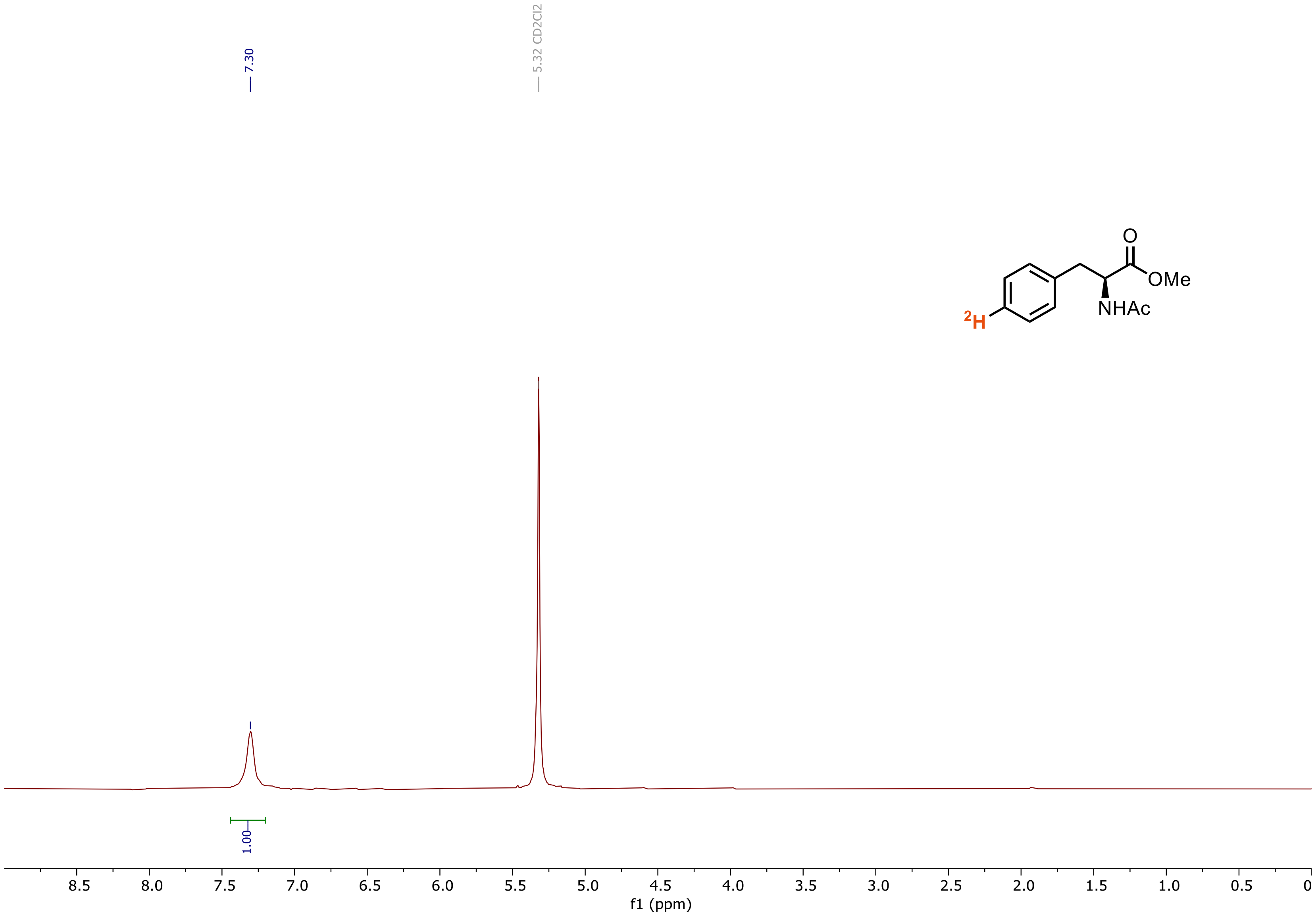

^{13}C NMR of 4-[^{2}H]-acetylmethylalanate ([^{2}H]14)

CD_2Cl_2, 23 °C

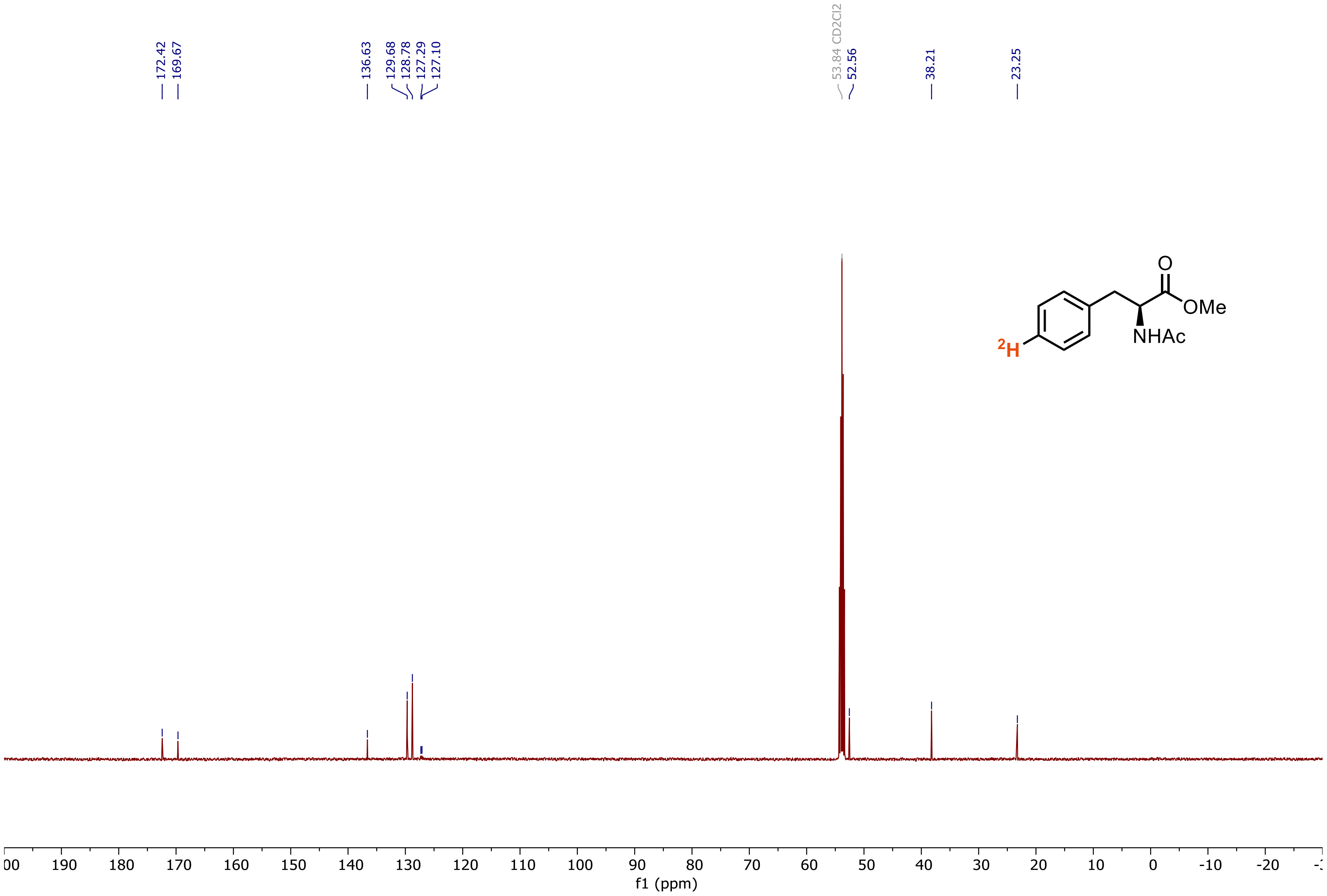

^{1}H NMR of [^{2}H]indomethacin methylester ([^{2}H]15)

CD_3CN, 23 °C

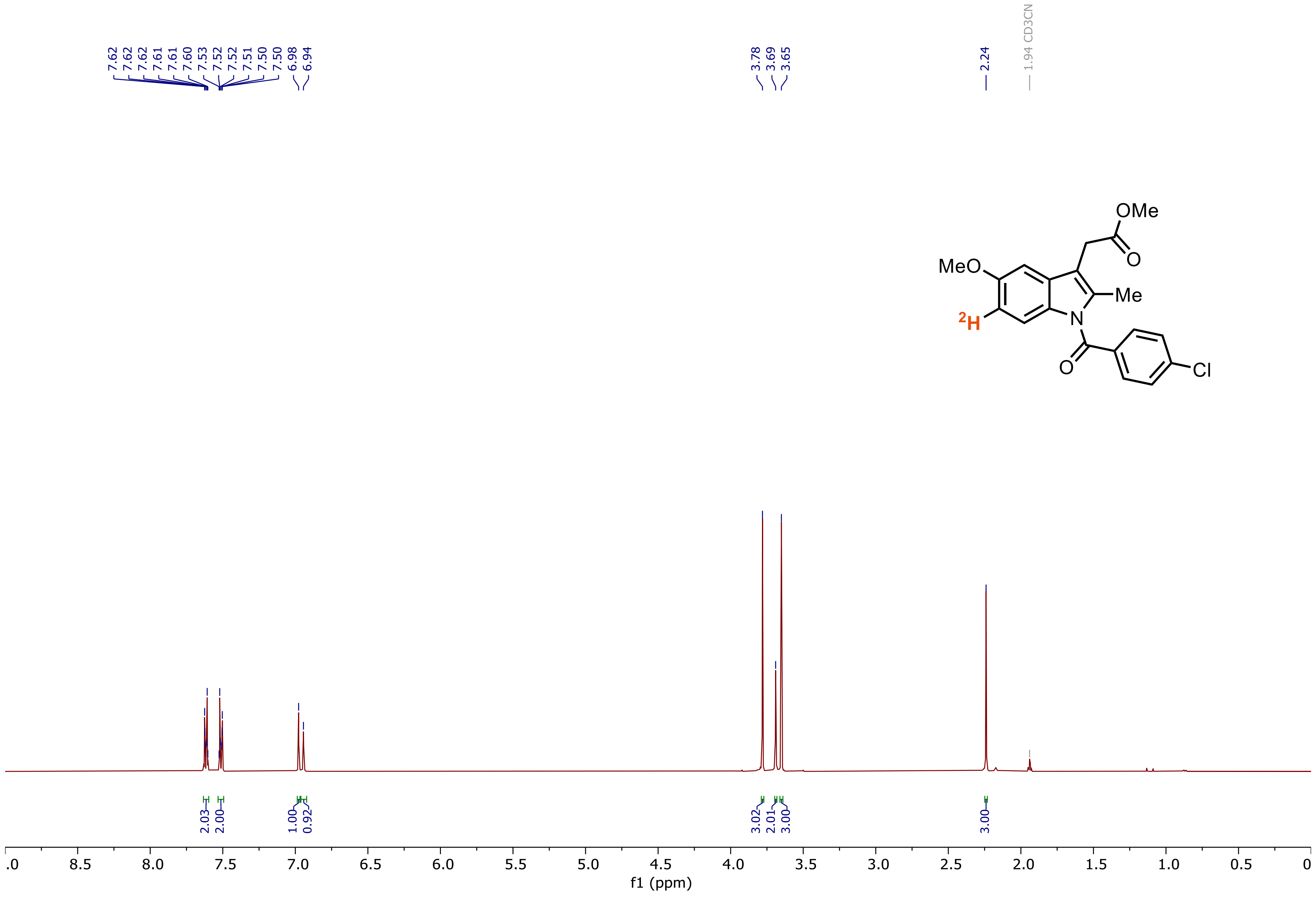

^{2}H NMR of [^{2}H]indomethacin methylester ([^{2}H]15)

CH_3CN, 23 °C

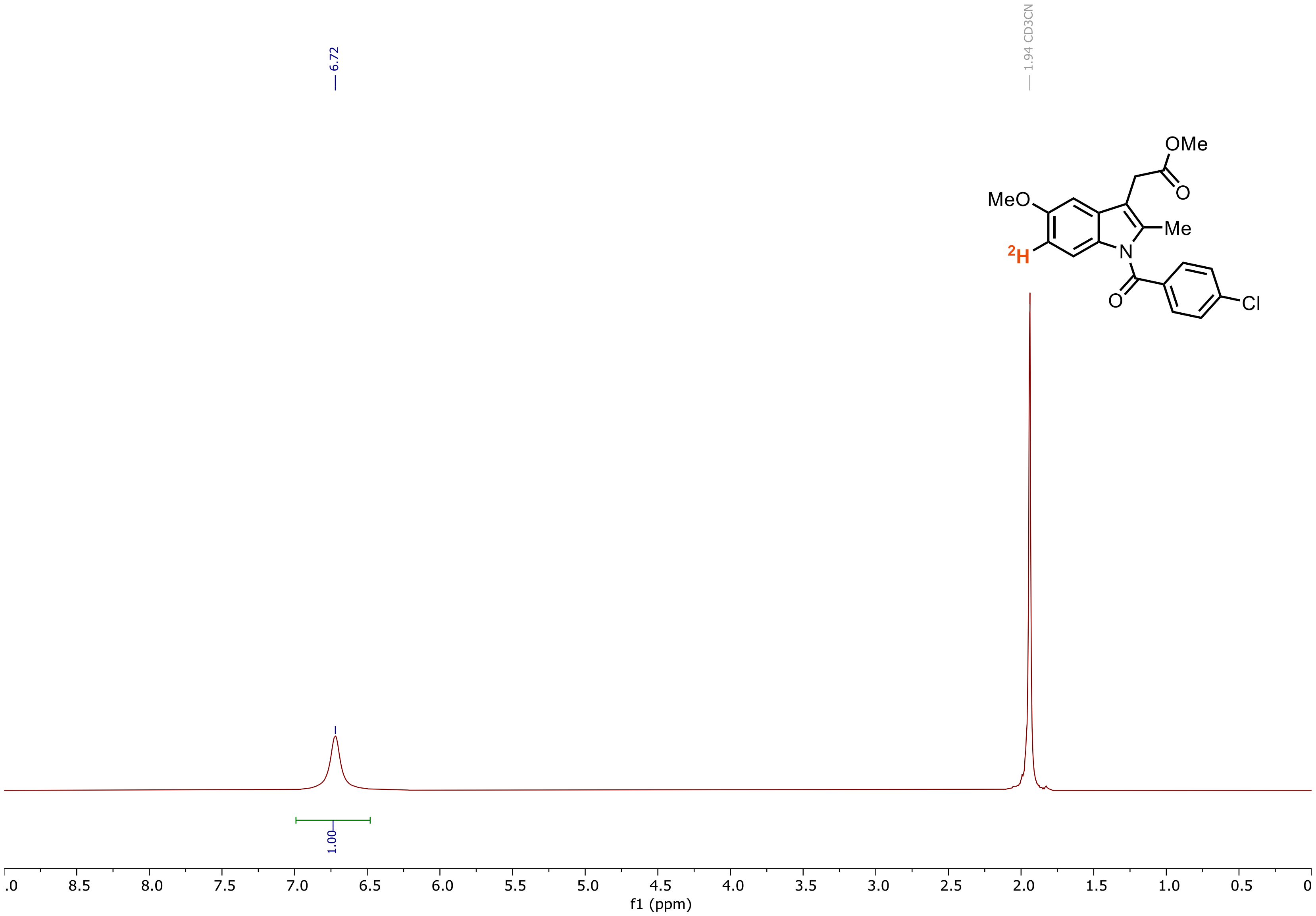

^{13}C NMR of [^{2}H]indomethacin methylester ([^{2}H]15)

CD_3CN, 23 °C

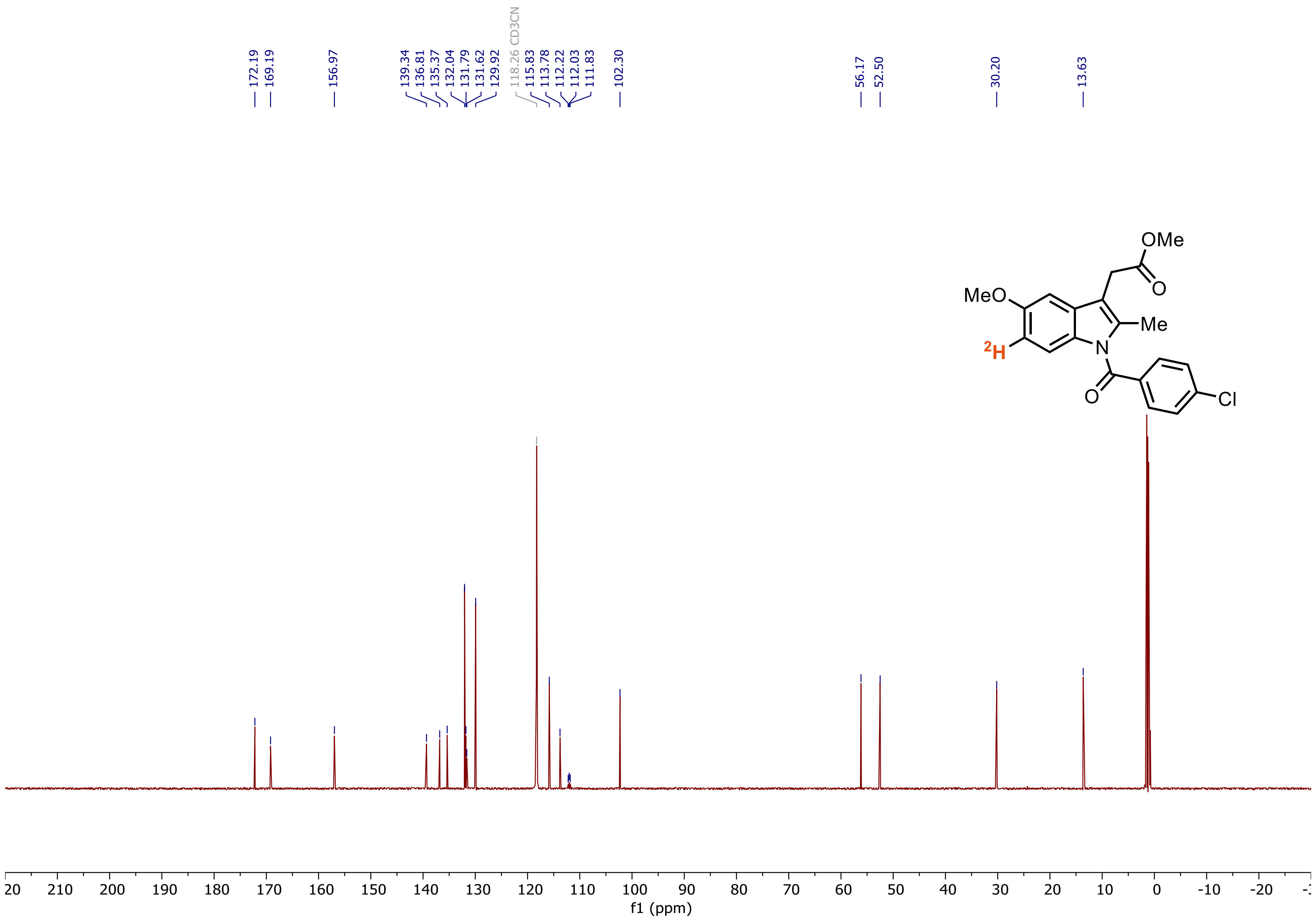

^{1}H NMR of [^{2}H](±)-famoxadone ([^{2}H]16)

CD$_3$CN, 23 °C

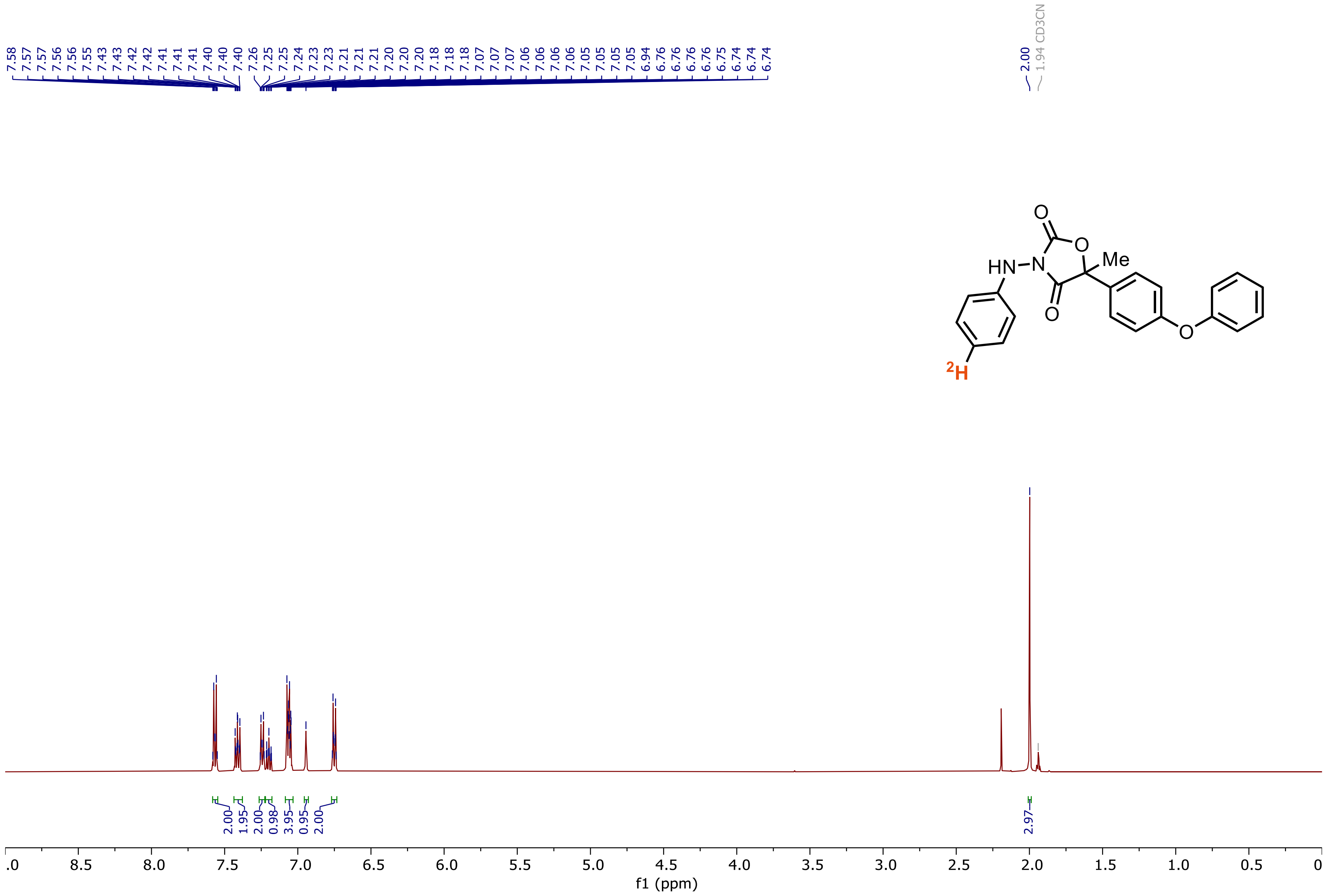

^{2}H NMR of [^{2}H](±)-famoxadone ([^{2}H]16)

CH_3CN, 23 °C

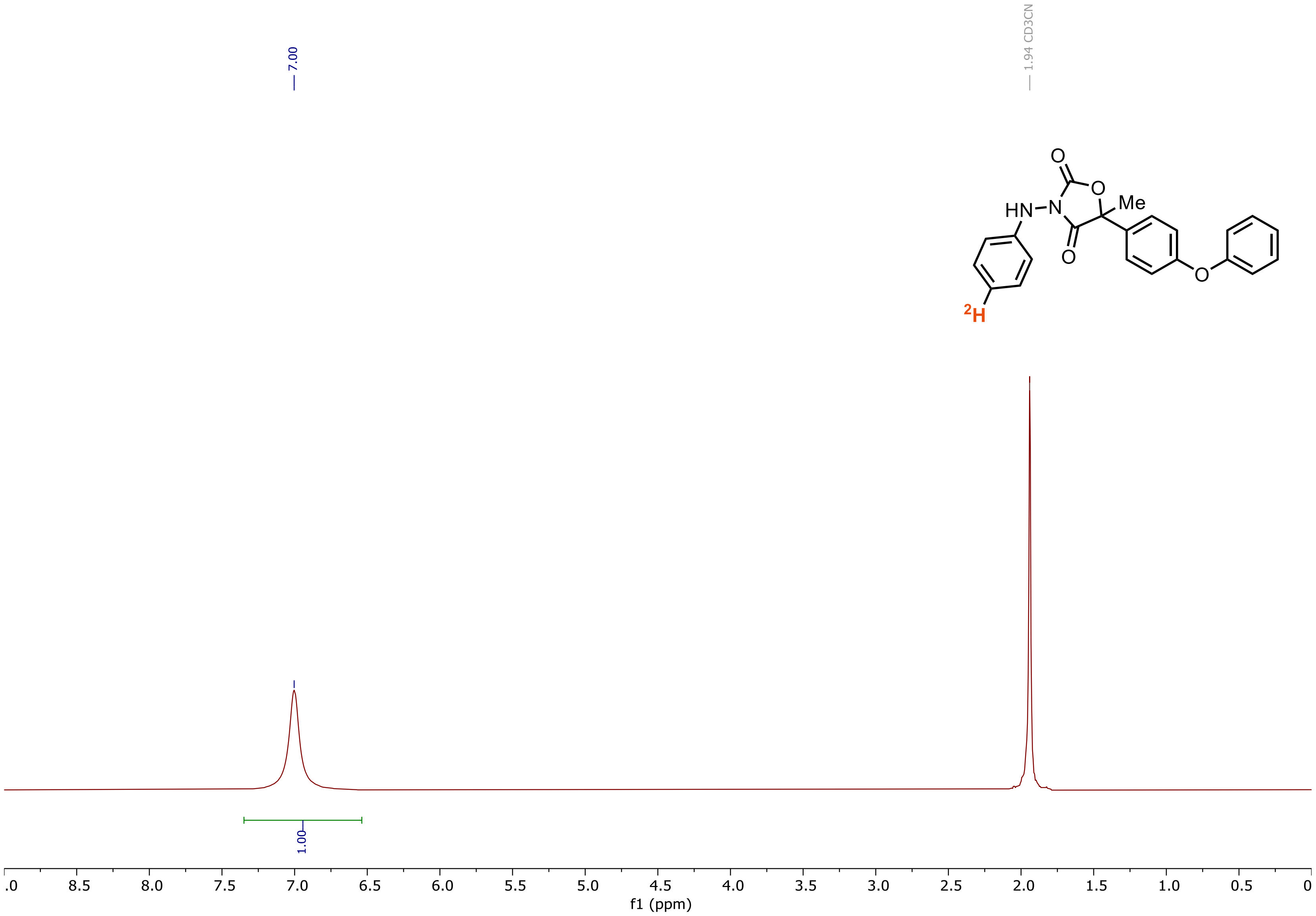

^{13}C NMR of [^{2}H](±)-famoxadone ([^{2}H]16)

CD_3CN, 23 °C

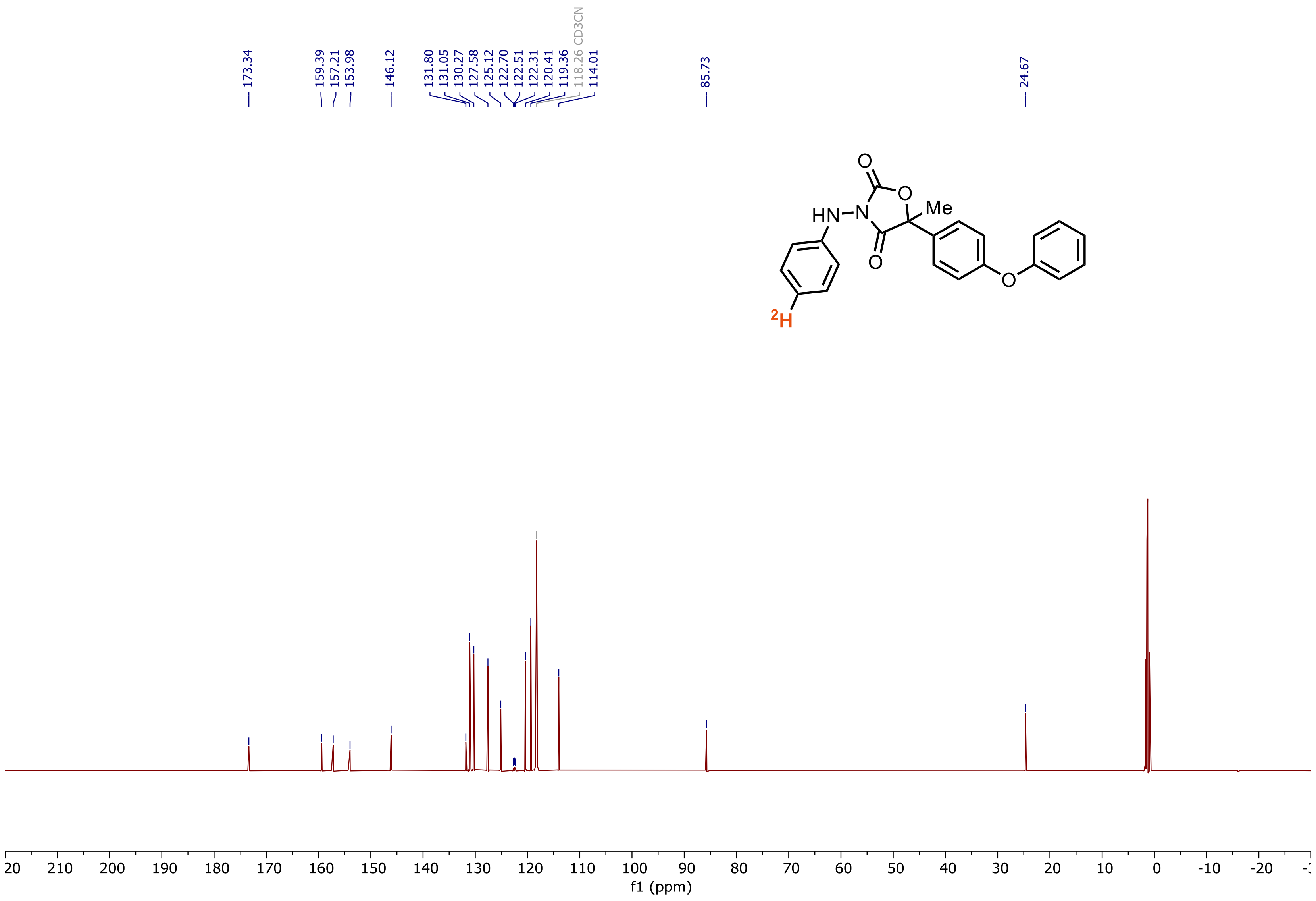

^{1}H NMR of 2-[^{2}H]-3-methyl-5-acetyl thiophene ([^{2}H]17)

CD_2Cl_2, 23 °C

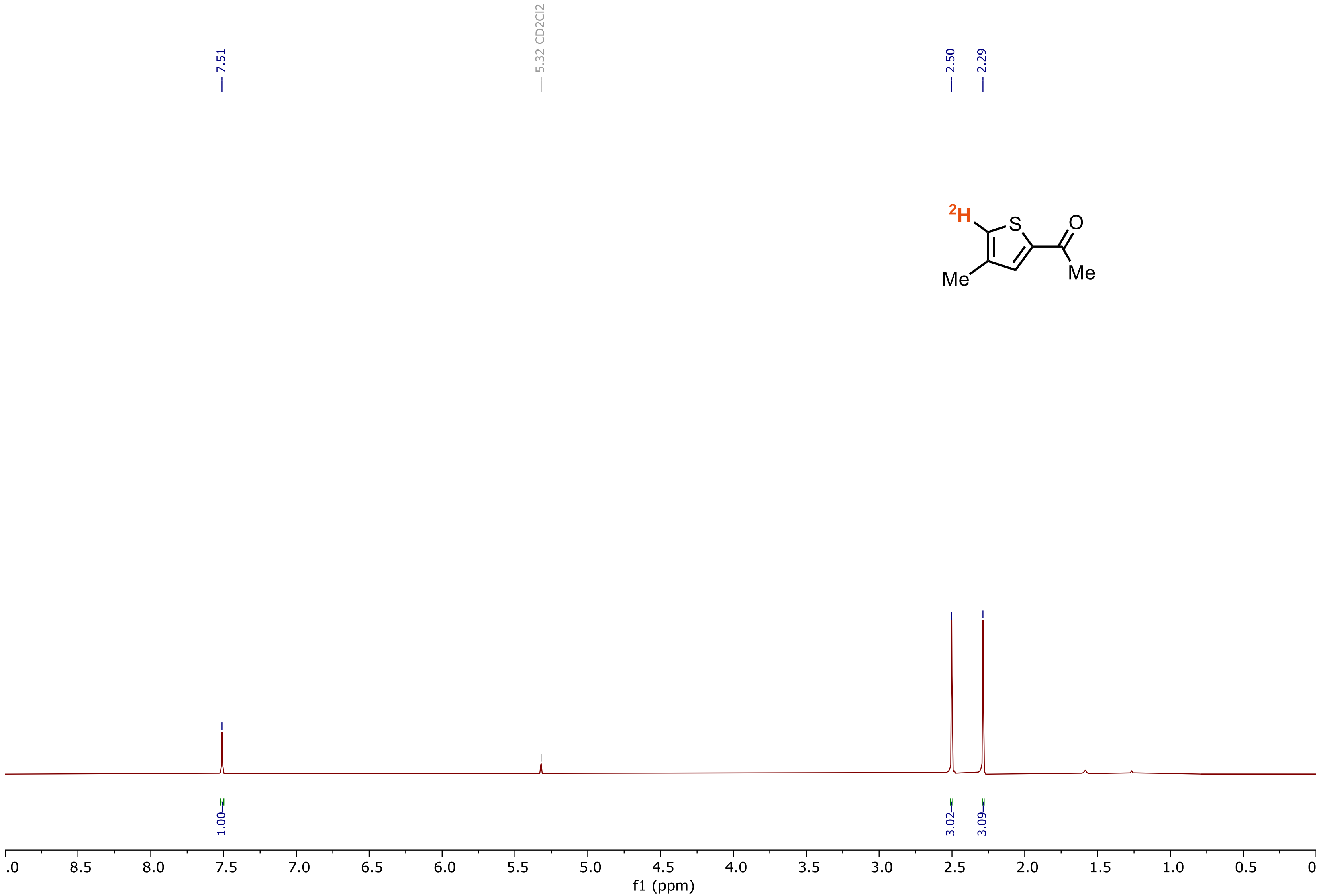

^{2}H NMR of 2-[^{2}H]-3-methyl-5-acetyl thiophene ([^{2}H]17)

CH_2Cl_2, 23 °C

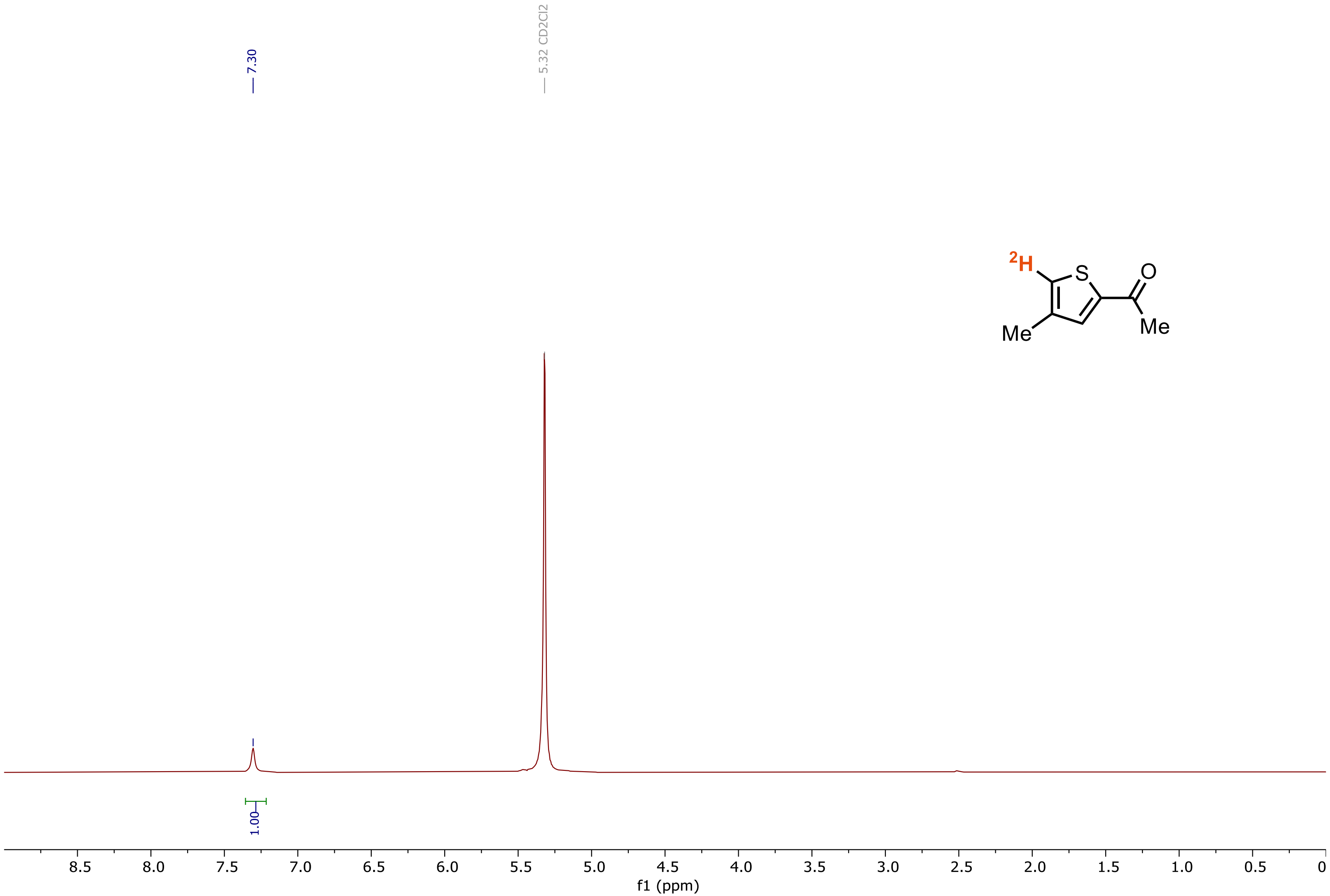

^{13}C NMR of 2-[^{2}H]-3-methyl-5-acetyl thiophene ([^{2}H]17)

CD_2Cl_2, 23 °C

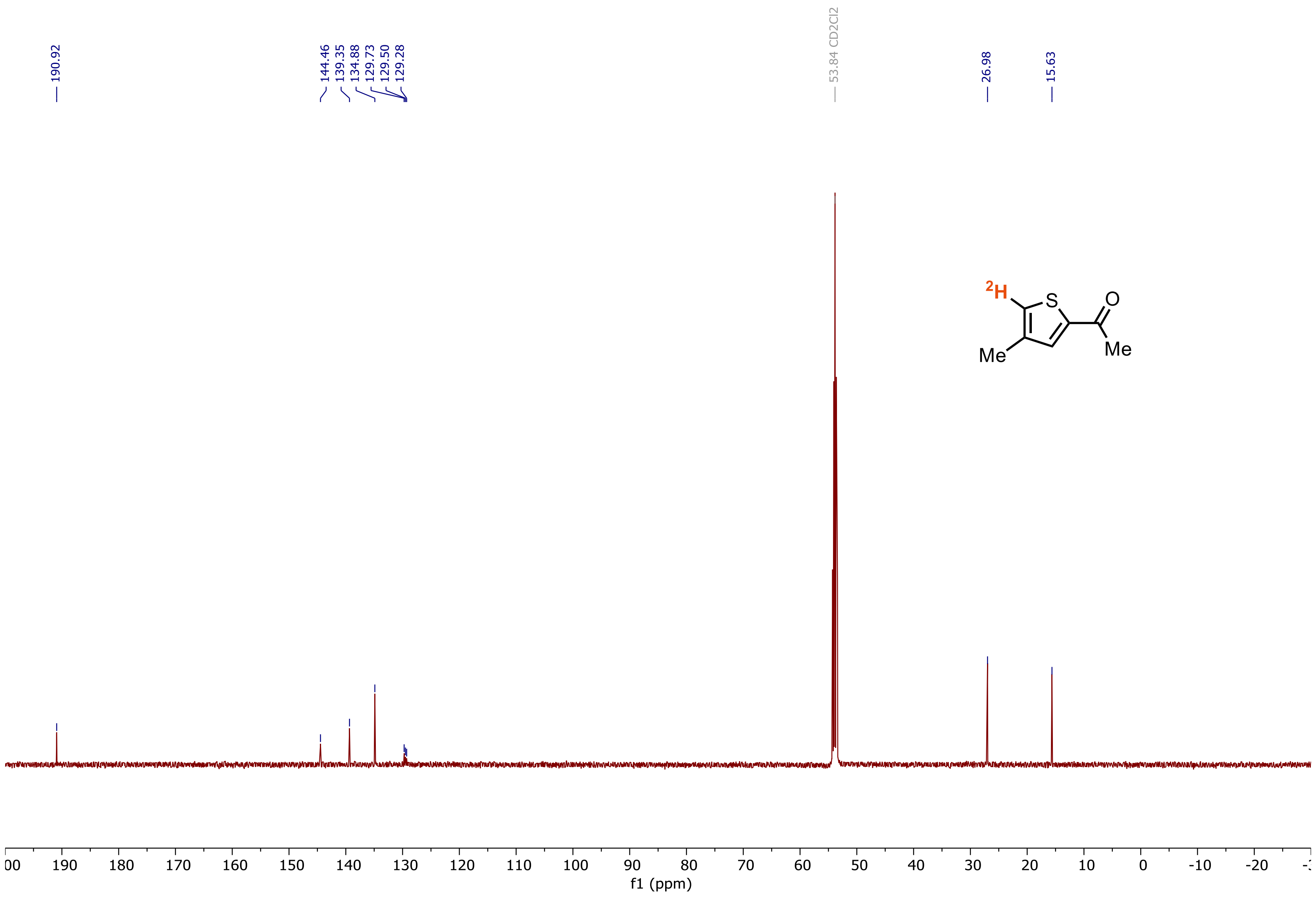

^{1}H NMR of 1-phenyl-4-[^{2}H]-1H-pyrazole ([^{2}H]18)

CD_2Cl_2, 23 °C

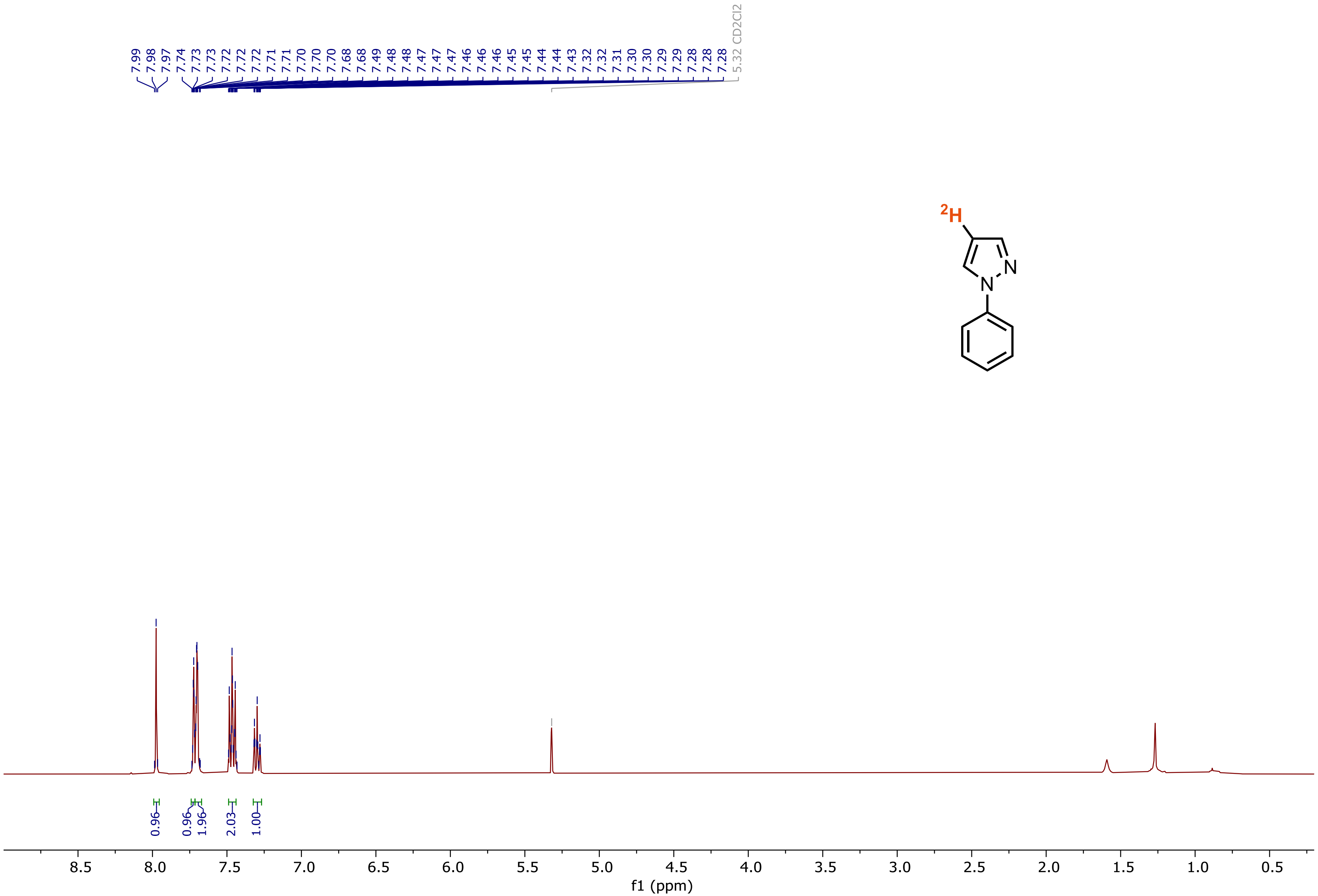

^{2}H NMR of 1-phenyl-4-[^{2}H]-1H-pyrazole ([^{2}H]18)

CH_2Cl_2, 23 °C

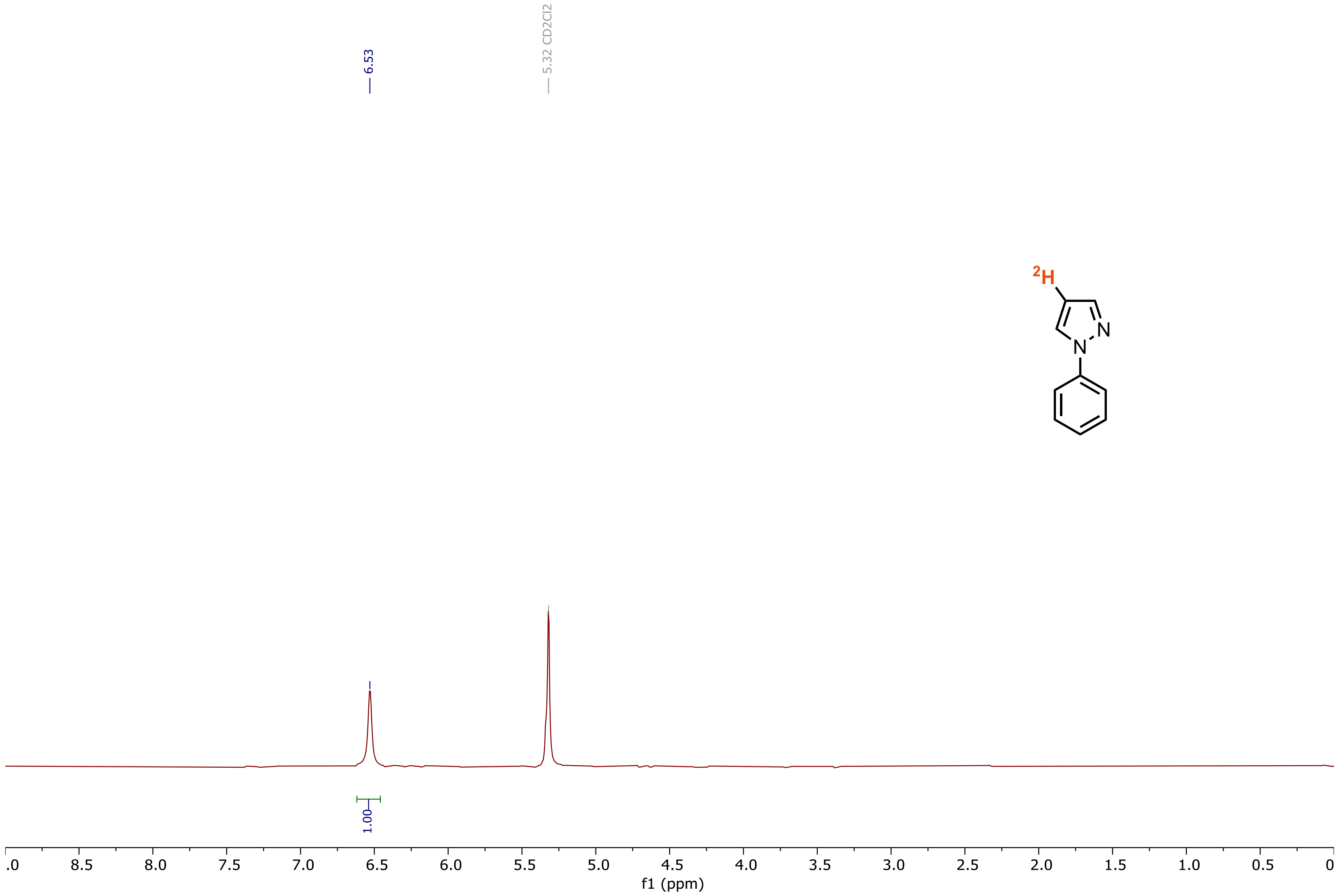

^{13}C NMR of 1-phenyl-4-[^{2}H]-1H-pyrazole ([^{2}H]18)

CD_2Cl_2, 23 °C

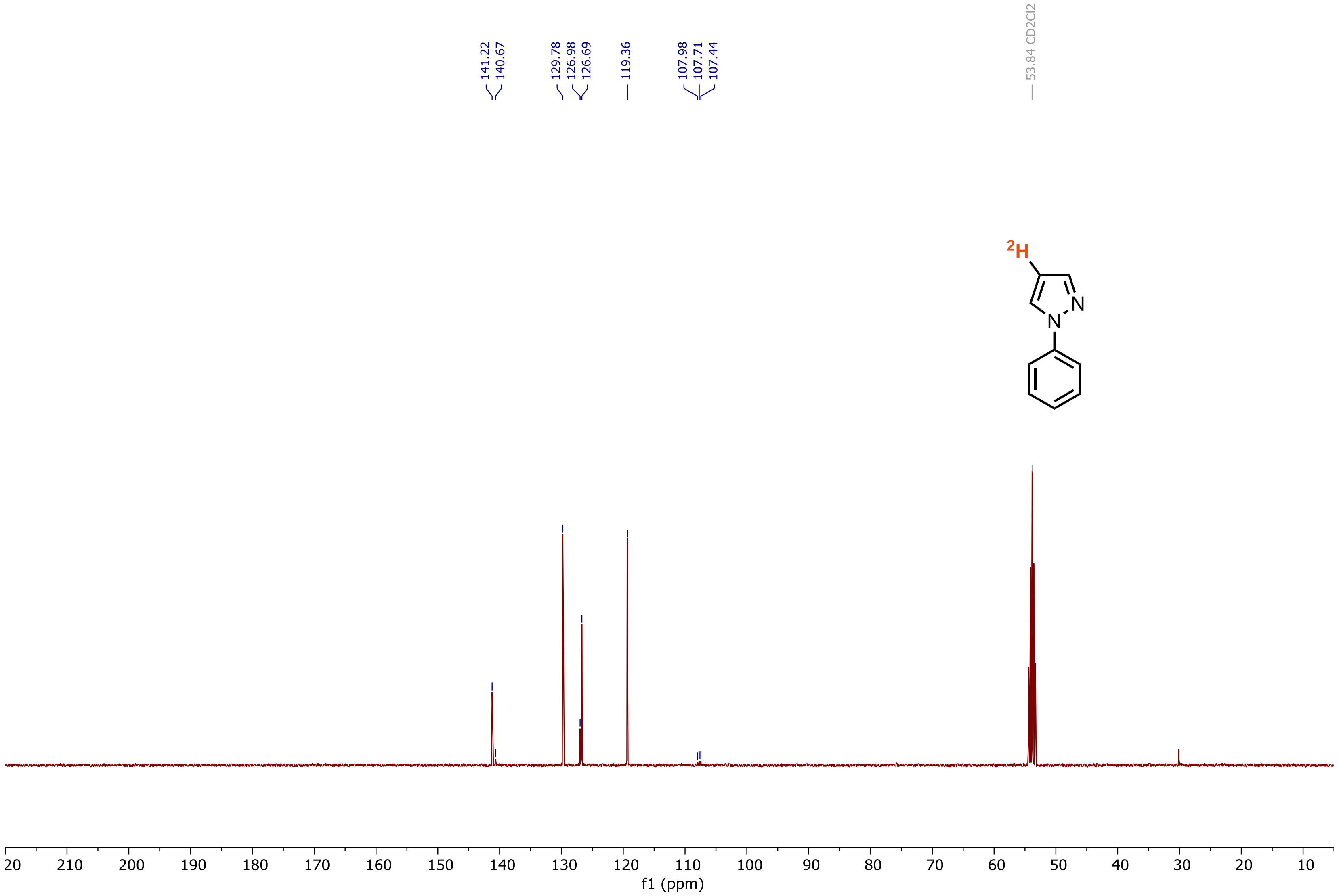

^{1}H NMR of 2-nitro-4'-[^{2}H]-biphenyl ([^{2}H]19)

CD_2Cl_2, 23 °C

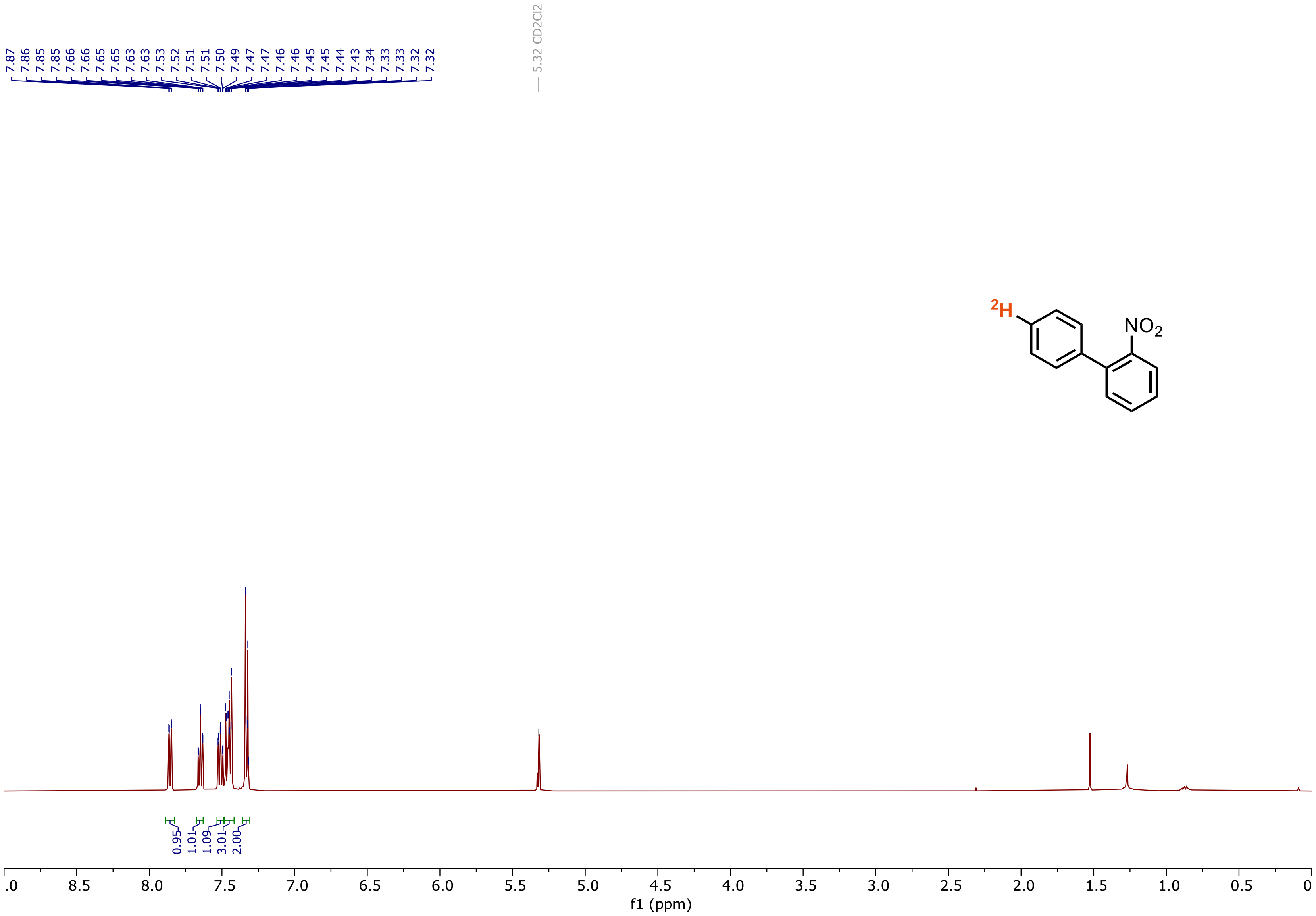

^{2}H NMR of 2-nitro-4'-[^{2}H]-biphenyl ([^{2}H]19)

CH_2Cl_2, 23 °C

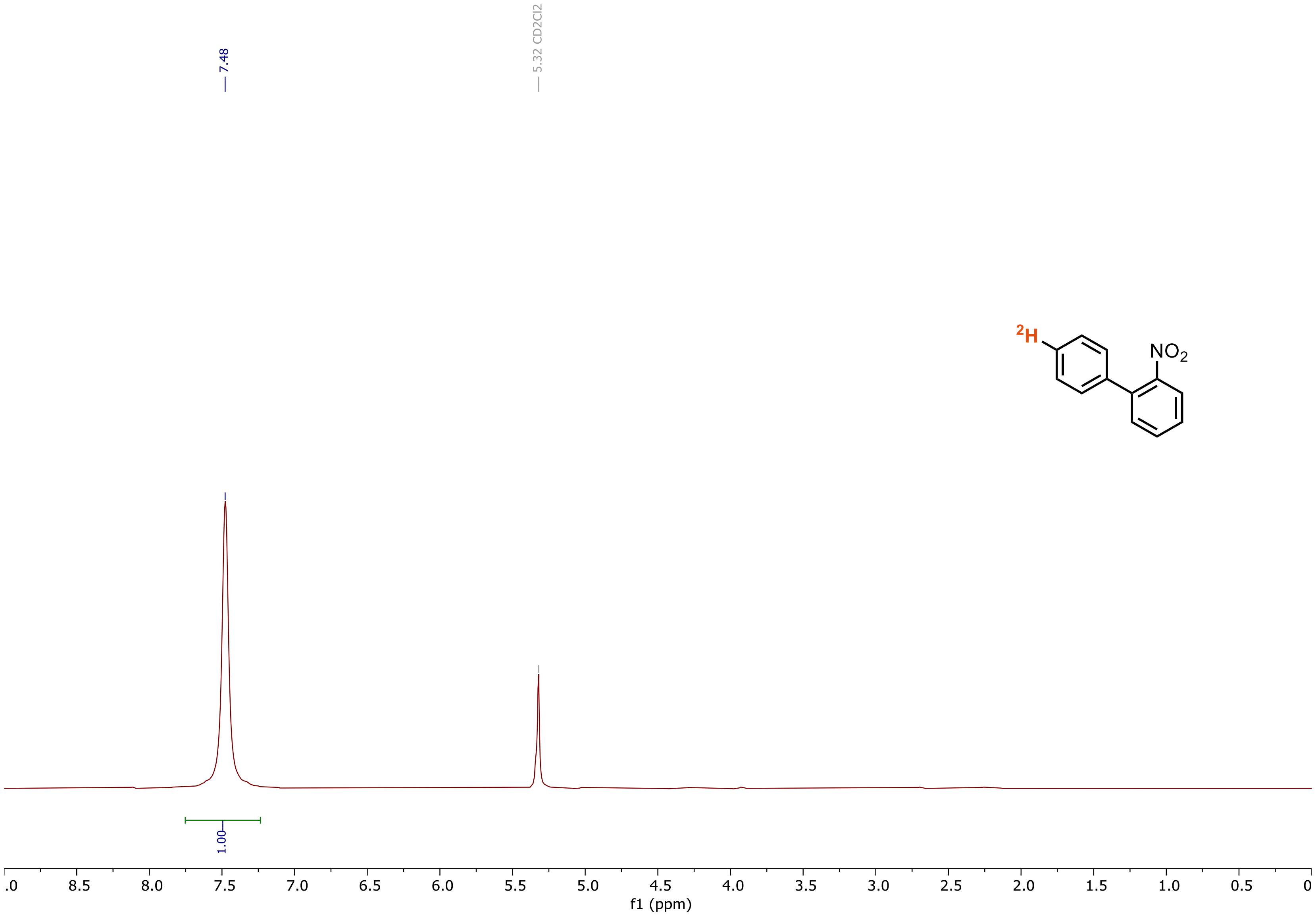

^{13}C NMR of 2-nitro-4'-[^{2}H]-biphenyl ([^{2}H]19)

CD_2Cl_2, 23 °C

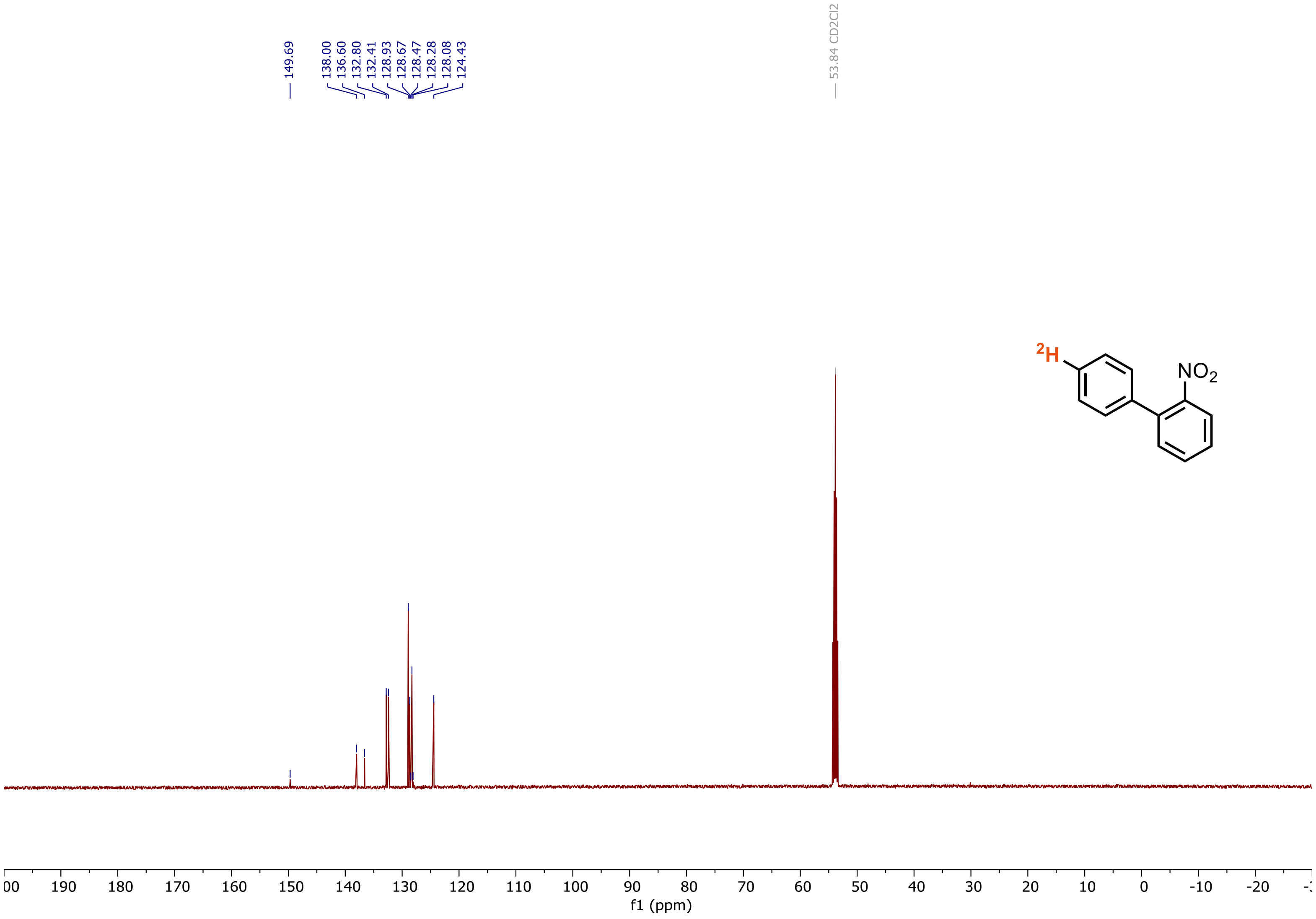

^{1}H NMR of [^{2}H]strychnine ([^{2}H]20)

CD_2Cl_2, 23 °C

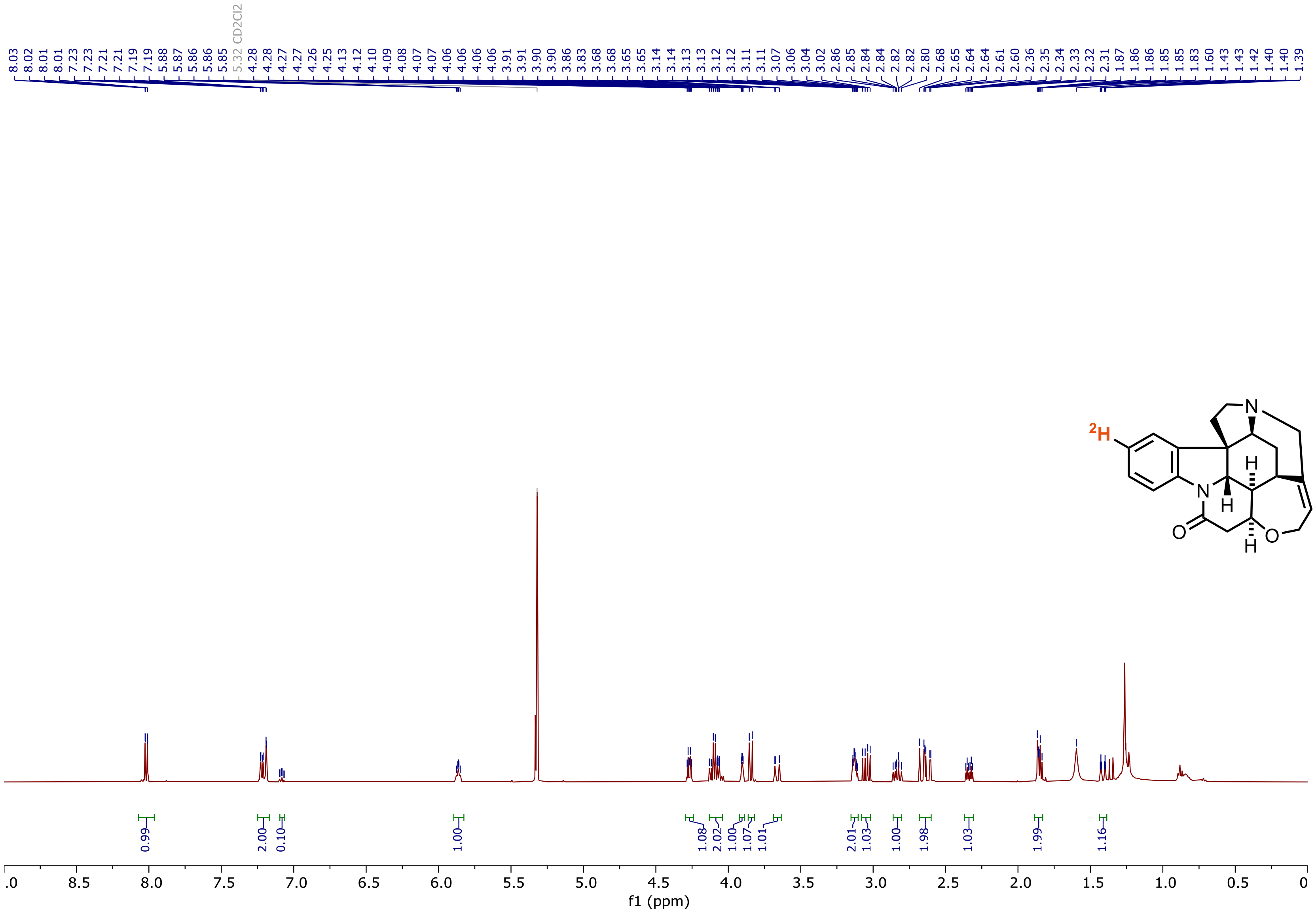

^{2}H NMR of [^{2}H]strychnine ([^{2}H]20)

CH_2Cl_2, 23 °C

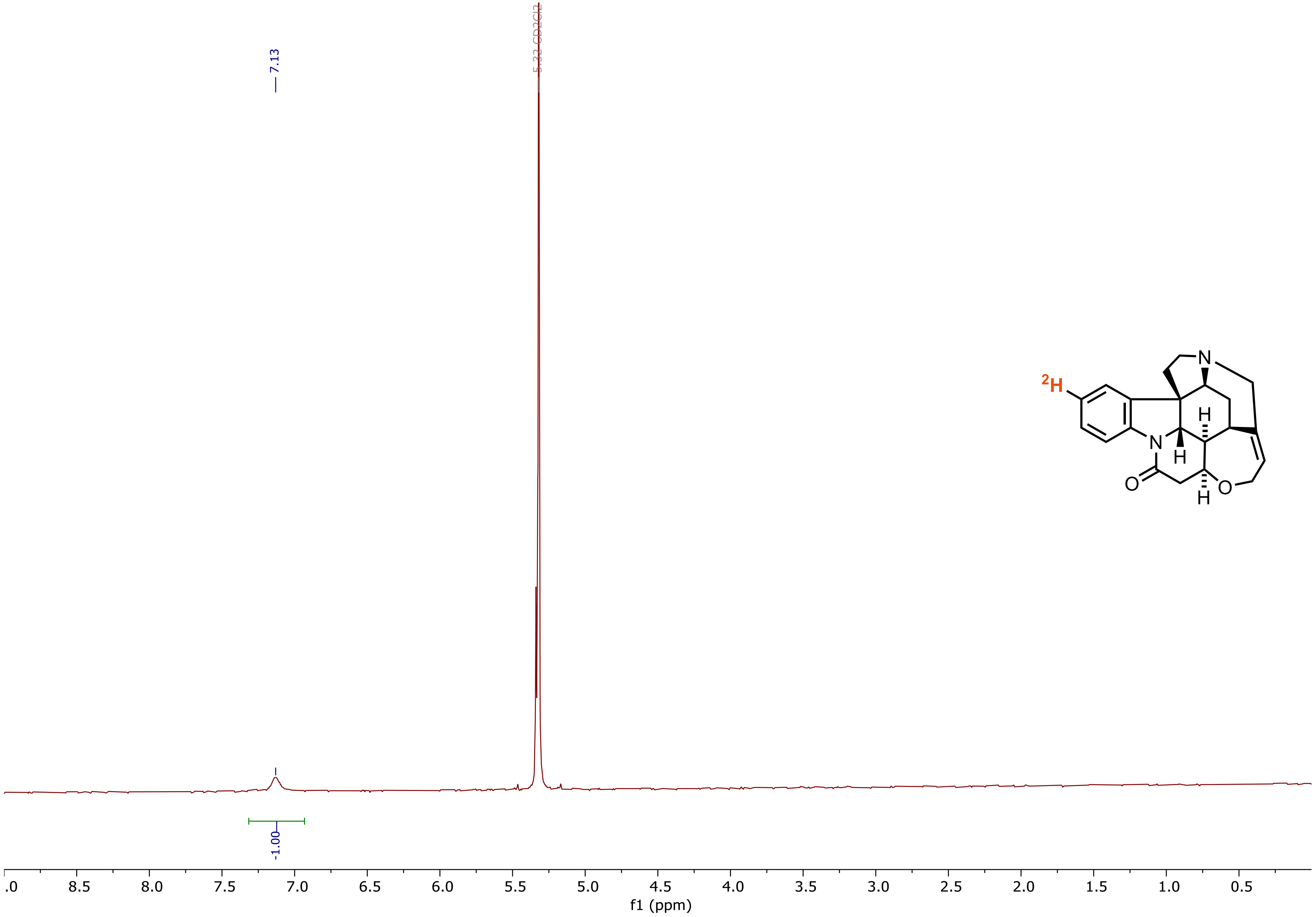

^{13}C NMR of [^2H]strychnine ([^2H]20)

CD_2Cl_2, 23 °C

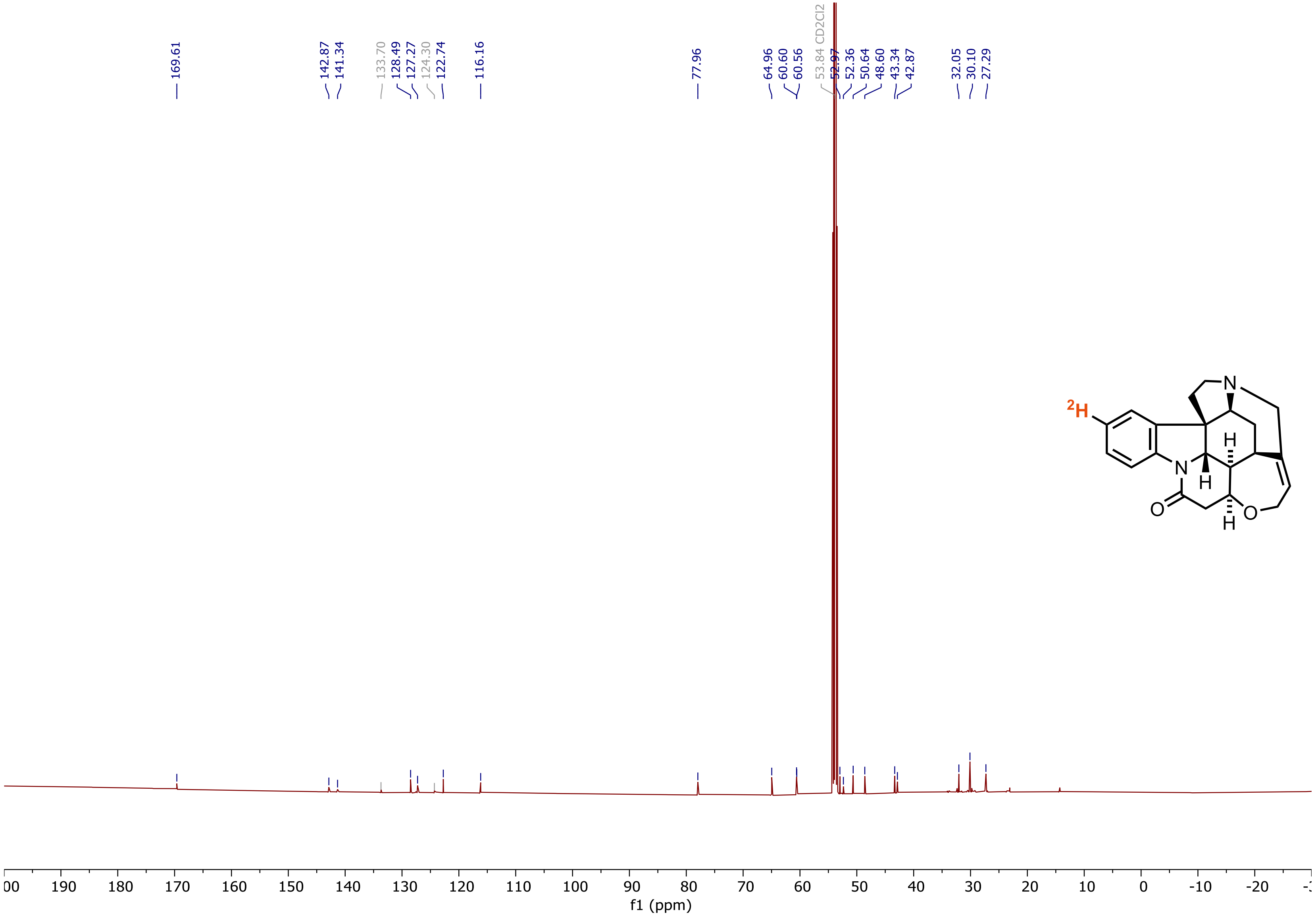

^{1}H NMR of [^{2}H]salicin pentahydrate ([^{2}H]21)

CD_3CN, 23 °C

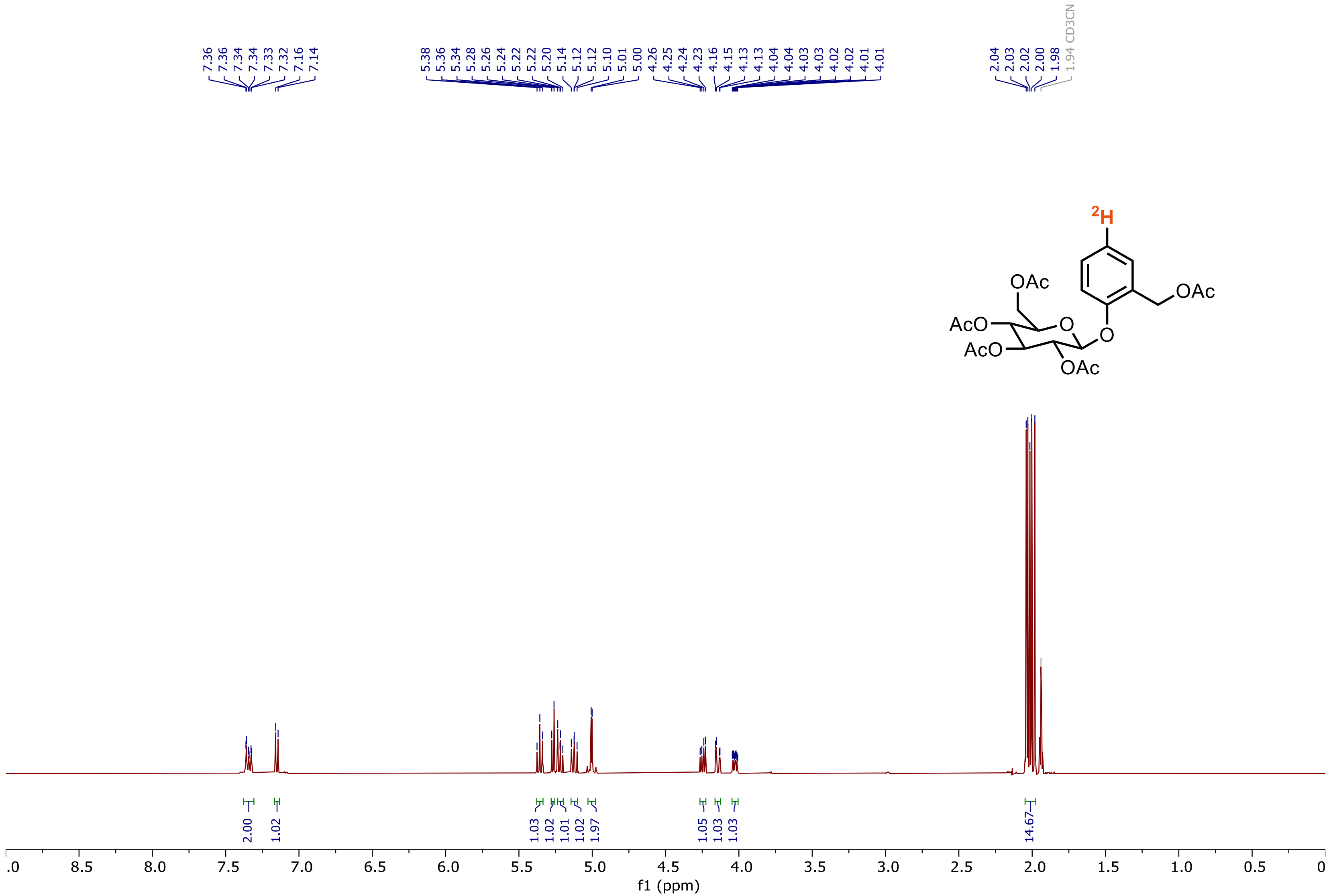

^{2}H NMR of [^{2}H]salicin pentahydrate ([^{2}H]21)

CH_3CN, 23 °C

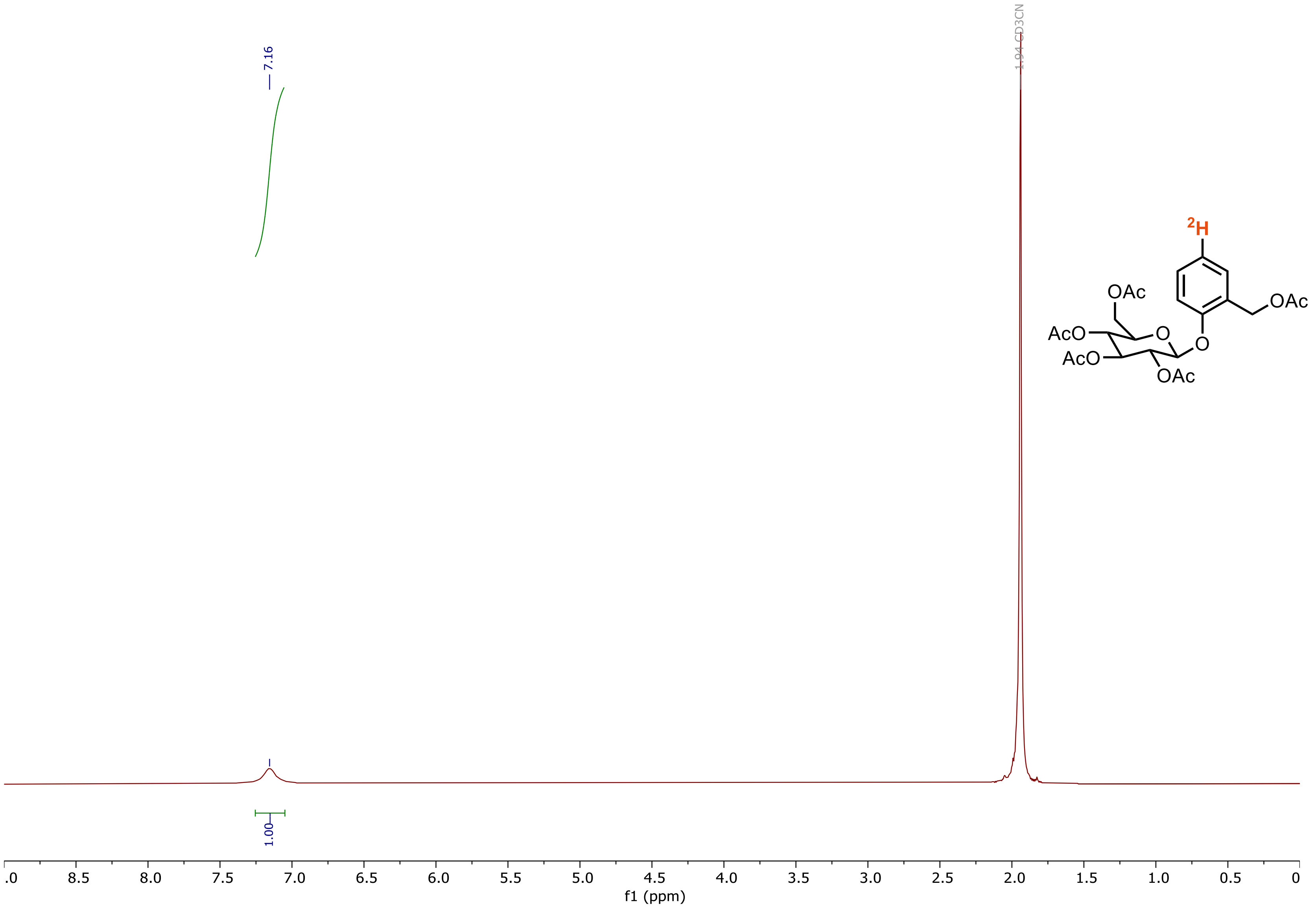

^{13}C NMR of [^{2}H]salicin pentahydrate ([^{2}H]21)

CD$_3$CN, 23 °C

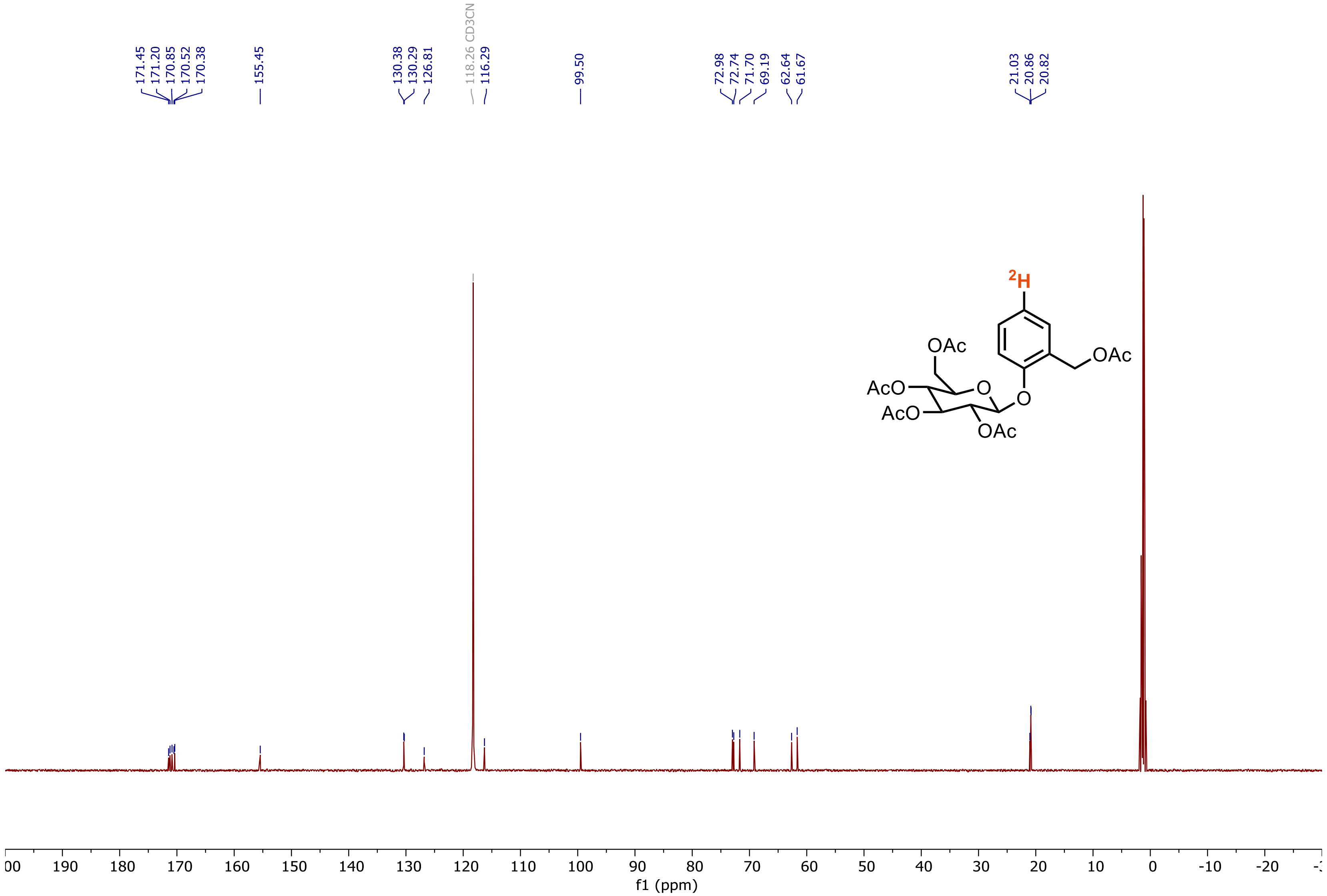

^{1}H NMR of 2-cyano-5-[^{2}H]-6-methoxyquinoline ([^{2}H]22)

CD_2Cl_2, 23 °C

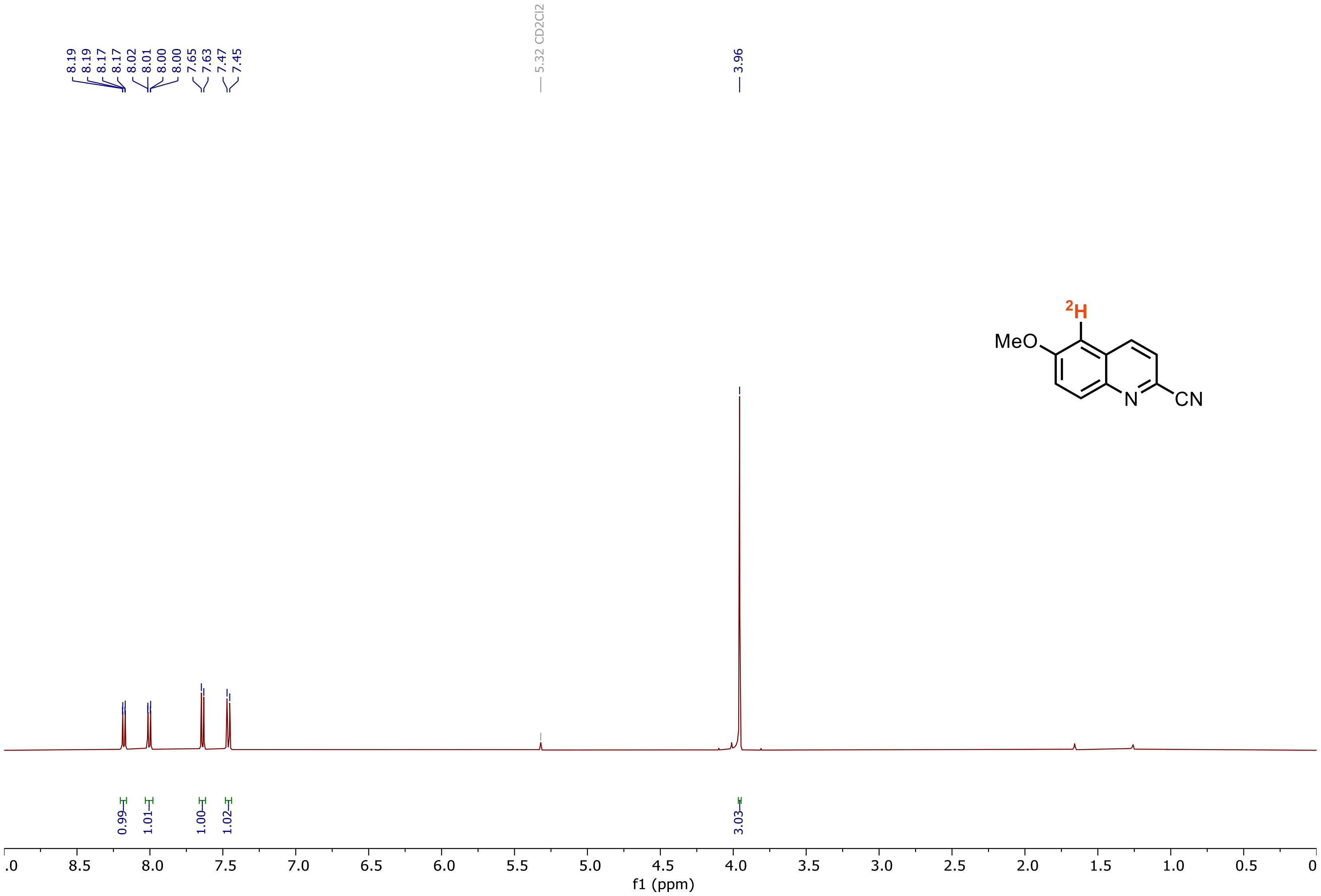

^{2}H NMR of 2-cyano-5-[^{2}H]-6-methoxyquinoline ([^{2}H]22)

CH_2Cl_2, 23 °C

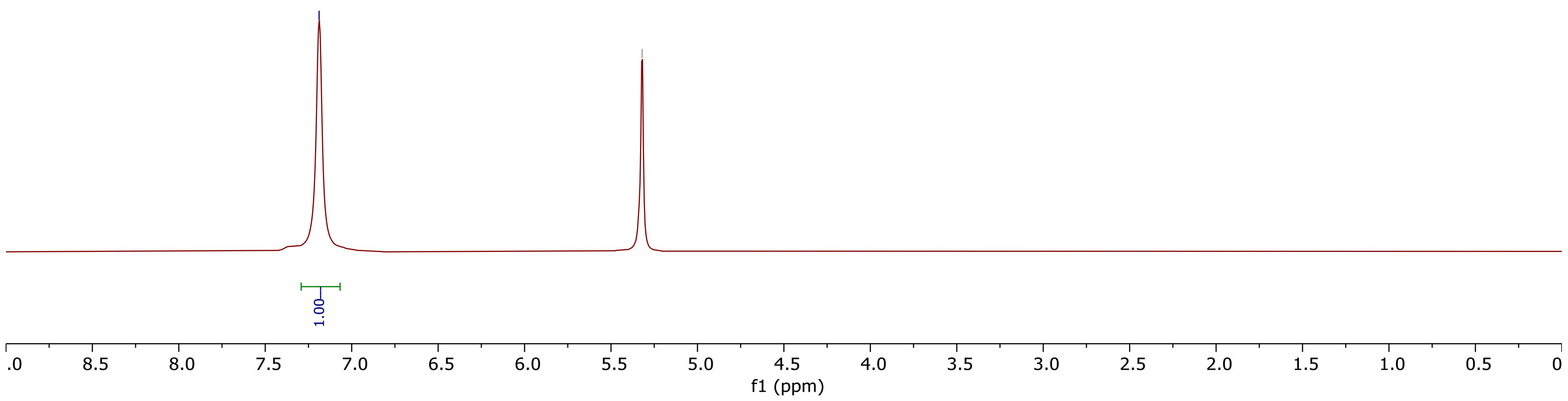

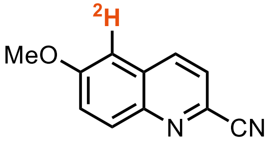

^{13}C NMR of 2-cyano-5-[^{2}H]-6-methoxyquinoline ([^{2}H]22)

CD_2Cl_2, 23 °C

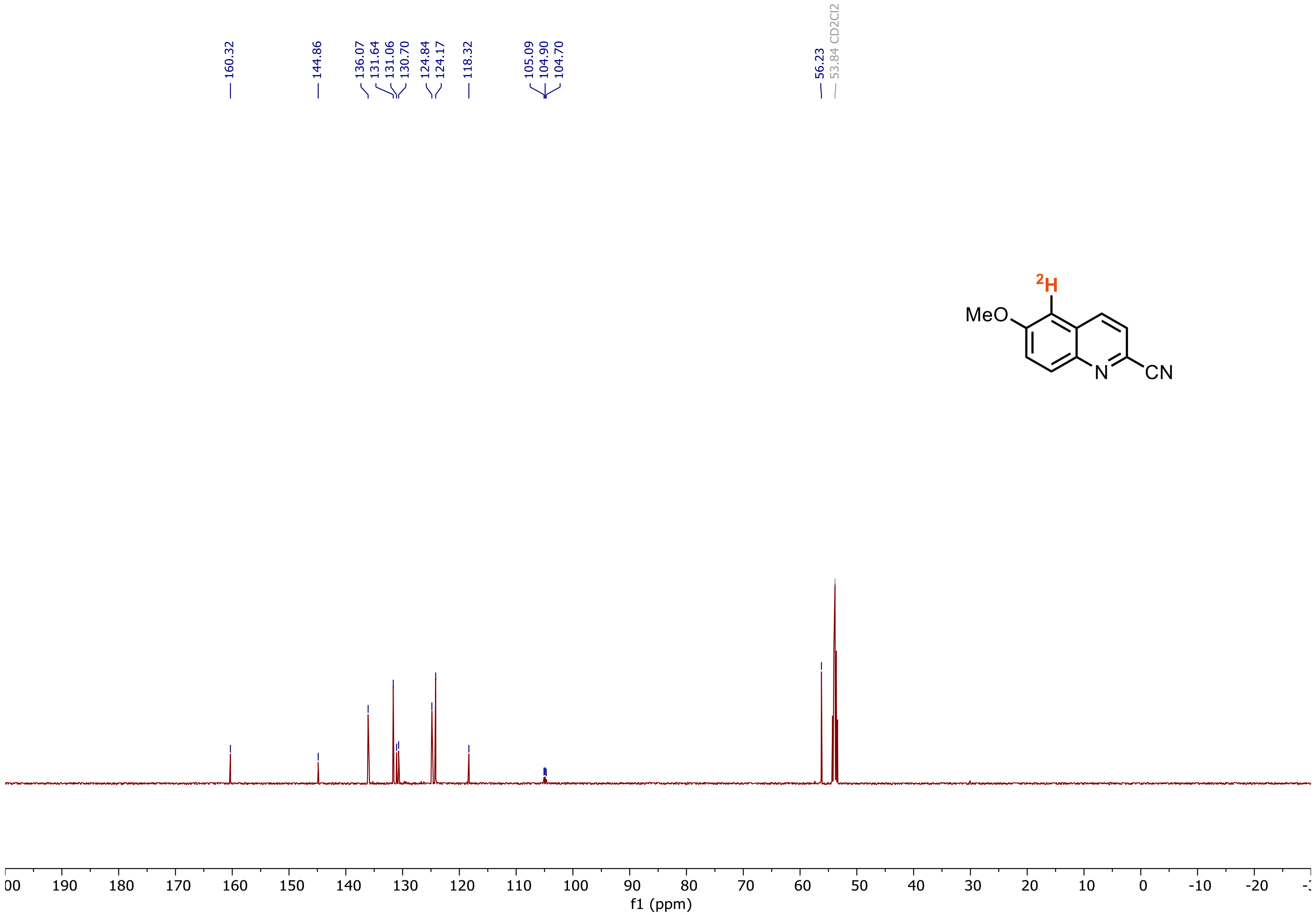

^{1}H NMR of 5-[^{2}H]-2-methoxybenzaldehyde ([^{2}H]23)

CD_2Cl_2, 23 °C

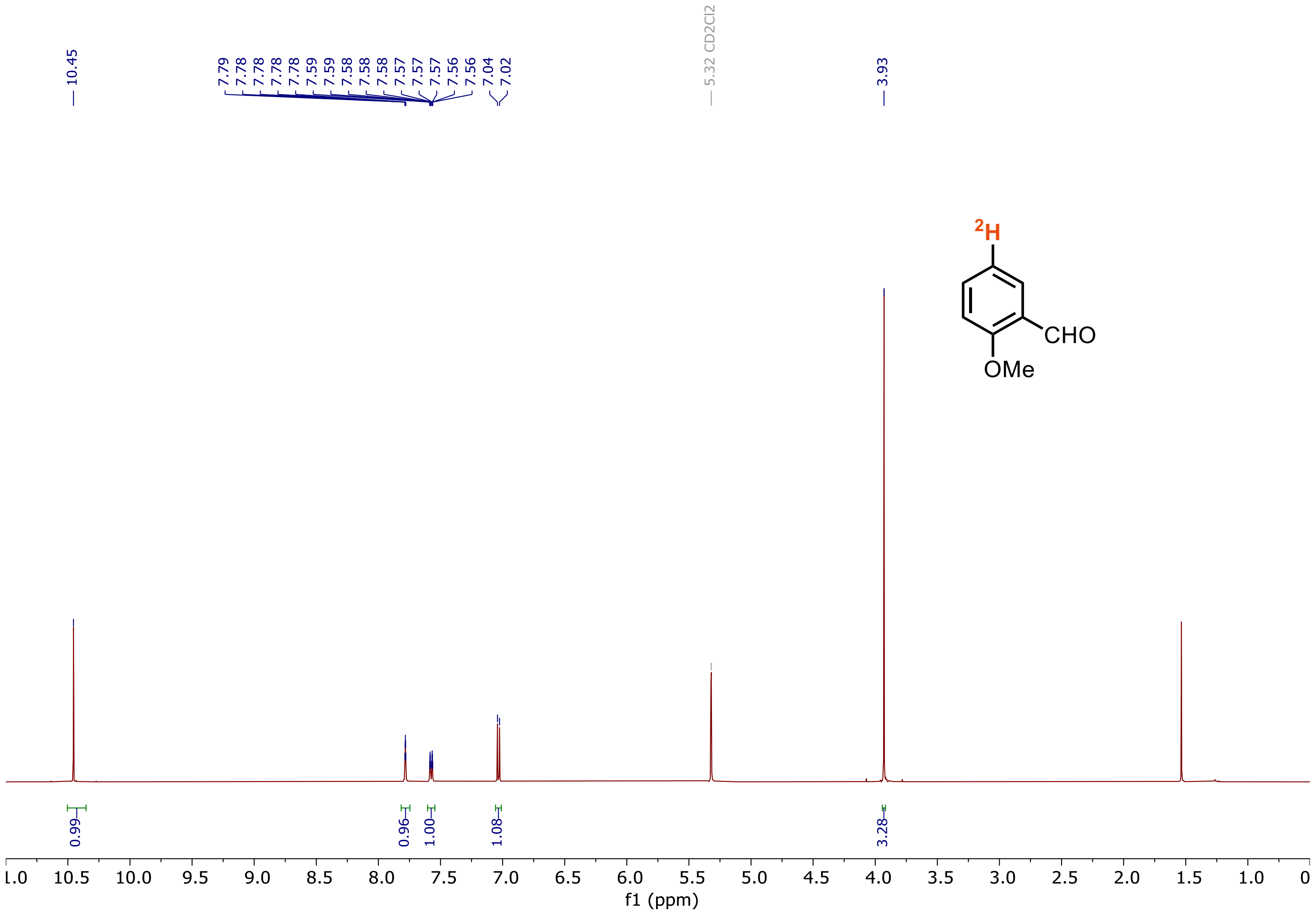

^{2}H NMR of 5-[^{2}H]-2-methoxybenzaldehyde ([^{2}H]23)

CH_2Cl_2, 23 °C

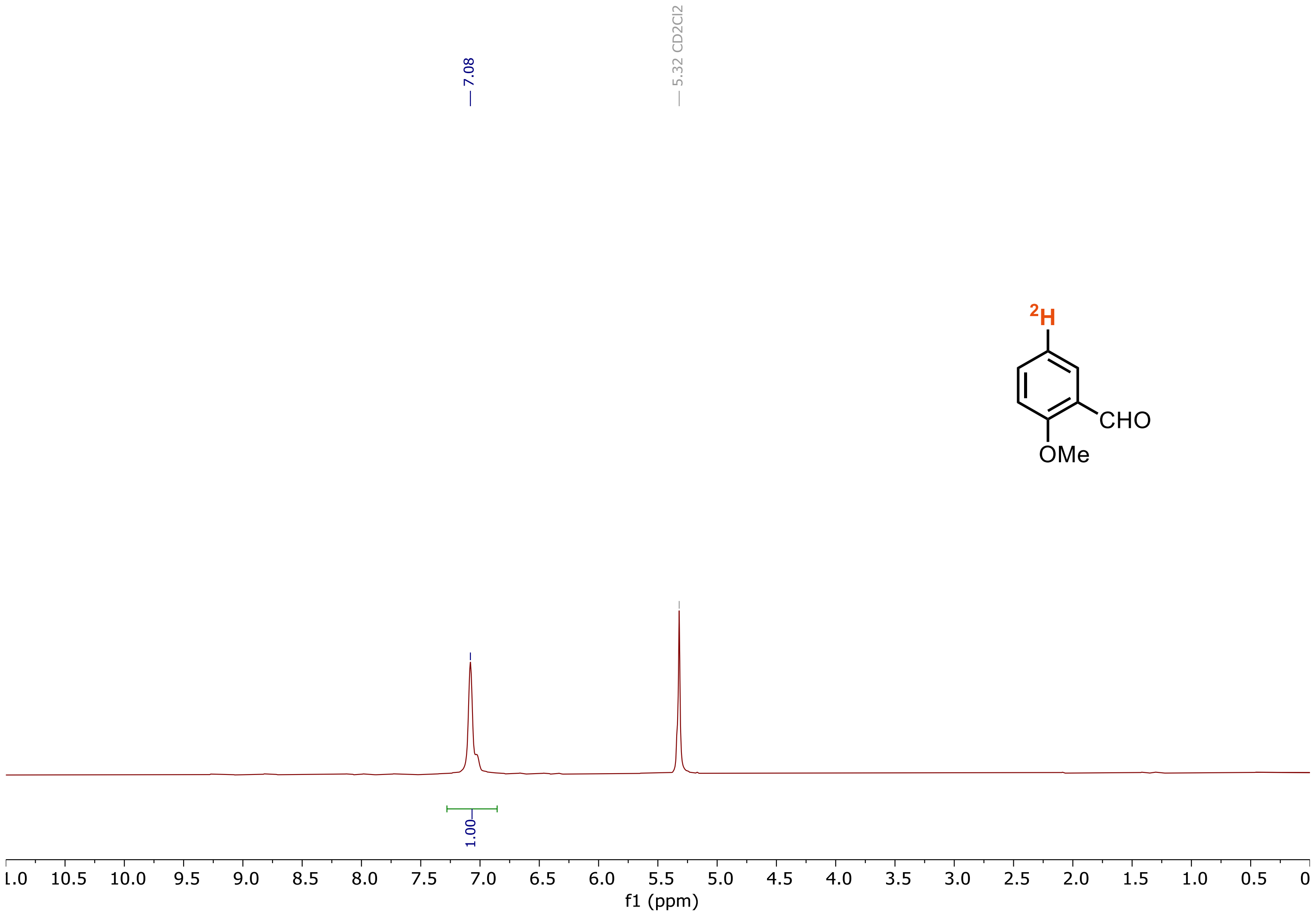

^{13}C NMR of 5-[^{2}H]-2-methoxybenzaldehyde ([^{2}H]23)

CD_2Cl_2, 23 °C

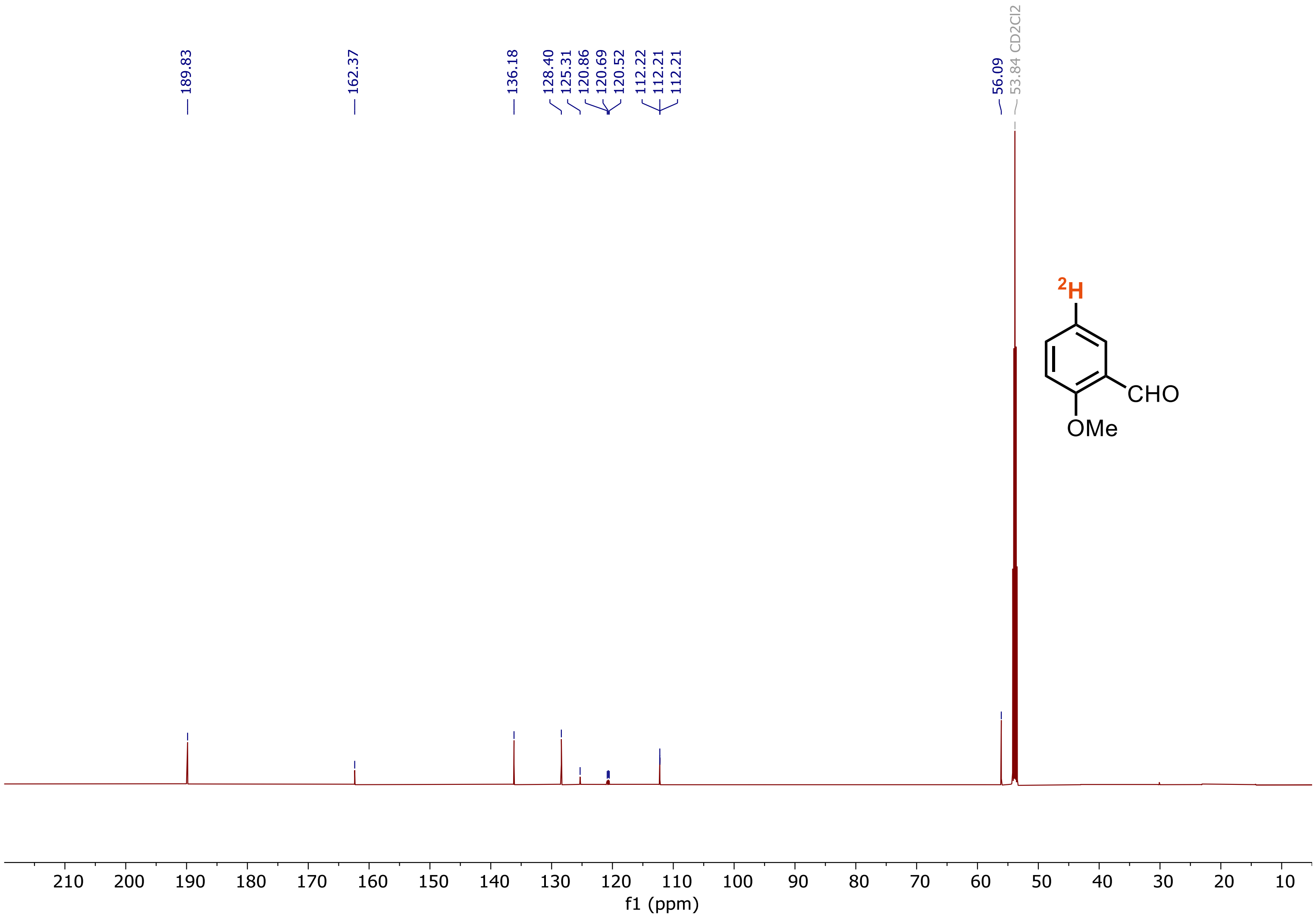

^{1}H NMR of 2,2,2-trichloroethyl (4-(4'-[^{2}H]phenyl)butyl)carbamate ([^{2}H]24)

CD_2Cl_2, 23 °C

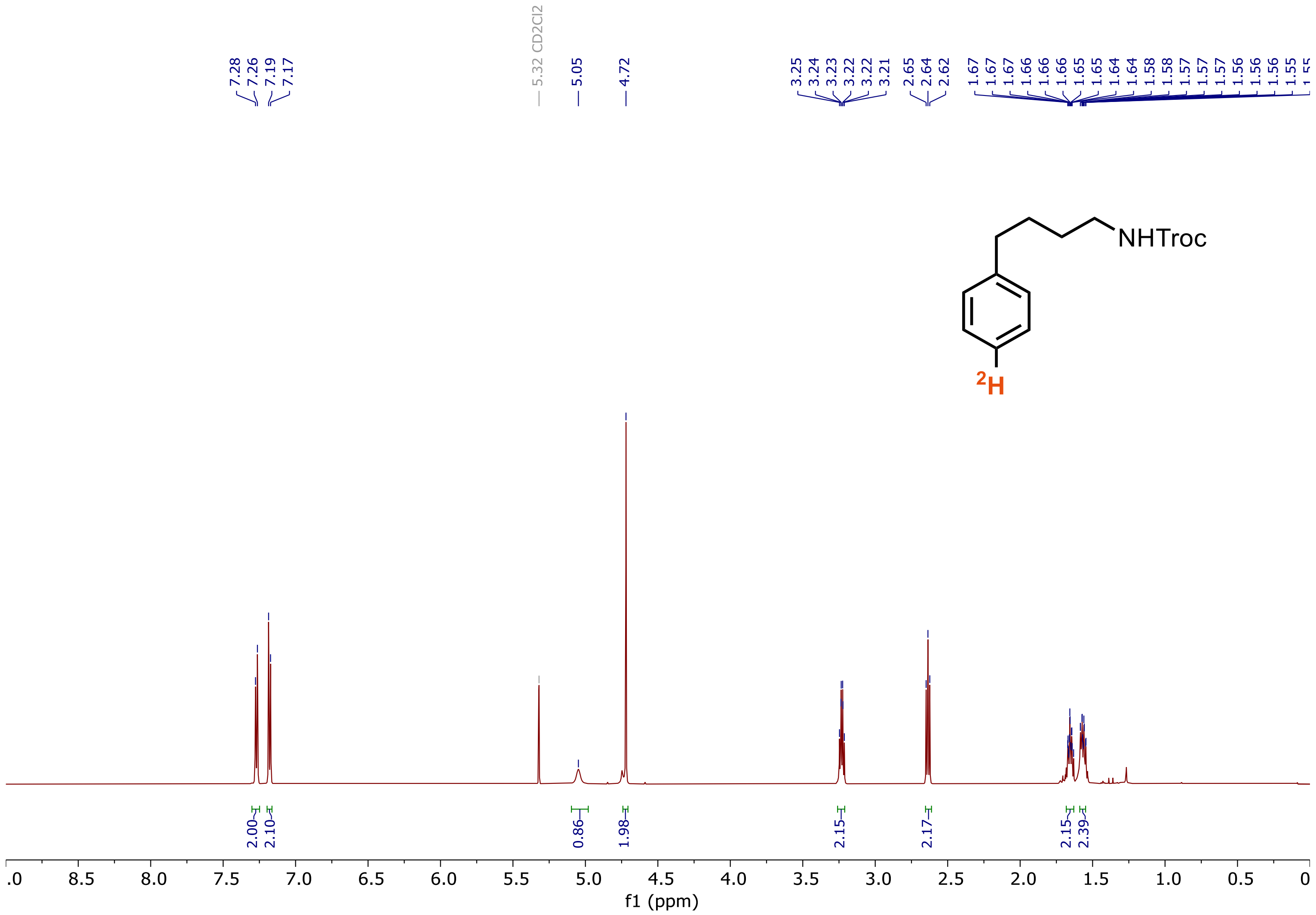

^{2}H NMR of 2,2,2-trichloroethyl (4-(4'-[^{2}H]phenyl)butyl)carbamate ([^{2}H]24)

CH_2Cl_2, 23 °C

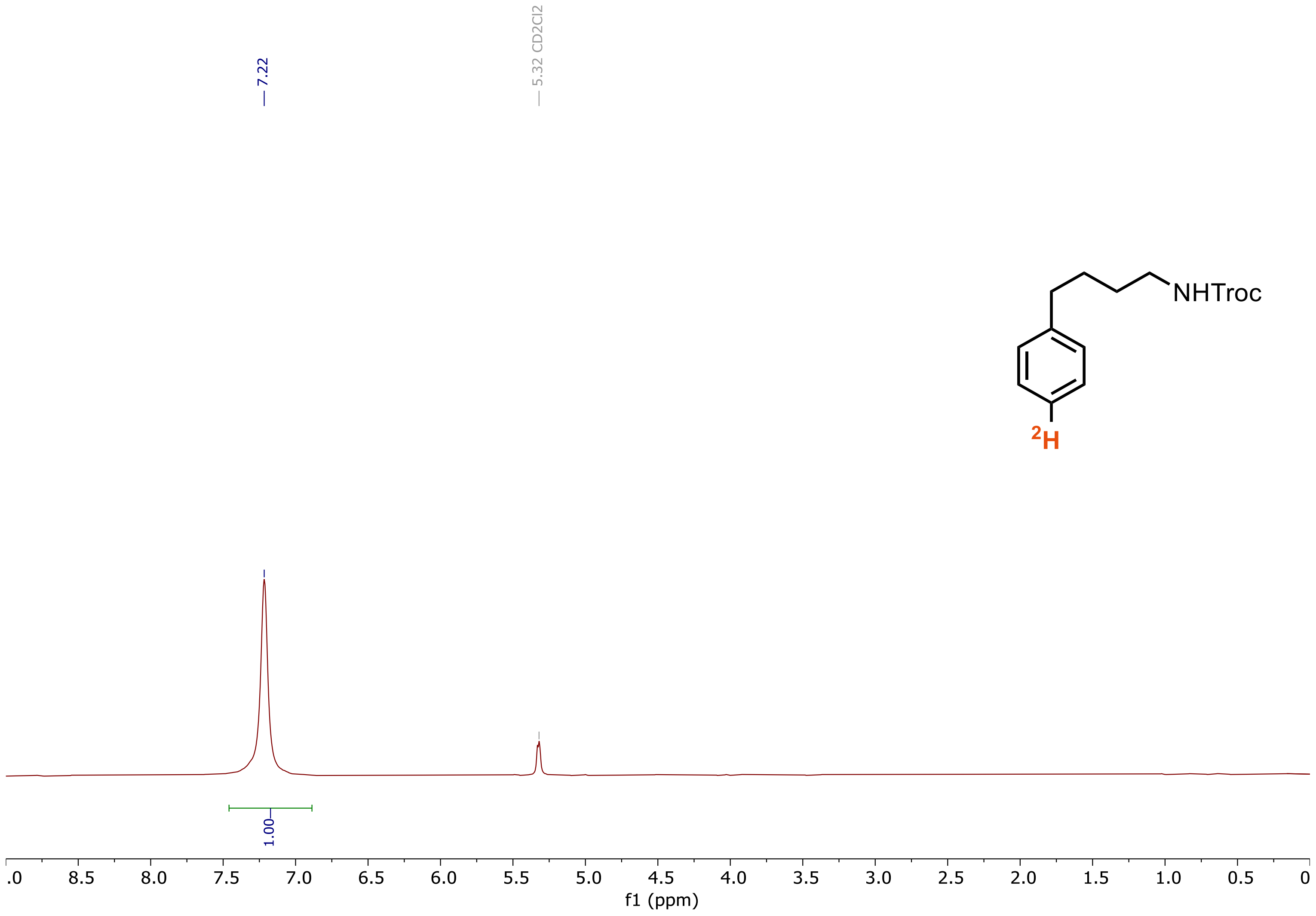

^{13}C NMR of 2,2,2-trichloroethyl (4-(4'-[^{2}H]phenyl)butyl)carbamate ([^{2}H]24)

CD_2Cl_2, 23 °C

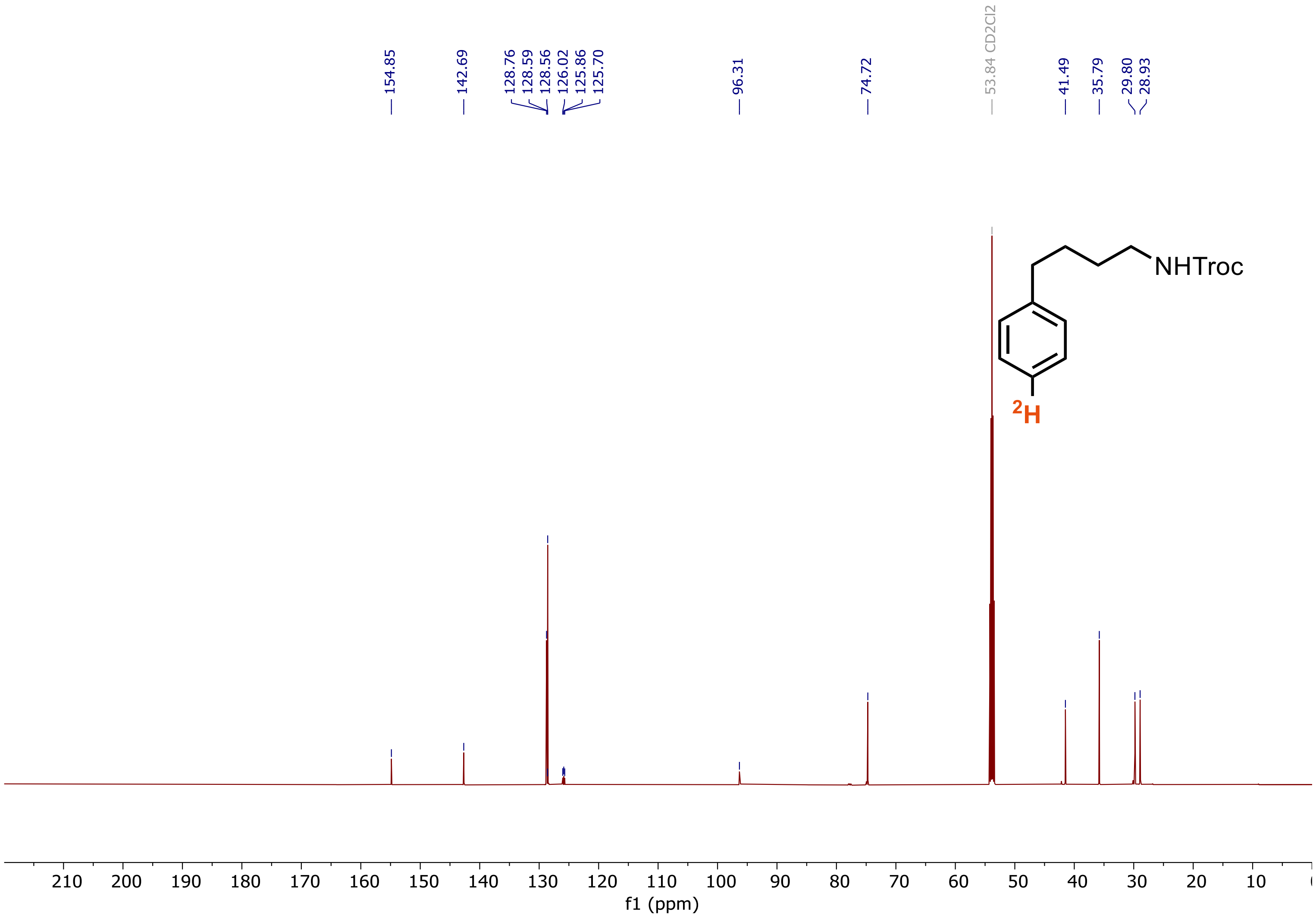

^{1}H NMR of [^{2}H]boscalid ([^{2}H]25)

CD_2Cl_2, 23 °C

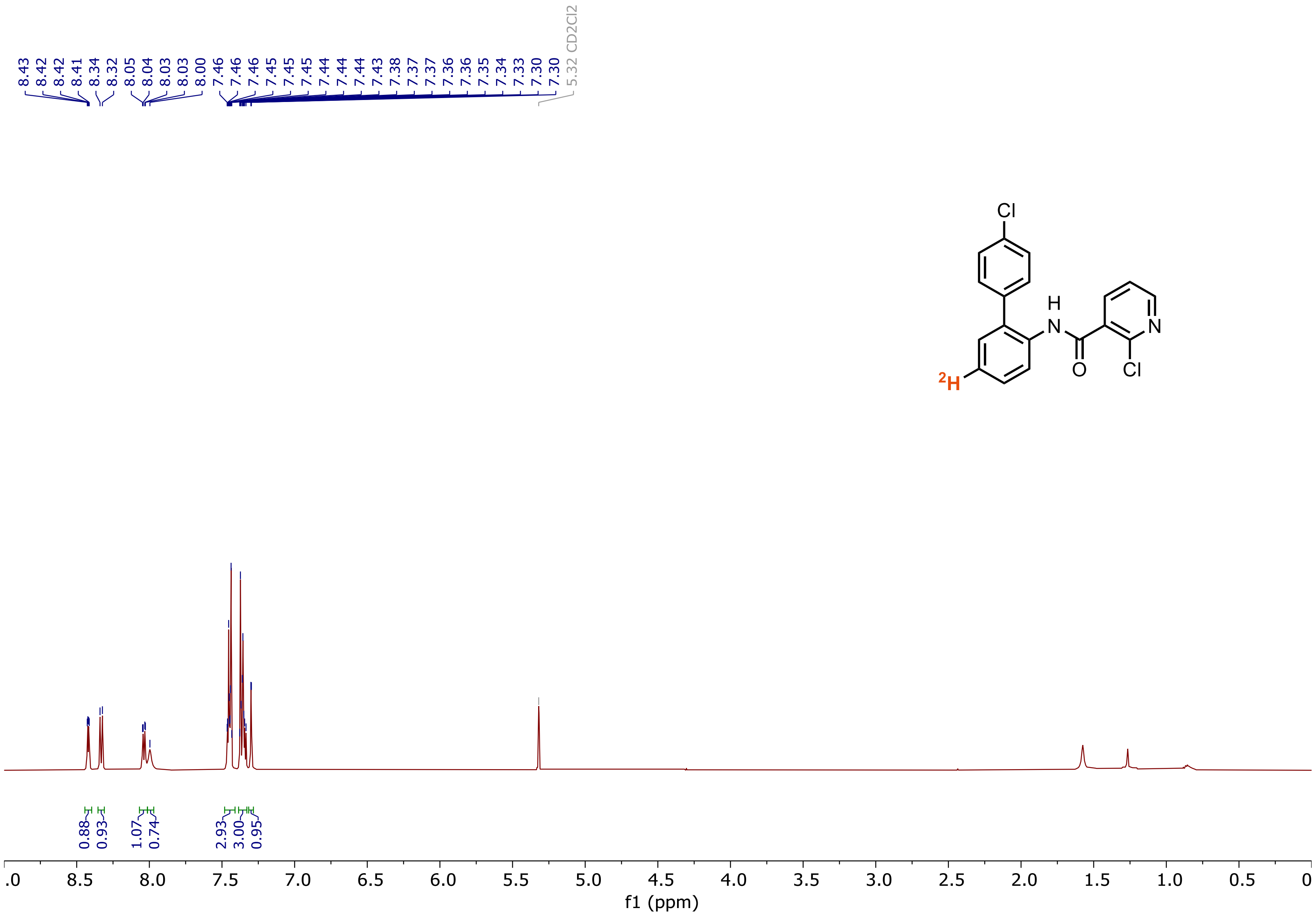

^{2}H NMR of [^{2}H]boscalid ([^{2}H]25)

CH_2Cl_2, 23 °C

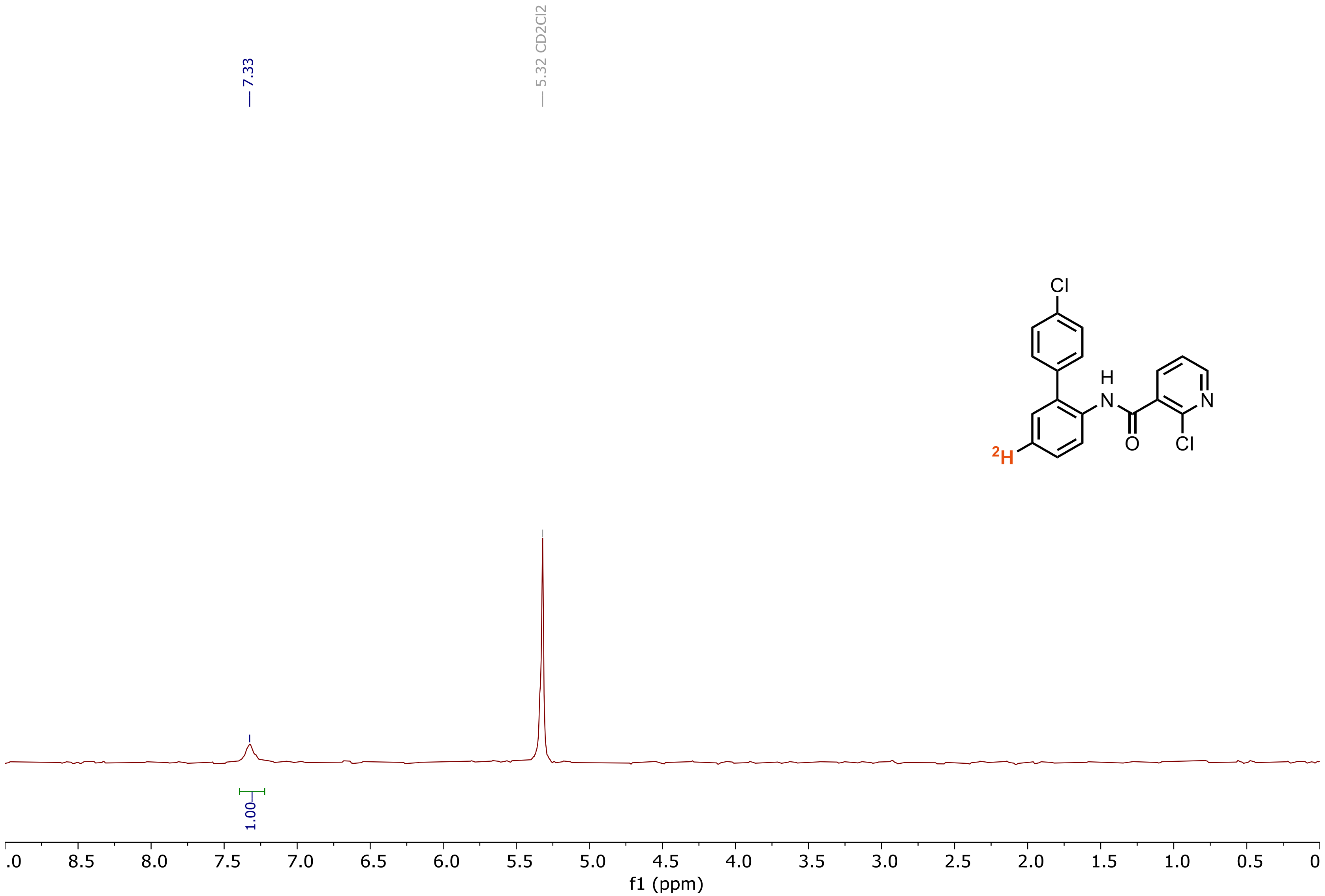

^{13}C NMR of [^{2}H]boscalid ([^{2}H]25)

CD_2Cl_2, 23 °C

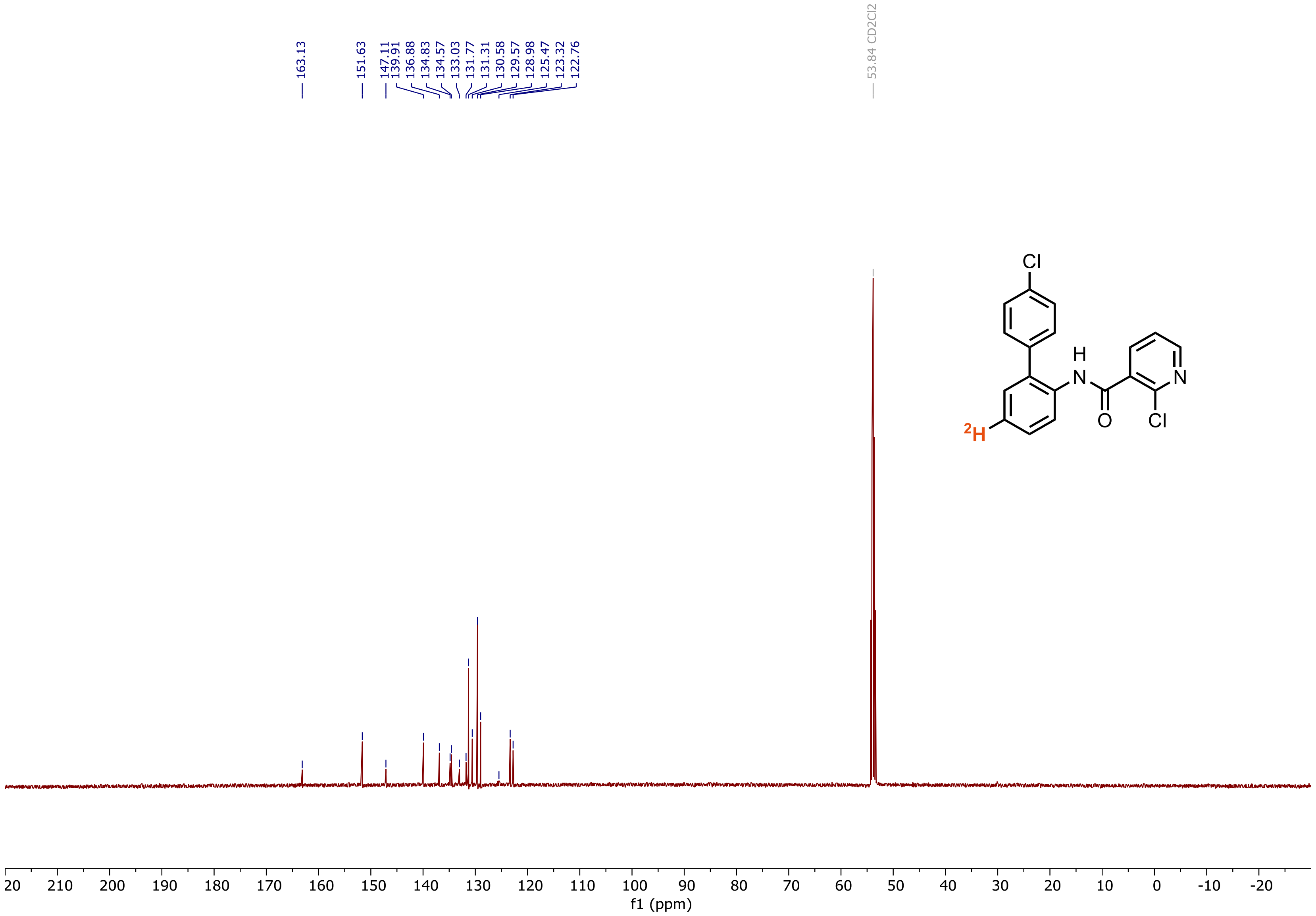

^{1}H NMR of [^{2}H]LHVS ([^{2}H]26)

CD_2Cl_2, 23 °C

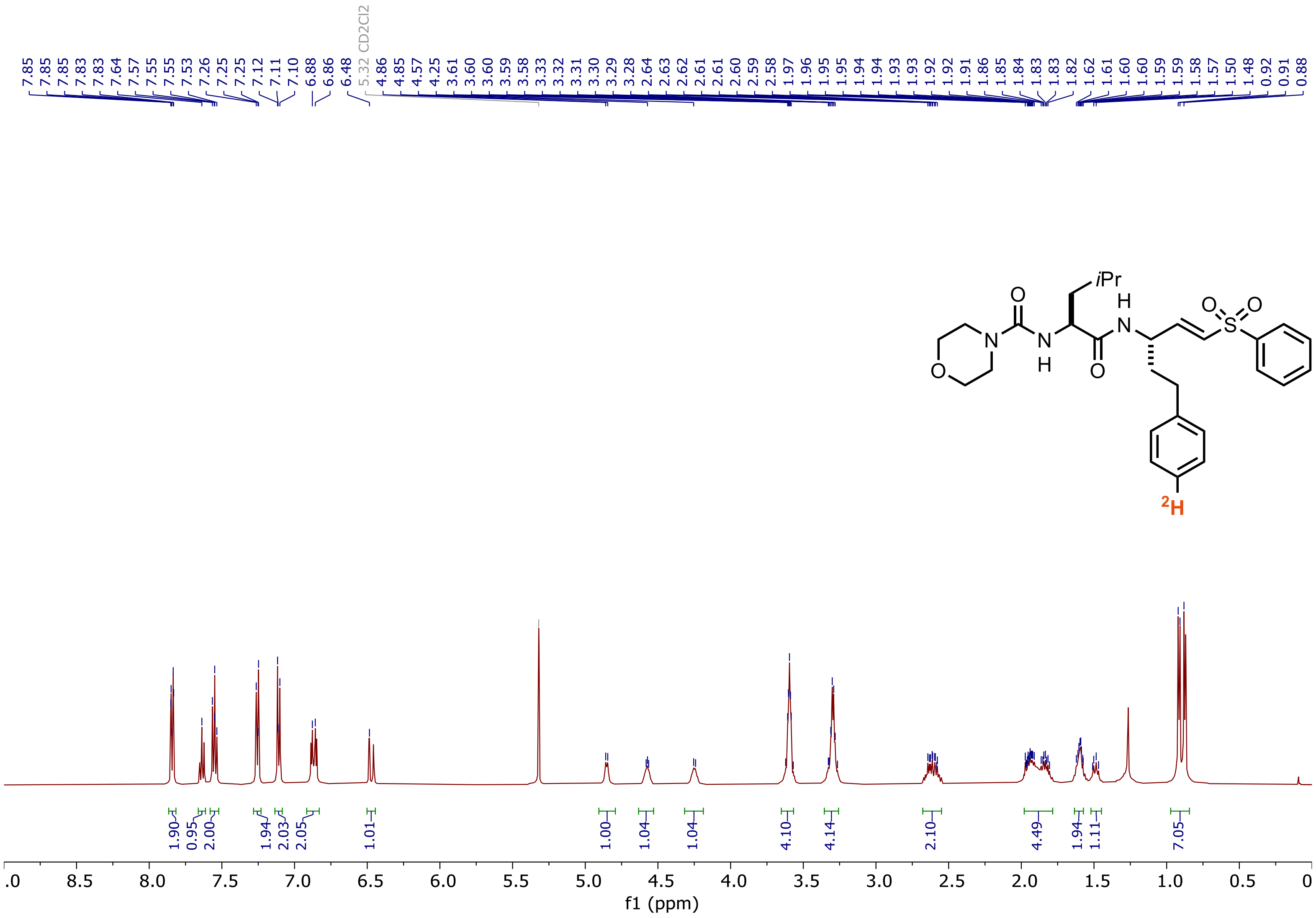

^{2}H NMR of [^{2}H]LHVS ([^{2}H]26)

CH_2Cl_2, 23 °C

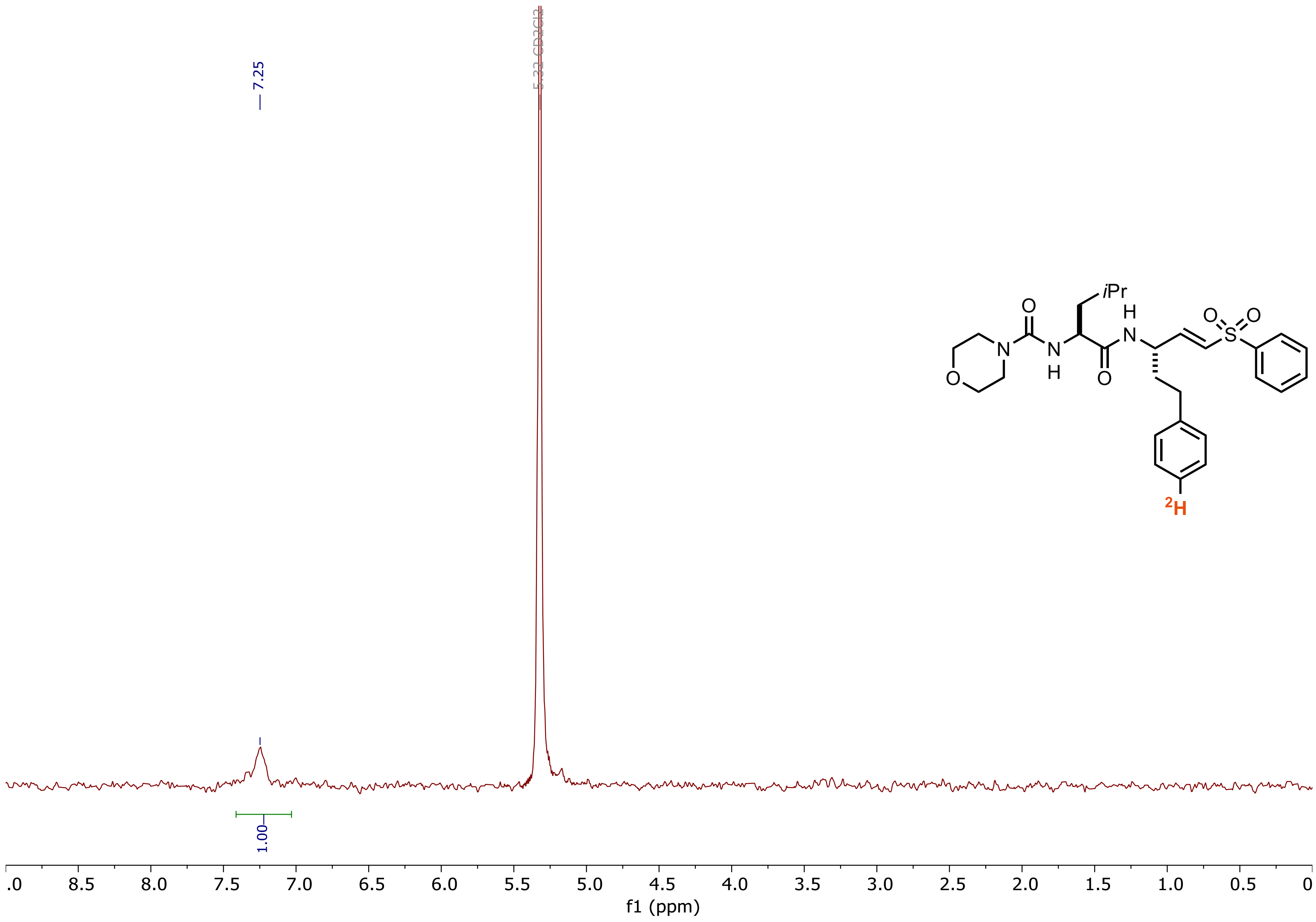

^{13}C NMR of [^{2}H]LHVS ([^{2}H]26)

CD_2Cl_2, 23 °C

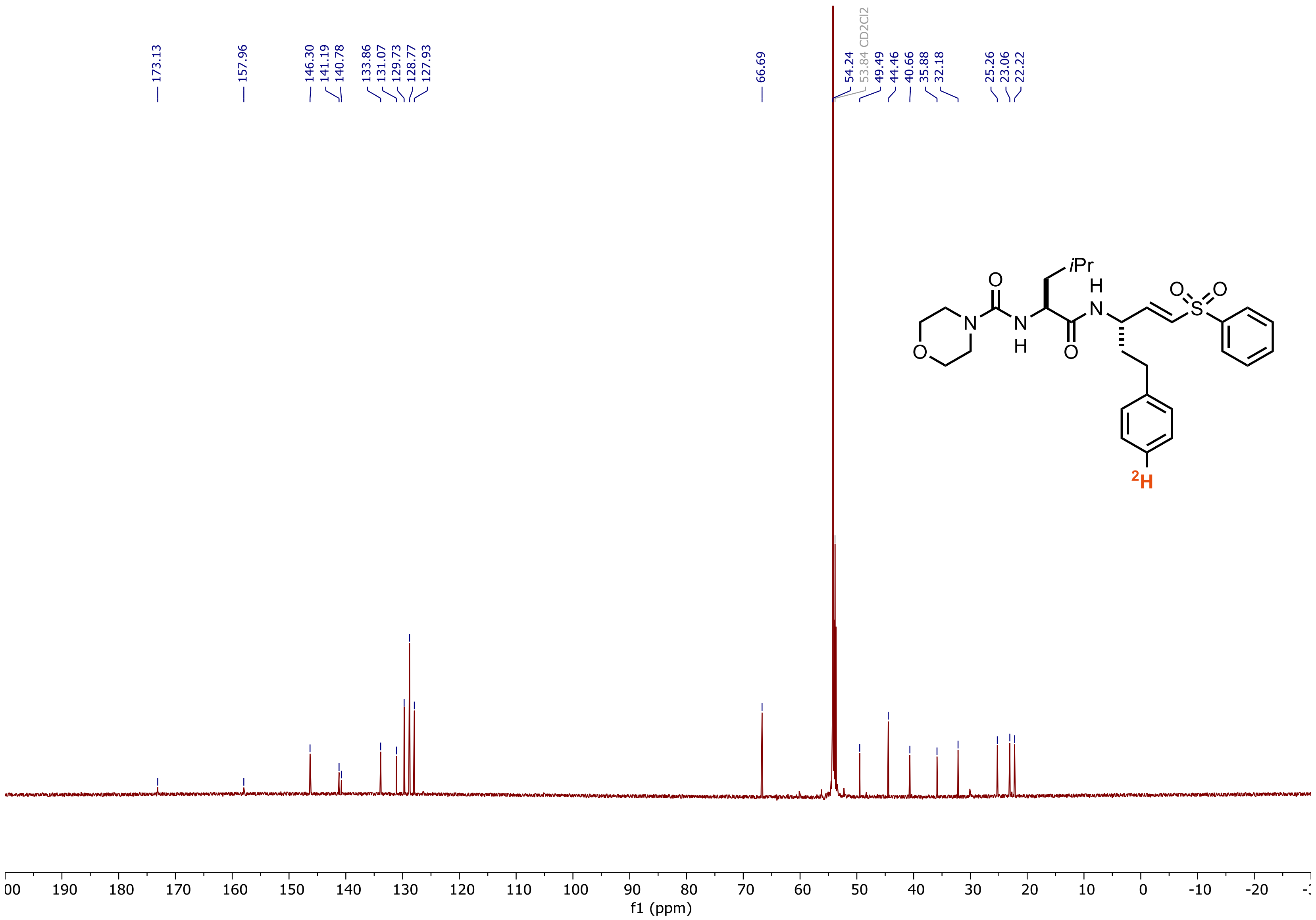

^{1}H NMR of [^{2}H]fenofibrate ([^{2}H]28)

$CDCl_3$, 23 °C

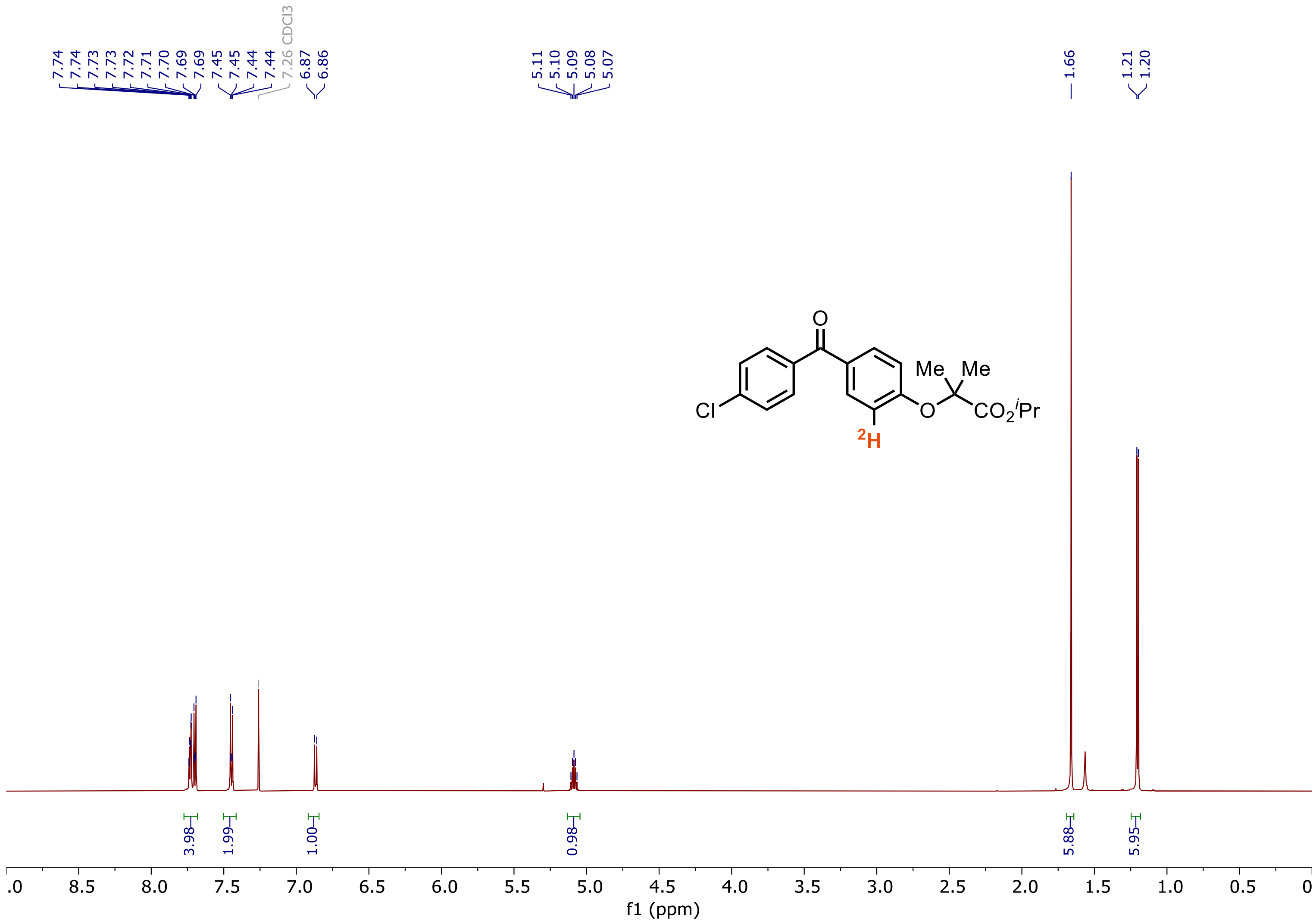

^{2}H NMR of [^{2}H]fenofibrate ([^{2}H]28)

CHCl$_3$, 23 °C

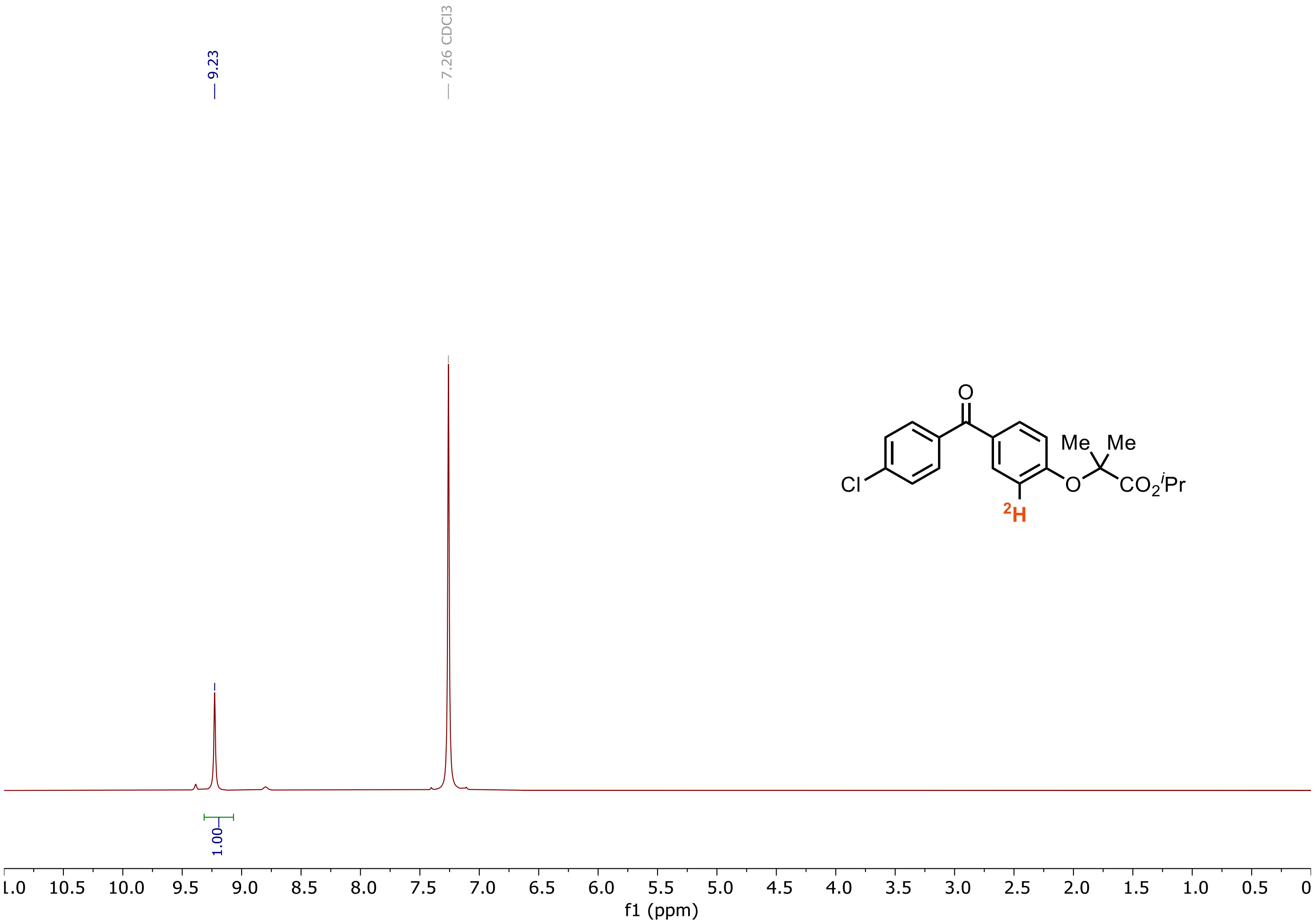

^{13}C NMR of [^{2}H]fenofibrate ([^{2}H]28)

$CDCl_3$, 23 °C

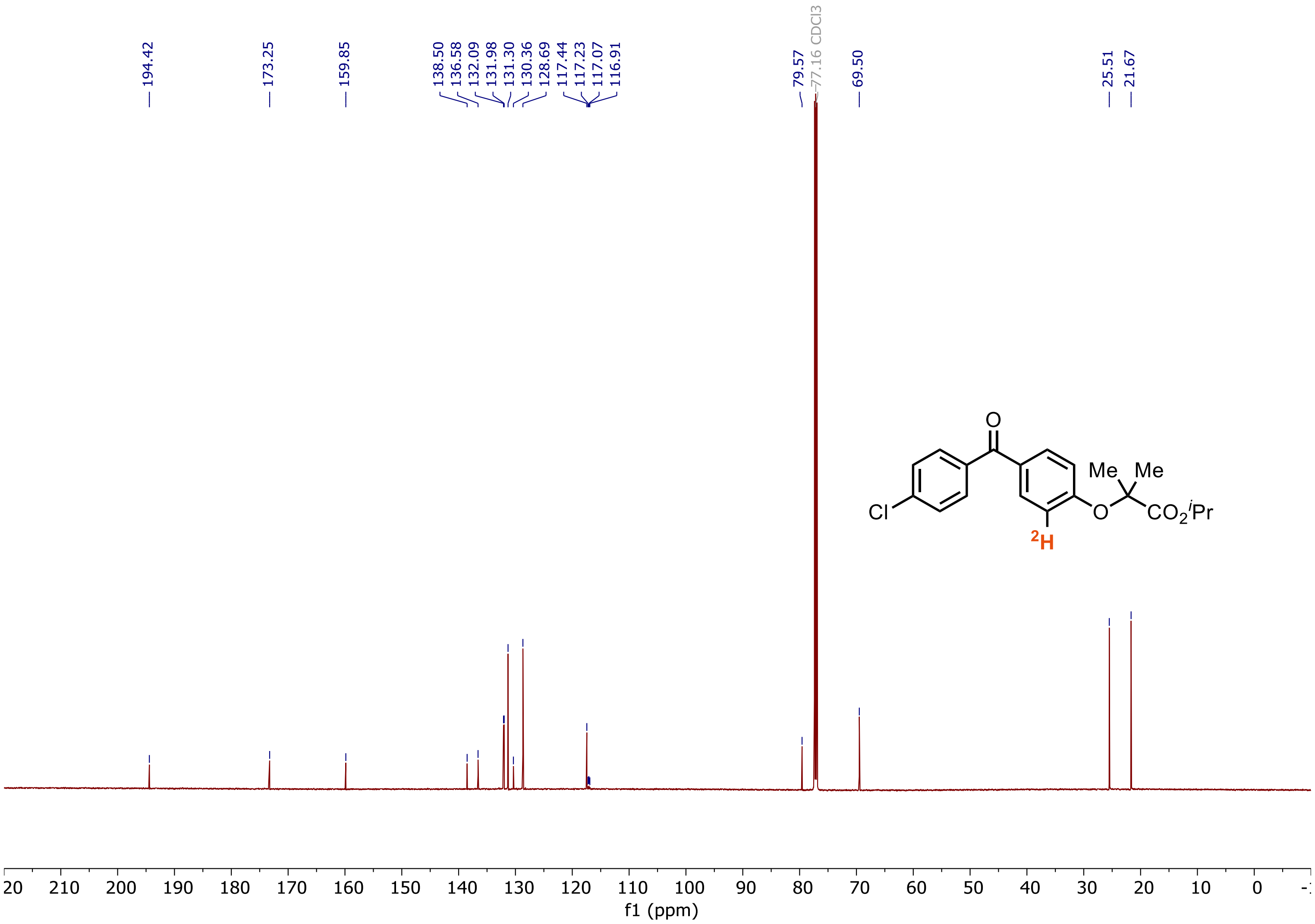

^{1}H NMR of [^{2}H]etofenprox ([^{2}H]29)

CD_3CN, 23 °C

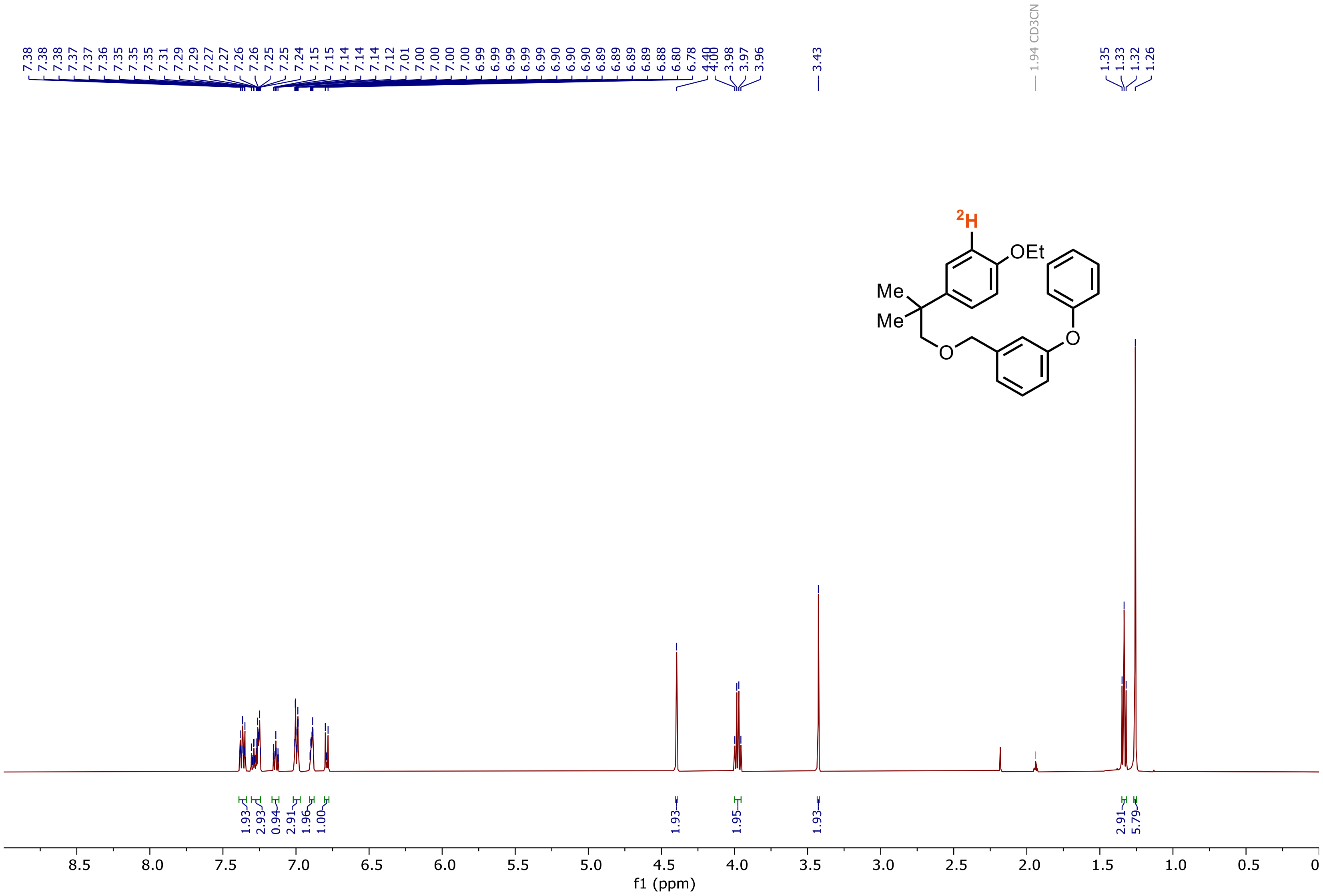

^{2}H NMR of [^{2}H]etofenprox ([^{2}H]29)

CH_3CN, 23 °C

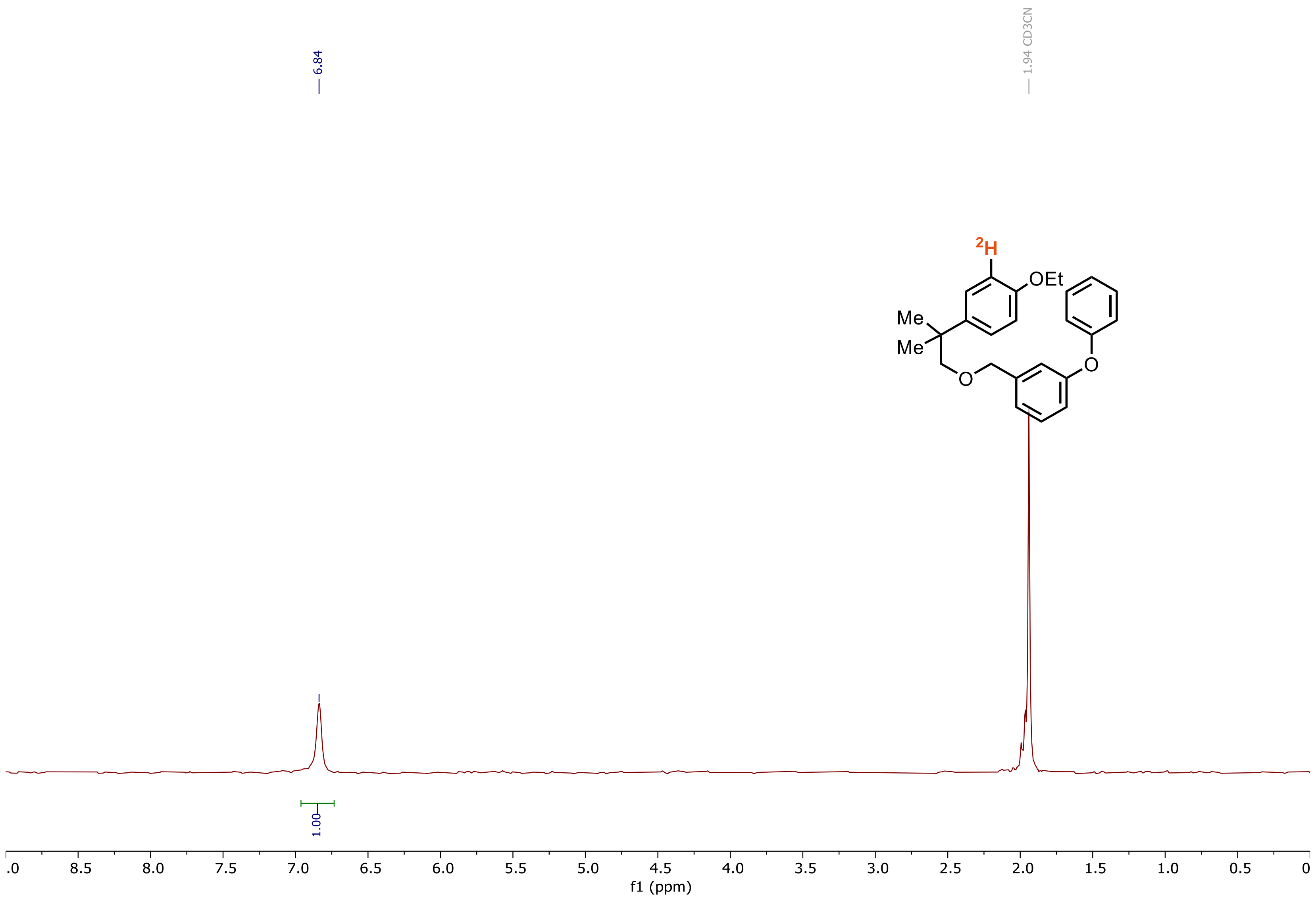

^{13}C NMR of [^{2}H]etofenprox ([^{2}H]29)

CD_3CN, 23 °C

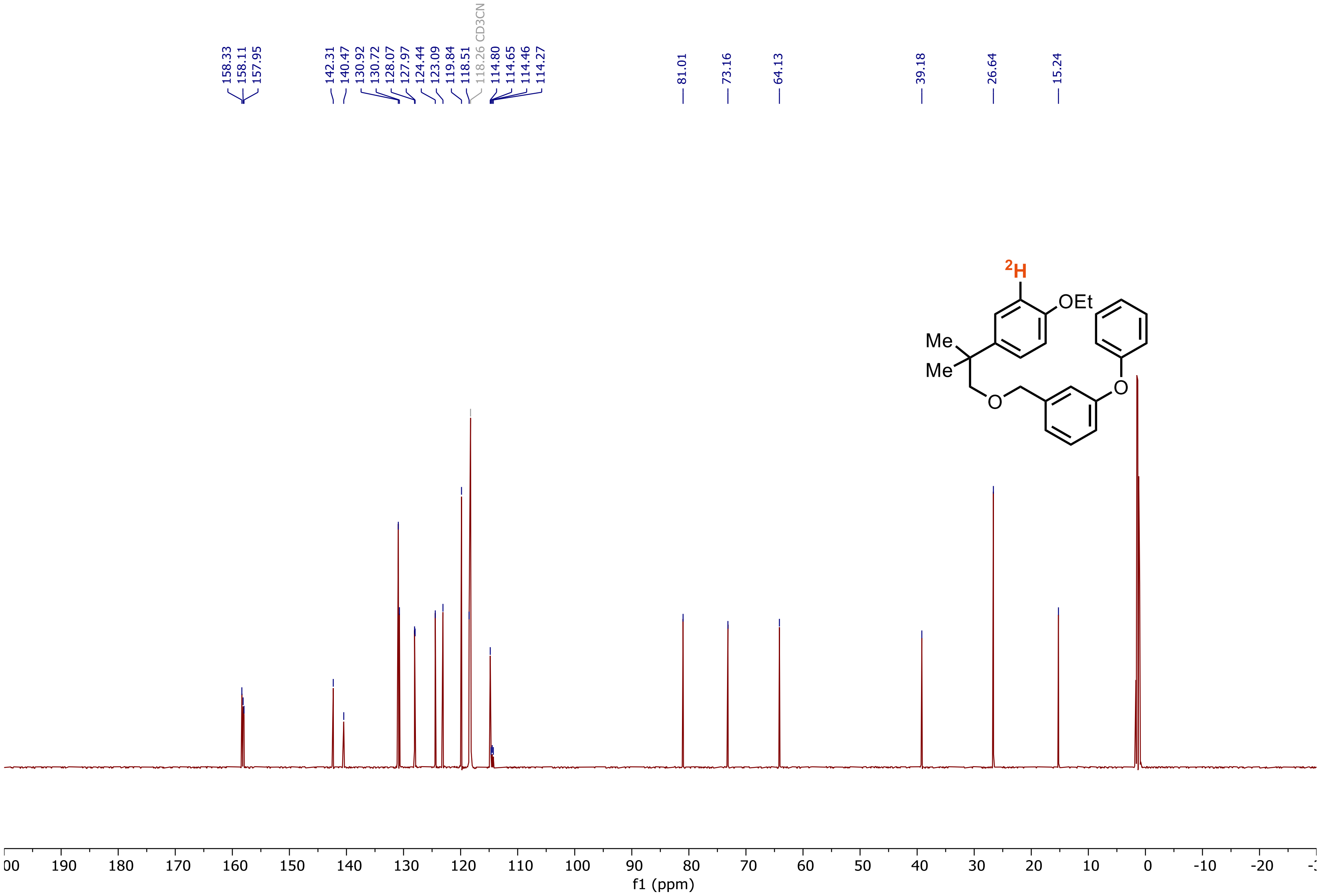

^{1}H NMR of [^{2}H$_2$]etofenprox ([^{2}H$_2$]29)

CD_3CN, 23 °C

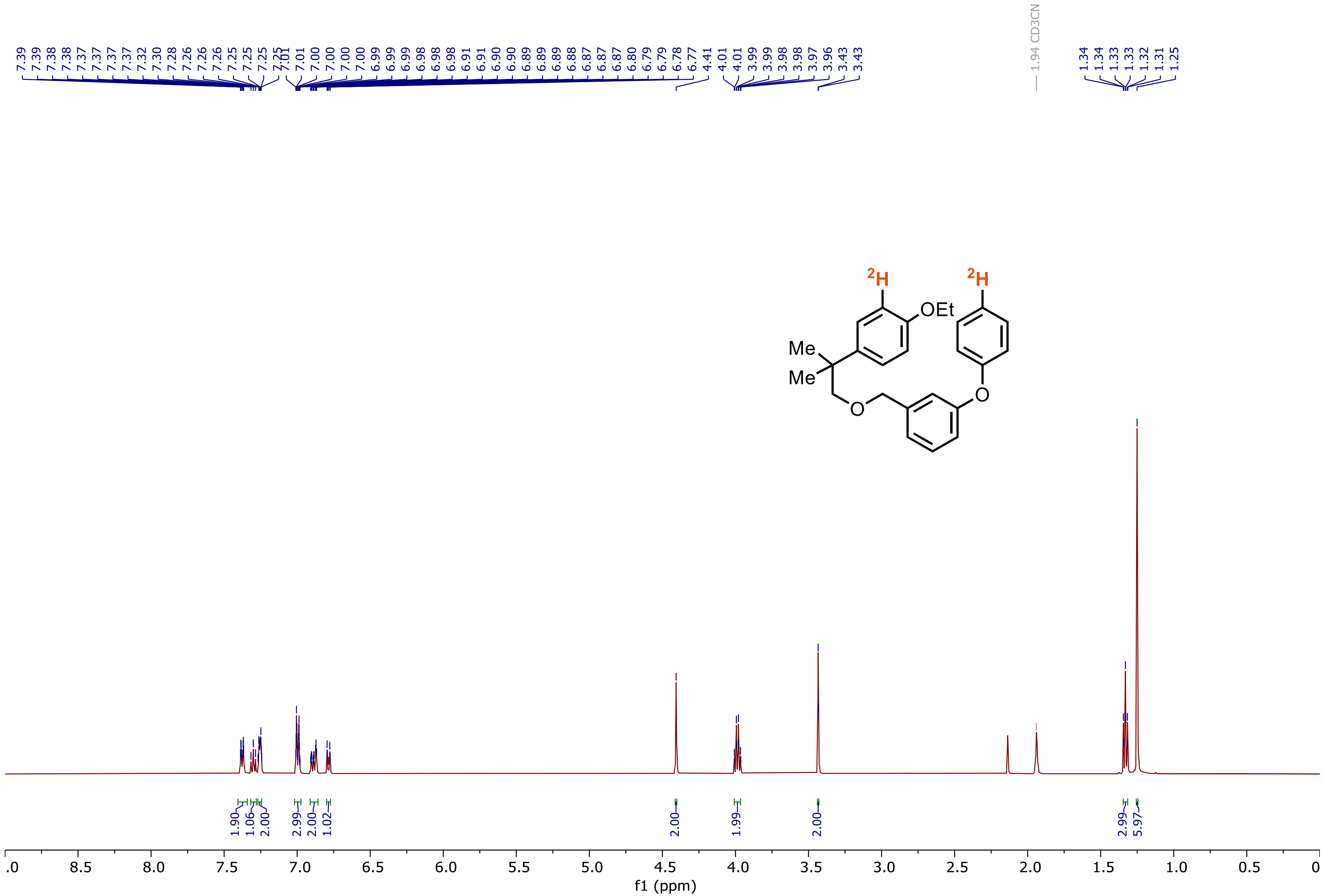

^{2}H NMR of [$^2H_2$]etofenprox ([$^2H_2$]29)

CH_3CN, 23 °C

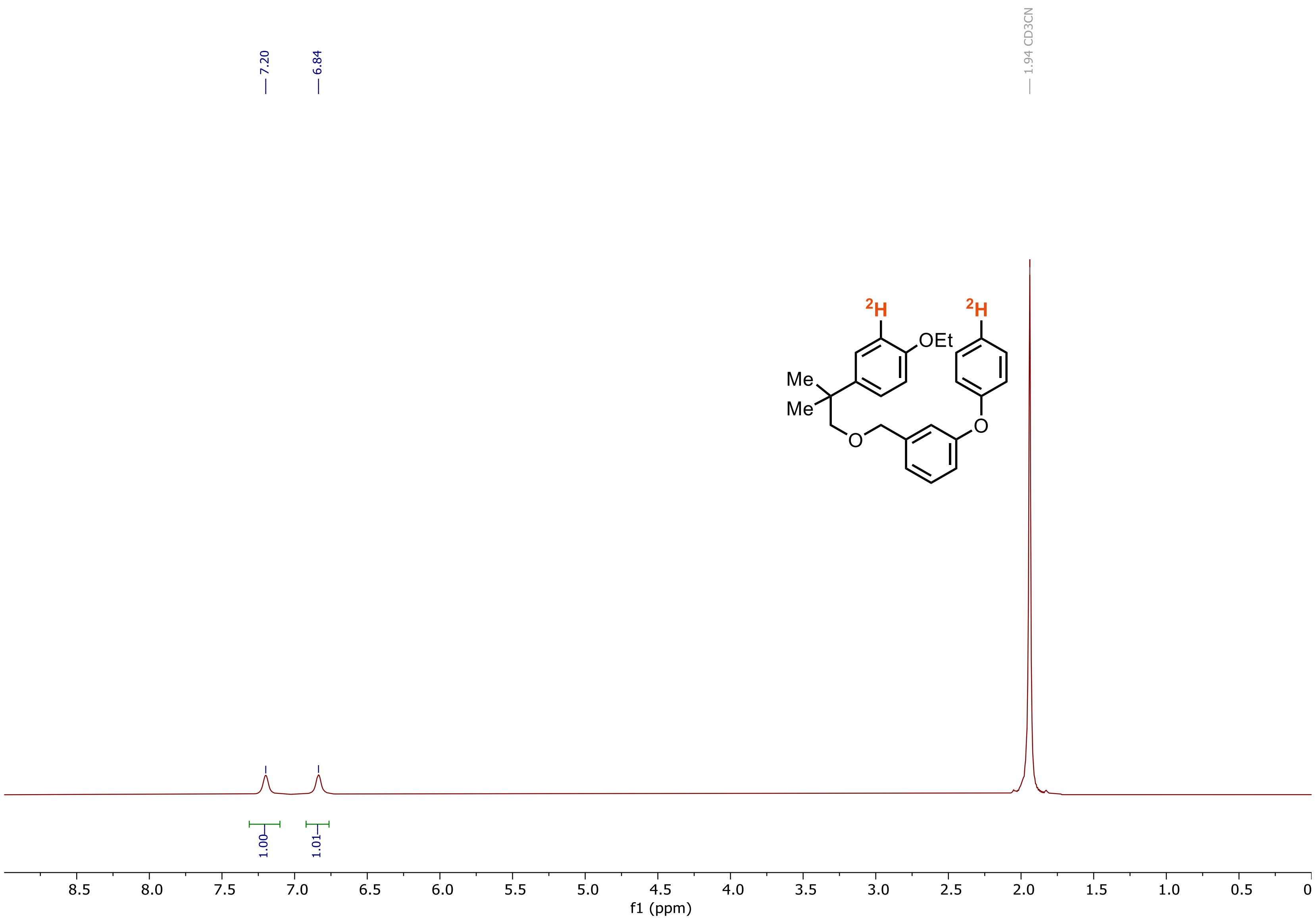

^{13}C NMR of [$^2H_2$]etofenprox ([$^2H_2$]29)

CD_3CN, 23 °C

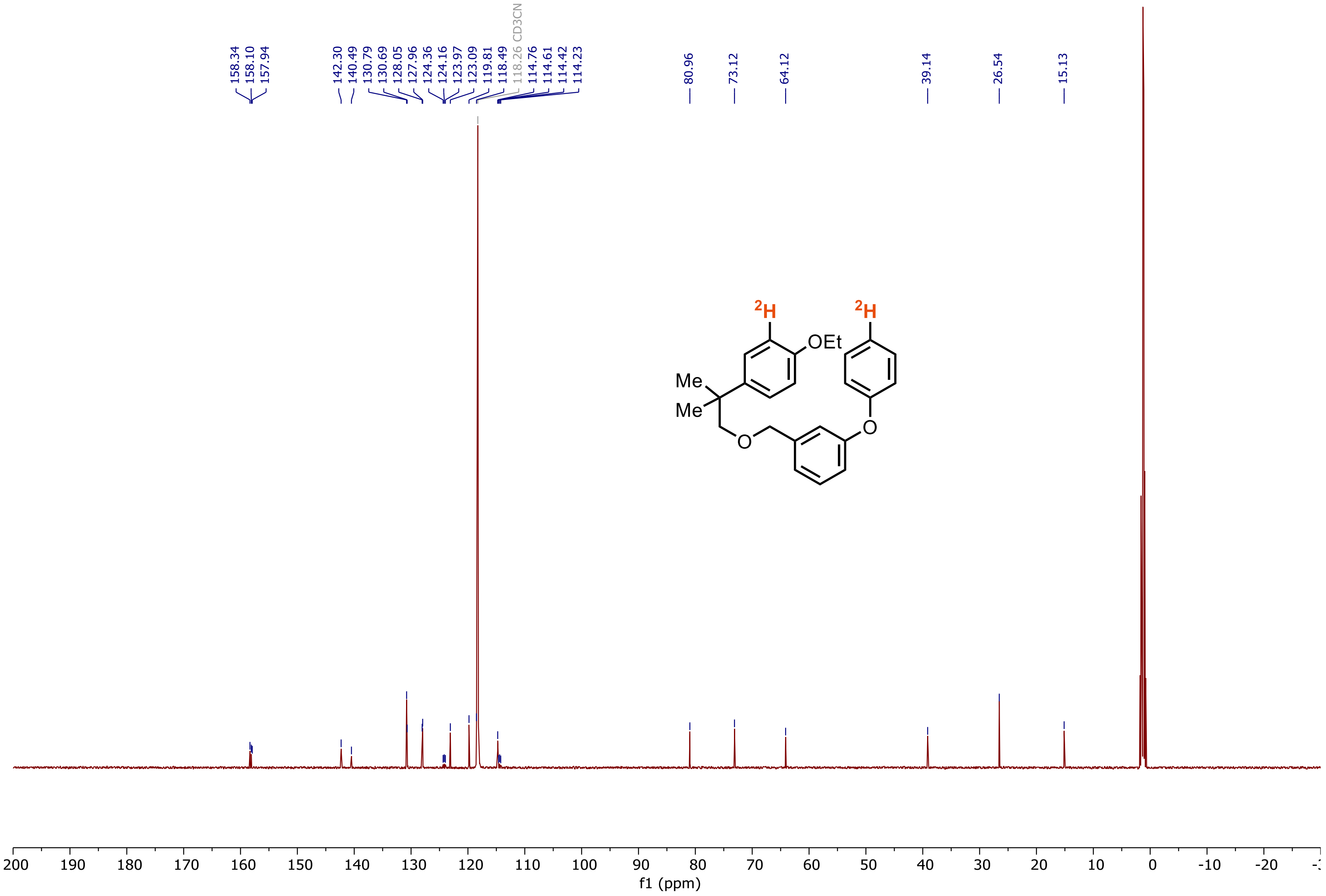

^{1}H NMR of [$^2H_2$]benazepril methylester triflimide adduct ([^{2}H]30)

CD_3CN, 23 °C

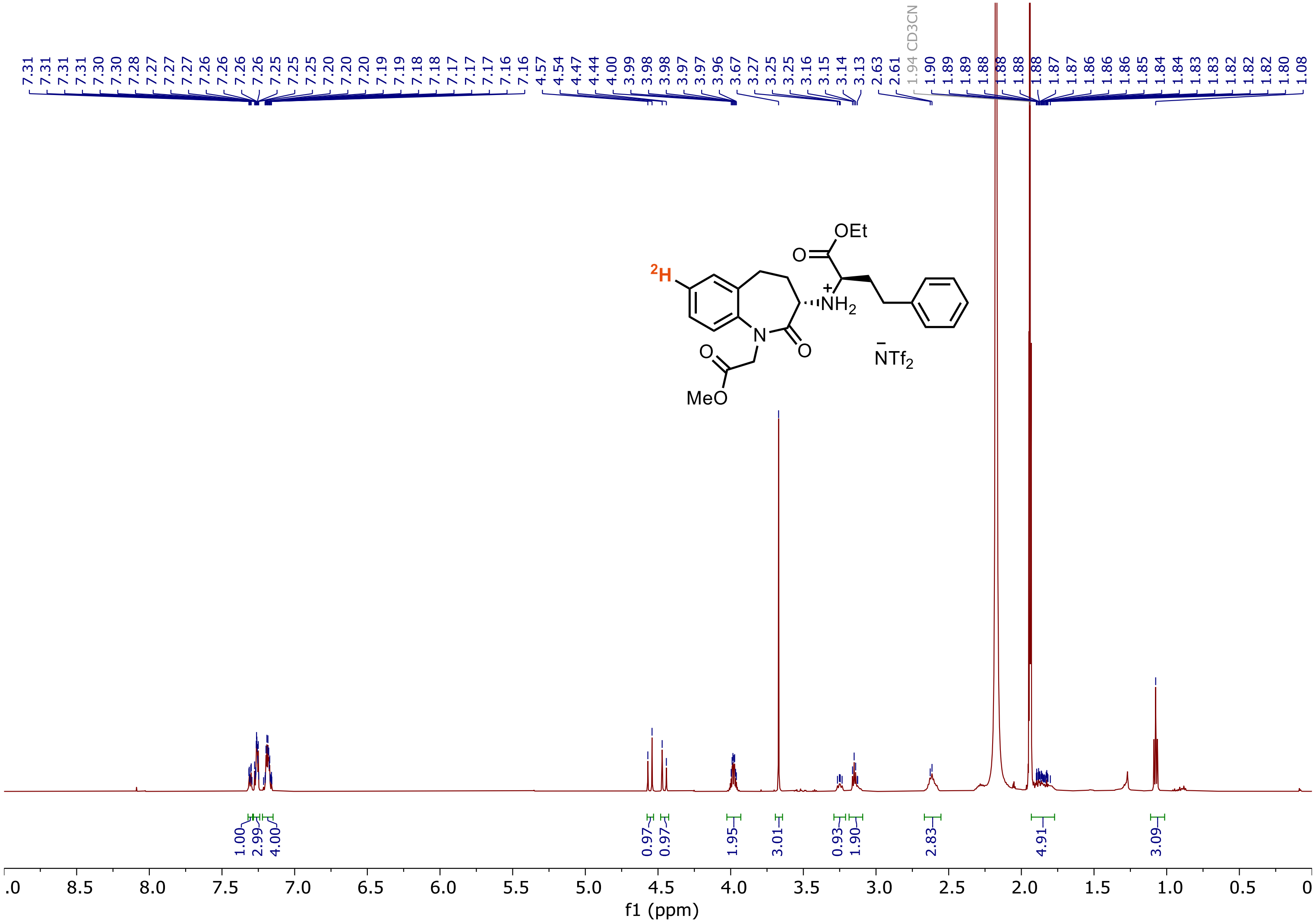

^{2}H NMR of [$^{2}H_2$]benazepril methylester triflimide adduct ([^{2}H]30)

CH_3CN, 23 °C

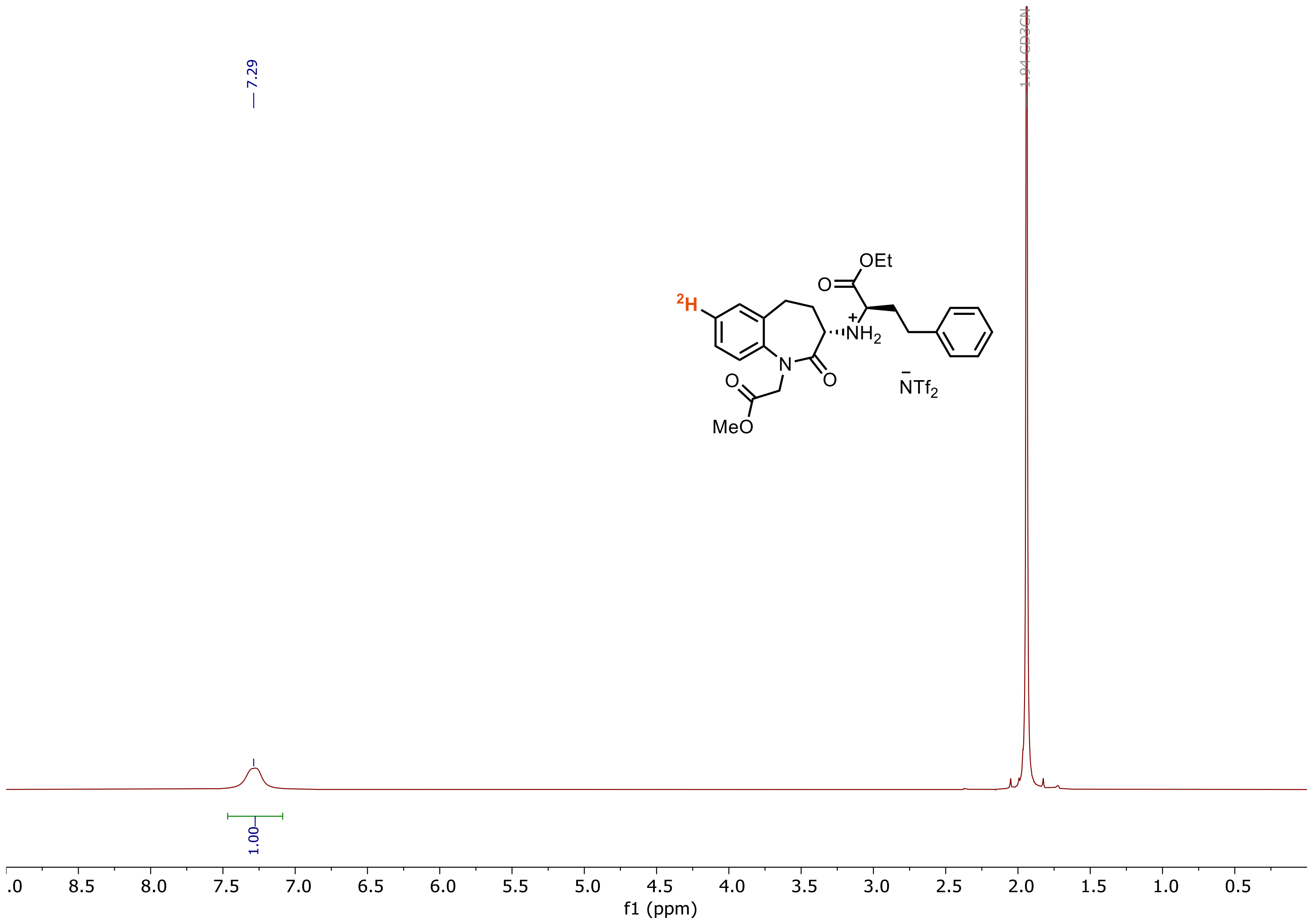

^{13}C NMR of [$^2H_2$]benazepril methylester triflimide adduct ([^2H]30)

CD$_3$CN, 23 °C

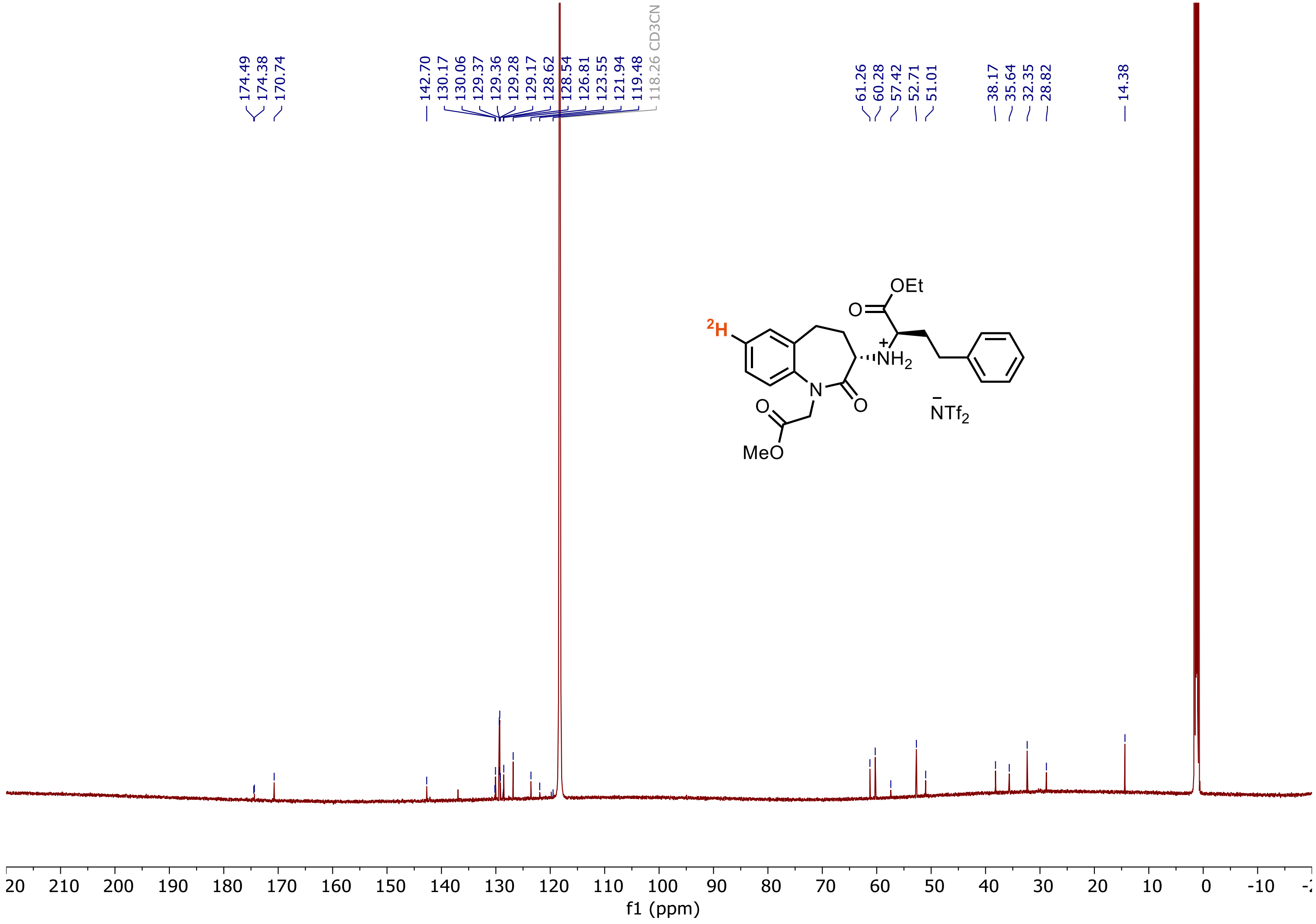

^{19}F NMR of [$^{2}H_2$]benazepril methylester triflimide adduct ([^{2}H]30)

CH_3CN, 23 °C

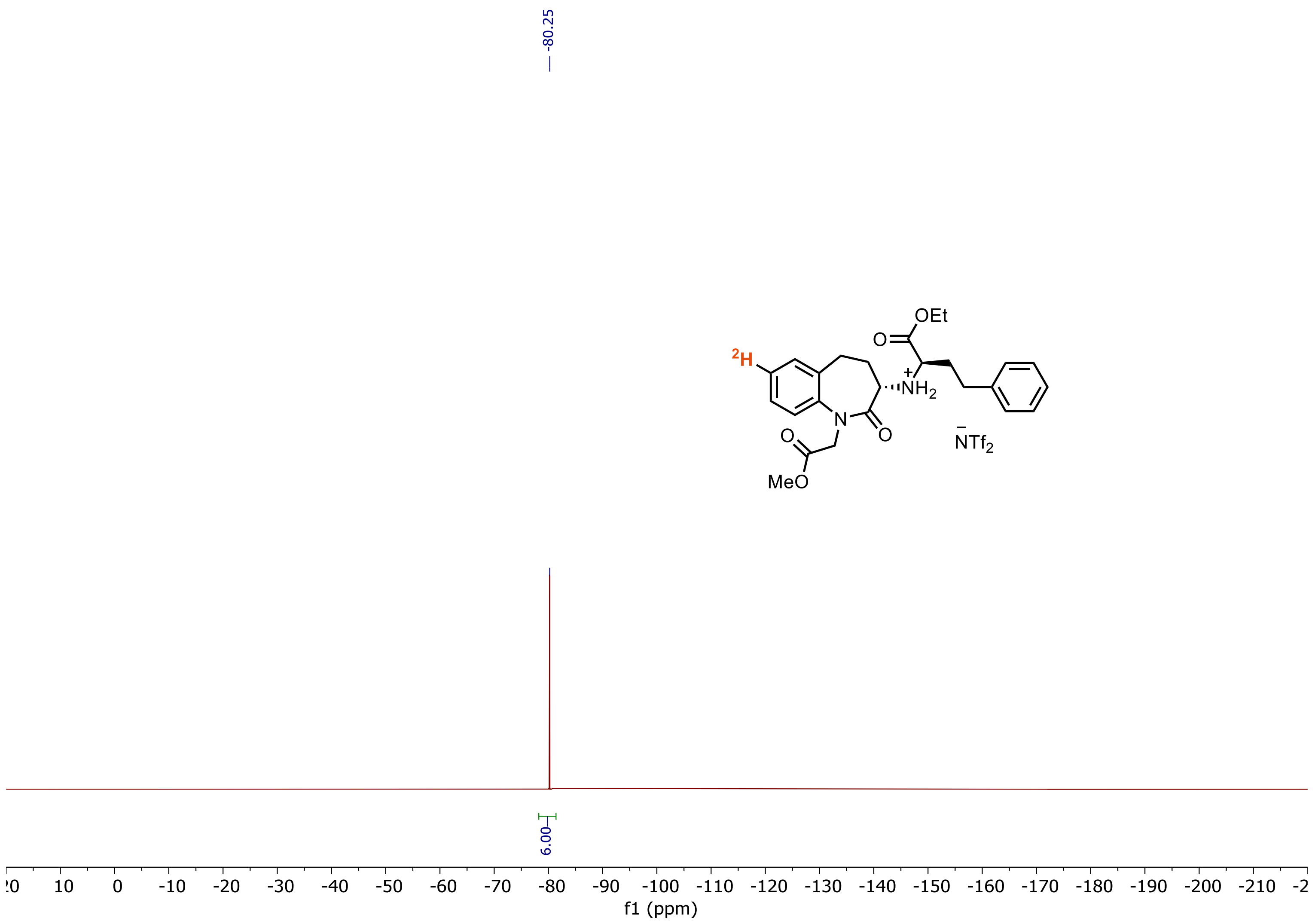

REFERENCES

1. Fulmer, G. R. *et al. Organometallics* **29**, 2176–2179 (2010).

2. Berger, F. *et al. Nature* **567**, 223–228 (2019).

3. Engl, P. S. *et al.* C–N Cross-couplings for site-selective late-stage diversification via aryl sulfonium salts. *J. Am. Chem. Soc.* **141**, 13346–13351 (2019).

4. Durr, A. B., Yin, G. Y., Kalvet, I., Napoly, F. & Schoenebeck, F. Nickel-catalyzed trifluoromethylthiolation of Csp^2-O Bonds. *Chem. Sci.* **7**, 1076–1081 (2016).

5. Shu, X.-Z., Zhang, M., He, Y., Frei, H., Toste, F. D. Dual visible light photoredox and gold-catalyzed arylative ring expansion. *J. Am. Chem. Soc.* **136**, 5844–5847 (2014).

6. Burés, J. A Simple graphical method to determine the order in catalyst. *Angew. Chem., Int. Ed.* **55**, 2028−2031 (2016).

7. Stambuli, J. P., Incarvito, C. D., Bühl, M. & Hartwig, J. F. Synthesis, structure, theoretical studies, and ligand exchange reactions of monomeric, T-shaped arylpalladium(II) halide complexes with an additional, weak agostic interaction. *J. Am. Chem. Soc.* **126**, 1184–1194 (2004).

8. Lawrance, G. A. Coordinated trifluoromethanesulfonate and fluorosulfate. *Chem. Rev.* **86**, 17−33 (1986).

9. Chernichenko, K. *et al.* Metal-free sp2-C-H borylation as a common reactivity pattern of frustrated 2-aminophenylboranes. *J. Am. Chem. Soc.* **138**, 4860−4868 (2016).

10. Garhwal, S., Kaushansky, A., Fridman, N., Shimon, L. J. W. & de Ruiter, G. Facile H/D exchange at (hetero)aromatic hydrocarbons catalyzed by a stable transdihydride N-heterocyclic carbene (NHC) iron complex. *J. Am. Chem. Soc.* **142**, 17131−17139 (2020).
